# Genome-wide identification, characterization, and validation of the bHLH transcription factors in grass pea

**DOI:** 10.3389/fgene.2023.1128992

**Published:** 2023-03-20

**Authors:** Alsamman M. Alsamman, Mohamed Abdelsattar, Achraf El Allali, Khaled H. Radwan, Ahmed E. Nassar, Khaled H. Mousa, Ahmed Hussein, Morad M. Mokhtar, Mamdouh M. Abd El-Maksoud, Tawffiq Istanbuli, Zakaria Kehel, Aladdin Hamwieh

**Affiliations:** ^1^ Agricultural Genetic Engineering Research Institute (AGERI), Agricultural Research Center (ARC), Giza, Egypt; ^2^ African Genome Center, Mohammed VI Polytechnic University, Ben Guerir, Morocco; ^3^ National Biotechnology Network of Expertise, ASRT, Cairo, Egypt; ^4^ International Center for Agricultural Research in the Dry Areas (ICARDA), Giza, Egypt; ^5^ Department of Genetics, Faculty of Agriculture, Mansoura University, Dakahlia, Egypt; ^6^ International Center for Agricultural Research in the Dry Areas (ICARDA), Terbol, Lebanon; ^7^ Biodiversity and Crop Improvement Program, International Center for Agricultural Research in the Dry Areas (ICARDA), Rabat, Morocco

**Keywords:** grass pea, bHLH gene family, phylogenetic analysis, gene structure, gene expression, qPCR analysis, cis-element

## Abstract

**Background:** The basic helix-loop-helix (bHLH) transcription factor is a vital component in plant biology, with a significant impact on various aspects of plant growth, cell development, and physiological processes. Grass pea is a vital agricultural crop that plays a crucial role in food security. However, the lack of genomic information presents a major challenge to its improvement and development. This highlights the urgency for deeper investigation into the function of bHLH genes in grass pea to improve our understanding of this important crop.

**Results:** The identification of bHLH genes in grass pea was performed on a genome-wide scale using genomic and transcriptomic screening. A total of 122 genes were identified as having conserved bHLH domains and were functionally and fully annotated. The LsbHLH proteins could be classified into 18 subfamilies. There were variations in intron-exon distribution, with some genes lacking introns. The cis-element and gene enrichment analyses showed that the LsbHLHs were involved in various plant functions, including response to phytohormones, flower and fruit development, and anthocyanin synthesis. A total of 28 LsbHLHs were found to have cis-elements associated with light response and endosperm expression biosynthesis. Ten conserved motifs were identified across the LsbHLH proteins. The protein-protein interaction analysis showed that all LsbHLH proteins interacted with each other, and nine of them displayed high levels of interaction. RNA-seq analysis of four Sequence Read Archive (SRA) experiments showed high expression levels of LsbHLHs across a range of environmental conditions. Seven highly expressed genes were selected for qPCR validation, and their expression patterns in response to salt stress showed that *LsbHLHD4, LsbHLHD5, LsbHLHR6, LsbHLHD8, LsbHLHR14, LsbHLHR68*, and *LsbHLHR86* were all expressed in response to salt stress.

**Conclusion:** The study provides an overview of the bHLH family in the grass pea genome and sheds light on the molecular mechanisms underlying the growth and evolution of this crop. The report covers the diversity in gene structure, expression patterns, and potential roles in regulating plant growth and response to environmental stress factors in grass pea. The identified candidate LsbHLHs could be utilized as a tool to enhance the resilience and adaptation of grass pea to environmental stress.

## Introduction

Grass pea (*Lathyrus sativus L.*) is a highly valued legume crop due to its remarkable ability to withstand a variety of environmental conditions, making it a key player in climate-resilient agriculture (Aci et al., 2020). With a rich history of cultivation stretching across three continents, grass pea is widely used as a source of high-quality protein, especially in the arid regions where it serves as a cheap and valuable food, feed, and fodder crop (Campbell et al., 1994; Lambein et al., 2019). Its tolerance to abiotic stresses such as drought, salt, and water-logging make it a favored crop in low-input farming systems, especially in South Asia and Sub-Saharan Africa, as well as throughout the Mediterranean Basin (Chtourou-Ghorbel et al., 2002; Piwowarczyk et al., 2016; Aci et al., 2020). Its importance as a food source in the developing countries such as India, Bangladesh, Pakistan, Nepal, Afghanistan, and Ethiopia cannot be overstated (Dixit et al., 2016). With its diploid genome (2n = 14, 8.12 Gbp), the analysis of its genome holds the key to unlocking numerous benefits of grass pea and filling the gap in food production (Almeida et al., 2015). In particular, studying its genome structure will provide valuable insights into the gene families controlling economically relevant traits.

Plant growth and survival are susceptible to biotic and abiotic stresses, especially in harsh environmental conditions such as dry areas. To overcome these challenges, plants employ a range of physiological and biochemical mechanisms, which are regulated by transcription factors (TFs) (Sun et al., 2015). These TFs, including MYBs (Jin and Martin, 1999), bHLHs (basic helix-loop-helix) (Massari and Murre, 2000), ERFs (ethylene responsive element binding factor) (Rehman and Mahmood, 2015), bZIPs (basic region/leucine zipper motif) (Corrêa et al., 2008), and WRKYs (Zhang and Wang, 2005), play a crucial role in plant defense mechanisms against environmental stress (Rehman and Mahmood, 2015). Studying gene expression in plants, particularly those grown in dry areas and subjected to various biotic and abiotic stresses, sheds light on the significance of gene families and TFs in physiological and biochemical processes (Sun et al., 2015), and helps to uncover the mechanisms involved in these processes.

The Basic Helix-Loop-Helix (bHLH) protein family is one of the largest and most important transcription factor families in plants. It plays a crucial role in controlling growth, development, and stress response by regulating gene expression (Rehman and Mahmood, 2015; Zhou et al., 2020). The bHLH motif was first discovered in the study of muscle growth in mice (Mao et al., 2017). The bHLH proteins are characterized by their highly conserved alkaline/helix-loop-helix domains, which give them their name (Yin et al., 2015). Based on sequence similarity, evolutionary relationship, and DNA binding ability, bHLH proteins are generally classified into six major groups (A to F) (Zhou et al., 2020). The exact number of groups in plants is still not well defined, but it is estimated to be between 15 and 26 (Pires and Dolan, 2010). Some studies have increased that number to 32 through genetic analysis of typical bHLH proteins (Carretero-Paulet et al., 2010). Its domain typically contains 50–60 amino acids and can be divided into two functional segments: the N-terminal basic domain, which binds the transcription factor to DNA at a consensus hexanucleotide sequence, and the C-terminal, which is made up of two *α*-helices joined by a loop. The BHLH domain can form dimers with different bHLH transcription factors through homo- or heterodimerization, leading to a range of diverse activities (Wang et al., 2018; Shen et al., 2019). The sequence variation of bHLH genes across plant species may shed light on its functionality and the biological processes it controls.

The study of the bHLH gene family structure in grass pea is crucial in gaining a deeper understanding of its evolution and gene functionality. While systematic identifications of bHLH genes have been conducted in various plant species, including apple, mango, potato, tomato, and sweet cherry, there is a lack of information on the structure, function, and activity of it in grass pea. This study aims to address this gap and uncover the secrets of bHLH functional proteins in grass pea. The phylogenetic analysis was employed to investigate the classification of bHLH genes across the genome. The functional biological properties, structures, and conserved domains of these genes were also analyzed. Furthermore, the protein-protein interaction network and sequence similarity with other plants were used to predict their interaction mechanisms. The expression levels of transcripts were analyzed using RNA-seq differential gene expression analysis, which was conducted on published data from various environmental conditions. To validate the activity and expression of some of the reported bHLH genes, real-time PCR was used in the laboratory to confirm their viability and significance. Our study highlights the importance of these genes in performing their key roles in grass pea and other plants, particularly in improving the crop’s resilience in dry areas and challenging climatic conditions. This research is expected to contribute to a better understanding of the role of bHLH genes in plant growth and survival, especially in the context of environmental stresses.

## Materials and methods

### Genomic data sources

The genomic data of grass pea genotype LS007 was obtained from the NCBI database (https://www.ncbi.nlm.nih.gov, NCBI ID: CABITX010000000). The genome assembly size is 8.12 Gbp with a coverage of 60X, an N50 of 59,728 bp, and a total of 669,893 sequences, as reported in Emmrich et al. (2020). To enhance the genomic data, transcriptomic sequences were obtained from NCBI (https://www.ncbi.nlm.nih.gov/bioproject/PRJNA258356), totaling 103.3 Mbp of sequences. To identify the bHLH genes in grass pea, a total of 7,021 amino acid sequences of bHLH genes from various plant species were obtained from NCBI. For categorization purposes, sequences of bHLH genes and their classification information were taken from previously published studies on apple (*Malus domestica*) (*MdbHLH*) (Mao et al., 2017) and *Arabidopsis thaliana* (*AtbHLH*) (Zhang et al., 2020).

### Annotation of bHLH gene family

The identification of bHLH genes in grass pea was carried out through a combination of sequence alignment, protein domain identification, and annotation techniques. Local tblastn was utilized to extract LsbHLH genes from the grass pea genome based on their similarity (90%) and sequence length (600 nucleotides) to the bHLH genes obtained from other plant species (Seppey et al., 2019). TransDecoder (Release v5.5.0) (Bryant et al., 2017) was applied to predict possible coding regions (https://github.com/TransDecoder/TransDecoder). HMMER (v3.1b2; http://hmmer.org) was used to search for the presence of bHLH domains in the grass pea proteins generated by TransDecoder. A Hidden Markov Model (HMM) file of the LsbHLH domains (PF00010) was obtained from the Pfam database (https://pfam.xfam.org) and used as a query against the grass pea proteins. The results of this search were then verified using the NCBI CDD tool (https://www.ncbi.nlm.nih.gov/Structure/cdd/wrpsb.cgi) to confirm the domain structure and to provide further information about the identified bHLH domains. Augustus (v3.3; https://bioinf.uni-greifswald.de/augustus/) was used to generate annotation files (GFF) for the LsbHLHs (Kapli et al., 2020).

### Phylogenetic analysis of bHLH protein

A phylogenetic analysis was performed to determine the relationships among the predicted genes and classify LsbHLH proteins into subfamilies. The alignment of grass pea bHLH proteins with previuosly classified Apple (*Malus domestica*) and Arabidopsis (*Arabidopsis thaliana*) bHLH proteins was conducted using the multiple sequence alignment tool MUSCLE (Edgar, 2004) (v3.8.1551; https://www.drive5.com/muscle/) with four iterations. The phylogenetic tree was constructed using Randomized Axelerated Maximum Likelihood (RAXML) (Guindon et al., 2010) with the “PROTGAMMAWAG” model, and the best likelihood was selected among the ten maximum likelihood trees (-N 10) generated from diverse starting points. The optimal number of bootstrap replicates was automatically determined by RAXML. The interactive Tree of Life (iTOL) (Letunic and Bork, 2007) online tool was used to visualize the resulting phylogenetic tree.

### Gene structure and conserved motifs identification

Characterization of gene structure and motifs was conducted to identify the gene structure and features and predict its potential function. The MEME software v5.3.1 (https://meme-suite.org/meme/) was used for motif identification, using the following parameters: minimum width of 6, maximum width of 50, and 10 optimum motifs. The predicted protein sequences of the annotated genes were extracted from the GFF file. The Gene Structure Display Server (GSDS) (http://gsds.gao-lab.org) was used to visualize the schematic structures of the intron-exon regions, conserved domains, and motifs generated by MEME. The conserved domains were retrieved from NCBI CDD (https://www.ncbi.nlm.nih.gov/Structure/cdd/wrpsb.cgi).

### Protein–protein interaction network prediction, gene ontology, and cis-regulatory elements

The prediction of protein-protein interactions among LsbHLH proteins was conducted using String tool (https://string-db.org/), where *Cicer arietinum* was used as a plant model, with a minimum interaction score of 0.4 (Szklarczyk et al., 2015). The highly interactive genes were visualized using Cytoscape software (Kohl et al., 2011). A functional enrichment analysis table (Supplementary Table S1) was produced by the STRING tool, and the frequency of Gene Ontology terms was plotted using R programming language. The 1.5 kb upstream promoter sequences of each LsbHLH start codon were retrieved from the grass pea genome and submitted to the PlantCARE database (Lescot et al., 2002) (http://bioinformatics.psb.ugent.be/webtools/plantcare/html/) for the prediction of putative cis-elements. The mapping of identified cis-element regions of LsbHLHs was performed using TBtools (Chen et al., 2018a).

### Gene expression analysis

RNA expression analysis was performed to evaluate and visualize the mRNA transcript levels of bHLH genes, identify functional genes, and infer potential biological roles. The gene expression data was obtained from four RNA-Seq experiments retrieved from the Sequence Read Archive (SRA) database https://www.ncbi.nlm.nih.gov/sra: SRP145030 (Xu et al., 2018) with three samples from shoots and seedlings after 2, 6, and 25 days of germination, SRP045652 with two control and two inoculated samples from seedlings, SRP092875 (Hao et al., 2017) with six samples from roots, stems, and leaves, and SRP327502 with 12 samples from shoots. The samples were single-end sequencing data. Quality control of the FASTQ files was performed using FastQC (Li et al., 2015), and adapter and low-quality sequence trimming was done using Fastp (Chen et al., 2018b), fqtrim (Islam et al., 2022), and fastq-quality-filter (Fu and Dong, 2018). After the quality control, the reads were then aligned to the reference genome of the grass pea using the HISAT2 software (Pertea et al., 2016). The HISAT2 software aligns the reads to the genome while taking into account splice-sites and exon information, obtained from the GFF annotation file using python scripts included in the HISAT2 package. HTseq (Srinivasan et al., 2020) was then used to count the number of aligned reads that overlap each gene exon (Ardern et al., 2011). The mapped gene counts were then normalised using the Z-score and plotted in a heatmap using the pheatmap R package. The heatmap was combined with the previously constructed LsbHLH phylogenetic tree, providing a visual representation of the gene expression patterns across different samples.

### Quantitative PCR (qPCR)

#### Plant materials, growth conditions, and stress treatment

The experimental material consisted of grass pea seeds that were germinated and grown in a 3:1:1 mixture of nutritious soil, vermiculite, and perlite in pots. The growth conditions were maintained in a controlled greenhouse with a temperature of 25°C (day) and 18°C (night), 80% relative humidity, a photoperiod of 16 h (day) and 8 h (night), and a photosynthetically active radiation of 250 mol *m* ^2^ *s* ^−1^ provided by cool white fluorescent lamps. After 30 days of growth, the plants were subjected to salt treatment by adding sodium chloride (NaCl) at concentrations of 0, 50, 100, and 200 mM to the soil. After 72 h of treatment, three biological replicates (one leaf from each seedling) were harvested, flash frozen, and stored at −80°C.

#### RNA extraction and gene expression analysis

The total RNA was extracted from both control and salt-stressed grass pea leaves using the TRIzol reagent (Invitrogen, Carlsbad, CA, United States) according to the manufacturer’s protocol and treated with RNase-free DNase (Promega Corporation, Madison, WI, United States) to eliminate genomic DNA contamination. The quality and purity of the extracted RNA were determined using a NanoDrop 2000 spectrophotometer (Thermo Fisher Scientific, Waltham, MA), and 1.5 *μg* was used for the synthesis of cDNA using the M-MLV Reverse Transcription Kit (Promega Corporation, United States). The cDNA was then diluted to 1/10 before being used in the quantitative PCR (qPCR) analysis. The qPCR-specific primers were designed using the Primer-Quest Tool (https://www.idtdna.com/PrimerQuest/Home/Index) (Supplementary Table S8).

#### Analysis of LsbHLH expression levels

Seven LsbHLH genes were selected for qPCR analysis based on their gene expression in RNAseq data and clustering in the phylogenetic tree. We made an effort to select genes from as many different clusters as possible to ensure that we had a representative sample of the gene family. To determine the expression levels of the selected LsbHLH genes in all samples, quantitative PCR (qPCR) was performed on an Agilent Stratagene Mx3005p real-time PCR detection system using BioEasy Master Mix Plus (SYBR Green Mix) according to the manufacturer’s instructions. The reaction mixture, in a final volume of 20 *μ*l, contained 1 *μ*l of diluted cDNA, 10 *μ*l of 2x SYBR Green PCR Master Mix, and 0.4 *μ*l (10 *μ*l) of each forward and reverse primer. The amplification program consisted of initial denaturation at 95°C for 60 s, followed by 40 cycles of denaturation at 95°C for 3 s and annealing at 60°C for 40 s. A melting curve analysis was performed from 65°C to 95°C to verify the specificity of the qPCR products. The genes *ABCT* and *Elf1b* were used as internal reference genes for normalization (Zhang et al., 2022). All reactions were conducted in triplicate, and relative expression levels were calculated using the 2^−△△*Ct*^ method (Schmittgen and Livak, 2008).

## Results

### Identification and characterization of bHLH gene family

The identification of LsbHLHs was performed on a genome-wide scale using a combination of genomic and transcriptomic screening based on the reported bHLH genes from NCBI and involved the use of HMMER search and NCBI CDD analysis. A total of 130 genes were indicated as having conserved bHLH domains. Out of these, eight genes were ruled out as non-functional, while 122 were confirmed as functional bHLH genes and fully annotated. The resulting LsbHLH amino acid sequences generated by the Augustus annotation tool ranged in length from 74 to 719 amino acids, with an average length of 310.9 amino acids and approximately 60 conserved domains. The molecular weight of the sequences varied from 8.56 to 79.5 kDa and the isoelectric point values ranged from 4.09 to 11.39 (Supplementary Tables S2, 4).

### Phylogenetic and evolutionary analysis of bHLH gene family

**FIGURE 1:**
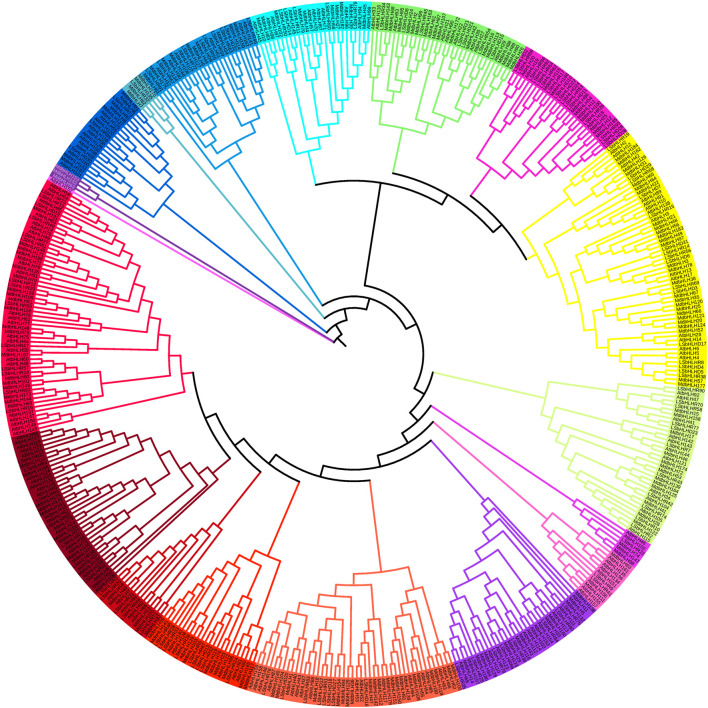
Phylogenetic tree of bHLH protein domains from *Arabidopsis thaliana*, *Manus domestica*, and grass pea. The tree was generated using the MUSCLE and RAXML algorithms, and the Maximum Likelihood method was employed with the best bootstrap replicates. The tree displays the 18 subfamilies, which are marked with distinct colors.

The sequences of the 122 LsbHLH proteins generated by Augustus were aligned with previously identified *MdbHLH* and *AtbHLH* proteins (187 and 157, respectively) (Mao et al., 2017; Wang et al., 2018) to classify the LsbHLH proteins into 18 subfamilies (Figure 1). The subfamilies ranged in size from one to fifty-seven proteins. The clustering of LsbHLHs into distinct subfamilies suggests that diverse evolutionary mechanisms drive gene differentiation. The phylogenetic analysis revealed sequence similarities and potential functional homology, which were further confirmed by gene enrichment analysis. The results revealed both similarities and differences in the classification of grass pea LsbHLH genes compared to these reference plants. Nonetheless, the presence of grass pea genes in all subgroups with sequence similarity to genes in other plant species suggests potential functional conservation. In Arabidopsis, some genes have been linked to both stomatal development and flower and fruit development, such as *AtbHLH45*, *AtbHLH97*, and *AtbHLH98* (Carretero-Paulet et al., 2010). These genes have been found to cluster with bHLH genes in grass pea, including *LsbHLHD16*, *LsbHLHD27*, *LsbHLHD18*, *LsbHLHD12*, *LsbHLHR51*, *LsbHLHR83*, *LsbHLHD20*, and *LsbHLHD76*. Similarly, genes involved in synthesizing anthocyanin in Arabidopsis such as *AtbHLH38*, *AtbHLH39*, *AtbHLH100*, and *AtbHLH101* (Zhao et al., 2011) were linked to several bHLH genes in grass pea, including *LsbHLHR68*, *LsbHLHD29*, *LsbHLHD19*, *LsbHLHD7*, *LsbHLHD31*, among others.

### Gene structure, cis-regulatory element analyses, and motif identification analyses

The gene structure analysis confirmed several key aspects of the main bHLH phylogenetic tree. The exon-intron diversity of the gene family members plays a crucial role in the evolution of multiple gene families (Xu et al., 2012). We analyzed 122 LsbHLHs to determine the intron-exon distribution and crucial protein domains in the bHLH gene family. Every gene has exons, three genes (*LsbHLHD10*, *LsbHLHD21*, *LsbHLHD8*) have four exons, four genes (*LsbHLHD11*, *LsbHLHD13*, *LsbHLHD30*, *LsbHLHD7*) have three exons, ten genes have two exons, and 105 genes are composed of a single exon. In the genes that contain introns, the number of introns ranges from one to three (*LsbHLHD10, LsbHLHD13, LsbHLHD21, LsbHLHD7*, and *LsbHLHD8*) (Supplementary Table S5). Despite intronless genes often showing elevated expression levels in response to pathogenic stimuli, they are thought to have lower response levels in plants (Wang et al., 2018). For instance, intronless genes such as (*LsbHLHR40* and *LsbHLHR1*) displayed higher expression levels in response to external stress.

**FIGURE 2:**
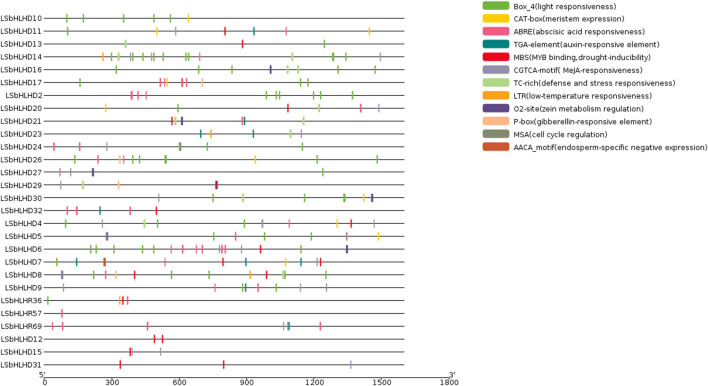
The cis-elements identified in the promoter regions of 28 bHLH genes in grass pea. The promoter regions, 1,500 bp upstream of the gene start sites, were analyzed using the PlantCARE software to predict potential cis-regulatory elements. The elements related to cell cycle regulation, meristem-endosperm expression, metabolic regulation, and responses to various plant hormones (abscisic acid, auxin, methyl jasmonate, gibberellic acid, and salicylic acid) and environmental stimuli (anaerobic induction, light, low temperature, and drought) are depicted in different colors.

**FIGURE 3:**
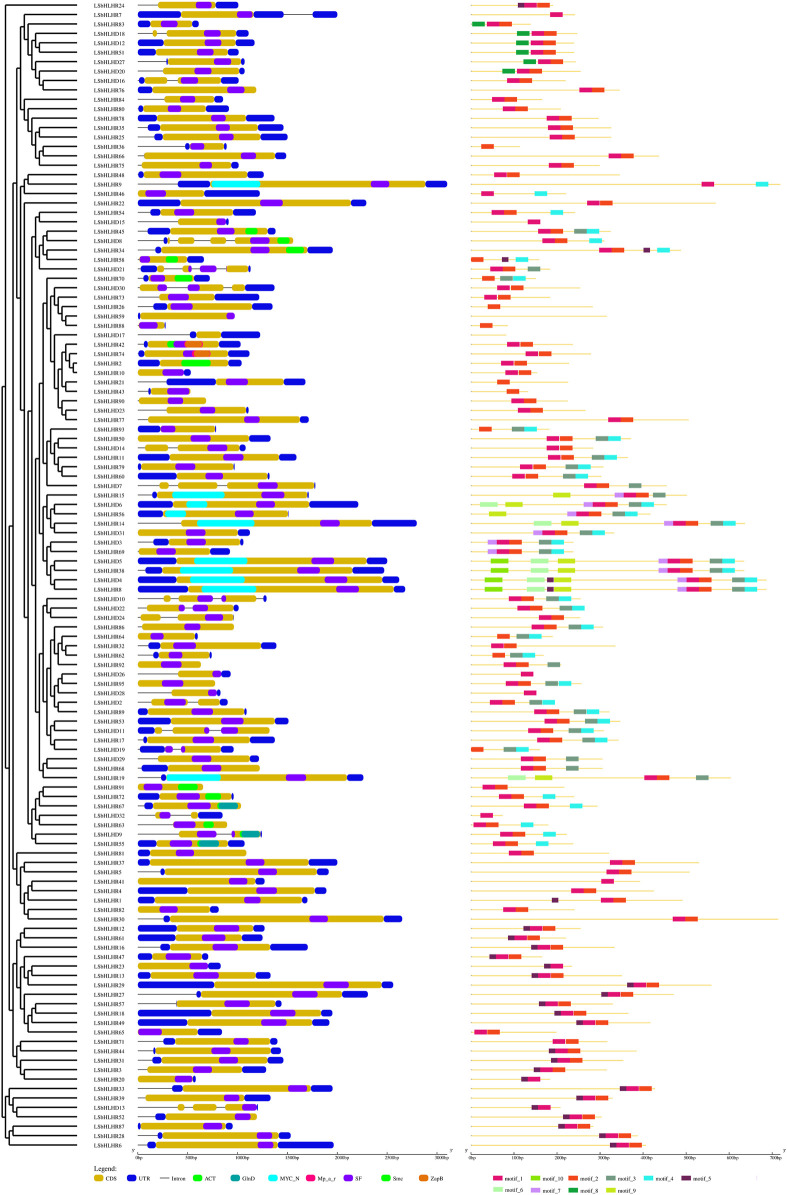
The gene structure, conserved motifs, and phylogenetic relationships of LsbHLH genes from grass pea. A phylogenetic tree of 122 LsbHLH proteins, their exon-intron organization, and transcription factor (TF) domains retrieved from NCBI-CDD are presented. Additionally, the arrangement of ten conserved motifs in the LsbHLH proteins is depicted, each represented by a different color box.

The analysis of cis-elements in the LsbHLHs of grass peas reveals their involvement in various plant functions, including cell cycle regulation, expression in meristems and endosperms, regulation of metabolism, and response to phytohormones, abiotic factors, and defense stress. These cis-elements have been grouped into three categories based on their functional roles (Figure 2). The first category includes elements related to cell cycle regulation and expression in meristems and endosperms, as well as regulation of metabolism, such as MSA, AACA_motif, CAT-box, and O2-site. The second category encompasses phytohormone-related cis-elements, including ABRE, TGA, P-box, and CGTCA-motif, and the third category encompasses elements related to abiotic factors such as defense stress, light response, drought inducibility, and low-temperature response, including TC-rich, Box_4, MBS, and LTR. The presence of essential Cis element domains, including MYC (Boter et al., 2004), ACT (Journot-Catalino et al., 2006), ZAPB (Takatsuji, 1998), and SMC (Schubert, 2009) (Figure 3 and Supplementary Table S6), confirms their involvement in key plant processes such as growth, gene expression regulation, and defence responses. Previous studies (Qian et al., 2019; Zhou et al., 2020) have reported the remarkable similarity of these domains to those found in our study, further supporting their importance.

The analysis of the amino acid motifs in the LsbHLH gene family revealed the presence of ten distinct sequence patterns. The identification of these motifs provides valuable information about the molecular interactions, localization, and regulation of the corresponding proteins. The number of motifs present in each LsbHLH gene varied from zero to nine (Supplementary Table S7). The lengths of the motifs range from 10 to 42 amino acids. The results of the LsbHLHs phylogenetic analysis demonstrate that proteins with similar sequences also possess similar amino acid motif structures. This highlights the importance of motif analysis in understanding the functions of proteins and in predicting the functions of homologous proteins in other organisms.

### Protein-protein interaction and GO annotation analysis

**FIGURE 4:**
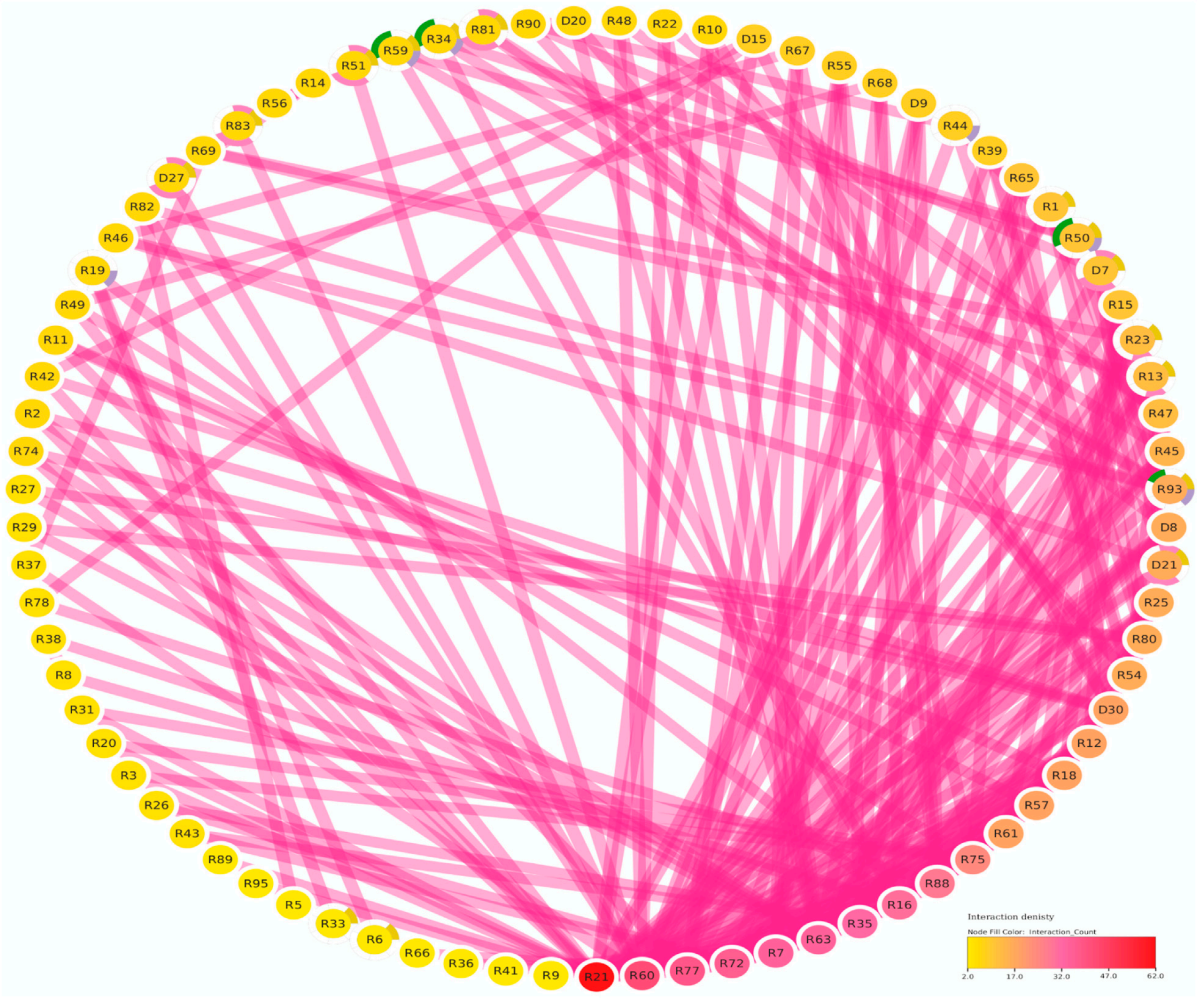
The Functional Regulatory Network of Grass Pea (LsbHLH) Proteins. The figure presents a visual representation of the protein-protein interactions among 79 grass pea LsbHLH proteins, predicted using the STRING database and visualized using Cytoscape software. The Interaction Count panel displays the number of interactions each gene has, with *R21* being the most interactive gene. Gene names are abbreviated by removing the “LsbHLH” prefix for clarity. The color-coding of the nodes provides insights into the biological functions of the genes. Green borders indicate involvement in stomatal lineage progression; yellow indicates involvement in embryonic development; blue indicates involvement in plant epidermis development; pink indicates involvement in flower development.

The protein-protein interaction (PPI) analysis showed a significant level of interaction between the bHLH sequences. The gene annotation revealed that several of the LsbHLH proteins exhibit high levels of interaction, as illustrated by nine genes (*LsbHLHR21*, *LsbHLHR60*, *LsbHLHR77*, *LsbHLHR72*, *LsbHLHR7*, *LsbHLHR63*, *LsbHLHR35*, *LsbHLHR16*, *LsbHLHR88*) demonstrating substantial interaction (Figure 4). Gene Ontology (GO) annotation revealed various crucial functions for the bHLH gene family in grass pea. Of the 92 genes that were annotated, most had significant roles in molecular functions, biological processes, or cellular components. According to the GO Cellular Component, the majority of the annotated genes were found to be important components of the nucleus. Biological Process annotated genes play important roles in the development of various plant systems, including 16 genes involved in plant system development, eight genes involved in flower development, and four genes involved in stomal development. The molecular function annotated genes were found to play crucial roles in protein dimerization activity and DNA binding.

**FIGURE 5:**
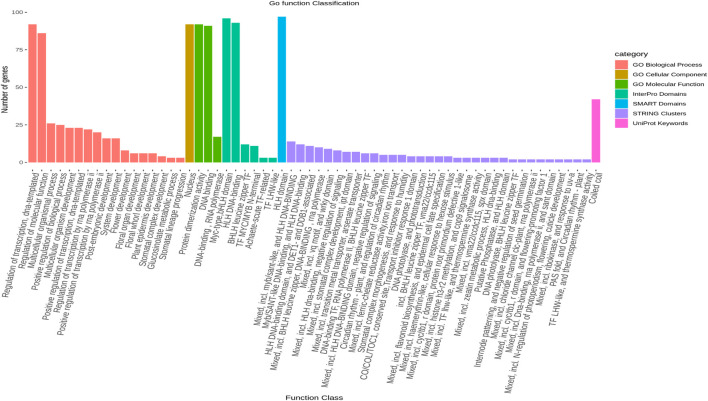
The gene ontology (GO) analysis of LsbHLH proteins was conducted in seven distinct categories, including Go Biological Process, Go Cellular Component, Interpro Domains, SMART Domains, STRING Clusters, UniProt, and Go Molecular Functions. The figure represents the number of genes involved in each gene ontology term as a bar graph.

The results of a gene ontology analysis revealed that seven significant genes (*LsbHLHR21*, *LsbHLHR60*, *LsbHLHR63*, *LsbHLHR72*, *LsbHLHR16*, *LsbHLHR77*, *LsbHLHR35*) are involved in multiple biological processes and molecular functions (Figure 5).

### Differential expression analysis of LsbHLHs

**FIGURE 6:**
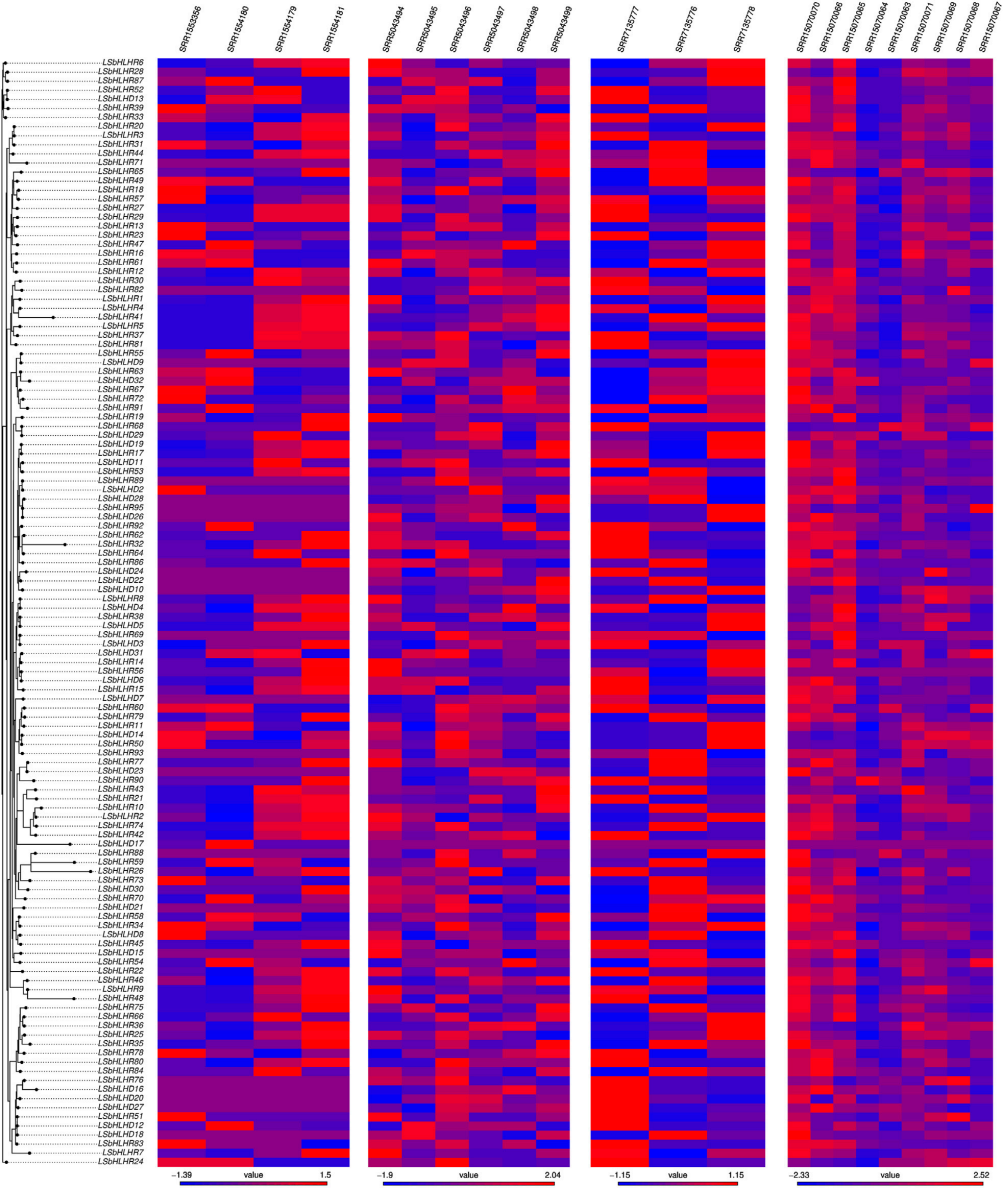
Heatmap represents the normalized count of mapped reads for the 122 identified LsbHLH genes, organized based on their phylogenetic relationships. The data was derived from four RNA-seq experiments conducted on grass pea under different conditions, each containing multiple runs or samples. The accession number for each experiment is displayed above the corresponding plot. The color scale represents the level of gene expression, with red indicating high expression and blue indicating low expression.

RNA-seq analysis was employed to validate and confirm the activity of the identified genes and assess the transcript levels of LsbHLHs mRNA under the influence of various external factors. The high expression levels of LsbHLHs observed across a range of conditions highlight the importance of these genes in the plant’s stress response. The expression levels of genes such as (*LsbHLHR1*, *LsbHLHR5*, *LsbHLHR6*, *LsbHLHR13*, and *LsbHLHR16*) increased with the plant development stage, while expression levels of (*LsbHLHD11*, *LsbHLHR76*, *LsbHLHR83*, and *LsbHLHR30*) decreased across different developmental stages as seen in the gene expression data of SRP145030 (Figure 6). In the gene expression study focused on disease infection (SRP045652), 45 genes, including (*LsbHLHD11*, *LsbHLHR81*, *LsbHLHR83*, and *LsbHLHR76*), showed no expression, while 77 genes displayed high expression levels, particularly in inoculated samples in response to pathogens. Additionally, the bHLH genes produced different amounts of transcripts in normal conditions as seen in other gene expression data such as SRP327502 and SRP092875 (Figure 6). The results reinforce the crucial role of the 122 identified LsbHLH genes for various aspects of plant biology, particularly in plant development and stress response. The abundance of LsbHLH genes in plants highlights their importance in these processes.

### Expression profiles of LsbHLHs genes under salinity stress conditions

**FIGURE 7:**
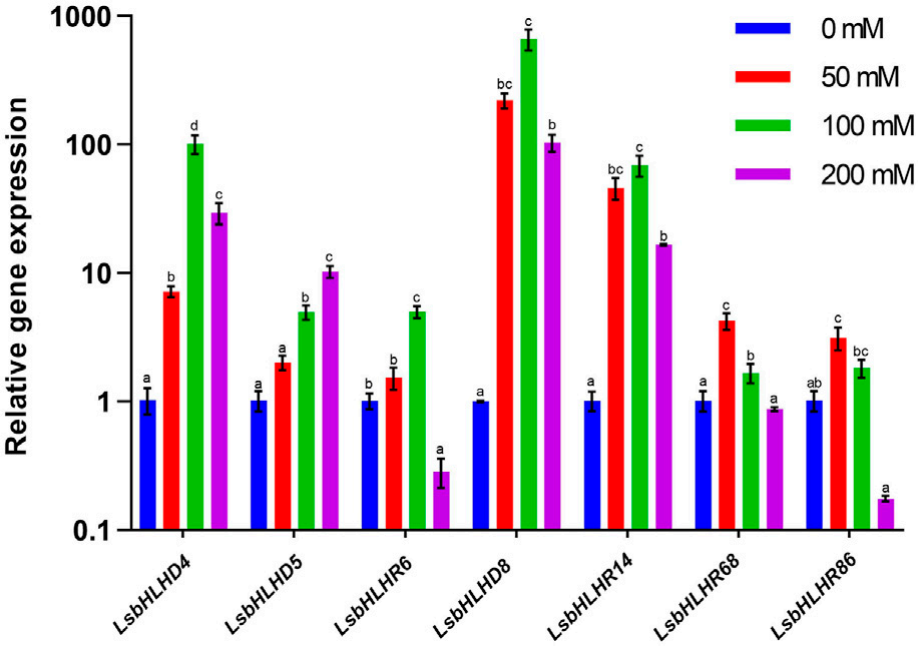
Gene expression profiles of seven selected *LsbHLH* were determined using different salt concentrations following a 72-h course. qPCR-analysis was used to determine the relative expression levels of the *LsbHLH* in seedlings treated with 50, 100, and 200 mM NaCl. Significant differences between treatments are indicated by a small letter (s) above the bars (*p* ≤0.05; LSD). The 2^−△△*Ct*^ method was used to calculate the relative expression level.

qPCR-based expression profiles of seven selected LsbHLHs genes were evaluated in response to salinity stress at different concentrations (Figure 7). Most of the genes exhibited comparatively high expression levels at 100 mM NaCl concentrations, compared to 50 and 200 mM NaCl. For example, in response to salt stress, *LsbHLHD8*, *LsbHLHD4* and *LsbHLHR14* showed significantly higher expression at 100 mM NaCl by 660, 100, and 69-fold, respectively. However, their expression levels at 200 mM NaCl were reduced by 103, 29 and 16-fold. Interestingly, the expression level of *LsbHLHD5* was lower than *LsbHLHD8*, *LsbHLHD4* and *LsbHLHR14* at 100 mM by 5-fold, while its expression levels were increased at 200 mM NaCl by 10-fold. Although the expression levels of *LsbHLHR68* and *LsbHLHR86* showed relatively higher expression at 50 mM by 4- and 3-fold, their expression levels were decreased at 100 mM NaCl by 1.7 and 1.8-fold, and then significantly decreased by 0.8 and 0.2-fold. Nevertheless, *LsbHLHR6* showed higher expression at 100 mM NaCl by 5-fold, it was severely down-regulated like the *LsbHLHR86* gene.

## Discussion

The cultivation of grass pea in areas facing numerous environmental stress factors presents a chance for the development of new food sources, especially in remote regions with inadequate nutritional options. It is crucial to ensure the suitability of grass pea as a food or forage crop through the provision of sufficient genomic information to support the development of sustainable genotypes. To this end, a comprehensive analysis of the grass pea genome was conducted to identify LsbHLH genes and associated transcription factors involved in plant cell growth, evolution, and disease resistance. The approach utilized legume bHLH genes as a reference for identifying LsbHLHs, which were then fully annotated using various bioinformatics tools to determine their functional roles and to confirm their inclusion in the bHLH gene family.

Our analysis identified 122 bHLH genes in the grass pea genome, which were classified into 18 subfamilies based on their alignment with previously identified MdbHLH and AtbHLH proteins (Figure 3). The clustering of LsbHLHs based on their protein sequence is indicative of functional homogeneity among these genes. This number of bHLH genes is similar to those found in other plant species, such as foxtail millet, where 187 bHLH genes were identified and classified into 21 subfamilies (Fan et al., 2021). Tomato also had a high number of bHLH genes, with 159 identified and classified into 21 subfamilies through phylogenetic analysis (Sun et al., 2015). In sweet cherry (*Prunus avium*), 66 bHLH genes were identified and classified into 17 subfamilies based on the classification of bHLH genes in *Arabidopsis thaliana* (Shen et al., 2021). We can draw the conclusion that our analysis has provided a comprehensive characterization of the majority of bHLH genes present in the grass pea genome. However, it is possible that some bHLH genes may have gone unnoticed due to their presence in not yet sequenced regions of the genome or as a result of their exclusion due to being partial or incomplete in nature.

The study of gene structure and protein domains is essential to comprehend the function of transcription factors and their impact on plant metabolism. The presence of exons and introns and their distribution play a crucial role in gene family evolution (Xu et al., 2012). In our study, the number of introns in the identified bHLH genes in grass pea ranged from one to four, which is comparable to what was reported in rice (Li et al., 2006), and fewer than what was reported in potatoes (Wang et al., 2018), *Ginkgo biloba* (Zhou et al., 2020), and apples (Yang et al., 2017). These findings highlight the diversity in gene structure and evolution within the bHLH gene family in different plant species.

Cis-regulatory sequences are essential in development and physiology because they regulate gene expression (Wittkopp and Kalay, 2012). Several cis-element domains have been identified within the bHLH gene family in grass pea, including MYC, ACT, ZAPB, and SMC. These domains, which have been studied extensively, are critical in plant activities such as stomatal growth and anthocyanin synthesis. For instance, in Dahlias, the bHLH transcription factor *DvIVS* was found to regulate anthocyanin synthesis and its expression was reduced in yellow ray florets (Ohno et al., 2011). The MYB-bHLH-WDR complex was identified as a central regulator of the anthocyanin synthesis pathway in Kiwifruit (Liu et al., 2021). In apples, the cold-induced bHLH transcription factor *MdbHLH3* was observed to interact with *MdMYB1* and promote anthocyanin accumulation and fruit coloration in response to low temperature (Xie et al., 2012). The SCREAM ACT-like domain has been found to play a role in bHLH partner selection during stomatal differentiation (Seo et al., 2022). The novel bHLH transcription factor *PebHLH35* from *Populus euphratica* has been shown to positively regulate drought stress responses through regulation of stomatal density, aperture, photosynthesis, and growth (Dong et al., 2014). Our findings could inspire researchers to further study the potential of these bHLH genes as a tool to improve the resilience and adaptation of grass pea to environmental stresses, such as drought and cold. Additionally, our analysis revealed ten unique gene sequence motifs in the identified bHLH genes in grass pea (Figure 3). Notably, three of these motifs were present in all genes, lending them significant and distinctive features. The number of motifs in bHLH gene families varies between different plant species, with twelve reported in apples (Mao et al., 2017), twenty in potatoes (Wang et al., 2018), ten in mangoes (Salih et al., 2021), and twenty in *Ginkgo biloba* (Zhou et al., 2020). These gene motifs offer valuable information about its evolutionary history and can serve as targets for further studies aimed at understanding the role of these genes in plant metabolism.

The analysis of protein-protein interactions and gene enrichment is crucial for understanding the activity of bHLH proteins, which can form homodimers or heterodimers to bind DNA and control transcription (Salih et al., 2021). The interaction network of 122 candidate LsbHLHs was studied using *Cicer arietinum* as a model organism due to the absence of biological pathway information for grass pea and the similarity between chickpea and grass pea as they belong to the same family. This resulted in a complex network with a high degree of consistency and interactive genes, indicating the important role these genes play in plant biology (Figure 5). Many of these genes were associated with plant system development, flowering, stomal development, protein dimerization, and DNA binding, which supports the conclusions drawn from our results and previous studies in other plant species. For example, some bHLH genes, such as *FLOWERING BHLH 4 (FBH4)*, are known regulators of nitrogen-responsive flowering in Arabidopsis; nitrogen levels were found to fine-tune *FBH4* nuclear localization and modulate flowering time by adjusting its phosphorylation state (Sanagi et al., 2021).

We confirmed the gene expression and activity of the 122 candidate LsbHLH genes in grass pea through the analysis of published expression data and laboratory-based qPCR experiments (Figures 6, 7). Four distinct RNA experiments were used to evaluate the expression of LsbHLH genes under different environmental conditions and to ensure that they are expressed and have a potential function. The results from these experiments showed that the expression levels of bHLH genes increased with plant age, which supports the impact of bHLH on plant growth and development. Additionally, some genes showed increased expression in infected samples, indicating that pathogen resistance is one of the bHLH gene family roles. It is important to note that genes with no expression in our data sets may still play a role in different contexts or have an expression that is undetectable. Additionally, the expression profiles of seven selected genes from the LsbHLH family were analyzed in response to different concentrations of sodium chloride (NaCl). Specifically, genes *LsbHLHD4*, *LsbHLHD5*, *LsbHLHR6*, *LsbHLHD8*, and *LsbHLHR14* displayed higher expression in response to salinity stress. These results align with previous studies, which reported increased expression of several basic helix-loop-helix (bHLH) genes under salinity stress. For instance, in the case of jojoba, the *JcbHLH8* gene responded significantly to salinity after being exposed to 100 mM NaCl for two and 7 days (Zhang et al., 2020). Similarly, the *JcbHLH6* gene responded significantly to 100 mM NaCl for 2 days (Zhang et al., 2020). In the case of Hibiscus, genes *HhbHLH6* and *HhbHLH8* showed significant response to 400 mM NaCl after 24 h (Ni et al., 2021). In green chiretta, *ApbHLH5* and *ApbHLH6* showed significant up-regulation in response to 50 and 100 mM NaCl for 5 days, whereas the expression level of the *ApbHLH68* gene showed down-regulation at the same concentrations (Xu et al., 2022). On the other hand, the expression level of *LsbHLHR86* was consistent with previous reports, while the expression level of the *CsbHLH86* gene in cucumber showed significant downregulation after exposure to 100 mM NaCl for 24 h (Li et al., 2020). Interestingly, our results revealed an opposite pattern for genes *LsbHLHD4* and *LsbHLHR14* compared to other studies that did not show significant upregulation clearly (Xu et al., 2022; Zhang et al., 2022). Overall, these results provide a solid foundation for further studies on the role of the bHLH gene family in grass pea.

## Data Availability

The original contributions presented in the study are included in the article/Supplementary Material, further inquiries can be directed to the corresponding authors.

## References

[B1] Aci M. M. Lupini A. Badagliacca G. Mauceri A. Lo Presti E. Preiti G. (2020). Genetic diversity among lathyrus ssp. based on agronomic traits and molecular markers. Agronomy 10, 1182. 10.3390/agronomy10081182

[B2] Almeida N. F. Krezdorn N. Rotter B. Winter P. Rubiales D. Vaz Patto M. C. (2015). Lathyrus sativus transcriptome resistance response to ascochyta lathyri investigated by deepsupersage analysis. Front. plant Sci. 6, 178. 10.3389/fpls.2015.00178 25852725 PMC4367168

[B3] Ardern C. L. Webster K. E. Taylor N. F. Feller J. A. (2011). Return to sport following anterior cruciate ligament reconstruction surgery: A systematic review and meta-analysis of the state of play. Br. J. sports Med. 45, 596–606. 10.1136/bjsm.2010.076364 21398310

[B4] Boter M. Ruíz-Rivero O. Abdeen A. Prat S. (2004). Conserved myc transcription factors play a key role in jasmonate signaling both in tomato and arabidopsis. Genes and Dev. 18, 1577–1591. 10.1101/gad.297704 15231736 PMC443520

[B5] Bryant D. M. Johnson K. DiTommaso T. Tickle T. Couger M. B. Payzin-Dogru D. (2017). A tissue-mapped axolotl de novo transcriptome enables identification of limb regeneration factors. Cell Rep. 18, 762–776. 10.1016/j.celrep.2016.12.063 28099853 PMC5419050

[B6] Campbell C. Mehra R. Agrawal S. Chen Y. Abdel Moneim A. Khawaja H. (1994). “Current status and future strategy in breeding grasspea (lathyrus sativus),” in Expanding the production and use of cool season food legumes (Springer), 617–630.

[B7] Carretero-Paulet L. Galstyan A. Roig-Villanova I. Martínez-García J. F. Bilbao-Castro J. R. Robertson D. L. (2010). Genome-wide classification and evolutionary analysis of the bhlh family of transcription factors in arabidopsis, poplar, rice, moss, and algae. Plant physiol. 153, 1398–1412. 10.1104/pp.110.153593 20472752 PMC2899937

[B8] Chen C. Chen H. He Y. Xia R. (2018a). Tbtools, a toolkit for biologists integrating various biological data handling tools with a user-friendly interface. BioRxiv, 289660.

[B9] Chen S. Zhou Y. Chen Y. Gu J. (2018b). fastp: an ultra-fast all-in-one fastq preprocessor. Bioinformatics 34, i884–i890. 10.1093/bioinformatics/bty560 30423086 PMC6129281

[B10] Chtourou-Ghorbel N. Lauga B. Ben Brahim N. Combes D. Marrakchi M. (2002). Genetic variation analysis in the genus lathyrus using rapd markers. Genet. Resour. Crop Evol. 49, 365–372. 10.1023/a:1020639625803

[B11] Corrêa L. G. G. Riaño-Pachón D. M. Schrago C. G. Vicentini dos Santos R. Mueller-Roeber B. Vincentz M. (2008). The role of bzip transcription factors in green plant evolution: Adaptive features emerging from four founder genes. PloS one 3, e2944. 10.1371/journal.pone.0002944 18698409 PMC2492810

[B12] Dixit G. P. Parihar A. K. Bohra A. Singh N. P. (2016). Achievements and prospects of grass pea (lathyrus sativus l.) improvement for sustainable food production. Crop J. 4, 407–416. 10.1016/j.cj.2016.06.008

[B13] Dong Y. Wang C. Han X. Tang S. Liu S. Xia X. (2014). A novel bhlh transcription factor pebhlh35 from populus euphratica confers drought tolerance through regulating stomatal development, photosynthesis and growth in arabidopsis. Biochem. biophysical Res. Commun. 450, 453–458. 10.1016/j.bbrc.2014.05.139 24909687

[B14] Edgar R. C. (2004). Muscle: Multiple sequence alignment with high accuracy and high throughput. Nucleic acids Res. 32, 1792–1797. 10.1093/nar/gkh340 15034147 PMC390337

[B15] Emmrich P. M. Sarkar A. Njaci I. Kaithakottil G. G. Ellis N. Moore C. (2020). A draft genome of grass pea (lathyrus sativus), a resilient diploid legume. BioRxiv.

[B16] Fan Y. Lai D. Yang H. Xue G. He A. Chen L. (2021). Genome-wide identification and expression analysis of the bhlh transcription factor family and its response to abiotic stress in foxtail millet (setaria italica l.). Bmc Genomics 22, 778. 10.1186/s12864-021-08095-y 34717536 PMC8557513

[B17] Fu X. Dong D. (2018). “Bioinformatic analysis of microrna sequencing data,” in Transcriptome data analysis (Springer), 109–125. 10.1007/978-1-4939-7710-9_8 29508293

[B18] Guindon S. Dufayard J.-F. Lefort V. Anisimova M. Hordijk W. Gascuel O. (2010). New algorithms and methods to estimate maximum-likelihood phylogenies: Assessing the performance of phyml 3.0. Syst. Biol. 59, 307–321. 10.1093/sysbio/syq010 20525638

[B19] Hao X. Yang T. Liu R. Hu J. Yao Y. Burlyaeva M. (2017). An rna sequencing transcriptome analysis of grasspea (lathyrus sativus l.) and development of ssr and kasp markers. Front. plant Sci. 8, 1873. 10.3389/fpls.2017.01873 29163598 PMC5671653

[B20] Islam M. Strawn M. Behura S. K. (2022). Fetal origin of sex-bias brain aging. bioRxiv. 10.1096/fj.202200255RR 35869938

[B21] Jin H. Martin C. (1999). Multifunctionality and diversity within the plant myb-gene family. Plant Mol. Biol. 41, 577–585. 10.1023/a:1006319732410 10645718

[B22] Journot-Catalino N. Somssich I. E. Roby D. Kroj T. (2006). The transcription factors wrky11 and wrky17 act as negative regulators of basal resistance in arabidopsis thaliana. Plant Cell 18, 3289–3302. 10.1105/tpc.106.044149 17114354 PMC1693958

[B23] Kapli P. Yang Z. Telford M. J. (2020). Phylogenetic tree building in the genomic age. Nat. Rev. Genet. 21, 428–444. 10.1038/s41576-020-0233-0 32424311

[B24] Kohl M. Wiese S. Warscheid B. (2011). “Cytoscape: Software for visualization and analysis of biological networks,” in Data mining in proteomics (Springer), 291–303. 10.1007/978-1-60761-987-1_18 21063955

[B25] Lambein F. Travella S. Kuo Y.-H. Van Montagu M. Heijde M. (2019). Grass pea (lathyrus sativus l.): Orphan crop, nutraceutical or just plain food? Planta 250, 821–838. 10.1007/s00425-018-03084-0 30719530

[B26] Lescot M. Déhais P. Thijs G. Marchal K. Moreau Y. Van de Peer Y. (2002). Plantcare, a database of plant cis-acting regulatory elements and a portal to tools for *in silico* analysis of promoter sequences. Nucleic acids Res. 30, 325–327. 10.1093/nar/30.1.325 11752327 PMC99092

[B27] Letunic I. Bork P. (2007). Interactive tree of life (itol): An online tool for phylogenetic tree display and annotation. Bioinformatics 23, 127–128. 10.1093/bioinformatics/btl529 17050570

[B28] Li J. Wang T. Han J. Ren Z. (2020). Genome-wide identification and characterization of cucumber bhlh family genes and the functional characterization of csbhlh041 in nacl and aba tolerance in arabidopsis and cucumber. BMC plant Biol. 20, 272. 10.1186/s12870-020-02440-1 32527214 PMC7291561

[B29] Li X. Duan X. Jiang H. Sun Y. Tang Y. Yuan Z. (2006). Genome-wide analysis of basic/helix-loop-helix transcription factor family in rice and arabidopsis. Plant physiol. 141, 1167–1184. 10.1104/pp.106.080580 16896230 PMC1533929

[B30] Li X. Nair A. Wang S. Wang L. (2015). “Quality control of rna-seq experiments,” in RNA bioinformatics (Springer), 137–146. 10.1007/978-1-4939-2291-8_8 25577376

[B31] Liu Y. Ma K. Qi Y. Lv G. Ren X. Liu Z. (2021). Transcriptional regulation of anthocyanin synthesis by myb-bhlh-wdr complexes in kiwifruit (actinidia chinensis). J. Agric. Food Chem. 69, 3677–3691. 10.1021/acs.jafc.0c07037 33749265

[B32] Mao K. Dong Q. Li C. Liu C. Ma F. (2017). Genome wide identification and characterization of apple bhlh transcription factors and expression analysis in response to drought and salt stress. Front. plant Sci. 8, 480. 10.3389/fpls.2017.00480 28443104 PMC5387082

[B33] Massari M. E. Murre C. (2000). Helix-loop-helix proteins: Regulators of transcription in eucaryotic organisms. Mol. Cell. Biol. 20, 429–440. 10.1128/mcb.20.2.429-440.2000 10611221 PMC85097

[B34] Ni L. Wang Z. Fu Z. Liu D. Yin Y. Li H. (2021). Genome-wide analysis of basic helix-loop-helix family genes and expression analysis in response to drought and salt stresses in hibiscus hamabo sieb. et zucc. Int. J. Mol. Sci. 22, 8748. 10.3390/ijms22168748 34445454 PMC8395896

[B35] Ohno S. Hosokawa M. Hoshino A. Kitamura Y. Morita Y. Park K.-I. (2011). A bhlh transcription factor, dvivs, is involved in regulation of anthocyanin synthesis in dahlia (dahlia variabilis). J. Exp. Bot. 62, 5105–5116. 10.1093/jxb/err216 21765172 PMC3193017

[B36] Pertea M. Kim D. Pertea G. M. Leek J. T. Salzberg S. L. (2016). Transcript-level expression analysis of rna-seq experiments with hisat, stringtie and ballgown. Nat. Protoc. 11, 1650–1667. 10.1038/nprot.2016.095 27560171 PMC5032908

[B37] Pires N. Dolan L. (2010). Origin and diversification of basic-helix-loop-helix proteins in plants. Mol. Biol. Evol. 27, 862–874. 10.1093/molbev/msp288 19942615 PMC2839125

[B38] Piwowarczyk B. Tokarz K. Kamińska I. (2016). Responses of grass pea seedlings to salinity stress in *in vitro* culture conditions. Plant Cell, Tissue Organ Cult. (PCTOC) 124, 227–240. 10.1007/s11240-015-0887-z

[B39] Qian Y. Chen C. Jiang L. Zhang J. Ren Q. (2019). Genome-wide identification, classification and expression analysis of the jmjc domain-containing histone demethylase gene family in maize. BMC genomics 20, 256. 10.1186/s12864-019-5633-1 30935385 PMC6444447

[B40] Rehman S. Mahmood T. (2015). Functional role of dreb and erf transcription factors: Regulating stress-responsive network in plants. Acta Physiol. Plant. 37, 178. 10.1007/s11738-015-1929-1

[B41] Salih H. Tan L. Htet N. N. W. (2021). Genome-wide identification, characterization of bhlh transcription factors in mango. Trop. Plant Biol. 14, 72–81. 10.1007/s12042-020-09277-w

[B42] Sanagi M. Aoyama S. Kubo A. Lu Y. Sato Y. Ito S. (2021). Low nitrogen conditions accelerate flowering by modulating the phosphorylation state of flowering bhlh 4 in arabidopsis. Proc. Natl. Acad. Sci. 118, e2022942118. 10.1073/pnas.2022942118 33963081 PMC8126780

[B43] Schmittgen T. D. Livak K. J. (2008). Analyzing real-time pcr data by the comparative ct method. Nat. Protoc. 3, 1101–1108. 10.1038/nprot.2008.73 18546601

[B44] Schubert V. (2009). Smc proteins and their multiple functions in higher plants. Cytogenet. genome Res. 124, 202–214. 10.1159/000218126 19556774

[B45] Seo H. Sepuru K. M. Putarjunan A. Aguirre L. Burrows B. A. Torii K. U. (2022). Intragenic suppressors unravel the role of the scream act-like domain for bhlh partner selectivity in stomatal development. Proc. Natl. Acad. Sci. 119, e2117774119. 10.1073/pnas.2117774119 35173013 PMC8892516

[B46] Seppey M. Manni M. Zdobnov E. M. (2019). “Busco: Assessing genome assembly and annotation completeness,” in Gene prediction (Springer), 227–245. 10.1007/978-1-4939-9173-0_14 31020564

[B47] Shen T. Wen X. Wen Z. Qiu Z. Hou Q. Li Z. (2021). Genome-wide identification and expression analysis of bhlh transcription factor family in response to cold stress in sweet cherry (prunus avium l.). Sci. Hortic. 279, 109905. 10.1016/j.scienta.2021.109905

[B48] Shen W. Cui X. Li H. Teng R.-M. Wang Y.-X. Liu H. (2019). Genome-wide identification and analyses of bhlh family genes in brassica napus. Can. J. plant Sci. 99, 589–598. 10.1139/cjps-2018-0230

[B49] Srinivasan K. A. Virdee S. K. McArthur A. G. (2020). Strandedness during cdna synthesis, the stranded parameter in htseq-count and analysis of rna-seq data. Briefings Funct. Genomics 19, 339–342. 10.1093/bfgp/elaa010 32415774

[B50] Sun H. Fan H.-J. Ling H.-Q. (2015). Genome-wide identification and characterization of the bhlh gene family in tomato. BMC genomics 16, 9–12. 10.1186/s12864-014-1209-2 25612924 PMC4312455

[B51] Szklarczyk D. Franceschini A. Wyder S. Forslund K. Heller D. Huerta-Cepas J. (2015). String v10: Protein–protein interaction networks, integrated over the tree of life. Nucleic acids Res. 43, D447–D452. 10.1093/nar/gku1003 25352553 PMC4383874

[B52] Takatsuji H. (1998). Zinc-finger transcription factors in plants. Cell. Mol. Life Sci. CMLS 54, 582–596. 10.1007/s000180050186 9676577 PMC11147231

[B53] Wang R. Zhao P. Kong N. Lu R. Pei Y. Huang C. (2018). Genome-wide identification and characterization of the potato bhlh transcription factor family. Genes 9, 54. 10.3390/genes9010054 29361801 PMC5793205

[B54] Wittkopp P. J. Kalay G. (2012). Cis-regulatory elements: Molecular mechanisms and evolutionary processes underlying divergence. Nat. Rev. Genet. 13, 59–69. 10.1038/nrg3095 22143240

[B55] Xie X.-B. Li S. Zhang R.-F. Zhao J. Chen Y.-C. Zhao Q. (2012). The bhlh transcription factor mdbhlh3 promotes anthocyanin accumulation and fruit colouration in response to low temperature in apples. Plant, Cell and Environ. 35, 1884–1897. 10.1111/j.1365-3040.2012.02523.x 22519753

[B56] Xu G. Guo C. Shan H. Kong H. (2012). Divergence of duplicate genes in exon–intron structure. Proc. Natl. Acad. Sci. 109, 1187–1192. 10.1073/pnas.1109047109 22232673 PMC3268293

[B57] Xu J. Xu H. Zhao H. Liu H. Xu L. Liang Z. (2022). Genome-wide investigation of bhlh genes and expression analysis under salt and hormonal treatments in andrographis paniculata. Industrial Crops Prod. 183, 114928. 10.1016/j.indcrop.2022.114928

[B58] Xu Q. Liu F. Qu R. Gillman J. D. Bi C. Hu X. (2018). Transcriptomic profiling of lathyrus sativus l. metabolism of *β*-odap, a neuroexcitatory amino acid associated with neurodegenerative lower limb paralysis. Plant Mol. Biol. Report. 36, 832–843. 10.1007/s11105-018-1123-x

[B59] Yang J. Gao M. Huang L. Wang Y. van Nocker S. Wan R. (2017). Identification and expression analysis of the apple (malus× domestica) basic helix-loop-helix transcription factor family. Sci. Rep. 7, 28–14. 10.1038/s41598-017-00040-y 28174429 PMC5428380

[B60] Yin J. Chang X. Kasuga T. Bui M. Reid M. S. Jiang C.-Z. (2015). A basic helix-loop-helix transcription factor, phfbh4, regulates flower senescence by modulating ethylene biosynthesis pathway in petunia. Hortic. Res. 2, 15059. 10.1038/hortres.2015.59 26715989 PMC4680862

[B61] Zhang Y. Liu F. Guo H. Ma H. Chen H. Song Y. (2022). Selection of reference genes for quantitative real-time pcr analysis in lathyrus sativus l. under different development stages and drought stress. Genet. Resour. Crop Evol. 69, 2319–2330. 10.1007/s10722-022-01374-x

[B62] Zhang Y. Wang L. (2005). The wrky transcription factor superfamily: Its origin in eukaryotes and expansion in plants. BMC Evol. Biol. 5, 1–12. 10.1186/1471-2148-5-1 15629062 PMC544883

[B63] Zhang Z. Chen J. Liang C. Liu F. Hou X. Zou X. (2020). Genome-wide identification and characterization of the bhlh transcription factor family in pepper (capsicum annuum l.). Front. Genet. 11, 570156. 10.3389/fgene.2020.570156 33101390 PMC7545091

[B64] Zhao Y. Zhou L.-M. Chen Y.-Y. Yang S.-G. Tian W.-M. (2011). Myc genes with differential responses to tapping, mechanical wounding, ethrel and methyl jasmonate in laticifers of rubber tree (hevea brasiliensis muell. arg.). J. plant physiology 168, 1649–1658. 10.1016/j.jplph.2011.02.010 21489651

[B65] Zhou X. Liao Y. Kim S.-U. Chen Z. Nie G. Cheng S. (2020). Genome-wide identification and characterization of bhlh family genes from ginkgo biloba. Sci. Rep. 10, 13723. 10.1038/s41598-020-69305-3 32792673 PMC7426926

