# Supplementary material for: Genome-wide identification, characterization, and validation of the bHLH transcription factors in grass pea
Source: Front Genet. 2023 Mar 20;14:1128992. doi: 10.3389/fgene.2023.1128992 (PMC10067732; doi:10.3389/fgene.2023.1128992)
Supplement: Supplementary file 1 [file Table3.DOC]

>LsbHLHD2

MDARIKKPKTESGYGENLNFGSEYDKLENKAATTITNRNPIQARDHVIAERKRRQKLNQK

FITLSSILPGLKKMDKATILEDAIIHLNQLKERVESLEEQVADTKVESAVFVKRSILFAN

DDSSSSFNENSDQSLPKIEARVSGKHMLIRIHCEKHHGRSATAILNKLEKHHLTIQSSSV

LPFGKKYIDITIVAE

>LsbHLHD3

MNKKSQKKHNIVSSCYIDSEHSDSEYYPQLPTPTTTNDSFEKREPKKRGRKPLTGIQTPM

NHVEAERQRREKLNNRFYALRAVVPNVSRMDKASLLSDAVDYINELKAKIEELESENQKE

SKKQKMETIESTVTTTSTVVDQKTTCSSNNNNNNVSALDIDVKIIGNDAMVRVQSENVNH

PGARLMSVFKDLEFQVHHASISCFNEIMVQDVVVVQLPDEMRNEESLRSAIRMRLEHE

>LsbHLHD4

MNLWSDDNSSVMEAFMTSSDLSTLWPPAPPSQPPQTTTGFNQDTLQQRLQALIEGASELW

TYAIFWQPSYDYSGTSLLGWGDGYYKGEEDKTKAKSKSKSKASSPAEQEHRRKVLRELNS

LISGNPVTDESAVDEEVTDTEWFFLVSMTQSFVNGGGLPGQAYFNSTPVWLVGGESLSVS

VCERARQGQEYGLQTLACIPSPNGVLELGSTELIYQNNDLMNKVKMLFNFNNSFEFGSSW

QLGNSATIAHQGENDPSSIWLNDPETRDSVDNNSLAAPTTTNTSISNPSHQQHQHQQQPQ

NNNNNQSLSVSVTKTIQFETHGSSTLTEVPSVVHVSSKQNNQSFFNKEMNVSEYGGSNHH

QRSLKPESGEILSFGGESKKTSYVTNNGNSNSNFFSGQSNLLSVAEENHNNNTNSGNGKR

RSPNSRGSNNDDGMLSFTSGIIVPPAASNLKFSSGTGCGDSDHSDLEASVVKEVDSSRVV

EPEKKPRKRGRKPANGREEPLNHVEAERQRREKLNQRFYALRAVVPNVSKMDKASLLGDA

ISYITELKTKLQKTESDKDGLEKQFDEVKSELQKINENSSHPPPTPPQQQQPVPKKSSPS

NQALIDLDIDVKIIGWDAMIRIQCSKKNHPAARLMAALMELDLEVHHASVSVVNDLMIQQ

ATVKMGSRFYTQEQLRAALSSQVGDVQ

>LsbHLHD5

MNSIWTDENSSVMEAFMSSSDLSSLWLPTPNSAASTTTPGPDTTKLPSQQQPLFNQETLQ

HRLQALIEDAKESWTYAIFWQTSYDYSTSRQLLGWGDGYYKGEDDKEKAKKVILPEQQAH

RNKVLRELNSLISGSSGSDDVVDEDVTDTEWFFLTSMTHSFVNGSGLLSQAYFNSSPVWI

NDRLSMSTCERTRAAHVHGLQTLVYIPAPSSNGVVELASTEIIPHSAGIMEKVRFLFDFN

NPEARSWPLNSGDNDPSSMWLDIPGSGGIEIRDSVNTVSAVSVTTSANTTITKKLPFEIH

GASSSVPETSTAVNISTGHRENQNQNQNQTFFPKEFNFSGSFKPESGEILNFGESKKSSY

SSANGNFFPGPSPFAANEENRKRRSPVSRSSIDDGILSFTSGKLLQASGIKSGGGDSDHS

DVEVSVVKEAVSCRIMEPEKRPRKRGRKPANGREEPLNHVEAERQRREKLNQRFYALRAV

VPNVSKMDKASLLGDAISYINELKSKLQGLESSKGELEKELDTTRKELEIATKKPVLLNE

EEKEKTNNNSKLIDLDIDVKIMGWDAMIRIQCSKKNHPAAKLMAALKELDLDVNHASVSV

VNDLMIQQASINMGSRFYTQEQLLSVLSSKIGDTQ

>LsbHLHD6

MRKRVLQKLHTAFGGSDEDNYAFGLDRVTDTEMFFLASMYFSFPKGYGGPGKCFDSGQNL

WLKSVSDYCVRSFLAKSAGIQTVVLLPTDFGVLELGSVRILPQSFELLNNVKSLFSLSNS

ITQSSSSLYPSPSVINEGRDDESGISNGVHVPPPKVALNLNNGRSHFREKLAIRKMDSSI

NFPSSRNGVSASTSWGKNQGQGEVFGSIHGVRKGFSNYQPQRQQVQMQMQIDFSGTKSRV

NSVRPVIGESGLVAADVDADQANDERRPRKRGRKPASGRDEPLNHVEAERQRREKLNQRF

YALRAVVPNISKMDKASLLGDAIAYINELQAKLKVMESDKETFGSSTSRDGSSNTRSENP

RQVPPPHDVDIQASQDEVIVKVSCPIDTHPISKVIETFKDAQIGVAESKLTAANDTIYHT

FVIKSEESEQLTKDKLIAAFSGESIPLQTQTTLSI

>LsbHLHD7

MSSCSGDKQNMHEQTGCFDPNTMDEGIENSLKDNPFSQTHPNQMVEGNNHNHSEEDFSYH

NPQHLDNNLVQEVYQNSTWDTNVHELQGMDYANHQEQHQLHEQQFQQIVETQNHNQTYNP

SNILDPHYPSPDLLNLLHLPRCSDSSFLANPPSNICMTNLNQKPPNFHNSMSFLGDLPIG

SSDNTSGSSVLYDPLFPLNLPPQPPALRELFQSLPRGYSMPTSSRNGSLFGGGDEMEGDG

DMGVLEFNRVAANVGKGRGGKATKHFATEKQRREQLNGKYKILRGLIPNPTKVDRASVVG

DAIEYIRELIRTVNELKLLVEKKRHGREMCKTHKAEDAAAESCNIKPFGDPDGSIRTSWL

QRKSKDSEVDVRIVDDDVTIKLFQRKKVNCLLFVSKVLDELQLELNHVAGGHVGEYCSFL

FNSKVSEGSSVYASAIANKVIDVLDTQYAAGVPYT

>LsbHLHD8

KMEFYDGDFMEELMALRTETPTQEENHFFSSSTNHNNLSFDNCFDQNSSCSQQVFPQSYV

NNDYYSNTFNEIYGSLLDETSTPQILDSYYNNNTLDAPFASQEDFPLSMIGEEEDQTMLR

EEIKNLELQTTCKMEETQSPEMPVVFNSANSLERKNRSKKLQGQPSKNLMAERRRRKRLN

DRLSMLRAIVPKISKMDRTAILGDTIDYMKELLEKINNLQQEIELDSNMSSIIKDVKSNE

ILIRNSPKFEVERGLDTKVEICCAGKPGLLLSTVNTLEALGLEIQQCVISCFNEFTMQAS

CSEEFEKGQC

>LsbHLHD9

MEMEDFDWGYDILGDHTNGFQVNSGVNGNGYGSSFSLVLDRERGMLVETPAKMEQKGVST

ERTAEALKNHSDAERRRRARINSHLDTLRSVIPGALKMDKASLLGEVIRHLKEVKRTATQ

ACEGLVIPKDNDEISVEEQECGFNGFRYSIKASLCCEYQPGLLSSIRQALEALRLVIVKA

DIATFGGRMKNVFVVISCEEQNFDDAEYRQFLAGSVYQALKSV

>LsbHLHD10

MLDEMEMEGCDLFQQNLFNDDDLVSDDIASVLQQQDKPLSCESSVSYSPVSSHSSTTNSQ

VMSFENTTTTHFHAFDCTLNTKHNNNNKVHVIAERKRREKLNKSLIALAALIPGLKKMDK

ASVLGDAIKYMKELQERLKVLEEQDRNSRVESVVTENKPWVIHESWSDDGSESLSHVDAR

VLDKDVLIRFLCQKQKGVLIKLLKEIQKLDLCVVNSTVLPFGDSINITIVAKMETGYSMT

NDLVKNLRVAAFKSL

>LsbHLHD11

FYEHFESMCLILQEIMEDPSLCLHQWYLNSIDESNSLPIASAFGDTLQHPSYIYPNFNLR

ASVETDQRPETQFVSYQNLLSYVDSNHFNQLGLVKPKDEMVCPQNSNTTSDAVSQGIMEA

QRISTRNKVSLPQDHIIAERKRREKLSQRFIALSALVPGLQKMDKVTVLGDAIKYLKKLQ

EKVKALEEERNMKKNVEYVVVVKKSQLTNDVENSSAESGSPFDEELPEIEARFCDRNVLI

RIHCEKSKGAAEKSIHEIEKLHLKVINSCVMAFGSCALDITVIAQMDIEFCMTLKDLVTN

LRSAFASFI

>LsbHLHD12

MDEDILKTLESDESSMDMMTMMMQMEKFPDFHQPFYSYNHNINLNNSHNEFPYGNSNVTY

PQPPFSHPQQPMTPSLQHNGVQIPSGRINNASFSSYSDKKNSMAAMREMIFRMAVMQPVN

IDPETIKPPKRKNVKISKDPQSVAARHRRERISERIRILQRLVPGGTKMDTASMLDEAIH

YVKFLKKQVQTLEQVGANRPMNVAGFPGMISNGNVNYSSFLRGCSSPCQMVGPTSKQMLS

>LsbHLHD13

MKGTGNYAAMNGSNGNVSPSINRLTCQVSFPSRNASSLGVLSQISEIDSEDIEATSPGDG

GSNGDTAHYGSGFPYSSWNDTQSFSENLSGLKRGRIGSEKMFSDFQSGGLENQVHTLSHH

LSLPKTSSEMIAMEKLFQFPDSVPCKIRAKRGCATHPRSIAERMRRTRISERMRKLQELV

PNMDKQTNTSDMLDLAVDYIKELQKQFK

>LsbHLHD14

MPPCFNTLDYSLDQQYQQFTKYRVGETSGENNNGMEDNYLPQTQNSGGFYGTTNNSFDKM

SFADVMQFADFGPKLALNRQESEIDDDPVYFLKFPVLNNKMEDQNLMLHQDGVGENEDRF

KVADNLRDHEETRVSDENNSVQLVQETNCALVQENSKKRKRPRTVKTSEEVESQRMTHIA

VERNRRKQMNEHLRVLRSLMPGSYVQRGDQASIIGGAIEFVRELEQLLQCLESQKRRRLV

GEAQSKQVGDSTQQQAPFFQQAPLPNEQMKIVEMESGLEEETAE

>LsbHLHD15

MNGQDQQDSLVYTDNFELRDFVDDPNFDQFIDLIRGENENAICNFGSDLINDCFIDNHQP

LSIPLMPLLDHNNNNINNIVNVYDPSSSTIGSFACYDEEVNGEGDNIGDDSPATTTTTSI

DDTKSRAKTDRSKTLVSERRRRGRMKDKLYALRSLVPNITK

>LsbHLHD16

MNEIFPNDENFNNYDVPCAKRQKLGYEETNQQQQQQQQQHFANYFVDEFIANPLTTSFEA

EPFSGSKMVNVVQCEKKVSERSISTQSVAARERRRKITEKTQELGKLVPGGPKMNTAEML

NAAANYVRFLQAQVGMLQVMETFSKEEKEPPPSEDLHKLVVSPFVQEKLYSEEKCFIPKE

IVTTLANNVDVRSKPSILQGLKQLVGREIDHENDKKLKQE

>LsbHLHD17

MSSIPKLTGLNAAALYIISFICAIASRSGSSVEDEQHIDDDDEFGCWKFMLVEQGLLSDL

GFLLFGGLSVIRLKPPSRDEEL

>LsbHLHD18

MDEDILKTLETDESSMDMMTMMMQMEKFPEFHPPFYNNNNILHINLNNTYNELPYGNSNV

TYPQPPLSYPQQPMTPSIQHNGVQIPSGRINNALFSSYSDKKNSMAIMREMIFRMAVMQP

VNIDPEAIKPPKRKNVKISKDPQSVAARHRRERISERIRILQRLVPGGTKMDTASMLDEA

IHYVKFLKKQVQTLEQVGANRPMNVVGFPGMVSNGNSNYNSSFLRGCSSPCQMHIVVGYG

IEVNAGK

>LsbHLHD19

MDKASVLGDAINHVKQLQEQVKILEQKNQRKNIESVVYVEKTTKSCSSDEDVSVSDTSSN

SGNGGNNYCHPNRSLPEVEARVSEKNVLIRIHCEKEKGVLMKIIHVIENLHLSVISNSTL

LFGTTKLDITIIAEMDDEFRLSVQELVRSIRVGLLQFMKY

>LsbHLHD20

METNEQNRIILQQDHHQIPMSNSSGIWPTNSYNNMMMPAAAPPPPPSSSGSLSDILGIHQ

IEEVEEQEEELGAMKEMMYKIAAMQPVEIDPATIRKPKRRNVRISDDPQSVAARHRRERI

SEKIRILQRLVPGGTKMDTASMLDEAIRYVKFLKKQIRLLQSTPQNQQYQLPLSSQCINS

TPPPPNALLLSPSCDIVWPFAPNLLLASTAAASIDLPAGIQFNAGEHSHPHPHPHSHSHS

HVHACDGSSSFNHHE

>LsbHLHD21

MEQVNDSNILYVSQDELFFNAEEDINKGKPKKKYYNEDGTRVFVSKNLEIERRRRDKLHS

RLCTLRSIVPNITNMLKESIIEDAITYVKKLQDDVDKLTQELQAMEAEENLERKINKVSD

AEEMKKWGIQEEIHVEKTDGTNLWIKMIIEKKRGRFKKLMENLGGEPFDVDQAKYWLQDI

IKII

>LsbHLHD22

MDHNENITAETMSLKLSPSSSISSFQSQILSFENLPNSPATNTPHFYEFDPTSTTLNSKP

NETRSVSLSSLPQLGNTHVSTQTPKGSSKNQNLETKTSQPKRSRANAEDHIMAERKRREV

LSQSFIALAALVPNLKKMDKASVLAESIKYVKELKERLEILEKHNKKTKADSVVVLNKPD

LCSDEDSSSCDENIEGADGGESETLVQVEARALEKEMLIRIHCKKKKGVLVKMMGEIQSL

ELFVMNTSVLPFGDSILDITIIAQ

>LsbHLHD23

MRPVSNTLPFHLQQLPQITPTQLFPIDHNDAIMRAIQNVLSTPPSQQSYAAHPGASAFGR

YRNDKSPIIVGSNFRRQSLMKRSFAFFRSLNLMRLRERNQAMRPSSNQLHHMISERRRRE

KLNDNFQALRALLPQGTKKDKASILITAKETLRSLMEEIEKLSKRNQELMSEKLTASNKE

TMKFSSNERINVRVLHVSESSSSDDEPMVVDLQVNVIGQVSQVDMLIRLLEFLKQVHHVN

LISMDATNSNQIFHNSSNNIQAKDYS

>LsbHLHD24

MVDEYILYDKDRNLNSNSNLLDLDEEEEFLSHDIASVFAFEEHRESLQQCLNTECISTTL

SETFTDETSFESFDNFDFDFEKPTKQMKTIDHSATSLSSYHLPPILSFDNPNPTEFYRYD

LKQTETVTKSLGNTNFLTQNSKGSSKTSRAKRSPADIKDHIMAERKRREKLSQSFIALAA

LIPDLKKMDKASILAETIKYVKELKERLEILETKGKKPKADQSTVTPSPIKPEVYNNKHC

SSSDESTETETAVE

>LsbHLHD26

MDSLPSPFDNFDFNYDNNYETTPNCLPLETQPDNHYVAQTRPTKKIKTSINPSSYSSPQL

ISFEHFNATPVASNELYNLDYSDVKPKLEKGCNENKDFAANYDIRANQTRNTAQAKEHVM

AERKRREKLTRSFIALSAILPGLKK

>LsbHLHD27

MMLMMMMQLPEFSSSNNNNNANEFPPSDQDFYSSSVNPNNNTRPLSDLIDNPPNPFPWSS

SSSLPLQSTNNISFTTNNPATPLFLQQQQQAPPLMFSNSNNNQAVSNPNHYGASEKRTSM

AAMREMIFRIAAMQPIYIDPESVKPPKRRNVKISKDPQSIAARHRRERISEKIRILQRMV

PGGTKMDTASMLDEAIHYVKFLKTQLKTLQEHANNNRTVAGSGIGFPVSFPKPHQARNVD

HYGD

>LsbHLHD28

MDFLPSPFDNNYETTPNSLPLETQPDDHYVAQNRPTKKIKTLIDTKQTKDHLIASNPKPS

SYSSPQLISFEHFNATPVSSQQFYSYDYSDVKPKLEKGCNENKDFSANYDYGTSKTRNTT

QAKEHVMAERKRREKLTRSFIALSAILPGLKK

>LsbHLHD29

LHHPLEDLTQEDTHYSQTVTTVLQNQWIDSPSINYINYSTQSSFTTWTNHHFHPPPPPDT

ATSQWLLKYILFTVPYLHTKNHDETSPQTHDTAGVNSNDPSARLRGKGTPQDELSANHVL

AERRRREKLNERFIILRSLVPFVTKMDKASILGDTIEYLKQLRRKIQDLETRNRQMESEK

SGVPVLVGPTEKKKVRIVEGNGGGGGVRAKAVEVVEKEIVASVQVSIIESDALLEIECLQ

REGLLLDVMMMLRELRIEVIGVQSSLNNGVFVAELRAKVKENGNGKKVSIVEVKRALNQI

IPHNNI

>LsbHLHD30

MGWLLQELDPPQSLNISHKEKNYATNSEYSLPQYHQFSSVKQQHVEIETPPPSPKLMVKK

LNHNASERDRRKKVNTLISSLRSLLPGDDQTKKMSIPVTISRVLKYIPELQKQVEGLTKK

KEDLLSRISQQQYAVNKESQRKIIPNYNSSFVVSTSRLNDSELVVHISSYDAYKIPLSEI

LMCLENNGLVLLNSSCSKTFGGRLFYNLHFQVDETQRLECDDLIQKLLLVYERQRSNQVV

LGAKDHMIRSVMIY

>LsbHLHD31

NDSRVEVIGVPKIFGQDLNSATHFREKLAVRKMDERPSWGVRPNGNGISFPNGVHGSGWR

GGSQVVRQHVPADVFTPRPSASNAPELANGGGRHDFVLNNYQQQRKAQMQIDFSGATSRP

SVRSIVGESEISDVEASCRDDAPSPSDDRRPRKRGRKPAHGRVEPLNHVEAERQRREKLN

QRFYALRAVVPNISKMDKASLLGDAIAYINELQAKLKSMESEREQQLATTSRDGSSSALQ

TNSRPENHQNKTPEINIQATQEGVIVKVSFPIDVHPISKLIQAFKDTEITILESKLNATN

DTVFHTFIIKSQTSEQLTKEKLIAAFSKESNSL

>LsbHLHD32

EFQSWPLPVEGSLEDRAARASKSHSQAEKRRRDRTNTQLANLRKLIPKYDKSLNSGATVL

VRDYSEAIREKKVY

>LsbHLHR1

MSQCVPSWEVDENPQSHARSASLRSNSNSTAPHDVPMLDYDVAELTWENGQLSMHGLGLP

RVPVKPLTTAPSKETWEKPRGSGTLESIVNQATSFPHHGKAPFLSVGGGVYGNMLVPWLD

PQRAAAIAAATATSNGIVVDALVPCSNLKKEQRLHARDPISIRGIGSCMVGGPTPVGSCS

AVGATPQEEGGIFTGAKRGRVTHVAGSGRDQSMSNSATLGRQSQQVTLDTYDREFGMTGF

TSTSIASMENTSSDKQCTRTTTVDDHDSVCHSRPTRDDAEEDDKKRENRKSSVSTKRSRA

AAIHNQSERKRRDKINQRMKTLQKLVPNSSKTDKASMLDEVIEYLKQLQAQVNMVNRFNM

SSMMMPMTMQQQLQMSMMNQMGMGMGPMGMAGMGMGMGMGMGMDMNPMNRANIPGMPQVL

HPSAFMPMPAWDAGAAATATDRLQGPPATGMADPMSTFFGCQSQPMTMEAYSRIAAMYQQ

MQQQPPAPGSKT

>LsbHLHR2

MVSPENTNWLFDYALIDDIPVSDGSFAFTWPPSQPLNVCVDMDGSLGDSDGIKDPGSKKR

GRSDSSAPSSSKACREKLRRDRLNDKFVELGSILEPGRPPKTDKAAILTDAVRMVSQLRG

EAQKLKDSNSSLQEKIKELKVEKNELRDEKQRLKAEKEKLEQQVKSMNAQPSFLTHPPAI

PAAFAPQGQAHSNKLMPFMSYPGVAMWQFMPPASVDTSQDHVLRPPVA

>LsbHLHR3

ILPFRVFNSFFQLTGDSSESNCLMANNPSDAPAADDFLEQILSLQTFASPDSGLTGPDIS

LTGTSPMMLQLNSSDANHHLAAGGSFHAPVYQLGLSLDQGKGGFLKPEEASGSGERFRND

VVHGRPNNVFHGQPMHTTVPAAPHPPAMRPRVRARRGQATDPHSIAERLRRERIAERIRA

LQDLVPSVNKTDRAAMLDEIVDYVKFLRLQVKVLSMSRLGGAGAVAPLVTDIPLSSVEED

GSEGGRNRPAWDKWSNDGTEKQVAKLMEENVGAAMQLLQSKALCIMPISLASAIYQSQPP

DSSSIVKPETNPPSQT

>LsbHLHR4

MDQPGLLRPKKPFIADDEIVELLWQNGPIVTQSQNHRYNNKPPPRRNSDDSTRGGTSSPR

ENEYLFMQEGEMASWLHYPNTDGDSPLDQSFCADFLNQPSAVNNNSTMQTPPSRVRAEPE

PSSILRTSARESTLVDSCDTPAVMPAAISETVRSSMEPTEGATCASAPWTTFDEQGGSSS

SGEPVRKVEELDRKRKGKHTDEWKYHSEDVDFESAEEKNKTNGSSTKRSRAAEVHSLSER

RRRDRINEKMKALQELIPQSNKSDKASMLDEAIEYLRSLQLQVQRVQLMQMMSMGCGMVP

MMFPGIQQYIPPIGMGINRPVMPFPNMLPASTLPANFGPRFTMPPFHMPHVPTPDSFTMQ

AANLADSNMPTSVGTHDANQQPIPNFTDPYQQYLGPQQMQFQLMQNKAMNQPDVSKSEIR

QSGDK

>LsbHLHR5

MNNTLPDWSFGSDNFVTNQKKQSMGLDHELVELLWENGQVVLHSQTSKKPINSRNVHKNL

QSTTLSNLIQDDETVSWIQQYPFEDPIGQELCSNLLSELPPCHVDSYDSQPTTKPSFVEE

FSIPRFHHHVPDLSLKNNELCGSNKVVNFSHFSRLPNVSLACNNSDGDLRDKVTGNLSQC

DIRESSAMTVGLSHCGSNQVQQDPDVSKVSSDGVWTNTISAEPQQVKDNVQTTTIPWHEK

GKSEMLEPTFTSSSGGSGSSLGKTCSLSTRSHGEKRKGIDVDDSVEQSEDTELKSALSNK

VSQRSGSGRRNRAAEVHNLSERRRRDRINEKMKALQQLIPHSSKTDKASMLEEAIEYLKS

LQLQLQVMWMGSGMAPMMHPGFQHYMTQMGMSMPTASFPPLQNPLQLPRMPFDPSVSLCQ

TPNPTLTCQNPLFGAFNYQNQMQNPALSEQYARYMNYHLMQNASQPMNMYQYGPQTVQNS

QTMIPTSNNSGSMSGAANINDNVNGKIG

>LsbHLHR6

MESDLHQQQQPQANPSGLTRYRSAPSSYFNNIIDKEFYEHVFNRPSSPETERVFSRFMNS

LGSEEDLLTPKISVESTVKEEEEIVNVNINQQQQEQQQEQEHMIHHHQSNNYEHNPVSSH

GFYQTSVMPPLPNQNVVSGLDASFQMKSHGGNNSNLIRHSSSPAGLFSQINIENGYVSMR

GMGNLGAVNSSMKDAKFSTGRSLKNSANYSSGIMSTIAEVGDKCNEENNLESEVFGESHG

NDYIADYQVVDTWDDTEMMSENVGGLKRFRDSDSKQQFSAGFNAVVVQNETGGHSSSPLA

HQLSMPNTSSEIAAIEKFLHFSDSVPMKIRAKRGCATHPRSIAERVRRTKISERMRKLQD

LVPNMDKQTNTADMLDLAVDYIKDLQKQAQKLQDCQAKCTCSHKKPQ

>LsbHLHR7

MQTHEYFHRGKVKLPLVDEAIGTYMNAPHTSAFGAVLPTGARQITPFERFNLHPSEVCPE

NFIVFDQTDQQNRVLFNPATTYKFNSPSFNFNTHAYTQDFEKDKINQMERELSSSFEEDS

RDIDALMSLDSDELEDYDEEEVSTARTHHEKDESTSDTCSSYCTKSRKKRLLSSSVQNSS

GTKGYCSSSEKKQHREMKRMVKILRNIVPGSGNEMDTVTVLDEAVKYLKSLKVEVEQFGV

GQ

>LsbHLHR8

MNLWSDDNSSVMEAFMTSSDLSTLWPPAPPSQPPQTTTGFNQDTLQQRLQALIEGASELW

TYAIFWQPSYDYSGTSLLGWGDGYYKGEEDKTKAKSKSKSKASSPAEQEHRRKVLRELNS

LISGNPVTDESAVDEEVTDTEWFFLVSMTQSFVNGGGLPGQAYFNSTPVWLVGGESLSVS

VCERARQGQEYGLQTLACIPSPNGVLELGSTELIYQNNDLMNKVKMLFNFNNSFEFGSSW

QLGNSAAIAHQGENDPSSIWLNDPETRDSVDNNSLAAPTTTNTSISNPSHQQHQHQQQPQ

NNNNNQSLSVSVTKTIQFETHGSSTLTEVPSVVHVSSKQNNQSFFNKEMNVSEYGGSNHH

QRSLKPESGEILSFGGESKKTSYVTNNGNSNSNFFSGQSNLLSVAEENHNNNTNSGNGKR

RSPNSRGSNNDDGMLSFTSGIIVPPAASNLKFSSGTGCGDSDHSDLEASVVKEVDSSRVV

EPEKKPRKRGRKPANGREEPLNHVEAERQRREKLNQRFYALRAVVPNVSKMDKASLLGDA

ISYITELKTKLQKTESDKDGLEKQFDEVKSELQKINENSSHPPPTPPQQQQPVPKKSSPS

NQALIDLDIDVKIIGWDAMIRIQCSKKNHPAARLMAALMELDLEVHHASVSVVNDLMIQQ

ATVKMGSRFYTQEQLRAALSSQVGDVQ

>LsbHLHR9

MGDNLHQLLRSLCFNTHWNYAIFWKLKHCAPNPMILTLEDAYYDNSDYFDSSENKYCQKT

LDQIKGGKFSHEALGLAVAKMSYNVYSLGEGIVGQVAVTGKHRWICADDQATSSGLSFEF

DDLWQTQFSAGIRTIVVMSVVPLGVVQLGSLIKVNEDMGVINQLRNLFLSTQDYSIDHIP

SQIQSSLKSSSSQDILKEKSSSDIMPACMTNETVGLLMPLQCSGRNGATNSAYWEMGDDV

VKYEGPELNSDASPILLQSAFGMINVEHQEFGEIRPLSARECAGGSDGCKNMKLESEQNL

SSFLNNSVINNDGVGDLIHRSEKARVDSACFPTDFLDAYVSESDKSHKHCEKSEFWTVPC

GKDTSYTELSFPAGCELHEALGPASLKGSKYFDLLAQVNQNVKIVDMPDAVNTSQSTCQS

PPEHLLEAMVANICHSSNDDVNSESSFYRSKQSAISSGKKPEVSIQNVHTVNSECYSIDH

PSLFQEGKHHCLSSSSGICGVMSSKGISSICPSACSEQLERSSGPSKNSKKRARPGESCR

PRPRDRQLIQDRIKELRDLVPNGAKCSIDSLLERSIKHMLFLQSVTKHADKLTKFAVSKS

KLHHVEADIHGSSSSEQGSSWAMEVGGHLKVHSILVENLSKNGQMLVEMLCEEWSHFLEI

AEAIRSLGLTILKGATKTRDDKMLICFIVEVENNKNIHRLDILWPLVQILQSKSNVQQQ

>LsbHLHR10

MVSRENNNWLFDYGLIDDISPPISTFMPTPSTGFTWPAHQPFNVSSNVGAEIDVSLGDSD

SLKESGSKKRGRSESCAATSSKACREKLRRDRLNDKFIELGSILEPGRPAKTDKAAILID

AVRMVTQLKGEAEKLKDANTGLQEKIKELKVEKN

>LsbHLHR11

MERLQGPINSSFFGEVNCLDQTLLDTESLRFEEDEQFLLSSLEDNMPFLQMLQSVEPPIY

PLKEPNFQTLLRLQHMKKPWEEDITFIPRMDSLQQVQTTLEFESCVTHDVLEMQSPVKSE

SYELQHKVSASCIEKLSYECNQEETKTCSKSQLVTTREKRKRKRTRPVKNKEDVENQRMT

HIAVERNRRRQMNDHLSVLRSLMPSSYIQRGDQASIIGGAIDFVKELEQLLESLEVQKRI

KKNEEFGSSSSSSSPPQASYGMKLSCCEENEVKAENKSEAADIKVTLIQTHVNLKIECKR

RCGQLIKVIVALENLRLTILHLNITSFESSVLYSLNLKVEEDCKLGTANDIAEAVNEIFN

YINGN

>LsbHLHR12

MDPAPIINAAPNTLSFHPPEIWQFPPSTTPQFEHGFGAFATEVPGRELVGSELRLPNRGR

KRRDTEEDSVKGVSTSNDVNGDGGDDGKRIKTSWKSKDEEVVGDENSSGKHAEKITEEPH

PKQDFIHVRARRGQATDSHSLAERARREKISERMKTLQDLVPGCNKVIGKALVLDEIINY

IQSLHHQVEFLSMKLEAVNSRLTPGIEVFPPKVFDQQTYETTPIPFVSQATREYSRGSSP

EWLHMQVGGGFERST

>LsbHLHR13

MLHCLNTSGNLGGEETLTTISDMTVLQRQKARINFQQFSSSPQFTMVACDSALGEVVANS

IKPDPGFGKDTVKKRKAEINNNNSKVDVVVVPECDKRIKISAEEEESKTSDQTSKGHKNK

KSNNGTKNNRENSGDTNSKEKLDYIHVRARRGQATDSHSLAERVRREKISERMKYLQDLV

PGCNKIAGKAGMLDEIINYVQSLQRQVEFLSMKLAAVNPRLDFNIDELFAKEVFPQNFPT

IGMQSDMTNPTYLQFNSAQQVSSCGGLINNMGIIPPEIGLRRNINIPAASSLPEIFDSSC

FTHILPSSSWEGDFQNLYNMNFDQARATSFPSQPQFYTGLVEASNLKIEMQ

>LsbHLHR14

MKIEVGLGGNGLWNEDEKAMVVEVLGVKAFDYLVTKSVSNENLLMAIGSGENLQNKLSDL

VERSNVLNFSWNYAIFWQISQSKYGDWVLGWGDGCCREPKEEEEKDLGMKSVVSLNSIED

EKQQRLRKRVLQKLHTTFGGSDEDNYAFGLDRVTDTEMFFLASMYFSFANGDGGPGKCFA

SGKHLWLCDALKSGSSDYCVRSFLAKSVGFQTIVLVPTDLGVVELGSVRMVGESFELLQT

VKSVFSTQSSLARVKSISSLDGNRGENVNENENDNEIAPFSCLKVGESIKNNNSNGNLHL

RRNDNDSRVEVIGVPKIFGQDLNSATHFREKLAVRKMDERPSWGVRPNGNGISFPNGVHG

SGWRGGSQVVRQHVPADVFAPRPSASNAPELANGGGRHDFVLNNYQQQRKAQMQIDFSGA

TSRPSVRSIVGESEISDVEASCRDDAPSPSDDRRPRKRGRKPAHGRVEPLNHVEAERQRR

EKLNQRFYALRAVVPNISKMDKASLLGDAIAYINELQAKLKSMESEREQQLATTSRDGSS

SALQTNSRPENHQNKTPEINIQATQEGVIVKVSFPIDVHPISKLIQAFKDTEITILESKL

NATNDTVFHTFIIKSQTSEQLTKEKLIAAFSKESNSL

>LsbHLHR15

MASGRFCKDEEDKAMLESVLGTDAVGFFSTAVSKHVFSDVIVPPNLDTGIHKRLCHIVKG

SRWNYAILWQVAGLKSGGYVLKYGEGHCQDPVGGPRNEQERERDEVRRRVLGRIHASWGG

SNSIENVYKKLDDVSDLYMLYLTSVYYVFGFNSQYGPGSSFKCSKPTWASDAGSCLKQYE

SRSFLAKSAGFQTVAFVPLKAGVVELGSMEIVPEEQGFLDMVRATFGESTSGQAKAAPKI

FGRELSLGGDAKSQSITISFSPKVEDDSGFTSDSFEVQALGPNHAYGNSSNGGVGDINEA

KMFPQLGQMAPGNFTSQARVSSIDLVNEESSSPLGDERKPRKRGRKPANGREEPLNHVEA

ERQRREKLNQRFYALRAVVPNISKMDKASLLGDAITHITDLQKKIKLLETEKNMAKGSQL

PLQDIDFQARQDDAVVRVSCPLDIHPVSGIVKVLREHQIIAQEANVSTAQDKVIHTFSIR

TQGGEAAALQLKEKLEASLSKN

>LsbHLHR16

MAAFSYQYNPFLVDHSHSPFMFNINATSPPLPLPSQFHPLHQDIINCVDHQSSKVNTVTE

NEPSSLTKNISPQSSMVLDKLETGDEQVTQKVNPTEKKRRTRNNGPFSTKPKSREIAAEG

RNKKQKKNKQEERKCLDEPPTGYIHVRARRGQATDSHSLAERVRREKISERMKKLQQLVP

GCDKVTGKALMLDEIINYVQSLQNQVEFLSMKLTSVNPMFYDMATDLDTFMVRPEKLNNL

ASPSPPLSSSVSRCNSPKQGTVFADTTTVTPTNIFQTTNDYNHLLDTSASIFLQGQRSNV

VSEDGSHFWEVEEQRQKFLNSHGFNNNLCSFSLI

>LsbHLHR17

MMEVAASKMTPNDESWTSWLCDLDPEDYKIINDINIVPLSQDHNILQQSLSSGSHCSHTT

SSTMSNSSGDVVNSFERPTKTLKTNSPSNIGSHSYVLCFNNPEPEQEQELEPKGKILNYG

KCLTSQGLSENQKKETKRSIVESKKSDSVAKHAQDHIIAERKRREKLSQQFIALSALIPD

LKKMDKASVLGDAINHVKQLQEQVKILEQKNQRKNIESVVYVEKTTKSCSSDEDVSVSDT

SSNSGNGGNNYCHPNRSLPEVEARVSEKNVLIRIHCEKEKGVLMKIIHVIENLHLSVISN

STLLFGTTKLDITIIAEMDDEFRLSVQELVRSIRVGLLQFMKY

>LsbHLHR18

MEQATLGLSGSIHDNTALDSVQFNDEIQGIMVPAPAPENASSFTALLELPPTQAVELLHL

PDCDGTDSRRLPCCHVSVNQKPYLPSSSDGNNLTFPTNAALIERAAKFSVFAGDISTREA

RLFPAVSGANLESVKNEPQETDSNPCSTQECVSDPAVENKNQRTAKRKEREKKAKANSKK

SKSIADETSGDGEKLPYVHVRVRRGQATDSHSLAERARREKINARMKLLQELVPGCDKIS

GTALVLDEIINHVQTLQRQVEILSMKLAAVNPRIDFNLDRLLATDGPSLMDGILPSMAMP

LVWPEIPLSNRQHYQQQWQVDDAFHQPLWGREEDTHNFMTQENSLLSYDSSANSASLHSN

QLKMEL

>LsbHLHR19

MKEQLALAVRSIQWSYVIFWSQSVNRPGVLSWGEGYYNGDIKTRKTSQGAELSSDEIGLQ

RSEQLRELFRTLKPVETSPQTKRPTAALSPEDLSDTEWYYLVCMSFVFNIGQGLPGRALA

NGQPIWLINAYSTDCKVFSRALLAKSASIQTVVCFPFMNGVIELGTTDLVLEDLSLIQQI

KTFLLNIQSVDDPINVRATLNSRNNEDVACMAAFDHNDYNVELIPEVGYEIINRTTSPSG

SSNALQTNQLRDETFMVESWGVMEDDLSNCVHNSMNSSDCISQTIASAPKGRGEDCNNND

QKMTLVDPLSEDWHYQKILAALSKSNDQLTMGMHFQNFHQESSFCVWNKGGPLDCHRPRQ

GTSQKLLKKILFEVPRMHMDGLVESQEENDYREGTRLETEEGMNHVLSERRRRAKLNERF

LTLRSMVPSNSKDDKVSILDDAIEYLRKLEKRIKEMQGQRDPSDIESRNKRTHHDMMERT

SDHYYNNKTNNGKKPMVKKRKICDIDETRRVIYSDGLKGSSTSDLSVKMSDNGVVIEMKC

PCRSGRILEIMEAVNNLNIDFNSVQSTEADGSLHLIIKSKFTGSTNATAKRIKQALQKVV

ASKF

>LsbHLHR20

MSNHPSDTPADDFLEQILGLPNFTSADGTDASSLASPMMLQLNSGDAATHLAGGAGFHAP

VYHLGLSLDQGTGGFLKPDDASGSGKRFREDVVDTRPKNTFHGQPMPTTVPTAPHPPAMR

PRVRARRGQATDPHSIAERLRRERIAERIRALQELVPSVNKTDRAAMLDEIVDYVKFLRL

QVKV

>LsbHLHR21

MGSESIAPMVETSKNRSSPCKMNKGKVPKRIHKAEREKMKREHLNDLFLDLANALDLNDP

NSGKASILCEASKLLKDLLCQIQSLKKENVSLLSESQYVTVEKNELKEENSSLETQIEKL

QGEIQARMAHSKPDLNAPPQLELELPEQTKFAGYGLQMPTIEPSLQQGPAVLVVPFRTDL

QAAFPAPNVTDLIPNPTSVISKPHARYPTPADSWPLQLLGEQPTSN

>LsbHLHR22

MELPQSRPCGAEEAGRKPTHDFLSLYSNSTAEQDPRSSSAQASYLKTHDFLRPLEQVETK

SSAKEEATDEISSAVQKQPPLPTPASVEHLLPGGIGTYTISHINSYVNNTQRVPKPEASL

FTVHQATSADRNDDSNCSSHTSSGFTLWEESEIKKGKTDKENNVGEKPIIGVADSAAKLG

PWTTTERISQSFSNNRHGSFNSRSSSQTVGQKNQSFLEMMKSAQDCAQDEELENEGTFFL

KRETLDTQRGELRVRVDGKSTDQKPNTPRSKHSATEQRRRSKINDRFQMLRELIPHSDQK

RDKASFLLEVIEYIHFLQEKVHKYEGSFQGWNNEPEKLMPWRNNDRPAETFQPRGTNSGS

NPSPTLLFPSKIEEKNIPISPTIHASTPNVESGLSTTAAFKTIDHQSGIMNKTFQIPTSS

QPNIFPSTQIGGSGGTVSQLRHRLASDAENIIYQPSVESQTMTSTNEKLKEKELTIEGGA

ISISSVYSQGLLDTLTHALQTSGVDLSQASISVQIELGKQAKITPNVPISMCVSKDDEDP

SKNQRKIRTRVAGSEKSDQAVKKLKTCRS

>LsbHLHR23

MLHCLNASGNLPTESCSSDITVQERQSETIKWQQYQQQQQGQGYFNSAVFCSSVQQVQNS

QDLVPLQIPSAISRTFSSPPVLIDATIEKENCSKKRKSEKAHHNHKLKVVDEIENKEKRI

KLGAEDGESKITGYPSTKKNINSNKNNKENYAGEGSSNSKGNSKASEPQKLDYIHVRARR

GQATDSHSLAERVRREKISERMKYLQDLVPGCNKITGKAGMLDEIINYVQSLQRQ

>LsbHLHR24

MQPTPGRGGGGGVKRFRSTPASWIESFNLKEEEEDEIQQQDNFSFTQLLSNNAAAGPSTP

DSHPYLPDYYHYSSPHTPTAGNVSNNPFTQGVEESDNALDLKIDKILEDSVPCKIRAKRG

CATHPRSIAERVRRTRISDRIRKLQELVPNMDKQTNTAEMLDEAVAYVKFLQNQIEELSE

HQQRCTCTIHE

>LsbHLHR25

MNRGVVQSSPVQQMMASNPNWWNINNMRPPSPQPPPFFSNPNSNFLIPNFTPNSSFSSSL

PFPSWHDNHQDLPESWSQLLMSGIVGEEDKIGMSQFQNQMLITQAPNSSHVDVKQESSGN

SYAYGHVNEELNSCVTSFNTNNMLDFSNNNNNTDLRHLPPDLSSECNSTASGGAMKKARV

QQATTQSTFKVRKEKLGDRITVLHQLVSPFGKTDTASVLLEAIGYIRFLQTQIEALSLPY

LSNGSGNTRQSHSVQGDKNCLFPEDPGQLLHENSLKRKAAEEVSQEEAKKDLQSRGLCLV

PVSCTLQVGSDNGADYWAPALGGAFR

>LsbHLHR26

MECTPARKTQKADREKLRRDRLNEQFVELGNILDPDRPKNDKATILGDTVQLLKDLTSQV

SKLKDEYTMLNEESRELSQEKNDLREEKASLKSDIENLNNQYQLQLRTMYPWPTMDHSVM

MAPPSYPYPVPMPVPPGSIPMQPYPYYANQHPAIISNPCSTYVPFLAPNTIVEQQSTQYV

SPPLHPGSRSHISGKQESKNKSSRESRAERNADSNDVATDLELKTPGSSADQDLSSAQKR

SNKSPSRKENSYTEGSSLGKCSSSHSVQDSSSSSVVGSRKASE

>LsbHLHR27

MSEREEFEVDGKKGPLDWRFVSGNLANSSMGLVSMENSMMGCSPSCSNSMVDSYGSHFLD

LLPSSESFGFCDVNGHSNGNGKDGLCFARVGCDDRTLGFGWNVASSMMKRDGVLTNGHEM

FPQSLSQFPTDSGFIDAAQMPCFNAGGFGDMVNSCRIPQSTALHVSRPVEYPGSDGIPLQ

NDGRSDCPVMSLDEGKQALGGSCDEVDRAESTGEGDDGVAVGSHDGSQMLDCTSGEPSIK

GLNSKKRKRSRQDGDSDNAVGTPELPRETAKESRQKGEQQPNSKAKASGKNAKQGSQASD

SANEGYVHVRARRGQATNSHSLAERVRREKISERMKFLQDLVPGCSKVTGKALMLDEIIN

YVQSLQQQVEFLSMKLATVNPHVDFNVERLLPKDILQHRPVPSSTLGFLAEMPMAFPPLL

HPSQQGLVRSSLPNMANSSDILGRTVEPQFTPLTGEFKEPDQVNPLQEAKH

>LsbHLHR28

MSVMYEEIRRNQEIDSKQHYHQQNNSTLSTPLFTNLIDNTSNHHHGYINNEESFTTENYL

PSTSSEMDTMLSKLMSSNNGWNNCEEPLEEFDVKNVKKEVGESVGQNGDYSYGGSELIYQ

GFSSGSGNGFYGSFGGGNFRDSEDCAQAKMGVRSCNNLVRQKSSPAGFFSHENGLTTLRE

DTNGHETPHGTLNFSSMPSTCLKRMPQIAENRIQSLEANGDSKTQHMPSFTNEFWDNSSF

NAQKTETEDEIMFSTSNGLESHEADFCYQNLGLTHHLSLPSSSTKITSSIEKFLQIQDSV

PCKIRAKRGFATHPRSIAERVRRTRISDRIKKLQGLFPKSDKQTSTADMLDLAVEYIKDL

QEQVQILTDCKEKCKCASHEKQHSRHCS

>LsbHLHR29

MSDKEKFEVDRNGDPMSYSSGMPPDWRFGGGNLVNSSVGLVSIGDSMKINRGDLVGSSSC

SSASMVDSYSPNFWDHSANSQNLGFCEMNIQHNGSSSNAVGIRKDGFGFGRGGHGEIGWN

QANSMLKGDGFLPNGQGFFPQSLSQFPTDSGFIERAARLSCFGGGNFGDAMNSYGIPQSM

AMYVGGAVHGGRDALAAGVGLKIATGGQSQESSDPNVVEAATKGVSPSIEQLVARGSPLM

NDKSESRTMSQDEGKQTLVRNANDSDRGESGDDDGGGGRQGGSPMLEGTTSGEPSIKGLN

SKKRKRSGQDADNNKVNEAQELQSEGAKDNPENQPKGDQQPTSTTKVSGKNAKQGSQTSD

PPKEEYIHVRARRGQATNSHSLAERVRREKISERMKFLQELVPGCSKVTGKAVMLDEIIN

YVQSLQRQVEFLSMKLATVNPRLDFNIEGLLAKDILHQRPGPSSALGFPLEMSMNFPPLH

QSQPGLIQSVIPNMTNPSDILRRAIHPQLSGGFKEPNQMPDMWEDELHNVIQMSFATTAP

TSNEDVDGTNASNQMKVEL

>LsbHLHR30

MPLYELYRLARENLGNETNTTSVADQPSSPENDFFELVWESGQISSQGQSSRGRKSSPSC

RSLPSHCLPSHSPRGRDKDVGGGGYGNNPKIGKFGDLESGLNEIPISVPSREVDEMMPWL

DYSMDGSLQTEYGSDFLQELHSDIPASNSFTLFDKRSNGSQIFRDSNKDSAEPMNVSKGS

SAEQVETARNKASTNQLYPPSSNQCQTSFVTVRSKESDRTENNNPSNGNQDVPYGEITRI

PSSSGDFSNLKAQKQDPKMSGNGSNVMNFSHFARPAAIVRANLQHIGLKSGLVSASARAD

SMGIKNRGEASTSSNPPESTLVNSSGECSKEPEIHCQKVAVQSKADLKPLQTKSLETNAV

ASKQSEPVCKESGIKKDQPSNPVLGDGSAKVQTAAEKGMEAVAVASSSVCSGNGADRGSD

DPNRDLKRKSRDTDDSECHSEDVEDESVGVKKGGSGRGVAGSKRSRAAEVHNLSERRRRD

RINEKMRALQELIPNCNKVDKASMLDEAIEYLKTLQLQVQMMSMGAGLYMPQMMFPPGMQ

HMHAPHMAAFSPMGIGMHMGLGMGYGMGIPDMNGGSSRFSMMPQMQGTHIPMSGPSAING

MARSNPQGFGLPGQGISMPMPRAPVFPFSGGPVLNSSAPGPSAYGSTGNAETVNPASVSG

SKDPMRNVDSQIQQSTGGRDLTNQIPNQSALAHNGGHPSVVDDSGAANPGNITL

>LsbHLHR31

MAGNCSEGLGDDFFEQILAVPESGVGYGRNTTGMDHVGGVLQLGSTPGIMPLGLNLEQTH

HGFLRHQDAATRFVDNIVDVETSITNGNNNNHHHLRLHDINNNNNTSSSPSSTPGITDRD

SMQHMRGLFSTFGQMHTPTHAQPVRPMLPSPTPQPQIHLHHQHQQQHFQSQQPNPASVTA

MPQQPPGIRPRVRARRGQATDPHSIAERLRRERIAERMKALQELVPSINKTDKAAMLDEI

VDYVKFLRLQVKVLSMSRLGGAGAVAQLVADVPVSAVEGEDIEGGTNQQAWAKWSNDGTE

QQVAKLMEEDVGAAMQLLQSKALCIMPISLASAIFRMPQSDSSSIIKPESNNHT

>LsbHLHR32

MKAGKVNHEEDEYDEEDFNSSKKQGTSSAPNTNKDGKATDKASVIRSKHSVTEQRRRSKI

NERFQILRDLIPHCNQKRDTASFLLEVIEYVQYLQEKVQKYEGSYQGWSQEPSKLMPWRN

SHWRVQNFVGQPPVVKNGSGPALPFPGKFDESNISISPTMLSGTQNMMDHDLSRDIVRKT

MERQPDLASKGIPLPVLPMHANMSVPVRSDGVFSHPLQGTVSDTQSSECPTTSEQLNQQD

ELTIEGGTVSISSVYSQELLNNLTQALQSAGLDLSKANISVQIDLGKRANKEPSGTTSSP

KDHDNSLSGNQNIAHFRDVDIREDSSQAQKRMKAYK

>LsbHLHR33

MESDLQQHPTMFLDHQNHHHQPQMNSGLTRFKSAPSSYFSNIIDKEFYEHLFNRPSSPET

ERVFARFMNSLSGSGSGGGDAESASVAVAVAAGDDDSLTQNLLTVQQQLPIVKEEIEQQS

QTLMNSMNNETVDVQQLQRQQSNMNNYGSSGPQKFYQSSGRPPLPNQMKTGRGSCSNLIR

HGSSPAGLFSNINIETGFAVMRGIGTIGAANSTSKEGNFSSSAVLLKNVRAPNYSSVLGG

EIGNSSNPQNNLESEGFAETRGNDFIPGFPLGSTWEDTAMISDNITGLKRYRDDDDVKPF

SSGLNAADTKNETGGQTPATPLAHQTSMPNTTAELAAIEKFLQLSDSVPCKIRAKRGCAT

HPRSIAERVRRTKISERMRKLQDLVPNMDKQTNTSDMLDLAVEYIKDLQKQVETLSQNRA

KCTCSHHQ

>LsbHLHR34

MERYTLPLPQGLHNSLTIPWTQPQQQHQEQQSPSSWSTPNSEPKLNTNDQDIAIAIAMAA

STTSLPFFKPEPDNFYNLNSTTTTTTNNNNVVPFVSNHTSTNDNFLMHQNTNTMDSISNP

HPFFHNNNNNYFFNNTNNNNNPFEMGFENGFFMGNNNTNASNSPVFMGGSLDLSSASEFP

PSLELDASVAPFSASFSMPLELSQPQPQQQQQQQPTTLFQKRRGALEIPRLETVGNKKKR

KVEKKWEEDGNGGGEDDVEDFSELNYDSDENGNDLNNSNGTVVTGGDQKGKKKKGLPAKN

LMAERRRRKKLNDRLYMLRSVVPKISKMDRASILGDAVDYLKELLQRINNLHNELESTPP

GSLLQPSASASFHPLTPTPPTLPCRVKEDLYPNDLLSPKNQSPKVEVRVREGRAVNIHMF

CTRRPGLLLSTMRALDNLGLDVQQAVISCFNGFALDVFRAEQCREGQDIPPEQIKAVLLD

SAGYHGLN

>LsbHLHR35

MMAGNPNWWNMHPQSLNIPQYMLGSSSIPFNSLSENAQVPPQSWSQLLFTGLPGEEERLG

FDHFQPKNTENWDVQILNPSSRVPIMDVIKHEVSQSENFYNQGHHHHEEFHTSGLGSSWS

HMVPVSSPSSHVTTSLSSDNILDFTYNKLDHSKNQLPDQISECNSSTVGVNKKARVQPSS

SQAPLKVRKEKLGDRITALHQLVSPFGKTDTASVLLEAIGYIRFLQSQIEALSSPYLDTA

ASKNMMRNQHSVHVERNSVFPEDPGQLLDDTGLKRKGAPIPNQNVEGNKIKDLRSRGLCL

VPISCTQHVGSENGADYWAPAFGSGF

>LsbHLHR36

MQVRKEKLGDRITALQQLVSPFGKTDTASVLHEAIDYIKFLHDQVNVLSTPYMKNGSPIQ

HQQGCDNVKELERKKQDLRSQGLCLVPISSTFPMTNETSIDFWTPTFGGTLFGR

>LsbHLHR37

MNHCVPDFDIQIDDEEENPVLLSKKPSAARNNEIMELLWQNGSVVMHNQSHRQSKKPPSP

VTNTGNQVIPDHREIRSSDAENFNINQHLFMQEDEMASWLFDSINEDPPIINTETLFHPS

TGVIPGSSLLQSEQLLPQPIPSASRPPIHPARKEDQVLNRKHNNFTYFANHSNASIEPSI

SSSSMIPARQETTMVDSCDTPIITEMTNTASKLSETIKSTAYTECVSVSTAGKAAAYTGC

GTISQVGKAATATTKGVKETSKLDITMTSSHDCSSGSVDPIQRKLELERKRKGRVSDKSE

LQRQEGKKQVRGSTSTKRSRAAEVHNLSERKRRDRINEKMKALQELIPRCNKSDKASMLD

EAIEYLKSLQLQVQMMSMGCGMVPMMFPGMQQYMPTMGMRMGMGMGMSLGMEMGMNRPVM

SFPNILPSSPLPPTTTTVATPYGSRFSVPPFHMPQPHVPTVPESSDNPLNSLGTLLPDQS

RIPNTNFADPYQQFLGPHQLQQLIQGMNQQNVNRPGNSGGQGNSEKHQAG

>LsbHLHR38

MNSIWTDENSSVMEAFMSSSDLSSLWLPTPNSAASTTTPGPDTTKLPSQQQPLFNQETLQ

HRLQALIEDAKESWTYAIFWQTSYDYSTSRQLLGWGDGYYKGEDDKEKAKKVILPEQQAH

RNKVLRELNSLISGSSGSDDVVDEDVTDTEWFFLTSMTHSFVNGSGLLSQAYFNSSPVWI

NDRLSMSTCERTRAAHVHGLQTLVYIPAPSSNGVVELASTEIIPHSAGIMEKVRFLFDFN

NPEARSWPLNSGDNDPSSMWLDIPGSGGIEIRDSVNTVSAVSVTTSANTTITKKLPFEIH

GASSSVPETSTAVNISTGHRENQNQNQNQTFFPKEFNFSGSFKPESGEILNFGESKKSSY

SSANGNFFPGPSPFAANEENRKRRSPVSRSSIDDGILSFTSGKLLQASGIKSGGGDSDHS

DVEVSVVKEAVSCRIMEPEKRPRKRGRKPANGREEPLNHVEAERQRREKLNQRFYALRAV

VPNVSKMDKASLLGDAISYINELKSKLQGLESSKGELEKELDTTRKELEIATKKPVLLNE

EEKEKTNNNSKLIDLDIDVKIMGWDAMIRIQCSKKNHPAAKLMAALKELDLDVNHASVSV

VNDLMIQQASINMGSRFYTQEQLLSVLSSKIGDTQ

>LsbHLHR39

HRFIMYRPPSSSASTSSSSSQQQQSSISQTGLTRYGSAPGSLLTSTVDAVLGGSRLLPGT

GHYFSGDSSHLQPQQQQQHQQQRSSYEGFDGSSLVRQKSSPAGFLNHLATLNHNNSAGFT

ITRGGNGGSRLKSELSFTGGGQGQECLSRISENGVDYAAVAAGNGSLHNSNWGGGPDSNN

NNNNNNSNSIVFSSSVSQTQTNNKRSSRNDDDPDLLLHCLNALESQYSLPQTSMEMDKLM

HIPQDSVPCKIRAKRGCATHPRSIAERERRTRISGKLKKLQDLVPNMDKQTSYSDMLDLA

VQHIKGLQTQVQKLHEDLENCTCGCKQNT

>LsbHLHR41

MMGETSDRLMPFQCPGKNYSPPPTVYQKTTVDARHGGLEIYSDQSSVLLQSMSNMMTAEN

PKLEGVKPSNERKCEGNNNSFKDTSLKSDKNVSPVLHNSVTDDNSISANVLVSPYLRADT

EMSTFQTESYYEDISYASKFPAGYELHEALGPGFLKESKYFDWAVQGNQDSKDAEMSDEF

SCSQLTSESRPEHLLEAVVTKVCHSNNVDNELSFSTSVHDAMVSGRNPEVPIHNVSTINS

EGYSIDQPSHVKENKHNHSLSSSGICGVISPKSFSSACPSSCSNQFERSSEPSKISKKRA

RPGESCRPRPRDRQLIQDRIKELRELVPNGAKCSIDSLLECTIKHMLFLQSVTKHADKLH

KFSETKTKLHDHMEKDIHGSSGYQHGSSWAMEV

>LsbHLHR42

MDSLELGNTDSWDFLDYSFIDPPPTDFLWSNPSDFASVNTEIDIRSSNFASVSADIGIPS

GVVACQEENNTRKRGRAESCHKAGTKACREKLRREKLNERFCDLSAVLDPGRPVRTDKPA

ILDDAIRVLNQLKTEAEELKETNGKLLEEIKCLKAEKNELREEKLVLKADKEKIEKQLKT

LPISPAGFMPPPPMAAYQTRVNKMAVYPNYGYIPMWQYLPQSARDTSQDHELRPPAA

>LsbHLHR43

MNQRHHHPPLNSSITPSSTAPPDSPPQHITNRKHTVRERSEVESHDPTAARKVQKADREK

IRRDRLNDQFHELGNALDPDRPRNDKATIISETIQVLKDITAEVDKLKTEHKAFSEESRE

LIQEKNELREEKA

>LsbHLHR44

MQSLNSLFNSSSPPPQIPIPLHNHTQIQINNDDTFQQQDDFLKQMLSNLPPSSPWNNPKP

LWDPNSDDNLTFPYDEQTNLSSKFRNHQITDKTAAALMLLMPSAADSGLLHIPADFDSSQ

NDVVNASSAGDGSVQALYNGFSGSLHGVANQTHHFQPPQVQSFGSGSVSATNQAPVSGAP

AQPRQKVRARRGQATDPHSIAERLRRERIAERMKALQELVPNANKTDKASMLDEIIDYVK

FLQVQVKVLSMSRLGGAAAVAPLVADMSSEGVSDCVQANGNGGVHPRNPKTSSSNESLTM

TEHQVAKLMEEDMGSAMQYLQGKGLCLMPISLATAISTATCHNRNPLINAPNNINPITAS

NGDGPSSPGMSVNSTVKDAISASKS

>LsbHLHR45

MELSQLGFLEELLAPRKDTWNTLSTGLNDLLLPNGWNFDSFDENLLINPSLNPSFASFST

PLDHRFECSYGTDAVSYPFVDGFTVPELDDSTPLLPQEGVEEFGFVGSESKGLEEGKISC

KVEEHVSEIPVFDMGLCGGGGGQKKGKSKRVEGQPSKNLMAERRRRKRLNDRLSMLRSIV

PKISKMDRTSILGDTIDYMKELLERIGKLQEEMDEGTDQINVLGISKELKPNEVMVRNSP

KFNVERRDQDTRISICCATKPGLLLSTVNTLEALGLEIHQCVISSFNDFSLQASCSEVAG

QRNCMNPEEIKQSLFRNAGYGGRCL

>LsbHLHR46

SVSTGYSKRPNEACKSNRKRLKPGENPRPRPKDRQMIQDRVKELREIVPNGSKCSIDALL

ERTIKHMLFLQSVTKHADKLKKTGESKIISKEGGLVLKDNFEGGATWAYEVGSQSMVCPI

IVEDLNTPRQMLVEMLCEERGFFLEIADLIKGLGLTILKGVMEAHNDKIWARFVVEANRD

VTRMEIFMSLVRLLEQTMKGNASSSNAIDDMLGYNSLPQKA

>LsbHLHR47

MMDFQETSSANSTPAVSESGSKTKLSGGRGKKMKSYETEEEKAKEVVHVRARRGQATDSH

SLAERVRRGKINEKLRCLQNIVPGCYKTMGMAVMLDEIINYVQSLQHQVEFLSLKLTAAS

TYYDFNSEADDLETMQRARASEAKELAMYKREGYGGISCFQPTWPL

>LsbHLHR48

MARFSKSNQDEELLDDDDQDLYTVNTSSALNINNNNNVKVDEPGRGKRANPHRSKHSETE

QRRRSKINERFQVLRDLIPQNDQKRDKASFLLEVIEYIQFLQEKLQIYEQSYEGWNQEPS

KLIPWRNHHGPAENTIDPSRAIPNGSVHETRNNVSLSLPKNVHNPIEPDPSTTIQKGCTP

GSSAEAAPLTMQMRLDMFDPVVSSGMVTQQMLELPVSSADMASNLQPQVWLSKPNKDDYI

VPDNTLKEVEELKNDSGSESDSISTAYSQRILGTLTQALQSSGVDLLQTSVSVQIDVGRR

TNTGLTPSQYSSKGHENQYMSNEAMACDYFSDDSEQSPKRFRREAS

>LsbHLHR49

MGGEEKAMEFQHGNENIMTSEMDMSSVSMANKPSSEIVNHHHPFLASSAWDNPLVSLSQA

HTFGGSSMVSHNEFANTNSTYNPLVLENHQGISSTSHLVQYSNLSGMIHKVPSYGSGSFS

EMVGSFGQHGDHVANTSGYPIPPQHYNHVKDAGIQRGQIHGEQSQVEDSIHEDGALGSAP

SSGNRRKRGFDQNSNFSPNKNAEGDGVKDSPRKISDASKEHEKKPKVEQSGKQAKDNSPS

GDTSKDNFIHVRARRGQATNSHSLAERVRREKISERMRLLQELVPGCNKITGKAVMLDEI

INYVQSLQQQVEFLSMKLATVNPEMNFDVERLLSKDILQSRLGLGIGGFVPGISSSHPFP

SSSFQGNLAGLPSSSTQFPPLPQNVLDHDFQSFYGMGYDSNTALDNLGPNGRLKPEL

>LsbHLHR50

MPPCFNTLDYSLDQQYQQFTKYRVGETSGENNNGMEDNYLPQTQNSGGFYGTTNNSFDKM

SFADVMQFADFGPKLALNRQESEIDDDPVYFLKFPVLNNKMEDQNLMLHQDGVGENEDRF

KVADNLRDHEETRVSDENNSVQLVQETNCALVQENSKKRKRPRTVKTSEEVESQRMTHIA

VERNRRKQMNEHLRVLRSLMPGSYVQRGDQASIIGGAIEFVRELEQLLQCLESQKRRRLV

GEAQLKQVGDSTQQQAPFFQQAPLPNEQMKIVEMESGLEEETAESKSCLADVEVKVLGFD

AMIKILSRRRPGQLIKTIAALEDMQLIILHTNITTIEQTVLYSFNVKVASDTRFTAEDIA

SSVQQILSFIHA

>LsbHLHR51

MDEDILKTLESDESSMDMMTMMMQMEKFPDFHQPFYSYNHNINLNNSHNEFPYGNSNVTY

PQPPFSHPQQPMTPSLQHNGVQIPSGRINNASFSSYSDKKNSMAAMREMIFRMAVMQPVN

IDPETIKPPKRKNVKISKDPQSVAARHRRERISERIRILQRLVPGGTKMDTASMLDEAIH

YVKFLKKQVQTLEQVGANRPMNVAGFPGMISNGNVNYSSFLRGCSSPCQMVGPTSKQMLS

>LsbHLHR52

GGGGGGGGLPPHYPRHGSAATSNSAMDRSFGLVGSLGMNHETSHKSFGSNLLRQGSSPAG

LFSNISFQNGFAAMKGTGNYAAMNGSNGNVSPSINRLTCQVSFPSRNASSLGVLSQISEI

DSEDIEATSPGDGGSNGDTAHYGSGFPYSSWNDTQSFSENLSGLKRGRIGSEKMFSDFQS

GGLENQVHTLSHHLSLPKTSSEMIAMEKLFQFPDSVPCKIRAKRGCATHPRSIAERMRRT

RISERMRKLQELVPNMDKQTNTSDMLDLAVDYIKELQKQFKSLSEKQSNCKCMRMQQADT

NQIP

>LsbHLHR53

MSSHMEISSIRGLPELGIIEDPNFLHQWNNHLNSIDTTNLSASDFGGALQKHFLSNTPNF

NNKTCMEASERAAKQLKNNSWNYNSSSLQTSETQYANLLSFADSNYANQLGLLKPKSEMM

VCPKIDSSTTTIANMLMNQAHLSENHQNHVFKAFQEAKDVETRPSKPSQAHDHIVAERKR

REKLSQRFIALSALVPNLKKMDKASVLGEAIRYLKQMEEKVSVLEEEQKRKKTVESVVIV

KKSKLSYDAEDSCSDTDNTFDETLPEIEARFCERSVLIRLHCLKSQGVIEKTVSEIEKLH

LKVINSSALTFGNFTLDITIIAQMDVGFCLTLKELVRKLRSAYSSFM

>LsbHLHR54

MENINDEEYKHYWETNMFLQTQELDSWGLDETFSGYYDSSSPDGAASSKNIVSERNRRNK

LNQRLFALRAVVPNISKMDKASVIRDAIEYIKHLHEQEKKIEAEIMELQSGMPNNIINRS

CDFDHELPVLLRSKKKRTDQFYDSVNSTNSPIELLELRVTYMGENTTVVSLTCSKRTDTM

VKLCQVFESLNLKIITANITCFSGTLLKTLFIQANEDDKDLLEIKIQTAIASLNDPLSPM

SV

>LsbHLHR55

MEMEDFSSTRMNEYGSSSSLVLDRESGELVEASVKLERKGVSPERTIEALKNHSEAERRR

RARINSHLDTLRTVIPGANKLDKASLLAEVITHLKELKTKEAQASEGLMIPKDSDEIRVE

SQEGGLNGFPYSIRASLCCEYKPGLLSDIRQALDELQLMIIRAEIATLGGRMKNVFVIIS

CKEQNFEDAEYRQFLAGSVHQALRSVLDRFSVSQDILETRKRRRISIFSSSSSLGDFL

>LsbHLHR56

MYFSFPKGYGGPGKCFDSGQNLWLKSVSDYCVRSFLAKSAGIQTVVLLPTDFGVLELGSV

RILPQSFELLNNVKSLFSLSNSITQSSSSLYPSPSVINEGRDDESGISNGVHVPPPKVAL

NLNNGRSHFREKLAIRKMDSSINFPSSRNGVSASTSWGKNQGQGEVFGSIHGVRKGFSNY

QPQRQQVQMQMQIDFSGTKSRVNSVRPVIGESGLVAADVDADQANDERRPRKRGRKPASG

RDEPLNHVEAERQRREKLNQRFYALRAVVPNISKMDKASLLGDAIAYINELQAKLKVMES

DKETFGSSTSRDGSSNTRSENPRQVPPPHDVDIQASQDEVIVKVSCPIDTHPISKVIETF

KDAQIGVAESKLTAANDTIYHTFVIKSEESEQLTKDKLIAAFSGESIPLQTQTTLSI

>LsbHLHR57

MEQTAFESIQFNEEIQGIMAPASETTSSFTALLELPPTQVVELLHSPEITGKPPRHVISP

KPYPLTSDTDNLIFPSNTSLVERAARFSVFAGENLANSPLPEVKDELPEIEEGGCVSDRT

VENKNSKHAKRKEPEKKVKASSKKSKSVADENSGNGEELPYVHVRVRRGQATDSHSLAER

ARREKINARMKLLQELVPGCNKISGTALVLDKIINHVQSLQHEVEILSMKLAAVNPIIDF

NLDSMLATEGVSLMDSNFPTAVAPVVWPEIPHNGNRQQFQQPGQSEAFHQLLWGREENTL

NFMTPENSLLSYDSSANSVSLHSNQMKMEL

>LsbHLHR58

MDRASILGDAIEYLKELLQRINDLHNELESTPAGSSLTPVSSFHPLTPTPSSMPSRIKEE

LCPSSLPSPNGQPARVEVRLREGRAVNIHMFCARKPGLLLSTMRAMDSLGLDIQQAVISC

FNGFAMDIFRAEQCKEDQDVHPEQIKAVLLESAGFNGMM

>LsbHLHR59

MNNNINGWVEEREGENTTTSPSSWPNTNPNTPSPASTLLQNKQHFAAFKTMLEIDDDDEW

YMKDMAFPPNLDTVLLNTVDSVSSSSCSPSSTVFNALDPSLSNLQYLLPQNHKPVDTLSS

LLNNPFEIPCEAGFLEPQGSALPPSPKMGSLVDFTATEMMSLPHLAQTSNGFMGFQNSEE

GSGKSLFLNRPKVLRPLDSLPPSGTQPTLFQKRAALRKNMGKGEIGEGSDKKRKFSGGDE

IEDLSFDGSGLNYDSDDFTEGNRKNRGNASNGNSGVTNQKGKKKGMPAKNLMAERRRRKK

LNDRLYMLRSVVPKIS

>LsbHLHR60

MALETIIYDTISATPFSSTSSYHHDTIESCCFLENAMSYEQQQYHEGVIFDQNNRKREFM

EQDETMSSHQVVEGRKKRRRKPRVCKNKEEAETQRITHITVERNRRKQMNEHLAVLRSLM

PESYVQRGDQASIVGGAIEFVKELEHILQSLEARKLQLFQQELALQQNNIEETSNVSKLN

NMKPPFAQFFVYPQYTWSQTTNNKYTSKTKAAIADIEVTLIETHANLRILTKTRPGQLTK

LVAGFQTLFLSILHLNVTTIQPLVFYSISAKVEEGFQLGSVDGIATAVHHLLGRIEEEAS

LCC

>LsbHLHR61

MEHPLINDCTFSSANPSLSEIWPHFPSQNKRNHSLSENDSATKHIKLALPENDQNAAFKS

KPNAISPKKQQQNEKRSSEPPPPPPKQDYIHVRARRGQATDSHSLAERARREKISERMKI

LQDLVPGCNKVIGKALVLDEIINYIQSLQHQVEFLSMKLEAVNSRLNMQPSIECFPLKDV

GAQPIDLSGIVFGSQARRGYAQGSQPGWLHMQLAGGLDKTS

>LsbHLHR62

MEESGENWPSDPDLGLIDDATFDDEYHSNEKNLTEMMLELSATLPDSNKIMDDASILDKA

RSYVKQLQERVKKLEQNAKPNNSGTSTYILPEVKAKVSKMEVLITIHCEKQNGVMVKILT

QLQKLHLIVKSSSVLQFGKSTFGITIVAQMGDGYNITVDDLVKTLQTLI

>LsbHLHR63

AASASKSHSQAEKRRRDRINTQLANLRKLIPKSDKMDKAALLGSVIDHVKDLKRKAMDVS

RVINVPTEIDEVSIDYNHVIEDETSTNKVDKFKNNIIIKASVCCDDRPELFSELIQVLKG

LRLTTVKADIASVGGRIKSILVLCSKDSEENVCINTLKQSLKSAVTKIASSSMVSNCPTR

>LsbHLHR64

MEESGENWPSSSDLEICDDVIFEDGEFESYDDEFGDSTDRYERDLREKMLALSATIPGLE

KMDDILILEKARKYVKQLQERVKELEGVGSNNISSDNCGTSNNIIPDVKARVLQKQILIT

IHCEKQKSVMLKILTHLENLHLFILSSSVLQFGKFNFDITIVAQMGDGYNITMDELVKTL

RIVILYPSPI

>LsbHLHR65

ARRGQATDSHSLAERVRREKISERMKLLQDLVPGCNKVTGKALMLDEIINYVQSLQRQVE

FLSMKLASVNTRVDFSIENLISKDIFQSNNSLAHPIFSQDSSAPSFYGQQHQQNPAIHNS

ISNGTMPHNSLDPLENALCQNLGMHLSSLNGFHEAASQYPLTFSEDDLHTIVQMGFGQTD

NRKSPIQFQNLNGTNQLPL

>LsbHLHR66

MAEEQFQGSGNWWETPARNMRFESVEQQQQQQSSSFGGWQQQQQHHDTMSASGSSSIVFH

DTTEKLQPSDSSTSNNDNSNLHMMNLGLSSQTIDWNHASLIRSDKASEGSFRSMLQENLN

SSSTNFDEETGGVGLSNWRQEKLFSTESSNNEFKQVNRGFSLDQTQFSPQYSSGDSNMIS

QMDSSALYGTPSILQGSSMTSFPYPTNNYGLLNSNNELNMNMPCNNWSNNKVPQFLMRTS

PPKQSSTSNQLHFTNNTPFWNASEAPNSIKDVRSSFFPSLQPQFSTPNFDSHSKNISEVN

TVVKKSGSEPAPKRTRNETPSTLPAFKVRKEKMGDRITALQQLVSPFGKTDTASVLSEAI

EYIKFLHEQVTLLSTPYMKSGAPSDIQQNSGKSKKSDGAKQDLRSRGLCLVPISSTFPVT

HETTVDFWTPTFGGTSR

>LsbHLHR67

MRQQNSFSFPGGDHHQVVSPILQQQPWSSMSMQQFHVHDPFVLPQQQTSSSPYASLFNRS

SRVPSLQFAYDHHHHGGSEQHLRIISDTLHQHGSFGGLQYQGGDVGKMSAQEIMEAKALA

ASKSHSEAERRRRERINNHLAKLRSLLPSTTKTDKASLLAEVIQHVKELKRQTSLISETC

QVPTECDELTVDAANDDDEEEYGNGNGNKFILKASLCCDDRSDLLPELIKTLKALRLRTV

KADITTLGGRVKNVLFIAGEDHDHEYCISSIQEALKAVMEKSVGDESASGNVKR

>LsbHLHR68

LHHPLEDLTQEDTHYSQTVTTVLQNQWIDSPSINYINYSTQSSFTTWTNHHFHPPPPPDT

ATSQWLLKYILFTVPYLHTKNHDETSPQTHDTAGVNSNDPSARLRGKGTPQDELSANHVL

AERRRREKLNERFIILRSLVPFVTKMDKASILGDTIEYLKQLRRKIQDLETRNRQMESEK

SGVPVLVGPTEKKKVRIVEGNGGGGGVRAKAVEVVEKEVVASVQVSIIESDALLEIECLQ

REGLLLDVMMMLRELRIEVIGVQSSLNNGVFVAELRAKVKENGNGKKVSIVEVKRALNQI

IPHNNI

>LsbHLHR69

MNKKSQKKHNIVSSCYIDSEHSDSEYYPQLPTPTTTNDSFEKREPKKRGRKPLTGIQTPM

NHVEAERQRREKLNNRFYALRAVVPNVSRMDKASLLSDAVDYINELKAKIEELESENQKE

SKKQKMETIESTVTTTSTVVDQKTTCSSNNNNNNVSALDIDVKIIGNDAMVRVQSENVNH

PGARLMSVFKDLEFQVHHASISCFNEIMVQDVVVVQLPDEMRNEESLRSAIRMRLEHE

>LsbHLHR70

MDSKVQRRVSLQHTFQQLREVTRSSAINKASIIVDASKYIQEMKKKVEGLNSELGIVESS

SSQMDELPMVSVETLEKGFLINVLLEKNKPGMLVSILEAFEDLGLDVLDARVSCEDNFQL

EAVGGDSHKDDSINAQVVKQAVLQAIKNTDD

>LsbHLHR71

MEGESINNKDNSSEPVSYENEEDTNDHDVSEDHHLQNQQFGKIEAQWNCLSKLPYSNYVE

YLSESSTYLPTAEYNLVGNNYQTLGGTNSFNDQGRAFGFKPIGSSSNDYGSRKHVGFWRD

NGEEEEAITAKTETTQNLLNAEGDATWPSDSVGDKHNASRFDPMGVVGDVGPFLLPNPKS

GSSSTKHKSEKARCTDRQRRQRIADNLKALHELLPNPEGGSQAQAYILDDIIDYVKYLQN

QLKELSGSKLQSDSNAIPLVFHEGYGHYIKDQMLNEPLEEIMGKLVEEHSAATSQLLESK

GLILLPIALVEELNQDS

>LsbHLHR72

MENNSELFQFIVANNPSFFDYSSTPMMQPSLCSSSDNNNYYHPFEVSEITDTPSSQQDRA

LAALKNHKEAEKRRRERINSHLDHLRTLLPCNSKTDKASLLAKVVERVKELKQQTSEITE

LETVPSETDEITVISAGGGDFTGDGRLIFKASLCCEDRSDLIPELIEILKSLRLKTVKAE

ICTLGGRTRNVLIVAGDKEDSSIESIHFLQNSLRSLLDRSSSCSDRSKRRRGMDRRMNMP

>LsbHLHR73

RPPKNLFYTPGVDTAYHCSNHNSIKDNNKKMIHKEIEKQRRQEMTTLHASLRSLLPLEFI

KGKRSISDQMNEGVNYINHLKKNIKELSAKRDELKSHSSCRFSIHKNNTTVGVEISTREE

GVPLSKLLEQLLKEGLDVVSCFSIQVNGRLLHSVQCEVIDSKSVDLSELRKKISKINPSF

SCSD

>LsbHLHR74

MDMDSTGGGSSCWLYDYGYDISLAAADFMVSSNHPSSAAADFMAPSDHYSSAAAFNWMPH

SQSQTHIINPPSSNISLEMEYSLDSTTVFESGPSNPLKRLEMEYSFDSTLLENGPSKRLR

TESYASGSKAGREKLRRDKLNDKFLELSSVLEPDTLPKTDKVTLLNDAVRVVTQLRNETQ

RLKERNDELREKVKELKAEKNELRDEKNKLKLDKEKLEQQVKLTSVQSSFVSNAMAAKAQ

TAGHKLMPFIGYPGISMWQFMSPATIDTSQDHLLRPPVA

>LsbHLHR75

MDNHHYESDPNCHLKQLPFGDSIGGVCNKMFSNVNDIQDMNKVKQEHHHQHHYHGNHEVF

GKSFMNPNGYLDGFNSSLNSVGENGKFYQGLPNISPCTKSFSDVISFNSRFGRPVIGIHA

QRPSMKYSNLSESRKQGLHTSSHMRTNSGREGTTREVKKKRSEESLEANLKKPKQDTSTT

NSSSSKVQAPKVKLGEKITALQQIVSPFGKTDTASVLFEAIGYIKYLEEQVQLLSNPYLK

ANSHKDPRGMYFDRKDKDDAKMDLRSRGLCLVPTSCTPVVYRENTGPDYWTPAYRGCLY

>LsbHLHR76

TNSNTDTSVENDHDGGISKLFSDCRNLWSFNYVSPTSSSGETDSNVSKFSNYETLTQTRV

SDKPSTRSVGLNGTSYEALGQTRAPVTRSIRSVSSNGISYKTQIQTPDKSLIRRVGKKAV

GCPFNVELDKTVSQSSSDQYITQRKAINSSTIHNSSSLEGGFSLITDKPPKSKKPRSDKR

PCSTNINFQQPNSSSSSICCSSSTEEPDREAIAQMKEMMYRAAAFRPVSFGIDDIDVIEK

KPKRKNVRISNDPQTVAARQRREKISDKIRVLQKIVPGGNKMDTASMLEEAANYLKFLRS

QIKALESLGNKVNAMDCNNPNSIAFSFDPSFSMQMVPSSYNHHSQG

>LsbHLHR77

FVTTFQSPSSQQQKSFFHDPSTSFLSRLSFLDGIYNVISSHQPSSSLGSLAQQLFDQYTI

LSFDINDDRIPGLAFRNQRRYLELQQVELLALSSTEIQKQFYKEARIKTSVFMGCNKGEI

ELGFLNMSHTDIQTALRSLFPEDFSRQIQQIDQNNNNTNPPSSSSSSMRSLSTAGSPEYS

SLIFNTNPAGTSPSSHHFPDHILGGVNIPPMRPVSNTLPFHLQQLPQITPTQLFPIDHND

AIMRAIQNVLSTPPSQQSYAAHPGASAFGRYRNDKSPIIVGSNFRRQSLMKRSFAFFRSL

NLMRLRERNQAMRPSSNQLHHMISERRRREKLNDNFQALRALLPQGTKKDKASILITAKE

TLRSLMEEIEKLSKRNQELMSEKLTASNKETMKFSSNERINVRVLHVSESSSSDDEPMVV

DLQVNVIGQVSQVDMLIRLLEFLKQVHHVNLISMDATNSNSNIPQLHQITFRLRITQVSE

WDEEAFQEAVRRVVADLIQYQVDQNL

>LsbHLHR78

MLNYTPPNSNMENYGILEDSSTTIQMKNSTNGEHKDMNALMLKNLYTGGDFYSNQNYPNI

GNQTQIHPSINISNLNHYSSSSTTSTLDMNMQSLDLLSSSQDHHLGRFTTNDHENLSFHL

HPMQNQLANRSSSSNSINKPSLLGNGGGETKRVMQSKASQSETALKKSRSSSESRPPSCA

PFKVRKEKLGDRIAALQQLVAPFGKTDTASVLMEAIGYIKFLQGQVETLSVPYMKSSQNQ

NNRLMQGDLAIGDTNGEPKQYLRSRGLCLVPLSCMSYIAGDGSSEVWQQRPNFGGPT

>LsbHLHR79

MALEPTLLYPPPPPQQQDPFTYGFKELFNYNLLTDSEPNYDYSNFNLHNEQQHAFLNNHT

ENWSNNNSSPLMEDQVLNVVSNPSLDTSIPTRSRSKKRRVKTSKNKEEIENQRMTHIAVE

RNRRKQMNQYLAVLRSLMPQSYVQRVDQASIIGGAINFVKNLEQKLQFLGVEKEKEGEFE

TIDGNKNKPFSEFFTFPQYSTSVCETKMGDEVHQSSNTIADIEVTMVESHANLKIRSKKK

PKQLLKMVSSLHGMCLTILHLNVTTSHEFVFYSISVKVEEDCKFGSVDEIASAVYKMLER

IQQESILN

>LsbHLHR80

MLCFGGNYQNEIEIITPTQKSVVTSSDSSSASSCNHTNTVFNNKKRNGSEGQQLGTRAGV

GGQKGPKKTKGDNHPTSTGHAKKKEKLGERIAALQQLVSPFGKTDTASVLHEATGYIRFL

HDQVQVLCSPYLQPSQIQFQNHPGDGDNNGREEEEENTKVNKDLTSRGLCLIPVGSTLHV

AGSNGADFWSPATTTDNNVVSPSTTIPKQ

>LsbHLHR81

LRSSSSSSSSSHLMPGSILSNPLQHANISPLHPTNRPNPSSSVGGGLSGNDTDEYDCESE

EGVEVLTEEAPTKSVPSRSSSKRSRAAEVHNLSEKRRRSRINEKMKALQNLIPNSNKTDK

ASMLDEAIEYLKQLQLQVQMLSLKNGLSLHPMCYPEGLQPLSLSRLSMELSDGNRSTPLN

MTSTLPHPQDNSPLLYASNLPNKNTLTSQPSMSSYPSYVNNAETSFAVESRIPPHKRPLQ

QTSEAVHGEDMLPNQTQQSNAIYSATNLLGGSQGVEEFESGMMVAPSTNNSLQTCIAGRD

QSGAIMRSSGPNAIFTSQLSS

>LsbHLHR82

MILVQSGVRSGWRGDQEGLSRFGNLVLLSSFTIFPGKALWEFILNGEDVEVESVGIKRTA

HGRGGFGSKRNRSAEVHNLSQKKRRDTINEKMRTLQELIPNCNKVDKASMLDDAIEYLKT

LQLQLRIMSMRGGGLYMPMMLPAGMQQMHMSPFSPMGVAMQMWLGARPNPQMLGLPGHGL

HMPMPGASMFSFPMPNVNSQVMQNINDCNSANPMSIQCEATIGGVSSTNDKEAFTLSQED

D

>LsbHLHR83

MAAMREMIFRIAVMQPVHIDPESIRPPKRRNVKISKDPQSVAARHRRERISERIRILQRL

VPGGTKMDTASMLDEAIHYVKFLKKQVQSLEQASNRSSHIGGAGGVVNNFNVMNYSSALM

MKGCQPFQMVGSTSKQLLS

>LsbHLHR84

MIASSFCNADSMFLSEEGIDVRKMMEHKRSICSVDQSNYNSIASKKQKPDLSITTKDRKE

KIGERIVALQQLVSPYGKTDTSSVLKEAMEYIGFLHKQVKLLSAPYLETTPAIQMQDTES

CSLRSRGLCLVPVSFTIGVAEGNGADIWAPIKTTSPGSEKDVSQIQ

>LsbHLHR86

EDEEMNQSINSFHEQEFLKDIILEDPECEPFSYLCSNEIHNNSSNSAGNINTEGGVTSPT

NSILSFDHENIEAIHKSFSSNSIISLERSCVGSPATYLLSFDNSSVEPITEPMSNKRSLK

KDERKVKEATKRLRRSCETVQDHLMAERKRRRELTESIITLSAMIPGLKKMDKCYVLSEA

VSYTKQLQKRIKELENNQNKDDTVNPEIYKWKSQSSSNKKKYSESVLEVEARVMKKEVLI

RIHCEKKKDLVLKIHEMLENFNLTITSSSILPFGASVLVINIFAQMDEEESMRMDELVKN

LKKYLLE

>LsbHLHR87

METMFTDSQHVLVKQEENEPFSSHYNGYSSYVLSSNQIIYQTQKNQGLLNDFDASFLASE

NYDTETNIDSSTRNCSNLIRHKSSPAEFFSNYSLHNGSMNFSSTQSSCSINMPLIVQNDE

REARKNIGKCYMPSFTTDCWDSSTFNPPKTSTINGEIMFSTSNALETQELDFGYQKLGLS

HHLSLPSSSAKMTSMDKYFHIQGSVPFKIRAKRGFATHPRSIAERERRIRISARIKKLQD

LFPNSNKQSSTADMLDVAVDYIKDLRKQLKMLSDTKAKCSCASN

>LsbHLHR88

MKILYTKLNSLLPNYNPKELPLALPDQVEEAINYIKSLEANVKMAEEKKERLLMEKKKRS

RECCFGVPKSPCFEIHEFGSSLEVVL

>LsbHLHR89

MIQISSSNYMPEFGMEDTSLFQEMDSFAFNFDDISYFNSFSETSTPESTNSSHNNNNKRF

HSESTQNNSFPIESPDQSVASATPPTKLQLKASSSSKIISFDNMDARIKKPKTESGYGEN

LNFGSEYDKLENKAATTITNRNPIQARDHVIAERKRRQKLNQKFITLSSILPGLKKMDKA

TILEDAIIHLKQLKERVESLEEQVADTKVESAVFVKRSILFANDDSSSSFNENSDQSLPK

IEARVSGKHMLIRIHCEKHHGRSATAILNKLEKHHLTIQSSSVLPFGKKYIDITIVAEVN

KEYCLTIKDLIRSINQVLKQLI

>LsbHLHR90

MDPFFPENLLLGDVFWDEPLLSTTNFVQTQQPCPIQNPSAFVQYRDQPKISLGKQNSLKG

SNSHSMNKRMFAFLRKSLPSERIKAAECERERGFKHMISERMRRQRQRQCCSNLHSVLPH

GTKTDNNSVVQTAAKEIQRLQGCKEELERKNFVLEGNIEGRKLQHLRVTYSSTSGIDSIV

ETLKLLKGHGVDTRRVKTDFSQQEFFAVLEIETEIARADVEEAVK

>LsbHLHR91

ESLIDKDSDKINGEKPKVERKSTEACKSHREAERRRRQRINNHLSTLRSLLPNTAKSDKA

SLLAEVVQHVKRLRKEADVVANRWNDEPSSSCSGEPGSVVSGEEAEAWPFPGESDEATVG

CCGEEEGGARRMKVTVCCEDRPGLNRDLAQVIRSVRANPVRAEIMTVGGRSKSVVVVEWG

DDGREGKEVEALERGLKAVMENRAFVDSGMGPLLLGR

>LsbHLHR92

QEPGQSKHVGGRCCLPKTFILSFDNSTVIPATTSQPCVNSEAKRDSKNKRSRESSEKMKR

NEEKVVKKGRNSFQCADHIIAERKRRQELTERFIALSATIPGLSKTDKASILRAAIDYVK

QLQERVHELEKQDKNVGVTSMMVLNKPNLRGIINNNEGDKNSGETSSDDDDCNKNILPEI

EARVMGKEVLIEIHCEKQIGIELKVLKHIE

>LsbHLHR93

MNEHLKVLRSLTPCFYIKRGDQASIIGGVIEFINELHQVLQSLESQKRRKSLSPSPNPSP

KTLQPTFHQFDISSGGIEANNTFKELGASCNSSVADVEVKISGPNVILRVISQRIPGQVS

RIITVLESFSFEVLHLNISSMEETVLYQFVVKIELGCQLSLEELAMEVQQSFCSEAVKLI

AV

>LsbHLHR95

YVAQTRPTKKIKTSINPSSYSSPQLISFEHFNATPVASNELYNLDYSDVKPKLEKGCNEN

KDFAANYDIRANQTRNTAQAKEHVMAERKRREKLTRSFIALSAILPGLKKMDKASVLGDA

IMYMKKLQARLHTLEAQVEDNKKTGSAIQVKRSVIFSTDDHDDDSNSNNQTLPEIEVRVS

RKDVLIKIQCDKHSGRATTSTVLGKLETLNLTVQSSNLLPFGNNIVDLTIVAQMNEENCV

TAKDLLGSIRHALITNK
