# Supplementary material for: Genome-wide identification, characterization, and validation of the bHLH transcription factors in grass pea
Source: Front Genet. 2023 Mar 20;14:1128992. doi: 10.3389/fgene.2023.1128992 (PMC10067732; doi:10.3389/fgene.2023.1128992)
Supplement: Supplementary file 8 [file Table2.DOC]

>LsbHLHD2

CTTTAGACTAAATAGTCATGAGTCAGAAAATTCGGATGCCTTGAAAATTGATGTTTTGAA

TCAAATGAGCTGCTTCAAAACCTGATTTATGCTTCTTATCAGATCTTTTATCGTCAAGCA

ATATTCTTTATTCACCTCAGCAACAATGGTTATGTCAATATACTTTTTTCCAAATGGTAA

AACGGAACTGCTCTGAATGGTGAGATGATGTTTTTCGAGTTTGTTGAGTATTGCGGTCGC

ACTGCGACCGTGGTGTTTTTCACAGTGGATTCTGATAAGCATGTGTTTACCAGAAACTCT

TGCTTCGATTTTTGGAAGTGATTGGTCTGAGTTTTCGTTGAAAGAGGAGGAGCTGTCATC

GTTGGCAAAGAGAATAGATCTCTTCACAAAAACTGCAGATTCGACTTTTGTATCGGCTAC

TTGTTCCTCTAAACTCTCCACTCGTTCTTTTAATTGATTTAGATGAATTATTGCATCTTC

CAAAATAGTTGCCTTGTCCATCTGCATGAAAAGCACACGTTGATTATTATTCATTGTGAA

TTATAGTAATAAGAATAAAGATAAATATTTCTAGTTTTCATAATTGAATAAAATGATTAG

TACCTTTTTGAGTCCAGGAAGAATGGAGGAAAGAGTAATGAATTTCTGGTTAAGCTTTTG

CCTTCTTTTTCTTTCGGCTATTACATGATCTCTTGCTTGTATCGGGTTTCTGTTTGTTAT

CGTTGTCGCAGCCTTGTTCTCTAGTTTATCATACTCACTTCCAAAATTCAAGTTTTCACC

GTACCCCGACTCGGTTTTAGGCTTCTTAATCCTTGCATCCATATTATCAAAGGAAATTAT

TTTGGAAGATGATGAAGCCTTGAGTTGTAGCTTTGTTGGTGGTGTTGCAGAGGCGACAGA

TTGATCAGGACTTTCAATGGGGAAAGAGTTGTTTTGTGTGCTTTCTGAATGAAATCTCTT

ATTATTATTATTATGAGAGGAATTAGTACTCTCAGGTGTGGATGTTTCTGAGAAAGAGTT

GAAGTATGAAATATCATCAAAGTTGAATGCAAATGAATCCATTTCTTGAAACAAACTAGT

ATCTTCCATTCCCTGTTCA

>LsbHLHD3

TCAATTTTATAACTGTGAGAGATCCAATGAAGCACACGTGCACGGAATCGAAACGTTGAT

TTGTATTCCAACAACCAGCGGAGTTGTCGAAATGGGTTCTTATGAAAATATCCAACAAAA

TTGGAACCTCGTACACCAAGCTAAATCCATGTTCCAAACATCCTCTTCAGAATCAAACTC

AACTCAAAACTAGATCTTCTTCCCACCAACCCACTTGACAAAATCCAAACTTTCGACCAA

AACATCTCATTCTCCGACATCCGTATCATCTCCGGCGCCAGCGAGGAAACCGAGAAGACA

CAAAAAACAATGAACAAAAAATCACAAAAAAAGCACAACATTGTTTCATCATGTTACATA

GATTCTGAACATTCTGACTCAGAATATTATCCACAATTACCAACACCAACAACAACAAAT

GATTCTTTTGAGAAAAGAGAACCGAAGAAGAGAGGAAGAAAACCACTAACCGGAATTCAA

ACTCCGATGAACCATGTAGAAGCAGAGAGACAGAGGAGAGAGAAACTAAACAACAGATTC

TACGCTCTAAGAGCAGTGGTTCCAAACGTTTCAAGGATGGATAAAGCTTCATTGTTATCA

GATGCAGTGGATTACATCAACGAGTTAAAAGCGAAAATCGAAGAACTAGAATCAGAGAAT

CAAAAGGAATCAAAAAAACAGAAGATGGAAACAATCGAAAGTACCGTAACAACAACTTCA

ACTGTGGTGGACCAAAAAACAACATGTAGCAGTAACAACAACAACAACAATGTTAGTGCT

TTGGATATTGATGTGAAGATTATAGGTAATGATGCTATGGTGAGAGTTCAATCCGAGAAT

GTTAATCACCCTGGAGCAAGATTAATGAGTGTGTTTAAGGATTTGGAGTTTCAGGTACAT

CATGCGAGTATATCATGTTTTAATGAAATTATGGTTCAAGATGTTGTGGTTGTTCAACTT

CCTGATGAGATGAGAAATGAAGAGAGTCTTAGATCTGCGATTCGAATGAGATTGGAACAT

GAATGAATGAACGAACAAGAAGAAAATTATAATCCTAATAATGATT

>LsbHLHD4

ACTCAATGAATGATAAGCTGAAAAATAACATGTTACCCATTTATGAGAAAGATACATACA

CATATATACACACACGGGCACGGATACATAATATTACATATTAAAATAGACTTAGTTTTT

TCTTACTTCATCAAAGCTAAATCTATGACAGTGAGAAAACTGTCCCTAGCATATTCTAGT

TCTAATACCCCATTACCATTTAACTTCTCAAATTAAATTACCTCAAAATTCATACTAAGC

TACCAAAAACAAAGACACTACTAGTTTACAGAGATAGCTTCCGGGAATCAGTCAAATATC

CCAGACCCTCACAATTTTGGAGGAGTGAGAGATTCACAGACATATCATTCCCGGTTAAAT

TCTCTCATTGCAGCAGATTTGCAGATTCTATTGAACATCCCCGACTTGAGAGGACAATGC

TGCCCGAAGCTGCTCCTGGGTGTAAAAACGACTCCCCATTTTCACAGTTGCTTGTTGTAT

CATCAAGTCATTCACCACAGACACACTGGCATGGTGCACTTCTAGGTCAAGCTCCATTAA

TGCCGCCATCAACCTCGCTGCAGGGTGGTTTTTCTTACTGCATTGGATCCTTATCATTGC

ATCCCAACCTATAATCTTGACGTCGATATCCAAATCAATTAAAGCTTGATTGGAAGGTGA

TGATTTTTTGGGTACTGGTTGCTGTTGTTGTGGTGGTGTGGGTGGGGGATGAGACGAGTT

TTCATTGATTTTCTGAAGCTCACTCTTCACTTCATCAAATTGCTTTTCTAATCCATCTTT

ATCGGATTCTGTTTTCTGAAGCTTTGTTTTCAGTTCAGTAATGTAAGATATAGCATCGCC

CAATAGTGAAGCTTTATCCATCTTTGAAACATTAGGCACAACCGCACGAAGAGCATAGAA

TCTCTGATTCAGCTTCTCTCTTCTTTGTCTCTCCGCTTCAACATGATTCAATGGTTCTTC

TCTTCCATTTGCTGGTTTCCTTCCTCTCTTCCTTGGCTTCTTTTCTGGCTCCACCACACG

ACTGCTATCCACCTCCTTCACCACCGAAGCCTCCAGATCCGAATGGTCTGAATCACCACA

ACCAGTACCACTTGAGAATTTCAAATTCGACGCCGCCGGTGGGACAATTATGCCGGAAGT

AAAAGATAACATTCCATCATCGTTGTTGCTTCCTCTTGAATTAGGCGATCTCCTCTTTCC

ATTTCCACTGTTGGTGTTATTGTTGTGGTTTTCCTCAGCAACCGATAGTAAATTTGATTG

ACCGGAGAAGAAATTCGAATTTGAATTTCCATTATTAGTAACATAAGAAGTCTTTTTACT

CTCGCCTCCAAAACTCAGAATCTCACCGGATTCCGGCTTCAACGAACGCTGATGATGGTT

ACTCCCACCGTATTCAGAAACATTCATTTCCTTATTAAAGAAACTTTGATTATTCTGCTT

GCTTGAAACATGAACAACACTAGGAACTTCCGTTAAAGTACTTGAACCATGAGTTTCAAA

TTGTATCGTCTTGGTCACACTCACACTCAAACTCTGATTATTGTTATTGTTCTGAGGTTG

TTGTTGGTGTTGATGTTGTTGGTGACTTGGATTTGAAATCGAAGTGTTTGTTGTTGTTGG

TGCAGCAAGAGAATTATTATCAACGGAATCTCTAGTTTCAGGATCGTTAAGCCAAATCGA

AGAAGGATCATTTTCACCTTGATGAGCTATTGTAGCAGAATTACCTAACTGCCAAGAAGA

TCCAAACTCAAAGCTATTATTGAAATTGAAAAGCATCTTCACTTTATTCATCAAATCATT

GTTTTGATAAATCAATTCCGTAGATCCAAGCTCCAAAACACCGTTCGGCGACGGTATACA

CGCCAACGTCTGTAAACCGTATTCCTGACCCTGTCTCGCTCTCTCGCACACCGAGACGGA

GAGACTCTCGCCGCCGACCAACCACACCGGAGTTGAGTTAAAGTAAGCTTGTCCGGGAAG

GCCGCCGCCGTTGACAAAAGACTGAGTCATAGAAACTAAAAAGAACCATTCCGTATCGGT

AACTTCTTCATCGACGGCGGATTCGTCGGTAACTGGATTACCGGAGATTAGAGAATTAAG

TTCCCGGAGAACTTTTCTCCGGTGTTCTTGTTCAGCAGGTGAAGAAGCTTTTGATTTTGA

CTTTGATTTCGCTTTTGTTTTATCTTCTTCTCCTTTGTAGTAACCGTCACCCCAACCGAG

AAGAGAAGTACCGGAATAGTCATAAGACGGTTGCCAGAAGATAGCATATGTCCAAAGTTC

CGATGCGCCTTCAATTAAAGCTTGAAGCCTTTGCTGGAGTGTGTCTTGGTTGAATCCGGT

GGTTGTTTGTGGTGGCTGAGACGGCGGCGCCGGTGGCCATAAGGTGGATAAATCGGAGGA

GGTCATGAAAGCCTCCATTACTGAAGAGTTATCGTCGCTCCAGAGATTCATTTACTTAAG

TTGAAACGAAATGGAAATTTTTTGGATTAATTTGAGGGTAAATTAGGGAGAAGAAAAGAA

AAAACAAGACAAGCTCACGCAGAACACACCGTGTGGTGTTGATAATGGATTGGGAGGAGA

GAGAGAGAAGGGACAAATGAGGATTGGTTATTTTTGGGTTTGGAATTAATTTGGAGTGAA

TTTAGGTTGAATTTGAGAGTGATTGTAAGGTTTCCTTTCATGAAATGATGACAAAGCCTG

TCATTAATTAATTATTTGCAAAATAAAATAGAAAAGGCGATGTAGTCTATACTATAGAAG

TATTTCGTTCAGTTTTCAAAAATAAAATAGAAGTATTTCTTTCTTGGGCTATGTAAAGGA

GATTGTTATAATTTTGAAGTATATCTTTTTG

>LsbHLHD5

TACACTTCAAGAGACAACCTCTTCAAACTAACCAAAAACACATAGAAATCACCTATGAAG

AATTTATAAAATGGTTAACAATATTATTAATACACATAAACGTGATCAAGCTGAGTTCAT

ATTTATGATAATTGAAAAACCCGTTTCCGATTAGAAGCGGAAGTAGATAGACGAGCGAAA

GCCGCCCCGCCCCAAGACTATTATGAACAAAAAAAAGAAATAGTCTACTTTACCTACATA

GAGAGATGTCTCCTAATTCTACTACTACTGATAAAATTACTTCAAAAATATAAAACAAGC

TAATCAAAGTACTAGTACTGGCTAAAATTCATAAAAAGGAACACAATTGTTTTACAAGAA

AGCAGAGAAAATTTGCAGCATTCAATCTCATTGTGTATCACCAATTTTGGAAGAAAGAAC

CGATAAAAGCTGTTCTTGTGTGTAAAATCTACTTCCCATGTTAATAGAAGCCTGTTGAAT

CATCAAATCATTAACAACAGAAACACTTGCATGATTCACATCAAGATCCAACTCTTTTAA

CGCTGCCATTAATTTCGCTGCAGGGTGATTCTTCTTGCTACATTGAATCCTAATCATTGC

ATCCCATCCCATGATTTTCACATCTATGTCTAAATCAATCAACTTAGAGTTGTTGTTTGT

TTTCTCTTTTTCTTCTTCATTTAACAGAACTGGTTTTTTAGTTGCAATTTCTAGTTCCTT

CCTGGTTGTATCTAGTTCTTTCTCCAATTCACCTTTAGATGATTCAAGCCCTTGTAGCTT

GGATTTCAATTCATTTATGTAAGAAATCGCATCTCCGAGAAGCGAAGCTTTGTCCATTTT

AGAAACATTCGGAACCACAGCCCGTAAAGCGTAGAATCTTTGATTCAGTTTCTCTCGTCG

CTGTCTCTCTGCTTCCACGTGGTTCAAGGGTTCTTCTCTTCCATTGGCTGGTTTTCTACC

TCGCTTCCGAGGCCGCTTCTCCGGTTCCATAATTCTGCAGCTCACAGCTTCCTTCACCAC

GGAAACTTCCACATCTGAATGATCGGAATCTCCGCCGCCAGATTTTATACCCGAGGCCTG

TAACAGTTTTCCAGAAGTGAATGAAAGAATTCCATCGTCGATACTACTTCTAGACACCGG

AGACCTCCTTTTTCTGTTTTCCTCATTGGCAGCAAACGGTGACGGACCGGGAAAGAAATT

ACCATTGGCGGAGCTGTAAGAACTCTTTTTACTCTCGCCGAAGTTCAGAATCTCGCCGGA

TTCCGGTTTGAAAGAACCTGAAAAATTAAATTCCTTTGGAAAGAAGGTCTGGTTCTGATT

TTGGTTCTGGTTTTCTCGGTGACCAGTTGAAATGTTAACGGCGGTGGATGTTTCCGGTAC

ACTAGAAGAAGCACCGTGAATTTCAAACGGCAACTTTTTAGTGATTGTTGTATTTGCTGA

AGTGGTTACACTAACGGCGCTAACAGTATTGACGGAGTCTCTAATTTCAATCCCACCGGA

ACCGGGGATATCCAACCACATGGAAGAAGGATCGTTGTCGCCGGAATTCAACGGCCAAGA

TCGTGCTTCTGGATTATTGAAATCAAACAAAAAACGAACCTTCTCCATTATACCAGCGCT

ATGTGGAATTATCTCAGTAGATGCGAGCTCAACGACACCGTTTGAAGACGGTGCTGGTAT

ATATACCAAAGTCTGAAGCCCGTGAACATGTGCTGCGCGGGTTCGTTCACATGTGGACAT

GGAAAGCCTATCGTTGATCCATACCGGGGAAGAATTAAAGTAAGCCTGGCTCAGTAAACC

ACTGCCGTTCACGAAAGAGTGTGTCATCGAAGTGAGAAAGAACCACTCAGTATCGGTGAC

ATCTTCGTCAACAACATCATCTGAGCCAGACGAGCCAGAAATTAAGGAGTTAAGTTCTCG

GAGGACTTTATTGCGATGAGCTTGTTGTTCAGGCAAGATAACCTTTTTGGCTTTTTCTTT

GTCATCTTCGCCTTTGTAATAACCGTCTCCCCAACCGAGGAGCTGTCTGCTGGTAGAGTA

GTCGTAAGAAGTTTGCCAGAAGATGGCGTAAGTCCAGCTCTCTTTTGCGTCTTCTATTAA

AGCTTGAAGACGGTGTTGGAGAGTTTCTTGGTTGAAGAGAGGCTGTTGTTGTGATGGAAG

TTTGGTTGTGTCGGGTCCTGGTGTGGTGGTTGATGCGGCCGAATTTGGTGTTGGAAGCCA

AAGAGAAGAAAGGTCGGAGGAGCTCATGAAAGCTTCCATGACGGAGGAGTTCTCGTCTGT

CCAGATACTATTCATTGTCGGAAGTGAGCGGTAATCTCTCATTCCATGCAATGGTCAGCT

GTGTTTGTTGAGAGAGTGAAGTGTTTTGCGGCGGAGCGGAGGAGGGAGTGAGAGTTGTTG

CTGATTTTGATAATGGGAGAATTTGTTGTTGAAAATAGAAATTTTGTTCTCTAGAGAGAG

AGTGAGAGGGTAGTGAGAGAGAGTAGACAAGGCTGATGACTAATGAGTGTAGTGCATTTG

TTTTTGAAGGAGAGACAAAGAGAGAGTATAATGTGTTGGTGAGGTTTCGAGGAAAAAGGG

CACACAGATTAAATTGCAAAGATAGGTCGGAAAATGACGGCTTCTTATTTTCATCCTTCA

CTCTAGAATTTATCATTATTCTTTTACATAAAATTATTCTAGAAAATCTACCAAGCATTT

AATAACTCTTTAAATTGCACAA

>LsbHLHD6

CTAAGGTTCTTTTCTTCCTGCATCTATTAGATAGATACCTCAAATAGTCATAAAAATGGT

GAAACAACTAACAACAAAAAATATATATTAGAACTTAAACATTTCAGTTACATAAATAAA

ACAATGGACATTGTAGGAACAATGTTTCTGATAATTAAACTGTGTATACTATAGACAAAC

ATAAATTGCTTGTCCTATACACAGGTGTGTTAATACTAATAGAATCTAGCTATATATTAT

TCTACTTCTGTCTAAATGCCACTGCTTTGTTCTTAAGATGGTTTTTCACCTGCACAACAA

AAATTTCTTACGAAGGTTTAGAAATTGGTTCCTTCTCTATGTATGATATGCCTATATGGA

CAATGTTGTTTGTGTTTGTAACGGGATGGATTCGCCGGAAAATGCTGCAATCAACTTGTC

CTTTGTGAGTTGTTCAGATTCTTCGGACTTAATTACAAATGTATGGTAGATAGTATCGTT

TGCTGCAGTGAGTTTCGACTCCGCTACACCGATTTGTGCATCTTTGAATGTTTCGATTAC

TTTCGAAATAGGATGAGTATCGATAGGACAACTCACCTTCACAATTACTTCGTCCTGTGA

AGCTTGGATGTCAACGTCGTGAGGAGGAGGAACTTGACGGGGATTTTCTGATCTTGTGTT

GGATGATCCATCCCTCGAAGTGCTGCTTCCGAATGTCTCTTTGTCAGACTCCATGACCTT

GAGTTTCGCTTGAAGCTCGTTGATGTAAGCAATGGCATCACCGAGTAGAGATGCTTTGTC

CATTTTGGAAATATTCGGCACAACAGCTCTTAAGGCGTAAAAACGTTGATTAAGCTTCTC

GCGTCTTTGTCTCTCCGCCTCCACATGATTGAGAGGCTCGTCCCTACCACTTGCAGGCTT

CCTTCCGCGCTTCCTCGGCCTCCTTTCATCATTAGCCTGATCCGCGTCAACATCAGCAGC

AACAAGTCCCGATTCACCAATAACCGGTCTCACAGAATTAACCCTCGATTTCGTTCCTGA

AAAATCAATTTGCATTTGCATTTGAACCTGCTGCCTTTGTGGTTGATAGTTGCTAAAACC

CTTTCTAACACCATGAATAGAACCAAAAACTTCACCTTGACCTTGATTCTTTCCCCAACT

AGTAGAAGCAGAAACACCATTTCTAGAACTAGGAAAGTTAATACTACTATCCATTTTCCT

AATAGCAAGTTTCTCTCTAAAATGTGAACGACCATTGTTCAAATTCAAAGCAACCTTTGG

TGGAGGAACATGAACACCATTTGAAATTCCACTCTCATCATCTCTTCCCTCATTGATCAC

ACTAGGACTAGGATACAACGACGATGAGGATTGTGTGATAGAATTCGAAAGTGAAAACAA

TGACTTAACATTGTTCAACAATTCAAAGCTTTGAGGCAAGATTCTAACGGAACCCAACTC

AAGAACGCCAAAATCAGTAGGTAGTAAAACAACAGTTTGAATTCCAGCAGATTTAGCCAA

GAAAGAACGAACACAGTAATCAGAAACAGATTTCAACCACAAATTTTGACCAGAATCAAA

ACACTTACCGGGTCCACCATACCCTTTAGGAAAAGAAAAGTACATAGAAGCTAAAAAAAA

CATCTCTGTGTCGGTTACACGGTCAAGGCCAAAAGCGTAATTGTCTTCATCAGAACCACC

AAAAGCTGTGTGTAGTTTCTGTAAAACCCTCTTCCTCATATTCTGTTCAACTCTTTCTTG

TTCTTCATTTCTTCCAAGGGTTAAAGCATCACTCTCTTGTTGTTCTTCTTTGTTTGGTTC

TCTGCAACATCCATCACCCCAACCAAGAACATAATCGCCGGATTTTGATTGAGAAAGTTG

CCAGAAAATAGCGTAGTTCCAATTGAAAGTGGAAGAGTTTTCGACAAGATCAGAGAGTTT

GTTTTGAAGAGAATCAGAATAGCAGTAGCACCAACAGCATTAAGATAGTTTCATTGGATA

AACAATTTGTTATCAAGAAATGGAAAGGTCGAGCGCCAAGAACAGTTTGAAGCATGGATT

TATCTTGTTCGTCCCATGATAAGATGCGGTTTGTTGAAGCCATCTCAAAAAGAAAAAGAG

AGGGAAATGGTGGTGGTGAGTGATAAAATAGACGCAGACATTAAGAAAAAAGAAGAGGGT

TTTTGGAAAATGAAAGAAAGAATTAAGGGAGTTATGTTATTGTTGGTGGTGGTGTGGTGA

TTGAGAACGTGAAGGAAGGGGTGGTGTGGTGCGTGTGTGAAAATTGAAGGAGAGAGGAGT

GTGGCGTGATTGTGTGTAGGGTAGGGTAAGAGAGAGAGAGAGAGAGAGAGTTTTA

>LsbHLHD7

TGCATCTGAGTTGAAGAAAATATGTCATGAACCACACATAAAGTACCAATAAGAAAAACA

CAAAGTAAGAAACTATTGAACCTATAAATTTTAGCATCTCCTAGATGTTTCCTATGAAAT

CATTACTATAACACTATCATTATTCTTCCAAAATGCCTCATTCATGTCAGACCAATTAAA

CAAGACATTTGGTTTCTGATATTGACTACAGCCTACTTGTATATGGAACACCAGCAGCAT

ATTGGGTGTCCAGAACATCAATCACCTTGTTGGCTATAGCACTTGCATAAACCGATGAAC

CTTCACTTACCTGCATAATGAAACAAAACAAATAATTTAATATATAACCAAATCAAATGT

AATAATTCTATATAGAATTTACTTGATTTTAATTTTACCAACCTTGCTGTTGAACAAGAA

GCTGCAATATTCACCCACGTGTCCACCTGCCACATGGTTGAGTTCTAGCTGAAGTTCATC

CAGAACCTTGGAGACAAAGAGTAAACAATTAACTTTCTTCCTCTGAAACAGTTTGATTGT

AACATCATCGTCGACGATCCGGACATCAACCTCACTGTCTTTGGATTTCCTCTGCAGCCA

TGAAGTCCTTATGCTTCCATCTGGATCACCAAAAGGCTTTATGTTACAGCTTTCTGCAGC

AGCATCTTCAGCTTTATGTGTCTTGCACATTTCCCTCCCATGTCTTTTCTTCTCCACCAA

CAGTTTCAGCTCATTCACTGTTCTAATCAGCTCTCTTATATACTCAATAGCATCTCCAAC

CACTGACGCTCTATCAACCTGCATTACACCCAAACTGCCTATTAATTAATCATTTCAATC

ATAGTTATCAAAATAGAGCTTCAAAAAAGCTCATAGATGGTTATTGTTGTATACCTTAGT

GGGATTTGGGATGAGACCCCTCAAGATTTTGTACTTTCCATTCAATTGCTCTCTTCTTTG

TTTCTCTGTTGCAAAATGTTTTGTAGCTTTTCCTCCTCTTCCTTTGCCAACATTAGCAGC

AACCCTATTGAATTCCAGCACTCCCATATCTCCATCTCCTTCCATCTCATCCCCTCCACC

GAAAAGAGAACCGTTCCTCGAGCTAGTCGGCATGCTATATCCACGAGGCAGAGATTGAAA

CAACTCTCTGAGGGCTGGTGGCTGTGGAGGAAGGTTCAAAGGAAATAGAGGATCATATAA

AACCGATGATCCACTTGTATTATCCGACGAACCAATCGGAAGATCTCCAAGAAAACTCAT

CGAGTTATGGAAATTCGGCGGTTTTTGATTCAGGTTTGTCATACAAATGTTGGATGGTGG

ATTTGCGAGAAAAGACGAATCTGAGCATCTAGGTAAATGGAGAAGGTTTAAAAGATCGGG

TGAAGGGTAATGAGGGTCGAGTATGTTTGAAGGGTTGTAGGTTTGGTTATGGTTTTGCGT

TTCAACAATTTGTTGAAACTGTTGTTCATGTAGTTGGTGTTGTTCTTGGTGATTAGCATA

ATCCATACCTTGTAACTCATGAACATTGGTGTCCCAAGTTGAGTTCTGATACACCTCTTG

CACCAAATTGTTATCAAGGTGCTGTGGATTATGATAAGAGAAATCCTCCTCAGAATGATT

ATGATTATTACCTTCAACCATTTGATTTGGGTGAGTTTGAGAAAAGGGATTATCTTTTAG

AGAATTTTCAATACCTTCATCCATTGTGTTGGGATCAAAGCAACCAGTTTGTTCATGCAT

GTTTTGCTTGTCTCCTGAACATGAACTCATTCCTGTTTGTCTGCAATAGTTAACACAAAT

TTAAAAATTAATCAAACAGAACATACAATGTGAATGCATATATAAT

>LsbHLHD8

ATAAATGGATATTTCATTTGAATCGATGATACTATTGAAAAAAACTAGCTGATTCTATAT

AACCGTGCGATGCAGATGCAGATCTCAGTCGTTCGATCTAAATCAATGATCAAGGTCATT

TAAACTAAGATACAGATTATGCAATTTGAATCGTCTAATCTAAAATTAATGATTGAAATC

CATTCCTTGCGGTAACCGCATAAAACTTCTATCTCTTGAAATTTGAATTCGAGACCTCAC

AAACATCTTCCTCCATACCTGCACTTCTAAATAAAGCTTGCTTAATATCTTCACAACTTA

GCATTGTCCTTTCTCAAATTCCTGTTTTACATAACAAATAAGAACTTACAATTGATTAAA

AATTAGAAACCAAAAATAATAGTATCAACTAATGTATACTACCTCTGAGCAAGAAGCTTG

CATTGTGAACTCATTGAAACAACTTATAACACATTGTTGAATCTCAAGACCTAATGCTTC

CAATGTGTTCACTGTGGATAGCAATAACCCTGGCTTCCCTGCACAACAAATTTCAACCTT

TGTGTCTAGGCCTCTCTCCACCTCAAACTGCGACGGCGAAATTTAAAAAATACACAAAAA

AAAATATATAACGTTAGGCTGAAGATTTGAACTCGTACCAAAAGTTATTTTACAAAAACT

CTTATTATCGAGCTGACTGATAAATGACTGATTACGAACTTCAAAAGGATTAGAAAATTT

TTGTACCTTTGGAGAGTTTCTTATTAAGATTTCATTAGACTTTACGTCTTTGATAATGCT

TGACATGTTTGAGTCTAGTTCAATTTCTTGTTGTAGATTGTTGATTTTCTCTAGAAGCTC

TTTCATATAATCAATAGTATCTCCGAGTATAGCCGTTCGATCCATCTATCGTATATATAC

ACATTGACATTCGACAAAATAGATTAGATCTGCGTAACGATTAATATACTAAAATTATGT

ATTCTAAACTACCTTGCTTATCTTAGGAACAATTGCTCGTAGCATCGATAGTCGATCGTT

TAATCTCTTTCGTCGTCTCCTTTCCGCCATTAAGTTCTTCGAGGGTTGTCCTTGAAGCTT

CTTCGACCGATTCTTTCTCTCCAAACTGTTTGCAGAGTTGAAAACTACCGGCATTTCAGG

TGACTGTGTTTCCTCCATTTTACACGTTGTTTGCAGCTCCAAATTCTTAATCTCTTCGCG

GAGCATAGTTTGATCTTCTTCTTCTCCTATCATGGACAGCGGAAAATCTTCTTGAGAAGC

AAACGGCGCGTCGAGTGTGTTGTTATTATAATATGAATCAAGAATCTGAGGCGTTGAAGT

TTCATCAAGTAATGAACCATAGATTTCATTGAAAGTGTTACTGTAGTAATCATTGTTGAC

GTAACTTTGAGGGAACACTTGTTGACTACAAGATGAGTTTTGATCGAAGCAGTTATCGAA

ACTCAGGTTGTTGTGATTCGTTGATGATGAGAAGAAATGATTTTCTTCTTGAGTTGGAGT

TTCTGTTCTTAGAGCCATTAATTCCTCCATGAAATCTCCATCATAAAACTCCATTTTT

>LsbHLHD9

AAAACTTTGTTTCTCATCCCCATTTTATAAGGAAAAATTCTCCTTCTCAATGCCCTAAAT

GAAACAATCCTCGCTAGCTGTTGAAAATTCACATCCTTAATCTCAATGACAATTTCTCTA

ATAATTTTCTCTTTATTGATTATTTGAAAACAAAAATCTTAAATTTGGTAAAAATGTGTA

GTATTGTAAAACAACCATTAGAAAGTTAAGTATATCCAAAACTCAAAATTAAGCACACCA

GAAAGATTGGAAAGCAACATTAGAGCATGTTTTAAAATGAAATATATAGAGATCCAAATC

ACTAATGAAAAGAACTTATAAGAAATCTTCCAAAGATGAAGAACTGAAAATAGAAACCCT

CCTTCTTTTTCTAGCTCCTAACATATCCTGTGAAACAGAAAATCTACTAACCACAGACTT

AAGAGCTTGATAAACAGAACCAGCAAGAAACTGTCTGTATTCAGCATCATCAAAATTCTG

TTCCTCACAACTAATTACAACAAACACATTCTTCATTCTTCCACCAAAAGTCGCAATATC

TGCCTTCACGATTACGAGACGAAGAGCCTCCAGTGCTTGTCTTATACTAGACAATAATCC

AGGTTGATACTCACAACACAGTGATGCTTTAATTGAATAACGAAAACCATTGAATCCACA

TTCTTGTTCTTCAACACTTATTTCATCGTTGTCCTTCGGTATCACTAAACCTTCACACGC

TTGTGTTGCGGTTCTTTTTACCTCTTTTAAATGTCGAATAACTTCACCGAGTAACGATGC

TTTGTCCATCTGTAATCATAAAAAGGAATATTAATTGGTTTTATCAATTGTTGAAGCTGA

AGTTCTCTGAGACTGAAATTTCAGTCTCTGAAAGCTTCAGCTTCAACATCAATGATTAAC

AAAGAGAGATTAAAAACGGATTGTGCTAAAGTAACTAACCTTCAAAGCACCTGGAATGAC

GCTGCGAAGTGTATCGAGATGTGAATTAATTCTCGCTCTTCTTCTTCGCTCTGCATCGCT

GTGATTCTTCAAAGCTTCAGCGGTTCTCTCAGTAGACACACCCTTTTGTTCCATTTTAGC

AGGTGTTTCCACAAGCATTCCTCTCTCTCTATCCAAAACCAGAGAGAAAGATGAACCATA

CCCATTTCCATTAACACCACTGTTTACTTGAAAACCATTAGTGTGATCACCAAGTATATC

ATAACCCCAATCAAAATCTTCCATTTCCATAGCCAAAAGTAGTACTAAATTGTGAACAAC

AACAATCTTGGAAACAATGGAACAGGAGATGTTTGTTCTCTGTCAGAGTTGTCAGTGTAC

AAGTGAAGTTCTTGTACCAACTAGATATAAGAAGTGGCTTGTAAACTCTACCCGCAATGC

AATACAGAGACTAATCTTTTGAATTTAATAAAACTTAACCGAAATTTCTTATTATAATAT

TGAAAAT

>LsbHLHD10

AACAAAATGAAGGGCAAAATGAAACATGCCAAAATAAATGGGTGATGCTTGATAAGTCAA

AACAGGTGGGCCAGGACACAGACAAACAGGAGTAGAAAATATCATATATTGATTCAAATT

TACCATCAACATTGAATCAAACTATTATGAGTGTAATCAATCAGTGTTTAGACACATGCA

TATATGATTATGGTTAATTATTGTTGCATTTTGCACCCACCACATGGACCACACCTTTCT

TCACATTACGACAATTACGACAAGGATTTGAATGCAGCAACCCGTAGGTTTTTTACAAGA

TCATTTGTCATGCTGTACCCCGTTTCCATCTGCAAAATCACGTGTACTAGTCATTTATAA

ACATCGAAAGCGACACGTGAACAAAAGATACAATAATTTTTTGTTTACCTTAGCAACAAT

GGTTATGTTGATAGAATCCCCAAAAGGTAGGACAGTGCTGTTAACAACACAGAGATCAAG

CTTTTGAATCTCCTTCAATAATTTGATCAAAACACCCTTTTGCTTTTGGCAGAGAAACCT

TATCAGAACATCTTTGTCCAAAACTCTGGCATCCACATGGGAGAGGGACTCAGACCCATC

ATCACTCCATGACTCATGAATTACCCACGGTTTGTTTTCCGTCACTACAGACTCAACTCG

ACTATTCCTGTCCTGTTCTTCTAACACCTTCAAACGTTCTTGGAGCTCTTTCATGTACTT

GATAGCATCTCCTAACACCGAAGCCTTGTCCATCTGTAGAAGGATTGATTCACAACATAA

TACAAAATCTGAAAAGTACCAAACATTTGGAATGAAAGGAATGGTTTAATTTGGTACCTT

CTTTAATCCAGGAATAAGAGCAGCTAAAGCAATTAAGCTCTTGTTAAGTTTCTCTCTTCG

CTTTCTCTCAGCTATGACATGAACTTTATTATTATTGTTATGTTTTGTGTTTAAGGTACA

ATCAAAAGCATGAAAATGAGTAGTGGTAGTGTTCTCAAAAGACATAACTTGAGAATTAGT

GGTAGAAGAATGAGAAGAAACAGGGGAATAAGAAACAGAGCTTTCACAAGACAAAGGTTT

GTCCTGTTGTTGAAGAACACTTGCTATGTCATCTGAAACTAAATCATCATCATTAAACAA

ATTTTGTTGGAACAAATCACAACCCTCCATTTCCATTTCATCCAACATCTGGAAAAAAAA

CATTCATAGAAATTCAGAACAATTTGTCACTAGAGTGAAGTATTAAAAAATTTCTTACCA

AATCAGATAACCAGCTAGTGGATGATGATGTGTTTAATTTTTCCATAACTTTTGATAATA

TTATATAGTAACACCTTTTGCTTCACAATTTCAGAGATTG

>LsbHLHD11

AGAGCACGCTTGAAGTAGATGAGATAGTTGACATTGTAGGCCTTGAAAAAAGAAATATCA

TTGCAAGAATTGAAACTTTAAACACCTTTTGTGTATATATAGTTGACCGTATCTTCTATA

TATTCTTATTCTTTTGGGATCTGTTCAGTATGAGCTATAGGAACGTCCTCAGATGAACGA

TGCAAAAGCTGAGCGTAGGTTTGTCACAAGATCCTTCAATGTCATGCAGAATTCTATATC

CATCTGCAAATTTCACGAGTAGTTTCAATAATAATGCATATTTGTAGTAATTTAGTTCGT

TTATTTGAACTTTCGGCTTTCAGCCAATATAAAAGATACAAAGATTACCTGAGCAATAAC

TGTTATATCAAGTGCACAACTCCCAAATGCCATGACACAGCTATTGATGACTTTTAGGTG

CAGTTTCTCAATTTCATGGATTGATTTTTCTGCAGCTCCTTTGCTTTTCTCACAGTGAAT

TCTTATGAGTACATTTCTATCGCAAAACCTTGCTTCAATTTCTGGCAGTTCCTCGTCGAA

GGGACTGCCAGATTCTGCAGACGAATTCTCAACGTCATTCGTTAGTTGAGATTTCTTCAC

AACCACCACATATTCCACGTTTTTCTTCATGTTCCGTTCCTCCTCAAGAGCCTTCACCTT

CTCTTGCAGTTTCTTCAAGTACTTGATAGCATCTCCAAGAACGGTAACTTTGTCCATCTG

TTAGCATCACATTAAAATTCGAGACCACAAAAATTAATACAGATAGTACTTTAAACACAA

TTCATCACGCATCCTAATAGCGAACAAAGTCCAACATATGCAAATTATGTATACCTTTTG

TAGTCCGGGAACAAGGGCAGAAAGAGCAATGAAGCGCTGGCTGAGCTTCTCTCGCCGCTT

TCTTTCAGCTATTATATGGTCCTGAGGTAGAGAGACTTTATTACGTGTCGATATCCTTTG

TGCCTCCATGATTCCTTGAGAGACCGCGTCAGAAGTTGTGTTACTGTTTTGAGGACAAAC

CATCTCGTCCTTAGGCTTCACTAATCCCAACTGATTAAAATGATTAGAATCGACGTACGA

AAGAAGGTTCTGGTACGAAACAAATTGAGTCTCGGGTCTCTGATCAGTTTCAACCGAAGC

TCTGAGATTGAAGTTTGGATAAATATAAGAAGGGTGTTGCAAAGTGTCTCCAAAAGCAGA

AGCTATTGGCAGTGAGTTTGACTCGTCGATAGAGTTTAAATACCATTGATGAAGACAAAG

AGAAGGATCCTCCATTATTTCCTGCAAGATCAAACACATGCTTTCAAAATGTTCATAGAA

A

>LsbHLHD12

TGTTAATTATTCAAACACTTATCCGACATTCTTTCTGATTAGACACAAGTAATAAAACAA

AACAAAAACTTAATCTGAAAAGATTGGACAACAAAAACATATTCATTATTAGCTTATAAC

TGTTGCTTACAATATTCCAACATTAACCTCAAAACCATATCCAACAATAATATGCATAAA

TATTTCAATTTATATTGGACATGAAAACAAGTTCATAATTCATATATCAAGAAAAATCAA

ATTTCTCTCAATCCTTTATCAACTAAGCATTTGTTTGGAAGTGGGACCCACCATTTGACA

AGGTGATGAACAACCTCTGAGAAAAGAAGAGTAGTTAACATTTCCATTAGAAATCATTCC

TGGGAACCCAGCAACATTCATAGGCCTGTTTGCTCCAACTTGTTCCAACGTTTGAACTTG

TTTCTTCAAGAACTTCACATAATGTATTGCTTCATCTAACATTGAAGCTGTGTCCATTTT

TGTCCCACCAGGGACTAATCTCTGCAAGATTCTTATTCTCTCACTAATCCTTTCCCTTCG

GTGTCTCGCTGCTACACTTTGTGGATCCTTTGAAATCTTCACGTTCTTTCTCTTTGGTGG

TTTGATTGTTTCCGGGTCTATATTAACCGGCTGCATAACCGCCATACGGAATATCATCTC

TCTCATGGCCGCCATTGAGTTCTTCTTATCGGAGTACGACGAGAACGAAGCATTGTTGAT

TCTCCCGGATGGAATTTGAACTCCGTTGTGTTGAAGAGACGGTGTCATTGGTTGTTGAGG

ATGAGAGAACGGTGGTTGTGGATAAGTTACGTTTGAGTTTCCATAAGGGAACTCGTTATG

TGAGTTGTTAAGATTGATATTGTGATTGTAGCTATAAAAAGGCTGGTGGAAGTCAGGAAA

CTTTTCCATTTGCATCATCATTGTCATCATGTCCATGCTGCTTTCATCACTTTCCAAGGT

TTTCAGAATGTCCTCATCCATGAATGTTTCTAGAGACAAAGAGGAATGGAAAAAGGAACG

AAAGTACAGAAAAATAGAGGCTGAGGTGAGAAAAGTGAAGTAGCAGAGTATGGGAAGAGG

TGAGAAAGTAGAATATTTGCAGTAAGGGGATGGAGAGAGTGCAGAGTGTGCCGGTTATCT

TTGTAACCTACCAACAACCACTTAGGCAATTCTACTGAGTATAGGAATGCCTTTTTATAG

CAAATTTGGAATGATTAATATTAAATATTTTTTAAAAAAAATATGAATAGAAAGATAATA

GTAATTTTATCCAAATAACTCTTAAATATTTTGATGTTTTTGTTTA

>LsbHLHD13

GACTGCAGCAAATAATTGAAAAATAAGTAATTTCATAATATGAAACATAGACACAACTGG

CACAGACACCAAATAAGTGATTTCAAAATATGAAACACGGACACAAGTGACACAAACACC

ATCAAGACATACTATAAAACATGAAACACGGACACAACTAACACAGACACAGACAAGACA

ATACTATAAAACACAGACACAACTGACACAAATACTGGACAGAATTTTGACATTGACATG

TTGATACAAATAATAATTTGAAAAAATAAACAAATTAAATTTAATCATAAATATCGATGT

CTGTGTCTGACCCTAACACAAAAGAGACACTTTTTTCAAAGATGTTGATGCTATTAGGAT

TGAAACCAAAACCAAATTAAATCTCCTTTGCATGTTTACCTTGAATTGTTTTTGTAGTTC

TTTAATGTAATCAACAGCCAAGTCCAACATGTCTGATGTGTTAGTTTGCTGCCAAGAACA

TGAACAAATGTCAAACAAAATTATCCAATATGTTCCTAGGTCAAACAAAATTGATGTGTC

TAATTATAGTACCTTGTCCATGTTTGGAACAAGCTCTTGTAATTTCCTCATTCTTTCACT

GATCCTAGTTCTTCTCATCCTTTCGGCAATGCTTCGAGGATGAGTAGCGCAACCTCGTTT

TGCTCTGATTTTACAAGGAACGGAATCCGGAAACTGAAACAACTTCTCCATTGCAATCAT

CTCAGATGAAGTCTTCGGTAAACTCAAGTGATGTGATAATGTATGAACTTGATTTTCTAG

TCCTCCACTCTGATAAATAATACAAGATATTAGTATCATGAACAAGAATCGAAATTTGAA

ATCCAAAACAAGAGAAGAAAACATCCTACACATACCTGAAAATCAGAAAACATTTTTTCG

CTACCGATTCGCCCTCGTTTCAAGCCACTGAGATTTTCTGAGAATGATTGAGTATCATTC

CAAGAACTATAGGGAAATCCAGAACCATAATGCGCAGTATCACCATTGCTGCCTCCATCG

CCAGGGCTAGTTGCTTCAATATCCTCGCTATCTATTTCAGAGATTTGTGATAACACTCCC

AAGGAAGATGCGTTTCTTGAAGGAAAGCTAACTTGACAGGTCAATCGGTTAATAGACGGA

CTAACATTGCCATTACTTCCATTCATTGCAGCATAGTTTCCCGTACCCTTCATGGCAGCA

AACCCTGCAATTTACAAAAACTATCATCAATTGGCATTAAGAATAAATAAAATAACCTTA

ACGCATAGAAGAAAAAAAA

>LsbHLHD14

TTCTCGATAGGATTTTAATCATTGCATCAAATCCTAAAACTTTTACTTCTACATCAGCCA

AACATGACTTACTCTCTGCAGTTTCTTCTTCAAGTCCACTTTCCATCTCAACTATCTTCA

TCTGCTCATTTGGTAAAGGAGCTTGTTGAAAAAATGGAGCTTGTTGTTGTGTTGAATCCC

CTACTTGTTTTGACTGTGCTTCTCCAACTAGTCTTCTTCTCTTTTGTGACTCTAGACACT

GAAGAAGTTGTTCCAATTCCCTCACAAACTCTATAGCTCCACCAATTATAGATGCTTGAT

CACCCTATATTGTTAAATCAATCATCCTTACCCTTAAACGTACAAACCAGGCAATGTAAA

GTTTTAGAAAAATCATTGTATGAAATGAATCTTACATACCCTTTGAACGTACGAACCAGG

CATGAGAGATCTGAGGACACGAAGATGCTCATTCATTTGCTTCCTTCGATTCCTTTCAAC

AGCAATATGAGTCATGCGTTGACTCTCAACTTCTTCGCTTGTCTTAACAGTTCTAGGTCT

CTTCCTCTTCTTGCTATTCTCTTGCACTAGTGCACAGTTCGTCTCTTGCACAAGCTGCAC

CGAGTTGTTCTCATCGGAAACCCGAGTTTCTTCATGATCTCTTAAATTGTCTGCCACTTT

GAATCTATCTTCATTTTCTCCTACCCCATCTTGATGCAACATCAAGTTTTGGTCTTCCAT

CTTGTTGTTTAAGACGGGAAACTTGAGAAAATAAACCGGGTCGTCGTCGATCTCGGACTC

TTGGCGGTTTAAGGCTAGTTTAGGTCCAAAATCTGCAAACTGCATCACATCTGCAAAGCT

CATTTTATCAAAAGAGTTGTTTGTTGTACCATAAAAGCCTCCTGAGTTTTGTGTTTGAGG

CAGGTAATTATCCTCCATTCCGTTATTGTTTTCACCGGAAGTTTCGCCGACACGATATTT

CGTGAACTGCTGGTATTGTTGATCCAGGGAGTAGTCAAGCGTGTTGAAACATGGTGGCAT

GGGTGGTGCCTGTAGAGATTCAAAATTGGAGCAATACAACATGGTGATATACGAAGTTAT

GGTGTATACGATGATATGAAAATATATAACATATATAAAAAGGCATGGTAGTAAGAGAGA

GAATATGTTTGTACGGAATAGTTATTATCTTTCTCCATTATGAACAAGTTGACTTTAATG

GAACGAAACCCTAAAACAAGCTCAGTTGATGCCCCACCAGAGTTTCTTGAGGGCCCTCAC

CTGAGCATAGGATATAATT

>LsbHLHD15

TAGAGGCCTTATCCATCTGTATATATAACAATTATATCTTGATTACTTTACAATTTTTAT

TCTCAAATTACTATCATATGTATATTAGATTGAAAACTTATAAGTAGTCCTAGTGCAAAA

AGAAACATAGTTTAAAGCAAATATATATATATATATATATATATATATATATATATATAT

ATATATATATATATATATATATATATATATATATATATATATATATATATATATATATAT

ATATATATATATATATATATATTCAATGTCTGCAAATCTTAGTTCAACTGACAAAAATTA

ATATTTATAGGTCGGACACCATAACTAGGGCTCGAACCTTGATACTTACAGTTGCATATA

TGAAGTTCTAATAGTTATTACCATCGACATAAAAACACTTACCTTTGTAATGTTGGGAAC

TAAAGAACGCAATGCATAAAGTTTATCCTTCATTCGACCTCTCCTCCTCCTCTCCGAAAC

GAGAGTCTTGGACCGATCAGTTTTAGCCCTAGATTTGGTATCATCGATCGAGGTCGTGGT

TGTTGTTGCCGGAGAATCATCACCAATATTGTCTCCTTCTCCATTAACCTCCTCATCATA

ACAAGCAAAGGAACCAATTGTTGAAGAGCTTGGATCATAGACATTCACAATATTATTAAT

ATTGTTGTTGTTATGATCAAGTAATGGCATTAGAGGAATTGAAAGAGGTTGATGATTATC

AATGAAACAGTCATTGATAAGGTCAGAACCAAAGTTACATATTGCATTTTCATTCTCACC

TCTGATTAAATCAATAAATTGATCAAAGTTTGGATCGTCAACGAAATCACGCAACTCAAA

ATTGTCTGTGTAAACTAGTGAGTCTTGTTGATCTTGACCATTCATATTTGAGTTCATGGT

GGAAACTTGGATATACTTGAGAGGTTTAGTAAATAGTATTATATATAAAGAAGAGTAACA

TAATTCTTATTAAAAATAATAGTTTTGGTATGAATGGAATAAGATGAGTTTCAATATGCA

CTGCACTGGGTCTATCATTTTAAGTTTCAAATTTAAAATAAGTAGTTAGGGATGTATTAA

TATTACTTAATATTTTTTAGCATATCACTTTCACATCATACTGTATAATTAGTATAATAA

TTGCTTTAATGTATTTCTAGAAATAGTTGAATGTGAAGATGATAAGATCCTTTCCTGGTT

TATCATATATAGGCTTAAAAAATTATTTAATTAGTTCTATATAGCAGTAAGCCTGTGCAT

GAGC

>LsbHLHD16

ATTCAACTAACCTAAAAAAAAATGAAACAAGTCTCAAAAATGGTGAAAACAACCATAGAA

AGTAAAATTATTCTTGTTTTAGTTTCTTGTCATTCTCATGATCAATCTCCCTTCCAACAA

GTTGTTTAAGACCCTGCAAGATGGAAGGTTTTGACCTAACATCAACATTGTTGGCCAATG

TTGTGACAATCTCTTTTGGAATAAAGCACTTTTCTTCTGAATACAACTTCTCTTGAACAA

AAGGAGAAACAACTAGTTTGTGTAAATCTTCACTTGGAGGTGGTTCCTTTTCTTCCTGAA

ACAAAACAGTTGAGATTGAAACTTAAGTAATTAACTTAAATTATAAGCTCTAATAACAAT

CTAAAAACAAAAAGCAGTTATTATTATTATTATTATTACCTTGCTAAAAGTTTCCATAAC

CTGAAGCATTCCAACTTGAGCTTGAAGAAACCTAACATAGTTAGCAGCAGCATTAAGCAT

CTCAGCAGTGTTCATCTTAGGCCCACCAGGAACCAACTTCCCAAGCTCTTGCGTCTTCTC

AGTTATCTTCCTTCTTCTTTCTCTCGCCGCAACACTCTGCGTCGAGATACTCCTTTCGCT

AACCTTCTTCTCACATTGCACAACATTAACCATCTTCGAACCCGAAAACGGTTCCGCTTC

AAAGGAAGTAGTCAAAGGGTTCGCTATAAACTCATCCACAAAATAATTGGCAAAATGTTG

TTGCTGTTGTTGCTGTTGCTGTTGGTTTGTTTCTTCATAACCTAGCTTTTGCCGTTTCGC

ACATGGAACATCATAGTTATTGAAATTTTCATCATTTGGGAAAATCTCATTCATGGAAAC

AAAGCCATCGTTTTGATCACAAGAGAAAGTTGAGTCAATGATTAGTTGATCTTCACAGGG

ATAAGTCTCGTTTGAGCAAAAATAAGGACTCGGAAAAACAAGGTTTCGTCTAGAAAAGAA

GACGAAAAGTCGTAGTTTTGTAGTTCAAACAGTTGTTGTTCTTGTCATGATGTTGGAAAG

TGATGGTTTCTGAGTTGTTGTCTATAGTTTGAAGAGTTTGAGAAGGATAAGAATTATTAT

AGTTGTATTCTGTACTAAGGAATCCCATGATAAAAAAAACACAGGGGAATCTTTGTTTAG

ACAACAAGTACGAGAATGTTTGTTTAGACACAACTACGAGAATGTTTGATGTAGACAATT

TAAGAAAGAGAGAAAAGAGTGTACAATGTTGTAGATATTTATTATGTGAGAATGGTTTTG

G

>LsbHLHD17

ATGCAATGACAATAATAGCGCACCAATAACACTTTAACTTAAGGTTTTAAATGTATTGTG

GCCAAATAAAAACTTTTGCAATTTTAATCGGTACAAATATTGTTGCAATTTTAATCATAC

GGAAGAAAGTTACAAAACAAAGATAAAATTAAATTAAAATTAATTTTAAAAAACTAATGA

AGATCAGATACATTAATCATTGTCTTTGCTAATAATAATGAAATGATTAGAGATGATTAT

ATAATTAAAATCAACCCTGTGAATGATGATTGTAACTACTAGGAACCATTTGCATGGAAA

AAGAAGGATCAAAAGAGAAAGCAATACTATTTGGATTATTACAATCCATAGCATTAACTT

TGTTACCTAAACTTTCAAGTGCCTTAATTTGTGACCTTAAAAACTTGAGTAATTTGCAGC

TTCTTCAAGCATTGATGCAGTGTCCATTTTGTTTCCACCAGGTACAATCTTTTGCAAAAC

CCTAATCTTATCACTTATTTTTTCCCTTCTTTGCCTTGCAGCCACAGTTTGTGGATCATT

TGATATCCTAACATTCTTTCTCTTAGGCTTTTTTTCAATCACATCGATGTCGTCGATTCC

GAAACTCACTGGCCTAAATGCTGCAGCTCTATACATCATTTCCTTCATTTGCGCTATTGC

TTCTCGATCAGGCTCTTCGGTTGAAGATGAACAACATATTGATGATGATGATGAATTTGG

TTGTTGGAAATTTATGTTGGTTGAACAAGGTCTTTTGTCAGATCTCGGTTTCTTGCTTTT

TGGTGGTTTATCAGTGATAAGGCTAAAACCACCTTCAAGAGATGAAGAATTATGAATAGT

ACTTGAGTTGATTGCTTTTCTTTGAGTGATGTATTGATCTGATGAGCTTTGAGACACTGT

TTTGTCAAGTTCAACATTGAATGGACATCCCACGGCTTTCTTACCAACGCGTCTGATCAA

AGACTTATCTGGTGTCTGTATCTGTGTTTTATAACTTATACCGTTTGAACTAACACTTCT

GATTGAACGTGTGTCTGGTGCACGTGTCTGTCCCAATGCTTCGTAACTTGTACCATTTAA

ACCAACACTTCTGGTTGAAGGTTTGTCTGACACCCGTGTTTGTGTCAGCGTTTCATAGTT

TGAAAATTTTGAAACATTACTATCGGTTTCACCGGATGACGATGTAGGACTGACATAATT

GAAGCTCCATAAATTTCTGCAGTCAGAAAATAACTTTGAAATT

>LsbHLHD18

TATCATTAATAATGTTGACTGAACTCTGAATTGCATATGCATGTGACATATCCCCCAACA

AGTAATAATAAAACAAAAACAGAAATTAAGCTAAAAAAAGATTGGACAACAAAAAGATAT

TCATTATTAGCTTATAACTCTTGCTTACAATTTTCCAGCATTAACCTCAATACCATATCC

AACAACAATATGCTTAAATATTTCAATATATATTGAACATTATATCAAGAAAATCAAATT

TCTCTCTATCATTTATCAACTAAGCATTTGTTTGGAAGTGGGACCCACCATTTGACAAGG

TGATGAACAACCTCTAAGAAAAGAAGAGTTGTAGTTAGAGTTTCCATTAGAAACCATTCC

TGGAAAACCAACAACATTCATAGGTCTATTTGCTCCAACTTGTTCCAACGTTTGAACTTG

TTTCTTCAAGAACTTCACATAATGTATTGCTTCGTCTAACATTGAAGCTGTGTCCATTTT

AGTTCCACCAGGGACTAATCTCTGCAAGATTCTTATTCTCTCGCTAATCCTTTCTCGTCT

GTGTCTCGCTGCTACACTTTGTGGATCCTTTGAAATCTTCACGTTCTTTCTCTTTGGTGG

CTTGATTGCTTCTGGGTCTATATTAACCGGTTGCATAACCGCCATACGGAATATCATCTC

TCTCATGATTGCCATTGAGTTCTTCTTATCAGAGTACGACGAGAACAAAGCATTGTTGAT

TCTCCCGGATGGAATTTGAACTCCGTTGTGTTGAATAGACGGTGTCATTGGTTGTTGAGG

ATAAGAGAGTGGTGGTTGTGGATAAGTTACGTTTGAGTTTCCATAAGGGAGCTCGTTATA

TGTGTTGTTAAGATTGATATGGAGAATGTTGTTGTTGTTATAAAAAGGAGGGTGGAATTC

AGGAAACTTTTCCATTTGCATCATCATTGTCATCATGTCCATGCTGCTTTCATCAGTTTC

CAAAGTTTTCAGAATGTCCTCATCCATGAATGTTTTTAGAGACAAAGAGGAATGGAAGAA

TGAAAGAAAGTACAGAAAAATAGAGGCTGAGGTGTGAAAAGTGAAAGTAGCAGAGTATGG

AAAGAGGTGTGAAAGTAGAATATTTGCAGTAAAGAGATGGAGAGAGTGCGGGTTTTTATA

ATCTTTGTAACCTACCAACAGCATGATTCTACCAGGATATTGGAAAATCTTCTTATACAG

CTTCCTTCAACTGCTTCTTAAATAAAAAGTTAGTAAAGTAATT

>LsbHLHD19

TTTCTCCACCTCAAAGATATAGTATATGCAATACTTTATTTATAACAAACAAACAAAAGA

GTATGTGATGCCATAAGGTGTCTTGATTTCCAAGGTTATCCATTTTACAATCAATCAAAC

CACACATTACAACAAGGGACCAAACCTCTTAACTCGTGCGGATCCACTCAACAAACTATC

ATGTATCTCACCACATATTCATCCTATCATGTGAATATTAATGAGAACAAAACACACACA

TTTATTCACATCACTAGTAGCAACTACTAACTAGTACTTCATGAATTGCAACAGTCCAAC

TCGAATGCTTCTAACCAGCTCCTGCACACTTAGCCTGAATTCATCATCCATCTGCATTTT

CACCACATCACAAACCAACTAAGTAACATCAAACAAAATCAACACTGTCGTATAATGTTA

AAAATATGTTACCTCAGCTATGATGGTTATGTCAAGTTTCGTGGTTCCAAAAAGCAAGGT

ACTGTTACTAATGACAGAAAGATGAAGATTCTCTATCACGTGTATTATCTTCATCAACAC

ACCTTTTTCTTTCTCACAGTGAATTCTTATCAGCACGTTTTTCTCTGACACTCTTGCCTC

AACTTCTGGCAGTGACCTATTCGGGTGACAATAATTATTACCACCATTTCCAGAATTTGA

TGAAGTGTCTGAAACTGAAACATCTTCATCTGAAGAACATGACTTTGTTGTCTTCTCAAC

ATAAACCACAGATTCAATGTTTTTCCTTTGGTTCTTCTGTTCCAATATCTTCACTTGTTC

TTGAAGCTGTTTCACATGGTTTATTGCATCTCCTAACACAGAAGCCTTATCCATCTACAA

ACCCATATGATTAATTAATTAATTAATTAATTAGTAACCCTTATGAATCTAATTAAGAAA

TCAACAATAAAATCAATTTGGGTATCATTATTAAGCATAAAAATTGAACCTTTTTTAAAT

CTGGAATCAGTGCTGAAAGAGCTATGAACTGTTGACTAAGTTTTTCTCTTCTTTTTCTCT

CAGCAATTATGTGATCTTGAGCATGTTTTGCAACTGAATCACTCTTTTTGCTCTCTACAA

TACTTCGTTTAGTTTCCTTTTTCTGATTTTCCGATAACCCTTGTGAGGTTAAACACTTGC

CATAATTCAAAATCTTACCTTTAGGTTCTAGTTCTTGTTCTTGCTCTGGTTCTGGATTGT

TGAAACAGAGAACGTAGGAATGAGACCCTATATT

>LsbHLHD20

CTCTAATTAATATTTCATATACATACATAAATTTGATCTCTGCGAGTCTTAACACAACAA

TAAAATTACTGTTTTTTGACTTGCGGAGATCAAATGATCAAATCCTGAAAATAATTTTAC

CGTTTTTGGATGATAAGATTGCGGAGCAATACATCAATCATTTTTAGAATCCACTAATGA

AAACTTCATACAAATACATAAAAATATAAGTATAATAAATTTAACCTAATTAAATAATTA

CTCACTAATTACCTCATGATGGTTAAAGCTTGATGAGCCATCACAAGCATGGACATGTGA

ATGTGAATGTGAATGTGGATGAGGATGTGGATGTGAATGTTCTCCGGCGTTAAACTGAAT

TCCGGCTGGCAAGTCGATCGATGCAGCCGCTGTGGAAGCGAGTAGTAGATTTGGTGCAAA

AGGCCAGACGATGTCACAAGAAGGAGATAACAACAAAGCATTAGGAGGAGGAGGAGTAGA

ATTAATGCATTGTGATGAAAGTGGTAATTGATATTGTTGATTTTGAGGAGTAGATTGAAG

CAACCTAATTTGTTTCTTCAAGAACTTAACATAACGAATGGCTTCATCAAGCATAGAAGC

TGTATCCATTTTAGTTCCACCAGGAACAAGTCTTTGAAGGATACGAATCTTCTCACTGAT

TCTTTCTCTTCTATGTCTAGCTGCGACACTCTGTGGATCGTCGCTGATTCGAACGTTTCT

TCGCTTTGGTTTACGAATTGTTGCCGGATCGATTTCCACCGGTTGCATAGCAGCAATCTT

GTACATCATTTCCTTCATAGCTCCTAACTCTTCTTCTTGTTCTTCAACCTCTTCGATTTG

GTGGATCCCTAATATGTCACTGAGTGAACCTGATGAAGATGGTGGCGGTGGCGGTGCTGC

TGCTGGCATCATCATGTTGTTATAGCTATTTGTTGGCCAAATTCCAGAAGAATTACTCAT

AGGAATTTGATGATGATCTTGTTGGAGAATAATACGGTTTTGTTCATTTGTTTCCATTTT

CTCAAGATTCCAAGTGCTTGTGAATGCACTACTAGAATTATTTAGGTTATTTTCCATGAA

CATAAACAATTATTTATACATATGAAAAAATAATATGAAGTGCTAGATTCTAATTTTGAT

GGATCTAGCTAGCTAGATGGTGATGTGTGTCTTTATTGATTTGATACTTTAATTAGCTAA

A

>LsbHLHD21

CTTAGATATTCAAATTTTAGAATGGTTTAAAAGGGAATGATTTTTATTACAGACTTGTTT

AAATATGGTTGAGATTCCCTTATACATAAAATACTATTATAAGGAAAAAGTTAATAAAAG

AAGTAAATACTACAAGTTGGATGAAGAATAAAAATCAAATATAAATGATAGGAAATAAAT

CATCTATCATCTCTAAATAATCTTGATTATATCTTGCAACCAATATTTTGCTTGATCAAC

ATCAAATGGTTCCCCACCCAAATTCTGTAAAATAACAAACAGAAAAATGAATCAAAGTTA

ATAATAATGATAGCTTTTTTCAAAAGTTATTGTAGTTAATCAACATTTTTATTTAGATTC

TTACCTTTAAGCATGTTGTAATCATGTATGCTCTTTTATAGTTGTAACACTAATGTCTGT

CATTTCAATGCATAAATCATTCATAACCTCCATTAGTTTCTTGAACCTCCCTCTCTTTTT

CTCAATGATCATCTTAATCCAAAGATTAGTCCCATCAGTTTTTTCCACATGAATCTCTTC

CTATACAAAAAACATGTTACATGATTAAATAATATTTCATATATTTAATAAAATTAAAAT

TAAAATTAATGTGTACAAAACCTGTATTCCCCATTTCTTCATCTCTTCCGCATCAGAAAC

TTTGTTTATTTTCCGCTCTAAGTTTTCTTCTGCTTCCATTGCTTGAAGCTCTTGTGTGAG

TTTATCAACATCATCTTGAAGCTTCTTGACATAAGTAATAGCATCCTCAATAATGCTTTC

TTTCAGCATCTGAAAAAATCATGTAAATTTTTTTCATAATATTGTAAACATTAAAATCAT

TAGATATAACAAAAAAAAAAGTGAGAGTGAAGTTTAGAAAGTTTACATTTGTGATGTTTG

GTACTATAGAACGAAGTGTGCAAAGCCTTGAATGAAGTTTATCTCTCCTCCTCCTTTCAA

TCTCAAGATTCTTTGATACAAACACTCTTGTGCCATCTTCATTATAGTATTTCTTTTTCG

GTTTTCCCTTATTGATGTCTTCTTCAGCATTGAAAAACAACTCATCTTGAGACACATATA

ATATGTTACTATCATTAACTTGTTCCATTTTGGAGAAACAAGTAATGAACAAAGATGTTT

TAAAAAGTGAGAGAGTGAAAGAGAAAGAAAGTACAATATAATATGTAAA

>LsbHLHD22

TAAATACATGACAAAAGTAGTGATGTAACCAAAAAAATTAAAAAAATATAGAGAGAAAAT

GTTTAGTGGAAGGTAGAGATTGTCCTTTACCTGAGCAATGATAGTAATGTCAAGGATAGA

ATCACCAAAGGGTAACACACTCGTATTCATAACAAATAATTCTAAACTTTGAATCTCTCC

CATCATTTTTACCAAAACTCCCTTCTTCTTTTTGCAATGAATCCGAATTAGCATCTCTTT

CTCTAACGCTCTTGCTTCCACTTGTACGAGTGTTTCACTCTCGCCACCATCAGCACCTTC

GATGTTCTCATCGCATGATGAGGAATCTTCATCGCTACATAGGTCTGGTTTATTCAGAAC

AACCACCGAGTCTGCTTTTGTTTTCTTGTTATGTTTTTCCAAAATTTCCAAACGCTCTTT

AAGCTCTTTCACGTATTTAATAGACTCGGCTAATACAGAAGCTTTGTCCATCTAACACAT

AATAAATTAGTGAGTAGTAGTGTGTGTGAAAGATAATACAAAAGGGTCATTGAAATTGAT

TTACCGTACCTTTTTTAGGTTAGGAACAAGAGCTGCAAGAGCAATGAAACTCTGGCTGAG

TACCTCTCTTCGTTTTCTCTCGGCCATGATATGATCCTCAGCGTTAGCTCGAGATCTCTT

TGGTTGTGAGGTTTTTGTTTCCAAGTTTTGATTTTTTGATGACCCTTTTGGAGTTTGAGT

TGAAACATGAGTGTTTCCCAACTGGGGTAGTGATGATAGTGAAACTGACCGTGTTTCGTT

CGGTTTTGAGTTTAAGGTAGTACTAGTAGGGTCAAATTCATAAAAATGGGGGGTGTTGGT

AGCAGGTGAATTTGGGAGGTTTTCAAAAGAGAGAATCTGAGACTGAAAAGAAGAGATAGA

AGATGATGGAGATAGTTTCAGTGACATGGTTTCTGCTGTGATGTTTTCATTGTGATCCAT

GGTTGGTTTCTCAAAACCAAAGTCAAATGACTCAAAATCAGTTTCATCAGTGAATGAGTT

GTTGAGAGTGGACTCTGAATTCAAACATTTTTGTTGGTTTTGTTCTTGAAAGACAGTATC

TATGTCATGTGAAACAAACTCTAGTTTATCATCAACATCTAGGAAATTCATGTTGCATTC

TTCCGGAAAAAAATTGTACTCGTCCATTTCCTGAAAA

>LsbHLHD23

CTAATTTGGGTGGCTGCCAAAATAGAATTCACTTTTTGAATTGAATTGTGGTTTGTATAA

TCTTTTGGAAGGTGACAAATTAAGCACACCAAGCCACCTAACAATTGTCTAAACCTACTT

ATCATAGTAATTAGTGAAATATCAAACTATAGACTTATCAAGCAGCCACACTTTTTTTTA

AGAGAAATATACAATTATGAATATTAGAGTATTCTGCTAATAACGTCCAACCTAATAATA

TAATCTATTAATATGAAAATAATAAATATTTATAATATTATTAGTACCTGAGTAATCCTT

AGCCTGAATGTTATTTGATGAATTGTGGAATATTTGATTTGAATTTGTTGCATCCATTGA

AATCAAATTGACATGATGAACTTGCTTTAAGAATTCTAATAATCGAATCAACATATCAAC

TTGAGAAACTTGTCCGATCACGTTCACTTGTAAATCCACTACCATTGGTTCATCGTCCGA

TGAACTTGATTCCGATACATGCAAAACTCGCACGTTAATTCTCTCATTCGATGAGAATTT

CATGGTTTCTTTATTCGAAGCAGTCAACTTTTCTGACATCAACTCTTGGTTTCTTTTGCT

TAGTTTCTCTATTTCTTCCATCAATGATCTTAAGGTCTCTTTTGCTGTTATCAGTATGGA

TGCTTTGTCTTTCTTAGTTCCTTGAGGAAGTAATGCCCTAAGTGCTTGGAAGTTATCGTT

GAGCTTTTCGCGCCTTCGCCGCTCCGATATCATATGATGAAGTTGGTTACTAGAAGGACG

CATGGCTTGATTTCGTTCTCTCAGTCTCATCAAATTCAAGCTTCTAAAGAATGCAAACGA

TCTCTTCATCAAACTCTGTCGTCTAAAATTCGACCCTACGATAATAGGACTTTTATCGTT

TCTATACCTTCCAAATGCACTAGCTCCGGGATGCGCCGCGTAACTTTGCTGAGAAGGGGG

TGTAGATAGAACATTCTGGATTGCTCTCATTATAGCATCATTGTGATCAATAGGGAATAA

CTGTGTTGGTGTGATTTGAGGTAGTTGTTGAAGATGAAAGGGTAGTGTGTTCGAAACAGG

TCTCATTGGAGGAATATTTACTCCTCCAAGGATATGATCAGGAAAGTGATGTGAGATGGA

GAAGTTCCGGCTGGATTTGTGT

>LsbHLHD24

TCTTGAAGAGAGATTCAGTGGAAGTACCTTCAACAGCAGTTTCAGTTTCAGTACTCTCAT

CAGATGATGAGCAATGCTTGTTGTTATAGACCTCTGGTTTTATTGGACTCGGAGTGACCG

TAGACTGATCTGCCTTTGGTTTCTTCCCCTTCGTTTCTAAAATCTCCAAACGCTCCTTAA

GCTCTTTGACGTATTTGATCGTCTCTGCTAATATCGAAGCCTTGTCCATCTAAGAATTTA

CACATGCTTTGTAATTAGTATCGGGAAAATCTCAAATTTTACATTAGTCGGAGATAGTGT

ATTGAAAGAGTTTATATAGAATGTCATCCTTCACCTTACAATCATTACAGACACATAGAA

TGCTGCTAAATAGATAAAAACAACAATGAGATCACATACCTTCTTTAAGTCCGGAATGAG

AGCAGCAAGAGCAATGAAGCTTTGGCTGAGCTTCTCTCTTCTCTTTCTCTCCGCCATAAT

GTGATCCTTTATGTCAGCTGGAGACCTCTTAGCTCGTGATGTTTTTGAAGACCCTTTGGA

ATTTTGAGTTAAAAAATTTGTGTTTCCCAGTGACTTCGTCACTGTTTCTGTCTGTTTTAG

ATCATACCTATAAAATTCAGTTGGGTTTGGATTGTCAAAAGACAAGATCGGAGGCAAATG

ATAAGAGGATAGTGAAGTAGCACTGTGATCAATTGTTTTCATCTGTTTAGTTGGTTTCTC

AAAATCAAAATCAAAGTTATCAAAAGACTCAAAACTCGTTTCATCTGTAAATGTTTCGCT

CAGAGTTGTGGAAATGCATTCTGTATTTAAACATTGTTGCAGGCTCTCTCTATGCTCCTC

AAAGGCAAAGACACTAGCTATATCATGCGATAGAAACTCTTCTTCTTCATCAAGATCAAG

CAAGTTCGAGTTCGAGTTCAAGTTGCGGTCCTTATCATATAGTATGTATTCATCCACCAT

TTCCTGCAAAAAGTAAACACTAGTTGAATAAAGAAAAAAAAGGAAGGAGAATTTGCTTTG

AACTCCTTAGTACTACCAAATCAGATAACCAACTGCTGTCTGCTGATATGTTTATTGGTG

TCTTCATTTCCTCCATTGTTAAACTTAGGTTTAACTCAGAAAGCAACAAAGAAGATGGAG

ACTTTTC

>LsbHLHD26

ACGAATCTCTTCACTTGAATTGCAGATCAGTTTTTGTTGTCTTCAACTTGGCCTCTAGTG

TATGCAAACGTGCTTGTAGTTTTCATATACATTTTGCATCTCCCCAAAACAGAACCTTAT

CCATTTCACATGTTATAAAATTTATAAATATAAATAAAATAATTCAATACATATATATAT

ATATAATATTATATATATATATATATATATAATATATATATATATATATATATATATATA

TATGTATATTATAGTCTGGTTCAACTTAATTAGGTTAAGTATTATTTGTGTTTATTCACT

AATTAGGTTAAACTAGGAAATCCAAATTTAATCTTTCTTTCTCAATAAAAAAAATATAAA

AAAAAACTCATTTTGGATCTCAGAGATTACTTAAGATCATACCTTCTTGAGGCCAGGAAG

GATAGCAGAAAGAGCTATGAAACTTCTGGTGAGCTTTTCCCTTCGTTTTCTTTCAGCCAT

TACATGTTCCTTTGCTTGAGCCGTGTTCCTCGTCTGATTCGCTCGGATATCATAATTTGC

GGCAAAATCTTTGTTTTCATTGCACCCTTTCTCCAGTTTAGGCTTTACATCAGAATAATC

GAGGTTGTAAAGTTCATTAGAAGCAACCGGCGTTGCATTGAAGTGCTCAAAAGAAATGAG

TTGAGGTGATGAATAGGAAGAAGGGTTTATAGATGTTTTGATCTTCTTGGTTGGTCTTGT

TTGAGCAACGTAGTGATTATCAGGTTGAGTTTCAAGTGGTAAACAGTTTGGTGTAGTTTC

ATAGTTGTTGTCATAGTTGAAGTCAAAGTTGTCAAATGGAGATGGAAGAGAATCCATAGG

GTATTGATGAAAGAAACTGAGATCCTCAAATCCCTATTAAAAAATAATCAATATAAGAGT

AAGTTTTCTTCCTATATATCTATATATGCAAGAAATAAAAATTGTCTCAACCCTAAGTTT

ATATGTTTTAAGAAAAGTGATCTTTGATTGATAGATAAGATTAGTAGGTATGCTTACAAG

TTCAGGCATATAGATGGGGGGCACAACTTTCATCATCTTCACAGTTTTGTTTCTAAAAAT

TTAATTTGATTTGGTATAGAGTATGCCCTGAAATAGTGGGAAAAAAAACTTGAAATCT

>LsbHLHD27

TTACCCTTAAAATAAACACATATTTATATAAAAAATTTAAAAATATCAAATAGTTAATAA

TCACATAAATCTATAAATTCTAATTTAACTAACAAATATGGATATTAGTTTGATCCTAAA

TACTAGAATTCAATTCCTATTATTGTGGTTATATGTGTAAATTTTTTTTTTATGGTAAAT

ATTCTAAATAGGTTAAAGCACTAAACCTTGAAATAAAGAGTACTATATTTAATAATTAAA

TGATCCCTCACACGTAGATAGTTAAGCTTATAAATACTAATTAAACCTTAATCAACAAAA

TTAATCACCATAATGATCAACATTACGAGCCTGATGAGGCTTAGGGAATGAAACAGGGAA

CCCTATCCCAGAACCAGCAACTGTCCTGTTATTGTTGGCATGTTCCTGCAATGTCTTCAA

CTGTGTCTTGAGAAATTTAACATAATGAATAGCTTCATCCAACATTGATGCAGTATCCAT

CTTCGTTCCGCCAGGTACCATTCTCTGGAGTATTCTTATTTTCTCGCTTATTCTTTCTCT

TCGGTGTCTCGCTGCTATACTCTGAGGATCCTTTGAGATCTTCACGTTCCTTCTCTTTGG

CGGTTTTACCGATTCCGGGTCTATGTAAATCGGTTGCATCGCTGCTATTCGGAATATCAT

CTCTCGCATCGCCGCCATCGAGGTTCTCTTCTCAGATGCACCGTAATGGTTCGGATTCGA

TACAGCCTGGTTGTTGTTCGAATTCGAAAACATTAACGGTGGAGCTTGTTGTTGTTGTTG

AAGGAATAATGGAGTAGCAGGGTTGTTGGTAGTGAAAGAGATATTGTTTGTTGATTGTAA

AGGTAGTGATGAAGATGATGACCATGGGAATGGATTCGGAGGGTTATCGATTAAATCTGA

AAGAGGACGTGTGTTATTGTTAGGATTAACGGAAGAACTATAGAAGTCTTGATCTGACGG

TGGAAATTCATTGGCGTTGTTGTTGTTGTTGGATGAAGAGAATTCAGGGAGTTGCATCAT

CATCATCAACATCATGTCAATTTGTTCCTCTGATGATGAGGAGGAAGATTTTAACTGGTT

ATTATTACAGTCCATTTGTGATGTGAAGGGAATAAAGATAGAAACA

>LsbHLHD28

ACAGATCGCTTCACTTGAATTGCAGATCCAGTTTTTTTGTTGTCTTCAACTTGTGCCTCT

AGAGTATGCAAACGTGCTTGTAGTTTTTTCATATACATTATTGCATCTCCCAACACAGAA

GCCTTATCCATCTACACATGTTATAAATTCATAAATATAAATAAAATAATTCAAAACATA

TATGTATATTATAGTCTGGTTTAACTTAATTAGGTTAAGTATTATTTGTGTTTATTCACT

AATTAGGTTAAACTAGGATATCCAAATTTACTCTCTCAATAAAAAATAAAAATAAAACAA

ACCCAATTTGGATCTCAGAGATTACTTAAGACCATACCTTCTTGAGGCCAGGAAGGATGG

CAGAAAGAGCAATGAAACTTCTGGTGAGCTTTTCTCTTCGTTTTCTTTCAGCCATTACAT

GTTCCTTTGCTTGAGTAGTGTTCCTTGTCTTACTCGTTCCGTAATCATAATTTGCTGAAA

AATCTTTGTTTTCATTACACCCTTTCTCGAGTTTAGGCTTTACATCGGAATAATCATAGC

TGTAAAATTGCTGAGAAGAAACTGGTGTTGCATTGAAGTGCTCAAAAGAAATGAGTTGAG

GTGATGAATACGAAGAAGGTTTAGGGTTTGAGGCTATCAAGTGATCTTTGGTTTGTTTAG

TGTCAATTAATGTCTTGATCTTCTTGGTTGGTCTATTTTGAGCAACGTAGTGATCATCAG

GTTGAGTTTCGAGTGGTAAAGAATTTGGTGTAGTTTCGTAGTTGTTGTCAAATGGAGATG

GAAGAAAATCCATAGGGTATTGAAGAAAGAAACTGAGATCCTCAAATTCCTATTCAAAAG

TAATCAATATAAGAGTAAGTTTTTTTCATATATCTATCTATATGCAAAAAAAAAAAAAGA

ATTTAAAGATAGACTAATTCGATAGATAGATAACATGTATGCTTACATGTTCAGGCACAT

AGATGGGGGACACAATTTGCATCATCTTCAGTTTCCTTCCAAAAAATTTGATTTATTTTG

ATATAGTGGTATGGTATGGGACATGCACTGAAATAGAGTGAAAAACAGAAGAAAAATAAA

AATTGAGATCTTAGAATTGAAGAAAAATAAAGTCATGGAATGGAAGAAAAGAAGATTGAG

AAAATAGGGTAC

>LsbHLHD29

AATAATTAAAAGACACAACTAAACAATTATAATCATTACTAAATTAAAAAGGGAGAATCA

ACCTCATTTCATACCAATTTTATTTGTTATAACAAAATTCATTTTGAAATATTAGAAAAA

AAGAAAAAAAATCATTTTGAAATAGTAATTGAGTAGAATACTTAACAATTGACATGTCTT

TGTTCTTTCTTTCTATGCAGTGCTTCATCATCCATTAGAAGACTTAACACAAGAAGACAC

ACACTACTCTCAAACAGTAACCACCGTTCTCCAAAACCAATGGATCGATTCACCTTCCAT

CAACTACATCAACTACTCCACCCAATCCTCTTTCACCACCTGGACCAACCACCACTTCCA

CCCGCCACCGCCGCCGGACACCGCCACCTCCCAATGGCTCCTCAAATACATCCTCTTCAC

AGTCCCATACCTCCACACCAAAAACCACGACGAAACCTCCCCACAAACACACGACACCGC

CGGAGTCAACAGCAACGATCCCTCCGCCAGGCTACGCGGCAAGGGAACTCCCCAAGACGA

ACTCAGCGCGAACCATGTCCTCGCTGAGCGACGGAGGAGAGAGAAACTCAACGAGAGGTT

CATAATTCTAAGATCATTGGTTCCTTTTGTTACGAAGATGGATAAAGCTTCTATCTTAGG

CGACACGATTGAGTATCTGAAACAGCTTCGGAGAAAGATTCAAGATCTTGAGACACGTAA

CCGTCAGATGGAGTCCGAGAAAAGTGGAGTACCCGTTTTGGTGGGTCCGACTGAGAAGAA

AAAAGTGAGGATTGTGGAAGGGAATGGCGGTGGTGGGGGCGTTAGAGCGAAAGCAGTCGA

GGTTGTTGAGAAAGAGATTGTTGCGTCGGTTCAGGTTTCGATTATAGAAAGTGATGCTTT

GTTGGAGATTGAATGTTTACAAAGAGAAGGGTTGTTGTTGGATGTTATGATGATGTTGAG

AGAGTTGAGAATTGAGGTTATCGGAGTTCAATCTTCGCTTAACAATGGCGTTTTCGTCGC

GGAATTGAGGGCTAAGGTTAAGGAAAATGGTAATGGGAAGAAAGTTAGCATTGTGGAAGT

TAAGAGAGCACTTAACCAAATTATACCTCATAATAATATTTAGTGTATGTTTGAATTCAC

AACGAGTTCGACAAAATCGCGATGATTGTTGTGATTCTGTCAAACTCGTTGTTAATCCAA

ACATAGTCTAATGAT

>LsbHLHD30

TGAGTTCTCATACTCAAACATGGGATGGCTCTTACAAGAGTTAGACCCTCCACAGTCCTT

AAATATTAGCCATAAGGAGAAGAACTATGCAACAAATTCAGAGTACTCTTTACCTCAATA

CCATCAATTCTCTTCAGTGAAGCAACAGCATGTTGAGATTGAAACGCCACCACCATCCCC

TAAGCTTATGGTGAAGAAGCTTAACCACAATGCTAGCGAACGTGATCGCCGTAAAAAGGT

CAATACCTTGATTTCTTCACTTCGTTCACTTCTTCCAGGCGATGATCAAACGGTAATAAT

AACTATTAATCTCTCTCCTTAATTACTGATTTATTTTAATTAAAATTAAAATGATATTTT

AGTTCTAACTATATATGTCACCATATAAAATGAAAGTGACGTGTATCAAATATGAACGGA

ATCATATCAATATAAAATTGAAATTTTGCACATAAAATATTGTGATTAACAAATACTTTA

TTCCATTGTGATGTTGCAGAAAAAGATGAGCATTCCGGTAACAATTTCAAGAGTGTTAAA

ATACATACCGGAATTACAAAAGCAAGTTGAAGGATTAACTAAGAAAAAAGAAGATCTTCT

ATCAAGAATTTCTCAGCAACAATATGCAGTCAACAAAGAATCACAAAGAAAAATAATTCC

TAATTACAATTCTTCTTTTGTAGTTTCAACAAGTAGGCTTAATGATAGTGAGCTTGTTGT

TCATATCTCATCTTATGATGCTTACAAGATTCCATTATCTGAGATCTTGATGTGTTTGGA

AAATAATGGACTTGTTCTACTAAATTCTTCTTGTTCTAAAACCTTTGGAGGGAGGCTCTT

CTATAACTTGCATTTTCAGGTATGTTTTGTAATGTTATGTTGTTAATGTTTTTAGTTTTA

GGTATTTATTTATTTTTTAATTGTTTTTATGCAATTTTTGTAGGTGGATGAAACTCAAAG

ATTAGAGTGTGATGATCTTATTCAGAAGCTTTTGTTAGTATATGAGAGGCAGCGAAGTAA

TCAAGTAGTGTTGGGAGCTAAAGATCATATGATCAGGAGTGTTATGATATATTAGTTAAA

GTTGGGGATTGAGTCTTAGTTGTAGTTTGTACATATTTTGATAGCAAGGATTGAAAGCAT

GGAAAGGAGTAATCACTTTTGAAATTTAATTCAATTTTGTGAGTATATATAAGTTAGTTG

AGTTAGTGAAGTTACTTTGATGTGTAAGTAACTGGTTAATTAAGTTGGTTATTATGATAT

TTTGAGCCTCATTGGTGTGTTGTAAGCATGATCCATGAGGTGTATAAGTAATCACTTCAG

TGATTATGAATGAGTTGTGAAGTGCATAACTAATTAAAATAATTGTTCTCTCTTCCTTTC

TTGTGTATGTGTTCAATTGGTTCTAACAAGTGGTACTTAGAGTTTCAAGTTCATGAGTTT

GAAACCACATGACAAGTCATAGAGAGTGGCTTTTGAACTTTTATGGCTTAAGAATCCTAA

CAAATT

>LsbHLHD31

TAATGATAGTAGAGTAGAGGTGATTGGAGTTCCGAAGATTTTCGGGCAGGACTTGAACTC

GGCGACACATTTTCGGGAGAAACTTGCTGTTAGGAAAATGGATGAGAGGCCGTCATGGGG

AGTCCGTCCGAATGGGAATGGAATTAGTTTTCCGAACGGTGTTCATGGTTCGGGTTGGAG

AGGAGGTAGTCAAGTTGTGAGGCAGCATGTGCCTGCCGATGTTTTTACTCCTAGGCCTTC

CGCGAGTAATGCGCCTGAGCTAGCCAATGGGGGCGGGAGGCATGATTTTGTGCTAAACAA

CTACCAACAGCAACGGAAGGCACAGATGCAGATTGATTTTTCCGGAGCCACATCAAGACC

TTCTGTGAGATCGATTGTCGGTGAATCGGAGATTTCTGACGTTGAGGCCTCTTGCAGGGA

TGATGCACCGAGTCCCTCCGACGATAGGAGGCCGAGGAAGCGGGGTAGAAAGCCAGCTCA

CGGAAGAGTAGAACCTCTTAATCATGTGGAGGCAGAGAGACAAAGACGCGAGAAGCTAAA

CCAGCGTTTTTACGCCTTACGAGCTGTTGTCCCAAATATCTCCAAGATGGACAAAGCATC

TTTATTGGGAGACGCTATCGCTTACATCAACGAACTTCAAGCAAAACTCAAGTCAATGGA

ATCCGAAAGAGAACAACAACTTGCAACCACTTCTAGAGACGGATCATCATCGGCATTACA

AACAAACTCTAGACCGGAAAATCATCAAAACAAAACTCCGGAAATCAACATTCAAGCTAC

ACAAGAAGGCGTCATCGTAAAGGTGAGTTTCCCTATTGATGTTCACCCTATTTCAAAACT

CATCCAAGCATTCAAAGACACCGAAATCACTATCCTCGAGTCAAAGCTCAACGCCACAAA

CGACACTGTCTTCCACACATTCATAATCAAGTCCCAAACCTCCGAGCAGCTTACAAAGGA

GAAGTTAATCGCAGCATTTTCCAAAGAATCAAACTCCTTATAGCCATAGTCCATCCATAC

TCATGATACATAGAGAAGAACCATTTTGCCCATACAAAAGAGGATAAATCTAAATATAAA

ATGCAGTGTGATCTAGTGAAGTAGAATAATAGGCAATATCTCCTATTAACCGTGTAGCAT

AGCAAATTTTATATTTGT

>LsbHLHD32

AACAAAATCGATTTTGTATGGTCCTTGAGAGGTCATCTCTATGTTTTCATCTTCAAGATC

ATCGATTCTTCTGAGTGGAATTATTGGTCTCAATACACTGATCGTTCACACTTTCCTGCT

CAAATTCAACCTCAAAATATCTCTGATTCTTCTTCTGCTTTTTTTTCAATGTAGTGAGTT

TCAGTCTTGGCCTTTACCTGTTGAAGGAAGTTTAGAAGATAGAGCTGCACGTGCTTCCAA

GAGTCATAGTCAAGCAGAGAAAAGACGCAGAGATAGAACCAATACTCAGCTTGCTAATCT

CAGAAAACTTATTCCTAAGTATGATAAGGTAGAACAATTTGTTTGTGTTATTTGCCATTT

TAAGGTTAGATTTTATAATGTTTTTCATGCAAGAAAATGGAATTAGCCACTTAGGGAAAA

GTTATGTGATTATGATGTTTTGCATTCAACTCATGAATTTCTAATTCCAACCCTAACTGA

GATTTAACACAATCCTCGCTCTCAACTGAATTGAACTTGGATTAATTAACAGAGCCTAAA

CTCTGGCGCGACGGTGTTGGTTAGAGACTACAGCGAGGCAATCAGAGAGAAGAAAGTATA

TTGAACGTATTTCTGAACATGTGAGTTTATGAATGTTACCTGAGTTTGCTGAACTCATGT

TTGTTGCTGTTAAAAACTTATCACCATGCTAAATGTTTGGTTGTTAAATGGAAGACTGCC

ATTAGGGCAGTATGTGCATGTTATTGCTTTTTTTTGTAAAAATGCTGATATATGCCTTTG

AAACATAGGGTGAGTTGAGGATTAACATTGTTATGTTGAACTCATGTTTGTTGCTGTCAA

AACATGAATTTCTAATTCCAATCCTAACTGATATTTAACACAATCCTCGCTCTCAACTGA

ATTGAACTTGGATTAATTAACAAAGCCTAAACTCCGGCGCGACGGTGTTGGTTAGAGACT

ACGGCGAGGCAATTGGGGAGAAAAAAGTATATTGAACGTATTTCTGAACATGTGAGTTTA

TGGATGTTACCTGAGTTTGCTGAACTCATGTTTGTTGTTGTTAAAAACTCATCACCATGC

TAAATGTTTGGTTGTTAAATGGAAGACTGGCATTAGGGAAGTATGTGCATGTTATTGCTT

TTTTTGTAAAATTGC

>LsbHLHR1

ATAAAATTAATCTTTATAAGTTGTACACCCCAAGATAAGGCAGTATATTTATTCTCATAA

TAAATTTTGTTGGAGCCAATATACATTTTAGTTTCTCACAAAGAATTAAATACAGATTAT

ATATTTTTGTTGCTGCTAATTAACACCAACAAAACACTAACTTGCAGCTCAAGTCTTTGA

TCCTGGTGCTGGAGGTTGTTGCTGCATCTGCTGATACATGGCAGCAATCCTACTATATGC

CTCCATTGTCATGGGCTGTGATTGGCATCCAAAAAATGTTGACATAGGATCAGCCATCCC

CGTTGCTGGAGGGCCTTGGAGTCGGTCGGTGGCGGTGGCAGCAGCACCAGCATCCCATGC

AGGCATAGGCATGAATGCTGAAGGGTGGAGAACTTGAGGCATTCCAGGGATGTTAGCTCG

ATTCATAGGGTTCATATCCATTCCCATGCCCATTCCCATGCCCATCCCCATCCCAGCCAT

GCCCATCGGCCCCATTCCCATGCCCATCTGATTCATCATGGACATTTGAAGTTGTTGCTG

CATGGTCATCGGCATCATCATAGATGACATGTTGAATCTATTCACCATGTTCACTTGTGC

TTGCAATTGTTTTAGATATTCTATCACCTCATCCAACATTGAAGCTTTATCAGTCTTGCT

GGAATTTGGGACCAACTTTTGCAATGTCTTCATTCTCTGGTTTATCTTATCCCTTCTTTT

CCTTTCAGATTGGTTGTGTATAGCTGCGGCTCTACTTCTTTTAGTGGACACAGATGATTT

TCGATTTTCTCTCTTTTTATCATCTTCTTCGGCATCATCCCTCGTTGGTCTACTGTGGCA

AACAGAATCATGATCATCTACGGTTGTGGTTCTGGTGCACTGCTTATCAGAGCTGGTGTT

TTCCATGGATGCTATTGAAGTTGAAGTGAAACCAGTCATGCCGAATTCCCTGTCATATGT

ATCTAACGTCACTTGTTGGCTTTGCCTCCCCAACGTTGCGCTGTTACTCATACTCTGATC

CCTTCCGCTCCCCGCCACGTGTGTCACTCTTCCGCGTTTTGCTCCTGTAAAGATCCCGCC

TTCCTCCTGTGGTGTAGCCCCGACAGCGCTACAAGACCCCACGGGCGTGGGGCCACCAAC

CATGCACGATCCAATACCTCTTATAGAAATTGGATCTCGTGCATGGAGTCTCTGCTCCTT

CTTAAGATTGGAGCAGGGAACCAACGCGTCCACAACAATACCATTGCTTGTGGCTGTTGC

AGCAGCAATGGCTGCCGCACGTTGAGGGTCAAGCCACGGGACTAACATGTTCCCGTAGAC

ACCGCCACCCACGGAGAGAAACGGTGCTTTACCGTGATGTGGAAAGCTAGTAGCTTGGTT

CACTATGGACTCCAAGGTGCCGCTTCCACGAGGCTTTTCCCATGTTTCCTTCGAAGGCGC

GGTTGTTAAAGGTTTAACCGGCACTCTTGGTAACCCTAAGCCATGCATAGAAAGTTGTCC

ATTTTCCCATGTCAGTTCTGCAACATCATAGTCCAACATGGGAACATCATGAGGGGCAGT

TGAGTTCGAGTTGGAGCGAAGAGAGGCCGATCTTGCATGGGATTGTGGATTTTCGTCAAC

TTCCCAACTAGGAACGCACTGACTCATTTTGAGCAAGAATCAAACCAGTTCTCCTTAACT

GTGTGTTTTGCTCTTACTCACATGTTGGGAAAGAAATATTGATGGAAGAAAGAGAGTGGA

AAATATTAATTGATGATTATATGATACGATTTTATCTAGTTGTAAGGGCCCATGTATTAC

AGCAATGAGTGAAAGTAGCTTGTGGAGTTCATTTGGCTACGTAAAGGGGAGAATATAGTC

CATATGTATTGGAGTGACAACATTTATGTGTGGACTCAGTTCTATTTTGTCCTTTCAAAC

TTTTTTTATTAATAGAGTAATGATTAGATTTCATTTG

>LsbHLHR2

CCCAAAAACCGGCTATAAGTATTAATGACGAGAGAAGACAACAAGACATGCTTATAAGAT

TTACATGCAAATACCAGCATTACTTCATAATAGTATTAGACCTCACAATAGCAAATACCA

GTTAGAATACATAGCAAATAACTAAAAGGAAAGGGCGTATGGGGAAGTAATACAACTTTT

AGGCCATAAATGGAATCCCAAAAAATTGTACAGTGCCAACTTAGGCAACTGGTGGACGGA

GCACATGATCCTGTGAGGTATCCACGGAGGCTGGTGGCATGAATTGCCACATGGCAACTC

CTGGATAACTCATGAAAGGCATTAGCTTGTTGCTATGGGCTTGGCCTTGTGGAGCAAATG

CAGCAGGGATTGCAGGAGGGTGTGTCAAGAAACTGGGTTGGGCATTCATTGATTTCACCT

GCTGCTCCAGCTTCTCTTTCTCAGCCTTAAGCCTCTGCTTCTCATCACGGAGTTCATTCT

TCTCAACCTTTAACTCTTTAATCTTTTCTTGAAGACTTGAATTGGAGTCTTTCAACTTCT

GGGCTTCACCACGTAACTGTGATACCATTCGGACAGCATCAGTCAGAATCGCTGCCTTGT

CTGTTTTTGGTGGCCTTCCAGGCTCCAAGATAGAGCCTAATTCAACAAACTTATCGTTCA

GCCTATCCCTCCGCAGCTTCTCTCGACATGCCTTAGAGCTGGAAGGAGCAGACGAATCTG

ATCTACCTCTCTTCTTTGAACCAGGATCTTTGATACCATCAGAATCTCCCAGAGAACCAT

CCATATCAACACAGACATTGAGAGGTTGTGATGGTGGCCACGTGAAGGCGAAGGAGCCAT

CGGAGACGGGAATATCGTCAATCAAAGCATAATCGAAAAGCCAATTGGTGTTTTCCGGGG

AAACCATAGTTCGGAGAGATTTTCCCGGCGGTGATTATTTTGTTTTTAATTTTTTAAATA

AGTGATTATTGTTTTATTTTGTGAGACAGAGAGAGAGTGATGATTGAAGAAACGCTCTGT

TGAAAAATGAAAAGCACGTTTAGGGGTTGATAAAAAAAGTGATTTTTTTTAAATGATTTC

TTTTTATGGTAATAAATAAATTGGGGAATGGAATCTAATGCTTATGACTGTTTTGGGGAG

ACGA

>LsbHLHR3

CCTTTTATGAAATTGAAAAGAAGACAAAGCCTCCCTCACTCACCCCATTCATTATTTTAG

GAAACTCACCACTCTCTTTTCCTTCATGTAGATAGATTCTCCCTTTCCGCGTCTTCAACT

CGTTTTTCCAACTCACTGGTGACTCGTCCGAGTCCAACTGCCTCATGGCCAATAACCCTT

CCGACGCACCCGCCGCTGATGACTTCCTCGAACAAATCCTCAGCCTTCAGACCTTCGCTT

CCCCCGACTCTGGATTGACCGGACCCGACATCTCTCTGACGGGAACGTCGCCGATGATGC

TGCAGCTCAACTCCTCCGACGCCAACCATCACCTCGCTGCCGGAGGTTCCTTTCATGCGC

CGGTGTACCAGTTAGGGTTGAGCTTAGACCAAGGCAAAGGAGGGTTTTTGAAGCCGGAGG

AAGCCTCTGGTAGCGGGGAACGCTTCCGCAACGACGTGGTTCATGGTAGACCTAATAATG

TTTTTCATGGGCAACCCATGCATACAACTGTTCCTGCTGCTCCACATCCTCCAGCCATGC

GTCCTAGGGTGCGGGCTAGAAGAGGACAGGCTACGGATCCTCACAGCATAGCGGAGCGAT

TACGCAGGGAGAGAATAGCAGAAAGAATTAGGGCGTTACAAGATTTAGTTCCAAGTGTCA

ATAAGACAGATAGAGCTGCCATGTTAGATGAAATTGTGGATTATGTCAAGTTCTTAAGGC

TTCAAGTGAAGGTTTTGAGCATGAGTCGATTGGGTGGAGCAGGTGCAGTGGCCCCACTGG

TAACTGATATCCCATTATCATCAGTCGAGGAAGACGGCAGTGAGGGTGGAAGAAATCGAC

CGGCTTGGGACAAGTGGTCGAATGATGGCACAGAAAAACAGGTTGCTAAGCTTATGGAAG

AAAACGTTGGAGCTGCCATGCAATTGCTTCAATCAAAGGCGCTTTGCATCATGCCGATCT

CACTGGCATCTGCGATATACCAGTCACAGCCGCCGGACAGCTCTAGTATAGTCAAGCCTG

AAACTAATCCACCTTCACAGACCTAGGGGCACAATCCATGGACCCCATCAAAGGCTTACA

AAAGACTTCTGAAACCCACAAATCATATTGTCTGTTTCACACCCTAAAATTAAGTCATTG

GCAAGCATCATGTTGCAATCAAAGTCAGATACCATGACTCTTTCCTTTTTGCCTTTCTCA

TCTTTTGTCCATACAAAGTTTAATGTCAATGCCTTAGAAAGTAGAGGCAGACCCAAATTT

CAAGAGGAAGAAATTGTCAAAAGTTCACTTGATATGCTTTTCTCTTTTGCTAAAATGGGT

GGTGGGTAGTAGTGGAACATGATGAAAGCACTTTTATAGTGGGGCTTAATTTTTGTGTAG

TGGGCTGTGGGACCATTGTAAAAGGTTATATTATAGAATGGTGTGTATATTATTATATTT

TAGTAGTAGTAGTGATAAAAAATGCTGGGAGGTGCCATGTCCAGCAGAAAATAATTGTGC

TTTTTGGTTATTTAAGGATAGGGACAACTTTTTCTATTCTGCTGAATCATTATTGGATTT

TGATGTATTGGATGGAGATGGAAGGGAACAAGTGTGGGAGCACATATCTATATCAATCAT

TAAGCTGCAGCAACCTTATTCAACTTATTTCTAGAATATAAGTATTATACGGTGCATACG

GTAATGGGAAACACTAGAAGTAGGGACCAAACGTTTCACTTCCTTTGTAGGATGGGATCT

CACATTAACAAGTAACCCAATTGTTATGTTTCCTTTTGTTTGTGAATTTAAGTCAATTAT

GATAGGCAATTTCTTTTTTCACCTTGTGGTGTCTGAAAATAGAGGACTAGTTCTCATAAT

TTTGGATATGCATGTCTGGATGCATTTTGAATCATCTCCTCCTTTTAAATCAAGCAGGCA

CCTAATTTTTCTTAAAATTTAGTTTGAAGATATGTTTTTGAAAATTTCAG

>LsbHLHR4

ACTAACTTCTAATAAACCTAGAGTTGAATTAAGTAACAACAAGAGATAGTTACTAAAATT

ATGAAGAAAAAAAATCCCGGGATACATCGCATGACCAAACGGGTCCGCCAAAAGAAAATC

ACAACCTAACCACAGCCATGTATTATCTTGGGACAGATTCACAACCTATTAAGGAATAAA

CAGTTGTAAACTATTTTCACAATCTGAACATTTACATTTACACACATTTAGTGTAATCCA

TGGACACTTTAACCAGTGATCAAGCGTACCAAATTATTTTGTAAAAAGAAAAAGTGTCAA

TGAGGGAATACCAAAAACGAGAGAAAATTAAGTTACAATAATTTACATGTTATCTAGATT

AGCTTGCAACTGGAACTGATAAAAAGGTCCTTCCGTTACACTACAAAACTTGGCATACTG

TAGTTTGGGTGGCAGAAAATATAAAATAATACTAGTAGTACCATTTCCGTAAGCCCTTGA

TTGTAACTATCAAATAATCATTTATCTCCTGACTGGCGGATTTCTGACTTGCTGACATCT

GGTTGGTTCATTGCCTTATTCTGCATTAATTGAAACTGCATCTGTTGGGGTCCAAGGTAT

TGTTGATAAGGATCAGTGAAGTTTGGGATAGGTTGCTGATTTGCATCATGTGTTCCAACT

GAGGTGGGCATGTTACTATCTGCCAGATTCGCTGCTTGCATTGTGAATGAATCAGGTGTA

GGAACATGCGGCATGTGGAAAGGAGGCATAGTGAACCTTGGTCCGAAATTTGCAGGCAAA

GTAGAAGCAGGCAACATGTTGGGAAACGGCATTACTGGTCTGTTTATTCCCATTCCAATT

GGTGGGATATACTGCTGGATTCCAGGAAACATCATAGGTACCATGCCACATCCCATGGAC

ATCATCTGCATAAGTTGTACTCTCTGAACTTGTAACTGTAGTGACCTCAAGTACTCAATT

GCCTCATCCAGCATAGACGCTTTGTCAGACTTATTAGATTGAGGTATAAGTTCTTGCAGG

GCTTTCATCTTTTCATTAATCCGATCACGGCGCCTCCTCTCTGAGAGACTATGGACCTCG

GCAGCACGAGATCTCTTTGTTGATGATCCATTGGTTTTATTTTTTTCTTCCGCAGATTCA

AAATCAACATCCTCGCTATGATACTTCCATTCATCTGTATGCTTTCCCTTTCGCTTCCGG

TCCAGCTCCTCTACCTTCCGAACCGGTTCTCCACTGCTACTCGAACCGCCCTGTTCATCA

AACGTCGTCCATGGTGCCGATGCGCACGTCGCTCCTTCCGTTGGCTCCATCGAACTTCGT

ACTGTCTCTGAAATCGCCGCCGGCATCACAGCTGGAGTATCGCATGAATCTACAAGCGTG

GATTCTCTCGCAGAAGTTCTCAAAATTGACGACGGTTCAGGCTCGGCTCTAACCCGAGAC

GGTGGAGTTTGCATGGTACTATTGTTATTAACAGCCGACGGCTGGTTGAGGAAATCGGCA

CAAAAGGATTGATCCAAAGGAGAATCACCATCGGTGTTAGGATAATGAAGCCACGAAGCC

ATTTCGCCTTCTTGCATAAAGAGATATTCATTCTCCCTCGGCGATGAAGTTCCGCCGCGA

GTGGAGTCGTCGGAGTTTCTCCGCGGCGGTGGTTTATTGTTATACCGGTGATTTTGACTT

TGCGTGACAATTGGTCCGTTTTGCCATAATAGCTCCACGATCTCATCGTCTGCAATGAAG

GGTTTCTTAGGGCGAAGTAGCCCTGGTTGATCCATTTGCAAAAGACACCTCAATTTAAAG

CATGCAAAGATGAAAAGGGAAATTTCAAGCTAGTTCAGTTCAGTATGAGGTGTTATAAAT

TATAATGGAATTTTTGCGGTGTAAATTGAATAGTGTAATTGGAGTTGGATTTTGAGGTTG

CAGAATATAACGTGGTGTGTTGTGTGATAGTTGATGTTGTTTGGCCTTGGAAATAAGAGA

GATGTCACAACAGCAACAGAACAGAGCACGAGCGAGGCCACAGATACCCAGAGATAAGAA

GATTTTTCTTTTTGAATAATGGTAAGCTAAAGGACGAAACACTTTTTTTGACGCTTTGTG

TTTATTTTCTCCTCTCT

>LsbHLHR5

TTAGGTCTCTCTGTGACTCTATCTAAATATGCTGATGTCATGTCTTTATTGTTGTTAAAA

TAAAAACTCATGTTCACAGTTCCCAAAGCACAAGGACACACTCAAAGAGAACTAAAGACA

GGTGAAAAAGCAGGAACAAACAAGAAGGAGCTTCTGTCTCAACGTTCCACCTACTTTCTA

CTTCAAAAATCAGAAGAAAAAAAAAACTGTTTTCTTCTCACTTCAGGTAGTTGAATTGAT

TTAGTTTGGTTGGGTTTGATTCGGTTACGATGAACAACACTCTTCCTGATTGGAGTTTTG

GGAGTGATAATTTTGTGACCAATCAAAAGAAGCAATCTATGGGGCTTGATCATGAACTTG

TGGAGCTTCTATGGGAGAATGGTCAAGTAGTATTGCATAGCCAAACAAGTAAGAAACCAA

TAAATTCAAGAAATGTTCATAAAAATCTTCAATCAACAACATTAAGCAACTTGATTCAAG

ATGATGAAACTGTTTCATGGATCCAACAATACCCTTTTGAGGATCCGATTGGACAAGAAT

TGTGTTCGAATCTTTTATCCGAACTTCCACCTTGTCATGTCGACTCTTACGATTCACAGC

CGACTACGAAACCGTCTTTTGTCGAGGAGTTCTCAATTCCTAGGTTTCACCATCATGTTC

CTGATTTGTCACTGAAAAACAATGAATTGTGTGGATCAAATAAGGTCGTAAACTTTTCTC

ACTTTTCAAGACTTCCTAATGTTTCTTTAGCATGTAATAACAGCGATGGAGATTTAAGAG

ACAAAGTTACGGGCAATCTGTCACAATGTGATATTAGAGAGAGTTCCGCGATGACAGTTG

GTTTGAGTCATTGCGGTAGCAACCAAGTCCAACAAGATCCGGATGTAAGTAAGGTATCAA

GCGATGGCGTTTGGACGAATACCATATCGGCCGAGCCTCAGCAAGTCAAAGACAATGTTC

AGACAACAACAATTCCTTGGCATGAGAAAGGGAAATCGGAGATGTTGGAACCGACTTTTA

CTTCATCTTCCGGTGGTTCGGGAAGTAGTCTTGGAAAAACATGTTCCTTATCTACAAGAA

GTCATGGAGAAAAGAGAAAAGGGATAGATGTTGATGATTCGGTGGAACAAAGTGAGGACA

CCGAACTTAAATCAGCCTTGTCGAACAAGGTGTCGCAGAGGTCGGGGTCGGGTCGAAGGA

ATCGGGCTGCTGAAGTGCATAATCTTTCAGAAAGGAGACGAAGAGATAGGATTAATGAGA

AAATGAAAGCATTGCAACAACTCATACCTCACAGTAGTAAGACAGACAAAGCATCGATGT

TAGAAGAGGCAATTGAATATTTGAAATCACTTCAGTTACAACTTCAGGTAATGTGGATGG

GATCTGGCATGGCACCAATGATGCATCCAGGATTTCAGCACTACATGACACAAATGGGCA

TGTCAATGCCTACAGCTTCATTTCCTCCGCTTCAAAATCCGTTGCAATTACCGAGAATGC

CGTTTGATCCGTCCGTATCATTGTGTCAGACACCAAATCCAACATTAACATGTCAAAATC

CTCTTTTTGGTGCCTTTAATTACCAAAATCAGATGCAAAATCCAGCTCTTTCAGAGCAAT

ACGCTCGTTACATGAACTATCATCTTATGCAAAACGCTTCTCAGCCAATGAATATGTACC

AATACGGTCCCCAAACTGTACAAAATAGTCAAACAATGATTCCAACCAGCAATAACAGTG

GATCAATGAGTGGAGCAGCAAACATTAATGATAATGTGAATGGCAAAATTGGTTAATTAG

TTTAGAAGCATGCCTAGAGGGATCTATGGTGTAACTAACAACAAAGTTGCAAGATGAATG

AAGATATATAGCATGTTTTCATATTATGCTCAACTATAAATTATTATGTATATATAGTCC

ACTTGATTCATACAAATGACTTATTTTTTTGGATTGATTATTTATAAGTTGGCGTCATCT

TTCCTCTTTC

>LsbHLHR6

ATTTTCTTCATTTTCCATTCCTCTGTTTTGTCCTGTTTCTTATGCACTCAACATACATCA

CACTTCAAACTTCAAACTCCAAAAACAACTTAATCATTTTTTTCTTCTTATAGATCTGAA

AAAAGGGTCAAAGAAAGTTTAGTTTTTGAAAGAAGAAAAATTCAATTCGAGTAGAAATCT

ATGGAGTCAGATCTTCATCAGCAGCAACAACCACAGGCTAATCCTTCAGGTTTAACTCGG

TATCGTTCAGCACCAAGTTCATATTTCAACAACATCATTGACAAAGAATTCTACGAGCAT

GTTTTCAATCGACCTTCAAGTCCAGAAACAGAGCGAGTTTTTTCAAGGTTTATGAACAGT

TTAGGTTCAGAAGAAGATTTACTCACTCCAAAAATCTCTGTTGAATCTACGGTAAAAGAA

GAAGAAGAGATTGTTAATGTTAACATTAACCAACAACAACAAGAACAGCAACAAGAACAA

GAACACATGATTCATCATCATCAGAGTAATAACTATGAACACAACCCTGTTTCTTCTCAT

GGTTTTTATCAAACCTCAGTGATGCCACCTTTGCCAAACCAGAATGTTGTTTCTGGTTTG

GATGCAAGTTTTCAGATGAAGAGTCATGGTGGAAATAATTCTAATCTTATTAGACATAGT

AGTTCACCAGCTGGATTATTTTCTCAAATCAACATTGAAAATGGGTATGTTAGTATGAGA

GGAATGGGAAATTTAGGAGCTGTTAATAGTTCTATGAAAGATGCAAAATTTTCTACTGGA

AGGAGTTTGAAGAATTCAGCAAACTATTCATCTGGGATAATGTCAACAATAGCTGAAGTT

GGGGACAAATGCAATGAAGAAAATAATTTAGAAAGTGAAGTTTTTGGTGAAAGTCACGGA

AACGATTATATCGCGGATTATCAGGTTGTTGATACTTGGGATGATACCGAAATGATGTCC

GAAAATGTTGGTGGTTTGAAAAGATTTAGAGATAGTGATTCGAAACAACAATTTTCTGCA

GGTTTTAATGCAGTTGTAGTTCAGAATGAAACAGGAGGACATTCATCTTCTCCATTGGCT

CATCAGTTGAGTATGCCAAATACTTCATCTGAAATTGCAGCTATTGAGAAGTTTCTGCAT

TTCTCGGATTCTGTTCCGATGAAAATTCGCGCAAAGCGCGGTTGCGCTACTCACCCAAGA

AGCATAGCCGAGAGGGTTAGAAGGACTAAAATTAGTGAACGTATGAGAAAGCTACAAGAT

CTCGTCCCAAACATGGATAAGCAAACAAATACAGCAGATATGTTAGACTTGGCTGTGGAT

TACATCAAAGACCTTCAAAAGCAAGCTCAGAAGCTTCAAGATTGTCAAGCAAAGTGCACT

TGCTCACACAAGAAGCCACAATAACATCAAGAATGAATGAATTTCTTAGTCAATGTATAG

ATTTGTAATAATTAATTTGGTCACCAAGTTAAAAAAAAAAAGCTAGCTAGATTAGCTTTT

ATTTGTTAATGCATTCACCTTTGATGTATATAAAGAAAAATGATAAATTTGTAGGGTGAC

ATTTTGTTGAATATTTATGGAAGTAGATGGTAGGTAGGGAAGAAAAGTCAGTTATAAAAG

TTGAGTGTTAATTAGGGAAGCATGTGTGTACAAAAAGTCTATGTGTTAAGTAGACGTAAT

TTAAGCATTTAGGGTCCATATTTGGTGATGAAATGTGTGGTTTTGCCGTAACTAATGTGT

TTTCATTTCACATTGAATATTGAATGAATGGCTGGCATTTGATAATATGGGAGGCGCCGT

GACACCGACATTTTTTGTTGGATATGTGATATACTATGATGTCTATGTGTGAGGTTTAGG

ATTTACCTTACTCTTTTCAAAAAAAAAGAAAGAACTCAATGTTGGTGGTTGTTGTTGCAT

TTTGGTGTTAGTTATGTAAAGTCAAATGAATAGCATTCATTTCATATTTTTTAAGCTTAA

TGTGAAGAGAATATAGTTGTGATCAATCATAAACATGTTGTCATGTTTAGATGGTATATT

ATTTCTTAAAGTATCAAGACAAAAATGTCTACATGTTAGAGTATACTTGAATGTATGTAA

AATAAACTTG

>LsbHLHR7

AACAACTTTTGTACATTTCAATGAAGGTTGACCAGAACAATTATAGAGAAAATCTACCCA

TCAGAGATTCAAACAGCCCCTGTTTCATAAAGCATGCATTCTTCCCAGAAAAGAATTCTT

AGCACAACACATAACAGCATCATGCAAATTCGTATCCAAGGTTATTTACTTATAAATTAC

ACTCACAATATAACCATACATAATAAACATTATTTTTAGGAAAGAACGGTTAATCCCAAC

AATTCATACAGAAAATAGAAAATAAATAACAAGCACCAAGTAGAAGTACAACTATTACAT

TATTTTCTTACATGACCAGAATAAGTAAAATGTCAGTTTAATTTATTACTTTGCAAGGCA

TCTTCCCTCGAATCGTAACGAGGAAGGTCAGCAAATGGCTTTCCACTGCAGTGTATTGTC

CAACTAACATTTTCATTGTCCAACTCCAAATTGTTCCACTTCAACCTTGAGAGACTTGAG

GTATTTTACAGCTTCGTCCAAAACAGTGACGGTATCCATTTCGTTGCCACTGCCAGGCAC

GATATTTCTCAATATTTTCACCATCCTCTTCATCTCCCGGTGTTGTTTCTTTTCACTGCT

GCTGCAGTAACCTTTGGTACCCGAGGAGTTCTGTACAGAAGATGATAACAACCTCTTCTT

CCTTGATTTTGTGCAATAGCTCGAGCAAGTATCAGATGTACTTTCATCCTTTTCATGATG

AGTCCTTGCTGTGCTAACTTCTTCTTCATCATAGTCTTCTAACTCATCTGAGTCTAGGCT

CATCAACGCATCAATGTCCCTAGAGTCCTCTTCAAAAGAAGAGGACAATTCCCTTTCCAT

TTGATTAATTTTGTCTTTTTCAAAATCTTGAGTGTATGCATGAGTGTTGAAGTTGAAGCT

AGGACTGTTAAATTTGTAGGTCGTTGCAGGGTTGAACAAAACCCTGTTTTGTTGATCGGT

TTGATCGAAGACAATGAAATTCTCGGGGCAAACCTCCGAGGGATGCAAGTTAAACCTTTC

AAAGGGCGTTATCTGCCTTGCACCTGTAGGTAAAACTGCACCAAATGCAGAAGTATGTGG

AGCATTCATATAAGTACCAATGGCTTCATCTACCAAAGGAAGTTTCACCTTTCCCCGATG

AAAATACTCGTGAGTCTGCATTATAATATCTAATTCACTCACTAAATTACGTGGAAGCAA

TGCTGATCTGGCCTATGCAACTGGAAATTATTTTGATCTAAAGCTGGATGGATGTTCGTA

CAGGATGTAACAACTTAACAGCAGGAAGTAAGTTTATTCCAATTCCATCTAATAAAAAAT

GGTAACTTTTACAAATTAAAACTTTGTCTTTTCGGACAGCCTTCTAATGATGCAGTTCAA

TATAAGGCAATCTCTACCCAAGTAAACGTTCTTTGAAGCTAGACTACGTAACAGGTTTAA

GCAACTGACGAAAATATTCAGCCTGACAAACCAACATCAATTGACTGTATGCAATAACTC

CAACACTCATTTTAATGTAGTCAACTTTCGCAAAGAACATTCTCAACCCTCCCAGTAGTT

TTGGTAATAAAAAGGAGAAAACACTAATCAGAGGAGTTCCTCTGCAGCTTAATCACCTCA

GATGATTAAATTACAGAATCACGCGAAAAAACACCACCAAGCAAACACAACTTCTCTTGA

AAAGAGAAAACCACGGCATCTTGATTCAGCTCAACTTCAGAATATCATATAAGATAAACC

CAAAGCTCTGACCTGAGATTTGTGCAGAAGATGAAGAGGATTTGGGAGGAAATTGAGGGT

GGATCGGAGAAGGGTTGTGTTGAGATGAAAATGAGTAGAAGAATTTTTTTGCGGAGAAGA

GTTTTGAGATTGGGAATGGAGCAATTTCTCTTTCAAGATATGAAGGCGTGATGGGATGGG

TCAAAGATGGATGATCTGAGCCGTTGGATTGGATCCAAATTGTTCTTGGAAATGCACTTC

CGTTTATCGCGGGCGTGATGGGGACCGTTCGATTAGAGCACGCGTGAAGAGGGATGGTGC

CCGTGAGGTTGACAAATTGAA

>LsbHLHR8

CTATGATTAACCAACAATTCAATCCTACTTGGAGGTACAAAGTGCAAGTGCAAGTGCATA

TTGGAGAACAAGCAAAATCACGTAAATGAATCCAACACACTAGCTCAATGAGTACAACTC

AATGAATGATAAGCTGAAAAATAACATGTTACCCATTTATGAGAAAGATACATACACATA

TATACACACACGGGCACGGATACATAATATTACATATTAAAATAGACTTAGTTTTTTCTT

ACTTCATCAAAGCTAAATCTATGACAGTGAGAAAACTGTCCCTAGCATATTCTAGTTCTA

ATACCCCATTACCATTTAACTTCTCAAATTAAATTACCTCAAAATTCATACTAAGCTACC

AAAAACAAAGACACTACTAGTTTACAGAGATAGCTTCCGGGAATCAGTCAAATATCCCAG

ACCCTCACAATTTTGGAGGAGTGAGAGATTCACAGACATATCATTCCCGGTTAAATTCTC

TCATTGCAGCAGATTTGCAGATTCTATTGAACATCCCCGACTTGAGAGGACAATGCTGCC

CGAAGCTGCTCCTGGGTGTAAAAACGACTCCCCATTTTCACAGTTGCTTGTTGTATCATC

AAGTCATTCACCACAGACACACTGGCATGGTGCACTTCTAGGTCAAGCTCCATTAATGCC

GCCATCAACCTCGCTGCAGGGTGGTTTTTCTTACTGCATTGGATCCTTATCATTGCATCC

CAACCTATAATCTTGACGTCGATATCCAAATCAATTAAAGCTTGATTGGAAGGTGATGAT

TTTTTGGGTACTGGTTGCTGTTGTTGTGGTGGTGTGGGTGGGGGATGAGACGAGTTTTCA

TTGATTTTCTGAAGCTCACTCTTCACTTCATCAAATTGCTTTTCTAATCCATCTTTATCG

GATTCTGTTTTCTGAAGCTTTGTTTTCAGTTCAGTAATGTAAGATATAGCATCGCCCAAT

AGTGAAGCTTTATCCATCTTTGAAACATTAGGCACAACCGCACGAAGAGCATAGAATCTC

TGATTCAGCTTCTCTCTTCTTTGTCTCTCAGCTTCAACATGATTCAATGGTTCTTCTCTT

CCATTTGCTGGTTTCCTTCCTCTCTTCCTTGGCTTCTTTTCTGGCTCCACCACACGACTG

CTATCCACCTCCTTCACCACCGAAGCCTCCAGATCCGAATGGTCTGAATCACCACAACCA

GTACCACTTGAGAATTTCAAATTCGACGCCGCCGGTGGGACAATTATGCCGGAAGTAAAA

GATAACATTCCATCATCGTTGTTGCTTCCTCTTGAATTAGGCGATCTCCTCTTTCCATTT

CCACTGTTGGTGTTATTGTTGTGGTTTTCCTCAGCAACCGATAGTAAATTTGATTGACCG

GAGAAGAAATTCGAATTTGAATTTCCATTATTAGTAACATAAGAAGTCTTTTTACTCTCG

CCTCCAAAACTCAGAATCTCACCGGATTCCGGCTTCAATGAACGCTGATGATGGTTACTC

CCACCGTATTCAGAAACATTCATTTCCTTATTAAAGAAACTTTGATTATTCTGCTTGCTT

GAAACATGAACAACACTAGGAACTTCCGTTAAAGTACTTGAACCATGAGTTTCAAATTGT

ATCGTCTTGGTCACACTCACACTCAAACTCTGATTATTGTTATTGTTCTGAGGTTGTTGT

TGGTGTTGATGTTGTTGGTGACTTGGATTTGAAATCGAAGTGTTTGTTGTTGTTGGTGCA

GCAAGAGAATTATTATCAACGGAATCTCTAGTTTCAGGATCGTTAAGCCAAATCGAAGAA

GGATCATTTTCACCTTGATGAGCTATTGCAGCAGAATTACCTAACTGCCAAGAAGATCCA

AACTCAAAGCTATTATTGAAATTGAAAAGCATCTTCACTTTATTCATCAAATCATTGTTT

TGATAAATCAATTCCGTAGATCCAAGCTCCAAAACACCGTTCGGCGACGGTATACACGCC

AACGTCTGTAAACCGTATTCCTGACCCTGTCTCGCTCTCTCGCACACCGAGACGGAGAGA

CTCTCGCCGCCGACCAACCACACCGGAGTTGAGTTAAAGTAAGCTTGTCCGGGAAGGCCG

CCGCCGTTGACAAAAGACTGAGTCATAGAAACTAAAAAGAACCATTCCGTATCGGTAACT

TCTTCATCGACGGCGGATTCGTCGGTAACTGGATTACCGGAGATTAGAGAATTAAGTTCC

CGGAGAACTTTTCTCCGGTGTTCTTGTTCAGCAGGTGAAGAAGCTTTTGATTTTGACTTT

GATTTCGCTTTTGTTTTATCTTCTTCTCCTTTGTAGTAACCGTCACCCCAACCGAGAAGA

GAAGTACCGGAATAGTCATAAGACGGTTGCCAGAAGATAGCATATGTCCAAAGTTCCGAT

GCGCCTTCAATTAAAGCTTGAAGCCTTTGCTGGAGTGTGTCTTGGTTGAATCCGGTGGTT

GTTTGTGGTGGCTGAGACGGCGGCGCCGGTGGCCATAAGGTGGATAAATCGGAGGAGGTC

ATGAAAGCCTCCATTACTGAAGAGTTATCGTCGCTCCAGAGATTCATTTACTTAAGTTGA

AACGAAATGGAAATTTTTTGGATTAATTTGAGGGTAAATTAGGGAGAAGAAAAGAAAAAC

AAGACAAGCTCACGCAGAACACACCGTGTGGTGTTGATAATGGATTGGGAGGAGAGAGAG

AAGGGACAAATGAGGATTGGTTATTTTTGGGTTTGGAATTAATTTGGAGTGAATTTAGGT

TGAATTTGAGAGTGATTGTAAGGTTTCCTTTCAT

>LsbHLHR9

GGTTGAGATTAAGAGTTAAGTGTGAAAATTTCCAACTAGAAAATAGAAAACTCACACACT

CTTCTCTTCCACCATAGACATTATTTCACTCTTTGCATTCTCACTTTCTTCTCAGAAATT

GGCAACATTTGTAAACTCCTTTTCTCTCTCTTTCTCTGTGAAAACGGTAAGAAGAACTTC

TGAATTTACCTTACAAATATCACAAATCTTCATTGTTAGCAAATTCGTTCACAATTTCAG

AGAAATTCAGCATCCCAACAACCTTAAACACTTTGATATGTCATCAGATATAAGCTAAAT

TTTGGAATTAAGCTTCAAGAGCGAAAGGTGTTTGTTTAAATGCGCGCTAATTTTATGGCT

ATTGGGAAATTTTGTTGTGCTAGTAGTGTTGTTTTTTCAGTGTAATTGAGGGTTTGGTTG

TGTTTAGTTTAACGATTGTGAGGTAAAGTTTGAAGCTTTTGTGGGTGGGGTGGTGGGAAT

TTTGTGGTGTGAGTGATGGGTGGGGATCGTGTGTTACTACTACAAACAGTTGGAAGAGAA

CCGCCATTAACACGGCGAGCTGGGTTGCGAAGGAAGCAGGCTGGAAGAGGGTCTTACAGA

GGGAGCTAGCTATAGTTAGGGGAAGGTGATTGATTGGTTCTTTCTGTGTGATTGGATTTG

GGGGGTTTTTTTCGTCTTTTTTTGGAGGTTTCAAGGTTTGTAAGTTTTTAAAGGGGAGAA

GAAGATGGGTGACAATTTACATCAACTACTCAGAAGTCTTTGTTTCAATACTCATTGGAA

TTATGCTATCTTTTGGAAGCTCAAACATTGTGCTCCAAATCCAATGATCTTAACATTGGA

GGATGCTTACTATGACAATTCGGACTATTTTGACTCTTCAGAAAATAAGTACTGTCAAAA

GACCTTGGATCAGATTAAGGGTGGGAAATTTTCGCATGAAGCTTTGGGGTTAGCTGTGGC

GAAAATGTCATATAATGTATACTCATTAGGAGAAGGCATTGTTGGACAGGTAGCTGTTAC

TGGAAAACATCGGTGGATCTGTGCAGATGATCAAGCAACAAGTTCTGGCTTGTCTTTTGA

GTTTGATGATTTGTGGCAAACTCAGTTTTCTGCTGGAATTAGGACAATTGTCGTAATGTC

TGTAGTCCCGCTCGGCGTTGTTCAGCTTGGCTCTCTAATAAAAGTGAATGAAGATATGGG

GGTTATTAATCAGCTAAGAAATCTTTTTTTGTCTACTCAAGATTACTCAATAGACCACAT

TCCTAGTCAAATACAATCTAGTTTGAAGAGCTCTTCATCACAGGATATCTTGAAGGAAAA

ATCATCTTCAGATATTATGCCTGCATGCATGACGAATGAAACAGTGGGTCTTTTGATGCC

CCTTCAATGTTCTGGGAGGAACGGTGCAACTAATTCTGCATATTGGGAAATGGGCGATGA

TGTGGTCAAGTATGAAGGACCCGAGCTCAATAGTGATGCGAGTCCCATTTTGCTTCAGTC

GGCGTTTGGTATGATTAATGTGGAGCATCAGGAATTTGGAGAGATAAGACCTCTAAGTGC

GAGGGAGTGTGCAGGGGGCAGTGATGGCTGCAAAAATATGAAATTGGAATCGGAACAAAA

TCTATCATCATTTTTGAACAATTCTGTTATAAATAATGATGGCGTCGGTGATTTGATACA

CCGTTCAGAAAAGGCCAGAGTTGATTCTGCATGCTTTCCGACAGATTTTCTTGATGCATA

TGTTTCTGAAAGTGACAAGTCTCATAAACACTGCGAAAAGTCAGAGTTTTGGACTGTGCC

TTGTGGCAAGGATACCTCCTATACAGAGTTGAGTTTTCCGGCTGGCTGTGAACTACACGA

AGCACTTGGACCAGCATCGTTGAAAGGGAGTAAATATTTTGATTTGCTGGCACAGGTTAA

TCAAAATGTGAAAATTGTTGATATGCCAGATGCAGTTAACACTAGTCAATCGACTTGTCA

ATCTCCTCCAGAGCATCTTTTGGAAGCAATGGTGGCCAACATTTGTCACAGTAGTAATGA

TGATGTTAATAGCGAATCATCATTTTATAGATCAAAGCAATCTGCAATAAGTTCTGGCAA

AAAACCTGAAGTTTCTATTCAAAATGTGCATACTGTTAATTCAGAATGCTATTCAATAGA

TCACCCTTCTCTTTTCCAAGAGGGAAAACACCATTGTTTGAGCTCATCGTCAGGGATATG

CGGTGTTATGTCATCTAAAGGCATTTCTTCTATATGTCCTAGTGCTTGTAGTGAGCAACT

CGAACGATCATCTGGACCATCTAAGAACAGCAAGAAGAGGGCTAGACCTGGAGAAAGTTG

CAGACCTCGGCCAAGAGACAGGCAACTGATCCAAGATCGAATTAAGGAGCTGAGAGATTT

GGTTCCAAATGGTGCAAAGTGCAGTATTGATTCACTACTGGAGCGCTCAATAAAGCACAT

GCTCTTTCTGCAAAGCGTAACTAAGCACGCTGACAAGCTAACAAAATTTGCTGTTTCTAA

ATCAAAGCTGCATCATGTGGAAGCAGACATTCATGGATCCTCTAGTAGTGAGCAGGGTTC

AAGTTGGGCAATGGAGGTAGGGGGTCATCTAAAAGTTCATTCAATATTAGTGGAGAATCT

TAGCAAGAACGGACAGATGCTTGTTGAGATGCTATGTGAAGAGTGGAGCCATTTTCTTGA

AATAGCAGAAGCCATAAGAAGCTTAGGCCTTACAATTTTGAAGGGTGCAACAAAAACTCG

CGATGACAAGATGTTAATATGCTTTATTGTTGAGGTTGAAAACAACAAAAATATACACCG

ATTGGACATCTTGTGGCCGCTTGTTCAGATACTGCAATCCAAGAGCAATGTGCAGCAACA

GTAACTAGTTGATTCGTTGAAACTTCGCGGCCCTTTATTTGTTGCCGGAATTCATTATTG

ATCATTGAAATGATGCTCAAGATTTCTTACCCTTGTGTGTTTAAGACTCTGATTGTTTTT

TTTTTTAAAGTCTGGTTTGAATATTTGAGTGTTTGTGTTTGGCATTGAAAAGTTATTTTA

AATCACTTGCAATTTCTGAAACATTAAAAAGTGGTTGGACATGTGGTTAGCCTAAATAGT

AAGGGACTGTGATAGTATCTTTAAGTTCAAATAGGGTTAGGTTTTTATATCTAATTCAAT

ATTACGAAATTGGCTCTAAGGTGAAAGATGTCGATTTATAATTTCTT

>LsbHLHR10

ATTCTTCTCAACCTTCAATTCTTTAATCTTTTCTTGAAGACCCGTATTAGCATCTTTCAA

TTTTTCGGCTTCACCCTTCAGCTGTGTCACCATTCGGACAGCATCAATCAGGATAGCAGC

CTTGTCTGTTTTGGCAGGCCTTCCAGGCTCCAAAATGGAGCCCAATTCAATAAACTTGTC

ATTAAGCCTATCTCTACGCAACTTCTCCCGACATGCCTTGGAGCTAGTGGCAGCACATGA

CTCAGATCTACCCCTCTTCTTTGAACCAGACTCTTTGAGACTGTCAGAATCCCCTAATGA

GACGTCAATTTCAGCACCGACATTGGAGGAAACATTGAATGGCTGGTGAGCGGGCCAGGT

GAAGCCGGTGGAAGGGGTGGGCATGAAGGTGGAAATGGGAGGGGAAATATCGTCGATTAA

GCCGTAATCGAAAAGCCAATTGTTGTTTTCCCGGGAAACCATTTTTGTACCCTAAGTTCC

CAGAAAAAAAATTGGGAAATTGAGTCGCTTTGATGTTGAAGGGAAACAAATAAGGAGTGG

GGGTTTGGTTAAGTTAAACGAATGAGAATCGAGAAGGGAAAAAAGAGGGATTTCCCTTTA

TTTTATTTTATTTAATTCAAT

>LsbHLHR11

CATACCAACCATTTATGCAAAACTAGTTAGCATAAAAAAGCCATCAACTCACCAAAATAT

ACTAAACAAATAAATAAGCATTACACACACACACTTTAGTATAAGATACTAGCAACTAAA

ATTCCAATAAAATAAACAGAAAAACAATAACATGATGTACCCTATCTCTATTTTGTGAGC

TACCTAATTAACATGATACATCATACATTGATATATGTAGACAAGAAGTCGTTGCCCTAT

GTTCCCCGTCACCTCTTTCTCCCCTTCAATAATTTCTTGTCCTTTATTCTTCTGATATTC

ACTCCCCTAAGGTTAAACCTAGTTACCATTAATGTAGTTGAATATTTCATTAACTGCTTC

TGCTATGTCATTTGCTGTGCCTAGCTTACAATCTTCTTCAACCTTGAGATTGAGGGAGTA

AAGTACAGAAGACTCAAAGGATGTGATGTTAAGGTGAAGAATAGTTAGCCTAAGATTCTC

CAAAGCAACAATAACTTTTATCAATTGACCACATCTTCTTTTGCACTCTATTTTCAAGTT

CACATGGGTTTGAATCAATGTCACTTTTATATCTGCTGCTTCTGATTTGTTTTCTGCCTT

CACCTCATTTTCTTCACAACATGATAATTTCATTCCATAACTAGCTTGCGGTGGTGAGGA

TGATGAGGATGAAGATCCAAATTCTTCATTCTTTTTTATCCTTTTTTGTACTTCAAGAGA

TTCAAGCAATTGTTCTAACTCCTTCACAAAATCTATTGCTCCTCCAATTATTGATGCTTG

GTCACCCCTTTGAATATAAGAAGATGGCATAAGAGACCTAAGAACACTAAGGTGGTCATT

CATTTGTCTCCTCCTATTCCTTTCAACAGCAATGTGTGTCATCCTTTGATTCTCAACATC

CTCCTTATTCTTAACCGGTCTTGTTCTTTTCCTCTTCCTCTTTTCCCTAGTTGTCACAAG

TTGAGATTTGGAACATGTTTTTGTTTCTTCTTGGTTACACTCATAGCTCAATTTCTCAAT

GCATGAAGCTGAAACTTTGTGTTGAAGTTCATAGCTTTCTGATTTCACCGGTGACTGCAT

CTCTAGAACATCATGTGTAACACAGCTTTCAAATTCCAATGTTGTTTGAACTTGTTGGAG

TGAATCCATTCTAGGAATAAAAGTTATGTCTTCCTCCCATGGTTTCTTCATATGTTGTAG

CCTCAAAAGTGTTTGAAAGTTTGGTTCTTTTAAAGGATAAATTGGAGGTTCAACACTTTG

AAGCATTTGAAGAAATGGCATGTTGTCTTCTAAACTAGATAACAAAAACTGTTCATCTTC

CTCAAATCTTAAACTTTCTGTGTCAAGTAATGTTTGGTCTAAACAATTCACTTCACCAAA

GAAAGAAGAATTGATGGGTCCTTGAAGCCTCTCCATGAAATAAAGATGAGAAATTTAATG

CTATGAATTTTTTTCTTAGTTTAGGTATCAATGAATGTGACCAACATTCTGTAACAAAAG

AATGGTAATAAATGTGAAAGCATAGAAAGAAGCAGGAGAAGACCCCATATGAATAACAAA

CACTAGAAAAAAAAAACTATTCTTTTGTTCTTATGATGTATCTTTGATAGTGTTATATAT

AATAGTAGATGTTTTGTTATAGGAACTAAGTTTTTGAGTCTAGTAAAAAGGAAATAGATT

CTTTTTTTGTGTGTGTGAAAAGAGAGAGAAAGTTTTGATGTAGAAACAGTGGAGGGATTG

TGGTAAGAAATGAAAGGTGGAAAGTTTTCAAATGGGTTGCTAGAGATATGCTATGCTATG

CTAGCAGATACTACATTTGAAGGAGAGAGAGAGAGAGAGAGGAGAGAGGTAGATACGGTT

TTTTTAAGGCCGGAGAATTTATAAGTAAC

>LsbHLHR12

TTTTCAACTTATTCAACATAAGTTCACAAACAAAAAACCATGTTGAATCTACAAAAATGA

ATGTTGAGACCAATATACAATATATAACCAATATGTATTAAGAACAAGCATCATTCTCTT

ACAACAACAATGATAATTTTAAAGTTATGAAGAATGCCAGCATATAAAGCCTATAGTCCA

CTGAGCTACATCTTCTGTAAATGAATTCATATCCAACACACTTACAAATGGTGTTGCTTA

CATAAATACTAACCGTAGCGACAGTGTGGTTTAGAAAAAGGAATCAAGTAGTGTTCATGT

CGACTTTTATACCTTGAGTATATGATTTATGGGAGCTCTGAAGTAGACAATAAAATTGCT

ATTTGCATACCAACTGCTGAAATTTTAAGATTACGTCGACCTTTCAAAACCACCTCCCAC

CTGCATATGTAACCACTCTGGTGATGAACCCCGGCTGTACTCTCTTGTAGCTTGTGAAAC

AAATGGTATGGGGGTTGTTTCATATGTTTGCTGATCAAAAACTTTAGGAGGAAACACTTC

AATACCAGGTGTCAATCTTGAATTTACTGCTTCCAGCTTCATCGACAAAAACTCTACTTG

GTGATGAAGTGATTGGATATAATTGATTATCTCATCAAGGACCAATGCTTTCCCAATAAC

CTTGTTGCAACCAGGGACTAAATCTTGAAGAGTTTTCATCCTTTCACTAATCTTTTCTCT

TCTAGCTCTTTCGGCAAGACTGTGGCTATCAGTAGCTTGACCCCTTCTAGCTCGAACATG

AATAAAATCTTGCTTAGGATGTGGCTCTTCAGTAATTTTTTCCGCATGCTTTCCTGAACT

GTTTTCATCTCCTACTACTTCCTCATCTTTACTCTTCCATGATGTCTTAATCCTTTTTCC

ATCATCACCACCATCACCATTCACGTCGTTGCTGGTGGAAACGCCTTTAACAGAATCCTC

CTCCGTGTCGCGCCTTTTCCGGCCACGGTTCGGAAGTCTGAGCTCAGAACCAACCAATTC

TCTACCGGGAACTTCCGTTGCGAAAGCTCCGAAACCATGCTCGAACTGCGGTGTTGTCGA

GGGTGGGAACTGCCAGATCTCCGGTGGGTGAAACGACAGCGTGTTGGGAGCAGCGTTGAT

TATTGGCGCTGGATCCATGAAAAGATTGGAAATTCCAAAAATAGCCCTATATGTTTAAGA

GCGAGGGAAGAATGAGATAATAAAACAAAGCATTCGAATTGAAGTTGTAGGAGAGAGAAG

AGAATCGTTTGGAGTGGTTGAATAGTGATATGGTTGGGAGAGTAGAGTAGTGCTCAGAGT

GGTAGTGTTGATAATTTTCTGTCTTTACTCTTGCTGTTTTAGAGACAACTACTTTGGGAG

TCAAATACAAGTCACTTCCATAGTCCCACCCTGCTCGCGCGGGTA

>LsbHLHR13

GTATTAATTAATATGATTTTATTCATGTTATGGAGTAGTATAAAGACACTTAACATATAG

GCTATGTACTTGTAGAAAAAAAAAATCTTGATAAGAGGCATTTGGATACTGTCATAGATT

TTGTGGTCTACTGCATCTCGATCTTTAGATTGCTAGCTTCAACTAGACCTGTGTAAAACT

GAGGTTGGGAAGGAAAAGAGGTTGCTCTTGCTTGATCAAAATTCATGTTGTAAAGGTTTT

GGAAATCACCTTCCCAAGATGAAGAGGGTAAGATATGGGTGAAACACGACGAGTCAAAGA

TTTCGGGTAAAGATGAAGCTGCGGGGATATTAATGTTCCTCCGAAGCCCGATTTCCGGAG

GGATTATCCCCATATTGTTTATTAATCCGCCACAACTCGAAACTTGTTGTGCTGAATTGA

ATTGAAGATATGTAGGGTTAGTCATGTCTGATTGCATCCCTATGGTTGGAAAATTTTGAG

GAAAAACCTCTTTGGCAAACAATTCATCAATGTTGAAGTCAAGTCTCGGATTTACCGCTG

CTAATTTCATCGACAAGAACTCAACTTGTCTTTGAAGAGATTGAACATAGTTAATGATTT

CATCAAGCATTCCAGCTTTTCCTGCAATTTTGTTGCATCCTGGCACTAAATCTTGTAAAT

ACTTCATCCTCTCGCTTATCTTTTCCCTTCTAACTCTTTCTGCTAAGCTATGACTATCAG

TGGCTTGACCGCGACGTGCTCGAACATGAATGTAATCAAGCTTCTCCTTAGAATTAGTAT

CACCAGAATTTTCTCGGTTGTTCTTTGTTCCATTGTTGGACTTTTTATTCTTGTGTCCCT

TTGAAGTTTGGTCACTGGTTTTGGATTCCTCTTCTTCTGCACTTATTTTGATCCTCTTGT

CACACTCCGGAACAACAACAACATCAACCTTAGAATTATTATTGTTAATTTCAGCTTTTC

TTTTCTTAACGGTATCTTTTCCAAACCCCGGGTCGGGTTTGATAGAGTTAGCAACGACTT

CGCCAAGAGCGGAATCACAAGCAACCATCGTAAACTGTGGAGAAGAAGAAAATTGTTGAA

AGTTGATTCTTGCTTTTTGTCTTTGTAACACTGTCATGTCGGAAATGGTTGTTAATGTTT

CTTCTCCTCCTAGATTACCCGAAGTGTTGAGACAGTGTAACATGTTGTTGTTGTCTTTGT

TATTGTTAATAGGAATAGGTACTTTAATGTTCTCGGTGTGTACTATTTTCTGTTGTGCTA

AATTGCGCAGTGGTGGGTAATGTTGATAACGTGCGCTACGCTCGCACAGGGAAGAAAAGA

GTGATGTGAGAGAAGAGGCTGTTGAAGAACATATATATAGTAATAATTAATTAATGGG

>LsbHLHR14

CCTTAGTTCGTTTGATTACACTTTTGCAGCGGTTACAGTTTAGAACTTCAGAGTTAGAAA

GCCTAACGCCGTTCTTCTCTCTCTAACATCTCAAAATTGTTTCAGCTTTTTTCATTCTCA

CCTTCATTTCATTGCATAAAAAAACAGTGAACCTTTTTTTCTTTGTTTTGAAACATGTTT

CAACTGTATGATGTTTGTTCTGCCATTGTTGTTGCCTCAATCACGGTGTCTTGTTTTCTA

TTTGGTCTTTCATGAATCTTATTTCTGGTTCCACAATTTATAGAACCTGAAATTGGTTTT

TTTTTTGTGAGATCTGAGATTGGTGGTTTTGATGCAAAGTGCTTAATTTGAAGTTTTGGG

TTTGGTTAATTATTTTTATTTTTGTTGAAAGTTGAGATTTTTTTGGAGTTTGGTTTCTGG

GTTTTGTTTAGAAGATGAAGATTGAAGTGGGTTTAGGAGGAAATGGGTTATGGAATGAAG

ATGAAAAAGCTATGGTGGTTGAAGTTTTAGGTGTAAAAGCATTTGATTACTTGGTTACAA

AATCAGTTTCTAATGAGAATCTTTTGATGGCTATTGGAAGTGGTGAGAATCTCCAAAACA

AGCTTTCTGATCTAGTTGAGAGATCCAACGTTTTGAATTTCAGTTGGAATTACGCAATTT

TCTGGCAAATTTCTCAGTCAAAATATGGTGATTGGGTTTTAGGTTGGGGAGATGGGTGTT

GTAGGGAACCAAAGGAAGAAGAAGAAAAGGATTTAGGAATGAAAAGTGTTGTTAGTTTGA

ATTCTATTGAAGATGAAAAGCAACAGAGATTAAGGAAAAGGGTGTTGCAGAAGCTTCATA

CAACATTTGGTGGTTCAGATGAAGACAATTATGCGTTTGGATTGGACCGAGTCACCGATA

CCGAGATGTTCTTTCTTGCTTCAATGTATTTCTCTTTTGCTAATGGAGATGGTGGACCAG

GTAAGTGTTTTGCATCTGGGAAGCATTTGTGGCTATGTGATGCATTGAAATCTGGTAGCT

CTGATTATTGTGTTAGGTCTTTCTTGGCGAAATCGGTTGGATTTCAAACTATTGTTTTAG

TTCCTACTGATTTAGGTGTTGTTGAATTGGGTTCTGTTAGAATGGTGGGTGAAAGCTTTG

AGTTGTTGCAGACTGTTAAATCTGTGTTTTCGACACAATCGTCGCTTGCTAGGGTTAAGT

CGATTTCGTCATTGGATGGAAACAGAGGGGAGAATGTGAATGAGAATGAGAATGATAATG

AGATTGCCCCTTTTTCTTGTTTGAAAGTCGGCGAGAGTATTAAGAACAATAATAGTAATG

GTAATCTTCATCTTCGTCGGAATGATAATGATAGTAGAGTAGAGGTGATTGGAGTTCCGA

AGATTTTCGGGCAGGATTTGAACTCGGCGACACATTTTCGGGAGAAACTTGCTGTTAGGA

AAATGGATGAGAGGCCGTCATGGGGAGTCCGTCCGAATGGGAATGGAATTAGTTTTCCGA

ACGGTGTTCATGGTTCGGGTTGGAGAGGAGGTAGTCAAGTTGTGAGGCAGCATGTGCCTG

CCGATGTTTTTGCTCCTAGGCCTTCCGCGAGTAATGCGCCTGAGCTAGCCAATGGGGGCG

GGAGGCATGATTTTGTGCTAAACAACTACCAACAGCAACGGAAGGCACAGATGCAGATTG

ATTTTTCCGGAGCCACATCAAGACCTTCTGTGAGATCGATCGTCGGTGAATCGGAGATTT

CTGACGTTGAGGCCTCTTGCAGGGATGATGCACCGAGTCCCTCCGACGATAGGAGGCCGA

GGAAGCGGGGTAGAAAGCCAGCTCACGGAAGAGTAGAACCTCTTAATCATGTGGAGGCAG

AGAGACAAAGACGCGAGAAGCTAAACCAGCGCTTTTACGCCTTACGAGCTGTTGTCCCAA

ATATCTCCAAGATGGACAAAGCATCTTTATTGGGAGACGCTATCGCTTACATCAACGAAC

TTCAAGCAAAACTCAAGTCAATGGAATCCGAAAGAGAACAACAACTTGCAACCACTTCTA

GAGACGGATCATCATCGGCATTACAAACAAACTCTAGACCGGAAAATCATCAAAACAAAA

CTCCGGAAATCAACATTCAAGCTACACAAGAAGGCGTCATCGTAAAGGTGAGTTTCCCTA

TTGATGTTCACCCTATTTCAAAACTCATCCAAGCATTCAAAGACACCGAAATCACTATCC

TCGAGTCAAAGCTCAACGCCACAAACGACACTGTCTTCCACACATTCATAATCAAGTCCC

AAACCTCCGAGCAGCTTACAAAGGAGAAGTTAATCGCAGCATTTTCCAAAGAATCAAACT

CCTTATAGCCATAGTCCATCCATACTCATGATACATAGAGAAGAACCATTTTGCCCATAC

AAAAGAGGATAAATCTAAATATAAAATGCAGTGTGATCTAGTGAAGTAGAATAATAGGCA

ATATCTCCTATTAACCGTGTAGCATAGCAAATTTTATATTTGTCTATAGTATTATACACA

TTTTATTTATCATCATCATCATAAGTATTATTCCTGCAAATTTCATTGTTTTGTTTAGTT

TTAACCAATCTAAAGCATTCTACTTTTGATTCATAGGAGTGCAGTTTTTGTCTTTCATCA

TGTTCTTTTTCTTGTATTCCATCAAGTTCTGTGCATAACAGCAAATTATTAGTTAATTGT

TTTGCTTCTTTTGTCATTTGGTTCATTTTTAGGGATAACAATATGTTCTCTATTTTTAGA

GGAATTTTCTGAGTTGTAATAACAATATCTTTTATCATTTCCTACTAGAAATGCAAGTTT

CTTATAAAATGTACAAGCTACTCTAAAGAAAGAGT

>LsbHLHR15

CTCGGAGATAATCAATGACAGATTAATAATTTTGGGAAGTTCAAAAATATTTTTAGCTCC

ATATCTTACAATTTAAATTATAATAAACTAAATTTCCTAGAATCTCTGGCATGTGATGTC

AGCTAGCATAAGAACAATACATACATAGTTTGCATTGAGATATACACTATATAATAACTA

TACTTTCACCACTAATTTTTGGATAAAGATGCTTCAAGCTTTTCTTTCAACTGTAGAGCA

GCTGCTTCCCCTCCTTGAGTTCTTATGGAGAAAGTATGAATTACCTTATCCTGTGCAGTT

GAAACATTTGCCTCTTGAGCTATAATTTGATGTTCTCTCAACACTTTAACGATACCAGAA

ACCGGGTGAATATCCAAGGGGCAACTCACTCTCACAACGGCATCATCCTGTCTAGCCTGA

AAATCGATATCTTGCAATGGCAACTGTGAGCCTTTAGCCATGTTTTTCTCAGTTTCCAAA

AGCTTGATCTTCTTCTGGAGATCAGTGATATGGGTGATAGCATCACCAAGTAGAGAAGCC

TTGTCCATTTTAGAAATGTTAGGAACAACAGCTCTTAAGGCATAGAATCTCTGGTTAAGC

TTCTCCCGTCTTTGCCTCTCCGCTTCAACATGATTCAGCGGTTCCTCTCGTCCATTGGCA

GGCTTTCTCCCTCTTTTTCTAGGCTTCCGCTCGTCTCCAAGAGGTGAAGATGACTCTTCG

TTAACCAAATCTATAGAAGAAACTCTTGCTTGAGAAGTGAAATTCCCAGGAGCCATTTGA

CCTAACTGAGGGAACATTTTCGCCTCATTAATGTCTCCTACACCACCATTTGAAGAATTA

CCATAAGCATGATTCGGCCCAAGCGCTTGTACTTCGAAAGAGTCGGAAGTGAAGCCAGAA

TCATCTTCCACCTTCGGGGAGAAACTAATAGTAATGGACTGAGATTTCGCATCTCCCCCT

AAGCTTAACTCACGCCCAAAAATCTTCGGTGCTGCCTTAGCCTGTCCAGAAGTAGATTCT

CCAAACGTTGCCCGGACCATATCCAAAAAACCCTGCTCTTCAGGTACTATCTCCATCGAA

CCAAGCTCAACAACCCCCGCCTTGAGAGGCACAAAAGCAACAGTTTGAAAACCAGCCGAT

TTAGCCAAAAACGACCTCGATTCATACTGCTTCAAACAACTACCAGCATCAGAAGCCCAC

GTCGGTTTACTACACTTAAAAGAACTGCCAGGACCATACTGAGAATTAAAACCAAACACA

TAATAAACAGAGGTCAAGTAAAGCATATACAAATCCGAAACATCATCCAATTTCTTATAA

ACATTCTCAATCGAGTTAGATCCACCCCAGGAAGCATGAATTCTCCCCAACACCCTCCTC

CTAACTTCATCTCTCTCCCTTTCCTGTTCATTCCTCGGACCCCCAACAGGATCCTGACAA

TGTCCCTCCCCATATTTTAACACATACCCACCAGATTTCAAACCCGCAACCTGCCACAAA

ATCGCGTAATTCCACCTAGAACCCTTAACAATATGACACAACCTTTTATGAATACCAGTA

TCCAGATTAGGCGGAACAATAACATCAGAAAAAACATGTTTAGAAACAGCTGTGCTGAAA

AATCCAACAGCATCAGTACCCAATACAGATTCCAACATAGCCTTATCTTCTTCATCCTTA

CAAAACCTCCCACTAGCCATCTTTCTCTCAATTCCTAAAAATTCAAACTTCCAGGGAAAC

AAAAACCCTAAACAGAACCCAAACTTTGCAGCAACTTGTTCAAACTACAAATTTATAAAC

ATAACCTCAGAAGCATAATCTTGGTAAAAAAGACCTGAAACAGAAAGATATTAGGGTTTT

TAAACATGGAATTCAGAAGAACTTCATTCCTGTTGAATCCAACTCCAGATCTCAAAAATC

AACTGCTCAATGCCAATAAATAATATTTCATTTTTATTTTTTTGGTACAAT

>LsbHLHR16

GGCAAATAATGAGGCACCAAAGAAACTACTCAATAAACTTTCTGGTTGATCATTATGTGT

GGCTTATTTATGTTAGACCAGTCAAATGTACTGCCTCTATTTTGCATTGCATGGAAAAAA

ACAAGTTACTGATTTACTACTAAAATAGTATTTCATCTTTTTATTATACTCCCATTATGT

GCCCTTCCATATTCTATATATACACCAAATACCTTTTTGTCACACACCAAACTTTCCCAT

TTCAGTATCCTCACTCATCACACCACTACTACTACTGTCTCTCTTTCTTCATAACTACTT

TACACCATTTTTTCAATCTCTAATGGCAGCTTTTTCATATCAATACAATCCTTTCCTTGT

TGATCACTCACACTCACCATTCATGTTCAACATAAACGCTACTTCTCCTCCTCTTCCTCT

TCCTTCACAGTTTCATCCTCTTCATCAAGATATTATTAACTGTGTTGATCATCAAAGCTC

CAAAGTTAACACCGTCACTGAAAATGAACCTTCTTCTCTCACCAAAAATATTAGTCCTCA

GTCCTCCATGGTATTGGACAAGCTCGAAACCGGCGACGAGCAGGTTACTCAGAAAGTTAA

CCCTACGGAGAAAAAGAGAAGAACTAGAAACAATGGACCTTTCTCAACTAAACCCAAATC

TCGAGAAATTGCAGCAGAAGGTAGAAACAAAAAACAGAAGAAAAACAAACAAGAGGAGAG

AAAGTGTTTGGATGAGCCTCCAACTGGTTACATCCACGTCAGGGCAAGAAGAGGTCAGGC

CACTGATAGCCACAGCCTTGCTGAAAGGGTTAGAAGAGAGAAAATAAGTGAAAGGATGAA

GAAGTTGCAACAACTCGTGCCAGGCTGTGATAAGGTCACTGGAAAAGCCCTTATGTTAGA

TGAAATAATCAACTATGTTCAGTCTTTACAGAATCAAGTAGAGTTTTTATCAATGAAACT

TACTTCTGTGAATCCTATGTTCTATGACATGGCAACAGATCTTGACACTTTCATGGTTAG

ACCAGAGAAATTAAATAACTTAGCATCGCCATCACCACCACTATCATCATCAGTGTCACG

TTGCAACAGTCCCAAACAAGGCACAGTTTTTGCTGATACAACCACCGTGACTCCAACCAA

CATTTTCCAAACTACTAATGATTACAACCACCTTTTAGATACTTCAGCTTCAATTTTTCT

TCAAGGACAGAGATCAAATGTTGTGTCTGAGGATGGTAGCCATTTTTGGGAGGTAGAAGA

GCAACGACAAAAGTTTCTTAATTCACATGGATTCAACAACAACTTATGTTCATTTTCATT

AATTTAAAATTCATAGAGGTGCATGTCATCATGTCCTACAATTCCACCAACATGTTTGAT

TTAGCATTTACATTAGGTAAGGAAATTCCAGAAAGAATATGAATATGTGTATATTAAAGG

GTATGAAAATGAATGGATGAAGAACAAGAAAATGACAGAAAATGCAGCAGACCAAATAAA

AGACCATATCTTTTTTAGATCTGTGTTCTGTTCCAGTTATTTTTGACTCTTATGTTACAC

AAATTCCTAGAGACTAGTATTGATCCTACGTAGACATCACAGACAGTGAAATTAATTTGT

GTCATTGTATATTATTGAACTAGTGATTGATTCTTGTGGTGGCAGTTATTCTGTTGGTAC

AGTTAATAAATTTTTTATCATTATTGAGAAATGAGAGGTTGAATACATTATTGGCCATGC

AATTGTGTTGGTCTATGCCTAGCTAGATTTATGTATATGTCTCCGTAGATTCTTCAAATT

TTGGAATTTTATACTATAT

>LsbHLHR17

CTATAATTTATGATTGATAGTCTTTTATATAAAGGATAAACAAAGCAACTTCTCTCACAC

TATTCAGACAGAAAGGTTCTAGCATTTGACTCATGATGGAAGTTGCAGCTAGCAAAATGA

CACCAAATGATGAGTCATGGACAAGTTGGTTATGTGATTTGGATCCAGAAGATTACAAAA

TCATCAATGACATAAACATAGTACCTTTGTCACAAGACCATAACATCTTGCAACAATCTT

TGTCCAGTGGTAGCCATTGTTCTCACACAACCTCAAGCACTATGAGTAATTCCTCAGGAG

ATGTTGTTAACAGCTTTGAGAGACCTACAAAGACACTCAAGACTAATAGCCCTTCAAATA

TAGGGTCTCATTCCTACGTTCTTTGTTTCAACAATCCAGAACCAGAGCAAGAACAAGAAC

TAGAACCTAAAGGTAAGATTTTGAATTATGGCAAGTGTTTAACCTCTCAAGGGTTATCGG

AAAATCAGAAAAAGGAAACTAAACGAAGTATTGTAGAGAGCAAAAAGAGTGATTCAGTTG

CAAAACATGCTCAAGATCACATAATTGCTGAGAGAAAAAGAAGAGAAAAACTTAGTCAAC

AGTTCATAGCTCTTTCAGCACTGATTCCAGATTTAAAAAAGATGGATAAGGCTTCTGTGT

TAGGAGATGCAATAAACCATGTGAAACAGCTTCAAGAACAAGTGAAGATATTGGAACAGA

AGAACCAAAGGAAAAACATTGAATCTGTGGTTTATGTTGAGAAGACAACAAAGTCATGTT

CTTCAGATGAAGATGTTTCAGTTTCAGACACTTCATCAAATTCTGGAAATGGTGGTAATA

ATTATTGTCACCCGAATAGGTCACTGCCAGAAGTTGAGGCAAGAGTGTCAGAGAAAAACG

TGCTGATAAGAATTCACTGTGAGAAAGAAAAAGGTGTGTTGATGAAGATAATACACGTGA

TAGAGAATCTTCATCTTTCTGTCATTAGTAACAGTACCTTGCTTTTTGGAACCACGAAAC

TTGACATAACCATCATAGCTGAGATGGATGATGAATTCAGGCTAAGTGTGCAGGAGCTGG

TTAGAAGCATTCGAGTTGGACTGTTGCAATTCATGAAGTACTAGTTAGTAGTTGCTACTA

GTGATGTGAATAAATGTGTGTGTTTTGTTCTCATTAATATTCACATGATAGGATGAATAT

GTGGTGAGATACATGATAGTTTGTTGAGTGGATCCGCACGAGTTAAGAGGTTTGGTCCCT

TGTTGTAATGTGTGGTTTGATTGATTGTAAAATGGATAACCTTGGAAATCAAGACACCTT

ATGGCATCACATACTCTTTTGTTTGTTTGTTATAAATAAAGTATTGCATATACTATATCT

TTGAGGTGGAGAAAATTACCATGAATGACAAAGTTATTGGACAAATTGAGTCTGTTGTAT

GTTGATGGAGTCTAGTTGGTAGAGTCATGACTCACCCCATTTATAAATGTTAAGGCCTAG

ATAAATAAATGGTGGAGACTGTTAAGTGTATCCCCTCA

>LsbHLHR18

ATCGAAAGGAAAACTTGAATTTTTGCATTCATCCATGAATAAAAGAAATCTTGAACTAAA

AGCTTAAGCTTCAAGTTGCACACAGGCTGCACATATAGTGCTGGTGTAACAGACTTAATG

AAAGAGATATTGATAGGACTAACAAACTTCCTAACTGCCTAACTAAACTTCCTAGCATAC

TTAACCAACTAATAATTACATAATGATCTCCAATACTTAATTTCAGAATTAAAATCTGAG

AAGAATCAGAAGACAACAACATGGATCTACTGTACTATGCTCAGATCAACAATACACACC

TTTATGTTTTGTGATGACAATGGGAAAAGAAAAGAAAGAAAGACTGGAAGTAATTCAAAG

ACTAGAGCAGTTTGTTCAGGCAAACTCTAATTACGAGTCAATCATCTAATTAAAATTCAA

CAAACAGTGCAACTCACTCCCGTGATATTTCAGTCATAGTCTGTTCTATGATATTACAAT

CTTGGTTCAATCACCTTATAGATTGAATAACCCCTATAGTTAGTATACATAATGGTAATA

ATACTATATATGAATAGCATACCAATTCTTCTTTTCTGGACGAAGAAAATCCAGGTTTCC

AAATGGCTCTCACAAATAGAAGACAGAGAAAACCTGCTGAATGTTGAGACGGCTCAAATC

ATTGCTAGCTGCAAATAATATATTTGATATATACACTCTAGGCTAATAGCTACTGGTACG

ACAACTTCTGTAAGCTTTCAGAGCTCCATCTTCAATTGATTTGAGTGCAGAGATGCTGAG

TTTGCCGAGGAGTCATAACTCAAAAGTGAGTTTTCCTGAGTCATGAAATTATGAGTGTCT

TCTTCCCTTCCCCAAAGTGGTTGATGGAATGCATCATCAACTTGCCACTGCTGTTGATAA

TGTTGTCTGTTACTGAGTGGGATTTCTGGCCACACAAGAGGCATAGCCATGCTAGGTAAG

ATGCCGTCCATTAAAGATGGCCCATCAGTAGCCAATAACCTGTCTAAGTTGAAATCAATT

CTTGGGTTAACTGCTGCCAGTTTCATTGATAAGATCTCCACTTGACGTTGTAGAGTTTGC

ACATGGTTGATGATTTCATCAAGAACCAATGCCGTTCCAGATATCTTGTCGCAGCCTGGG

ACCAGCTCTTGTAAAAGCTTCATGCGTGCATTAATCTTCTCTCTCCTAGCTCTTTCTGCC

AAACTATGGCTATCTGTGGCTTGACCTCGTCGAACGCGAACATGGACGTAGGGAAGCTTC

TCGCCGTCACCGGATGTTTCGTCGGCGATGCTCTTGCTCTTCTTCGAGTTTGCTTTGGCC

TTCTTCTCTCGCTCCTTTCTCTTCGCGGTTCTCTGATTCTTGTTTTCAACAGCTGGATCC

GAAACGCACTCTTGCGTCGAGCAAGGGTTGGAATCGGTCTCTTGAGGCTCGTTCTTAACA

CTTTCCAAATTAGCTCCAGACACTGCCGGAAACAGCCGCGCCTCTCTCGTTGAAATATCT

CCGGCGAACACGGAGAATTTCGCGGCACGTTCAATCAAAGCAGCGTTGGTAGGGAAGGTC

AAATTATTACCGTCGGAAGAGCTCGGAAGATAGGGTTTCTGATTCACACTGACGTGGCAG

CAAGGCAGTCTTCTGGAGTCGGTTCCGTCACAATCCGGCAAGTGAAGAAGCTCTACGGCT

TGCGTTGGAGGAAGCTCCAGTAGAGCTGTGAATGAGCTTGCGTTTTCCGGTGCCGGCGCC

GGCACCATAATTCCTTGAATTTCATCGTTAAATTGAACGGAATCAAGTGCAGTATTGTCG

TGAATGGATCCACTCAATCCTAGTGTAGCTTGTTCCATTGAAGAAGATAAAATAAACGAA

AGTGATGATTCAATTGAAGTTAAATTCTGTTCAATTTGACGAGTTTTGAACAATATGAAA

GTTTTTTATGAATGGAGAATTGGAGAGGGAGGGGAATGGTAGAAAGAGCAGTGAGAGTGG

TTTAATGGGGGTCAATCTGTTTCTCTCTCTATCTATCTCTTTGTTTCTATCTTCTTCTTC

TGTGATTCTGTGTCTGCGATTGCTTCTCAGCCATTTGAAGTACTGCGAAAGCAACGTTAC

TACGTATACGGTTACTCTACTACTGTGTTGGGTTGGGTTATACGAGTACGGTTCAATACA

GTTTCCTTGTGCGACAGTAATAGGGGACCAACACTTAAACCGGGGTCCACT

>LsbHLHR19

CTCACACACCAACCCTCTTATCTTAGAATATACTAGAATTATTGCTGATTTGAGTCATCT

TTTCCGAGTCAACATTTAAATCTACACCACACTTTTCAGCGATTAATACTTTTCTTTTGA

TTCAGAATCTAGTTACTTAACAATAATCACTTATTTGAGTCTGTTTCTGCAATACACCTT

CAACATTTTTCATTCAAGAAAACTACAAGCTTCTTTCTTTCAATGAATCTAGATATTGTA

AACATAGTTATATAGCTTGTGTTGCATAATGGTGCTGCAAAACATGAAGGAACAACTTGC

TTTGGCCGTTAGAAGTATCCAATGGAGCTATGTTATCTTCTGGTCTCAATCTGTTAACCG

ACCAGGGGTGTTGAGTTGGGGGGAAGGGTATTACAATGGAGATATTAAGACAAGGAAAAC

AAGTCAAGGAGCGGAGCTTAGTTCCGACGAAATCGGGTTACAGAGGAGTGAGCAATTGAG

AGAGCTGTTTAGGACTCTGAAACCTGTAGAAACAAGTCCTCAAACAAAAAGGCCTACTGC

AGCACTTTCACCAGAAGATCTCTCAGATACTGAGTGGTATTATTTGGTTTGCATGTCCTT

TGTATTCAACATTGGCCAAGGGTTACCAGGAAGAGCTCTAGCAAATGGCCAACCGATTTG

GCTGATCAATGCTTATTCTACTGATTGCAAAGTGTTTAGTCGCGCTCTTCTTGCAAAGAG

TGCATCCATTCAGACAGTGGTGTGTTTTCCTTTTATGAACGGGGTTATTGAGCTAGGCAC

AACTGATTTGGTATTGGAAGATCTCAGTCTCATTCAGCAGATCAAAACTTTTTTGTTGAA

TATTCAAAGTGTCGATGATCCTATTAACGTTCGAGCGACATTGAATTCGAGAAATAACGA

AGATGTTGCTTGTATGGCAGCATTTGATCATAATGACTATAATGTTGAATTAATTCCAGA

AGTTGGATATGAAATTATCAATAGAACAACCTCTCCTAGTGGTAGTTCAAATGCATTACA

AACCAATCAACTACGAGATGAAACATTCATGGTCGAAAGCTGGGGCGTTATGGAAGATGA

TTTGAGCAACTGTGTCCATAACTCCATGAATTCAAGTGACTGCATATCACAAACCATTGC

TTCTGCTCCTAAGGGTAGAGGAGAAGATTGCAACAACAATGACCAAAAAATGACCTTAGT

GGATCCTCTAAGTGAGGATTGGCACTATCAGAAAATTCTTGCAGCCCTTTCGAAAAGCAA

TGACCAGTTAACAATGGGGATGCATTTTCAAAACTTTCATCAGGAATCAAGCTTTTGTGT

TTGGAACAAAGGAGGGCCATTGGATTGCCATAGGCCAAGACAAGGAACGTCGCAAAAGTT

ATTGAAGAAGATCTTGTTCGAAGTTCCACGGATGCACATGGATGGTTTGGTTGAATCTCA

AGAAGAGAATGATTATAGAGAGGGAACAAGACTTGAGACTGAAGAAGGTATGAACCACGT

TTTGTCAGAAAGAAGGAGAAGAGCAAAACTAAACGAAAGGTTTTTAACCCTTAGATCAAT

GGTCCCTTCAAATAGTAAGGATGACAAAGTTTCTATACTAGACGATGCAATTGAATACCT

TAGAAAGCTTGAGAAAAGGATAAAAGAAATGCAAGGCCAAAGAGATCCATCAGATATAGA

GTCTAGAAATAAGAGAACACATCATGATATGATGGAGAGGACTTCTGATCATTATTATAA

CAACAAAACAAACAACGGCAAGAAACCAATGGTGAAAAAGAGGAAAATATGCGACATAGA

CGAGACGAGGAGAGTGATTTATTCAGATGGTTTGAAAGGAAGTTCTACTAGTGATCTTAG

TGTCAAAATGAGTGACAATGGAGTTGTGATTGAAATGAAGTGCCCTTGTAGATCAGGAAG

GATATTGGAAATTATGGAAGCAGTTAACAATCTCAACATAGATTTTAATTCAGTTCAATC

AACAGAAGCTGATGGGAGTCTTCATCTAATCATAAAATCTAAGTTTACAGGATCAACTAA

TGCAACAGCAAAAAGGATCAAACAAGCCCTCCAAAAAGTGGTGGCTTCAAAGTTTTGATG

GAAACAGTTTGATTCCTGCCACTACCTTGTTACACAGAGATTCTTTTTCGAAGACTTGTG

ATATATGGAAAAATAACTCTTGGTGATGTAACATAGACATTTGTAAATGGGTAGGTAGGC

TTAGTTGACATTATAGTACTGAATTAAAGTGACCAATGATTTCACATGAGACTAAAAGTC

TCATTTTGTTTATACTTTCACTACTTTGCTGGCTTTTGACGTATTAAATACCAGGCCGGG

CCATTAGATTAGTGCCAAGTCCAAACCTGCTCTCAAGAGATATTATATGGCCTATGGTCA

AAGTAATTTAATTTAATGTATACTTTTTAAAT

>LsbHLHR20

AACCTTCACTTGAAGCCTTAAGAATTTGACATAATCTACAATTTCATCCAGCATGGCAGC

TCGATCTGTCTTGTTGACACTAGGAACAAGTTCTTGCAAAGCCCTGATTCTTTCAGCTAT

TCTTTCTCTGCGCAACCTTTCAGCGATGCTGTGTGGATCTGTAGCCTGTCCTCTTCTTGC

CCGCACTCTAGGACGCATTGCTGGTGGATGTGGAGCAGTAGGAACTGTAGTCGGCATGGG

CTGCCCATGAAAAGTATTCTTAGGTCTGGTATCAACAACATCTTCCCGGAATCGTTTGCC

ACTCCCGGAAGCATCATCCGGCTTCAAGAACCCTCCCGTACCCTGGTCCAAGCTCAGGCC

CAAATGGTAGACCGGTGCATGAAATCCCGCTCCGCCAGCAAGATGAGTGGCAGCGTCGCC

GGAGTTAAGCTGCAGCATCATCGGAGAAGCTAATGAAGAGGCGTCGGTTCCGTCGGCGGA

GGTGAAATTCGGGAGGCCGAGAATTTGCTCGAGGAAATCATCGGCGGGTGTATCGGAAGG

ATGATTCGACATGACTCGGTTCGGACTCGGACGAGTTGAGAAAATGGTGTACTTTACTTT

TGGTATAGAGAGAAAATAGAGTTATAGTAATCTACTACTAGATGAAGTAGAGAAGATAAT

TTTTCAGTTTTTTTTTAATAGTTTTGGGGTTTGTTTGGTTGATTATTGTTGTGTGTGCTT

TTGTTGCTTTTTCAGCTTATTTTCTACTCTTTCTAACTTGAAAATGATGTTTGAGAGTTC

ATACTATGAAG

>LsbHLHR21

TTGCCAAAAAAAAAAAGTTCGTTTAAATGCATGACATTTTGATATTTGTTTGGAAAGAAT

GGTAGTATGGTAATATAAATGGAGAAGTGTGGTAGTTTTGTGCAGAATTCTTCAATCGTA

ATAGGAATTCATCCCACTAAACTGACCAAAGTAGAGAGATAAAAAAGTTGAGTCCAGTTT

CTTTTATCCCCTTGTGTTGAAAACGAAAAAACGAAATTAAAGGATATTACTACTTTTTTC

CCATGTTCAGTCACATCACATCCCTACAAAATGTTGCCAATTAAAGATTTCATCCTAGAA

CTTTAGTACACGAATCCACTCATTCTCGGGCATAATCATCGGACACGGTCCCAAGAAATA

GCACAACAGTATAATCTATACAAAAACCAGGGATCTTGATTCATTGCAGCCAATAACTCC

TGCAGCCCTTTTAAGAAGATACCCTGCAACCAGTAAATCTAAGCAGTAGGATAGAAGATC

AAATTACTCACAAACTATGGCAAATTGGAAACTTGAAATCTCAACAATCTGATATTTACG

GACGGCAAAGATCATTGATTACTGTAACTGTTGACGGCATCTTTCAGTTCAGCCAATGGA

AGACAAAGTGTTAAAGATGACCTAACTTAAATCATAACTAAGACACAATAAAATTGAGTA

AAATTTTCTAAGATAATTGAACATCTATAATTTATACATAGCTCCAGAGCAGCATTGCAA

TTAGGACAGGTAGTTGGCTGATCATACCAAAACTGAAACTAGAGCTCTACAAATTAACAA

GTTTAATTAGAAGTTGGTTGCTCTCCAAGCAGTTGCAATGGCCATGAATCTGCTGGTGTG

GGATATCTAGCATGTGGTTTGCTGATAACAGATGTAGGGTTTGGTATGAGATCAGTAACG

TTAGGAGCTGGAAAAGCAGCTTGAAGATCGGTACGAAAAGGAACAACCAAAACAGCAGGT

CCTTGCTGCAATGAAGGTTCTATGGTAGGCATTTGGAGACCATATCCTGCAAATTTTGTC

TGCTCTGGCAGCTCAAGCTCCAATTGAGGAGGTGCATTCAGGTCAGGTTTAGAATGAGCC

ATCCTTGCCTGTATCTCTCCTTGAAGCTTCTCAATTTGAGTTTCTAGGCTACTATTCTCT

TCCTTCAGCTCATTTTTCTCCACAGTCACATATTGAGATTCAGACAACAAAGAGACGTTC

TCCTTCTTGAGGGACTGAATCTGACAGAGCAAGTCCTTTAGCAGCTTAGAAGCTTCACAC

AATATAGAGGCCTTTCCACTGTTCGGATCATTGAGATCAAGAGCATTGGCAAGATCAAGA

AAGAGATCGTTCAAGTGTTCACGTTTCATTTTCTCTCTTTCAGCCTTGTGAATTCTCTTG

GGGACTTTCCCTTTGTTCATTTTACAAGGAGAAGACCTGTTTTTTGAAGTCTCAACCATC

GGAGCAATACTCTCTGATCCCATATCAACAAAACCAGTTATCCTGTCAACAATTTTCCAG

AAAGACGAATCGGAAGGAATAGAATTGGAATATGTTGAACGGTGAATCAAGGTTGAAGGA

TGAAGTTGATTATTGAGAGAATACTTTGGAATCGAGAAAGAAAGCGAAACAGTTATAATT

TGTGGGAAAATTAGGGTAGAAGAGAGAGAAAAGAGGATTGGTTTGTGATTAAAAAGGAAA

GGAGTGTGGCGGGAGGATACCTGTCACACGAAGGCGCGTGGCGTGGTCATGCTCATTCGC

TTCCATCATCTATCTCCAATGAGT

>LsbHLHR22

ATACGATCAAATTTTATTAGCCATCACAAAGAAAGTTAGGCTATAAATAATGAACTATAT

ATGGAAGGGGCTTAACTCAAACATAATACACAAGACCACCAAAATCAATGTTAAAAAGTA

TGCAGCAAATAAAAGACCAAAGTTTACTAACTTTACAAAAGGGAAGCCTTGAATAAAAAA

CAAGTTGTATTAGTGTGGAAGCCTCTATAATTATATATTTAGAAACTAAAATGATGCATA

TTATTTTGTTGGGGGAGTCTAAAGATCCCAACAAACTAAGACACTACATGTACAGTCAAT

CTTGTCCTTGCTAGATAATAATAATCCAGCACAGGACATAAATACCCCTTCCAAAAATTA

ATTTAAAAAAGTTACCCCAAATAATTGATAAAATTATTATAAGGAAAGAATGAATGTAAC

AGTTGAATTAACTTCTGCAGGTCTTTAGCTTCTTTACTGCTTGATCAGACTTCTCAGAGC

CGGCGACTCTAGTGCGAATCTTCCTTTGATTTTTTGAAGGATCTTCATCATCCTTAGAAA

CACACATCGATATTGGTACGTTAGGTGTGATTTTTGCTTGCTTGCCAAGTTCAATTTGCA

CTGAAATGCTAGCTTGTGACAAATCCACGCCCGAAGTCTGCAACGCGTGTGTAAGTGTAT

CCAACAACCCTTGTGAGTACACACTTGATATACTAATGGCACCTCCTTCAATAGTCAGCT

CTTTCTCTTTCAGCTTTTCATTGGTAGAAGTCATAGTCTGAGATTCTACTGAAGGTTGAT

AAATGATGTTCTCTGCATCAGATGCTAATCTATGTCTGAGTTGAGACACTGTTCCACCTG

AACCACCGATTTGGGTTGAAGGGAAGATGTTTGGTTGGGAAGAAGTAGGAATTTGAAAAG

TCTTGTTCATTATTCCAGACTGATGATCTATTGTCTTGAAGGCAGCTGTTGTGCTCAAGC

CAGATTCTACATTAGGGGTAGAGGCATGAATTGTTGGGGAGATGGGTATATTTTTCTCCT

CAATCTTTGAAGGAAACAGCAATGTTGGAGATGGATTAGACCCACTGTTTGTGCCGCGGG

GTTGAAAAGTTTCTGCTGGCCTATCATTATTTCTCCATGGCATCAATTTTTCTGGCTCGT

TATTCCAACCTTGGAATGAACCTTCATACTTATGTACCTTCTCTTGTAAAAAGTGAATAT

ACTCAATGACCTCTAACAGAAAAGATGCTTTGTCCCTCTTTTGATCACTGTGTGGAATAA

GCTCTCGTAGCATCTGAAATCTGTCATTAATTTTGCTTCTTCTCCTCTGCTCCGTTGCAG

AATGTTTTGATCTCGGTGTGTTTGGCTTTTGATCAGTACTCTTCCCATCCACTCTCACCC

TTAGTTCTCCTCTTTGAGTATCCAAGGTTTCTCTCTTGAGAAAAAACGTTCCTTCATTTT

CTAGCTCTTCATCCTGGGCACAATCCTGTGCAGATTTCATCATTTCCAAAAAACTCTGGT

TCTTCTGTCCAACTGTCTGAGATGAAGAGCGAGAGTTGAAGCTGCCATGCCGATTGTTAG

AAAAGGATTGAGATATTCGCTCCGTCGTTGTCCACGGCCCCAGCTTAGCAGCAGAATCTG

CCACTCCTATAATGGGTTTCTCTCCAACATTATTCTCCTTGTCTGTCTTTCCCTTTTTAA

TCTCAGACTCTTCCCACAATGTGAAACCGCTTGAAGTATGTGAACTACAGTTAGAGTCAT

CGTTTCTATCCGCACTAGTTGCTTGATGCACAGTGAAAAGTGATGCTTCTGGCTTTGGAA

CTCTTTGAGTATTATTGACGTAGGAATTAATGTGGCTAATAGTGTACGTCCCAATTCCTC

CAGGTAAGAGGTGCTCAACAGAAGCTGGTGTGGGTAAAGGCGGTTGCTTCTGAACCGCCG

ATGAGATTTCATCTGTGGCTTCTTCTTTGGCACTGGATTTTGTTTCTACCTGCTCTAGTG

GCCGCAAGAAATCATGGGTCTTGAGGTAACTTGCTTGAGCAGAAGAAGATCTTGGATCTT

GTTCGGCAGTCGAATTACTGTATAGCGACAGAAAATCGTGCGTCGGTTTCCTCCCTGCTT

CTTCGGCTCCACATGGACGAGATTGAGGAAGCTCCATCAAAACCTGGATCTCACGCTTCT

TCTCTGGTACTTAATTCAACGGTATTTCGGAATTCGGTCTTCTCTAAGTTCAAATCTCAT

AGAACACAATTCTGTTACTGCTTAGTAAAACTGAATTATACTTGAATTCCGAAACCACAA

TAGAGATGGAGAATTTGCTGCTTCGATCAGTATATATAAAATTCTCAGATCCGAATAAAA

TAAAAACCACAACTCTCTTGTTAATCTTTCTCTCCTCGATAGGTTGAAGATCGGAGACGC

ATTCGTTCAGTTAACTATATTTTCAAGCACGACGTTCTTAATCGCCAATCACAAGCTTAC

AGGATCGAGTCTCCGTTCTAAATGATCTACGAATTGTCGTTTATACGTATCTAATCAAAC

TTTCATGAATCACGAAGGTTACTGAACAAATCAATTTGGCTCCCAAAATCATGAACCACA

AAGCTTCCTGTCAACGATTTTACAGAATCGCAATGATAACCGAGGAATGAGCACGATTTG

GAAGAGTAGTGAAGGATAGAAACGTTCTAAGAGCGCTTTAACTACTTTCTCTATGGATTC

CTTTGTTACTCTGAAAACGAGCACTGCAGCGAAAGATGAGGTCTCAGGTTAGACCCTTTT

TCTTTCTGCTGTATCTGTGAAGTTTGAGAACTGATAATTGAGATAAAGATGTTTTTATAT

AGTTTCTCTTTGCTCTGTGTTTTCGCCATCCATTTTTATCTACTTTCTATTGTT

>LsbHLHR23

TTGTCTTTGAAGAGATTGAACATAATTGATAATTTCATCCAGCATTCCAGCTTTTCCTGT

AATTTTGTTGCATCCTGGTACTAAATCTTGCAAATACTTCATTCTTTCACTGATCTTTTC

CCTTCTAACTCTTTCGGCTAAGCTATGGCTATCGGTGGCTTGGCCGCGACGCGCTCGAAC

ATGAATGTAGTCAAGTTTTTGAGGCTCGGAAGCTTTTGAATTTCCCTTTGAGTTTGAACT

ACCTTCTCCTGCATAGTTTTCTTTGTTATTCTTGTTGCTATTTATATTCTTTTTGGTGCT

AGGGTACCCTGTGATTTTCGATTCACCATCTTCAGCACCTAGCTTAATCCTCTTTTCTTT

GTTCTCAATTTCATCAACAACCTTGAGCTTATGATTATGATGTGCCTTTTCAGATTTCCT

CTTCTTAGAACAATTCTCTTTCTCGATTGTAGCATCAATGAGAACAGGTGGAGAGCTGAA

GGTTCTTGAAATTGCAGATGGAATTTGAAGAGGCACTAAATCTTGAGAATTCTGAACTTG

TTGAACAGAAGAACAAAACACAGCACTGTTGAAGTAACCTTGACCTTGCTGTTGTTGTTG

ATATTGTTGCCACTTGATCGTTTCGCTTTGTCTTTCCTGCACTGTTATGTCAGATGAACA

ACTCTCAGTAGGTAAATTCCCAGAAGCATTCAAACAGTGCAACATGTTGAATTAAGCTGT

TTTTATATTTGATTATTGATTAGTGTTAGTGTATTGAGGTCAATTTTGTTGTTGAAAGAG

AAGATGAAATATATGTAGTGCAGAAAAATGGGAGTAGGAGGAGAAGGTGATGATAGAGTG

TAAGCTATTTAAGGTTGAAATGGGAGAATTGGTGGGGCATATATAAAGGCGTAT

>LsbHLHR24

AGATTCGAAATGAAAAAAAGAATTAAAAAAATGCAAATTGTGGAAGAAGAAAACGGCGGT

GCCAAAAGTGCGTGCTTATCTATCTTATCCCAAACGCAACAACACCGACTACAAATACAA

AAATAACCTTTTGTCCAACCGAGAGAACAAAAACCAAAACACAACCATTTAATTATCATT

TAATTATTATTACTATACTCAGCGATGCAACCTACTCCCGGCCGCGGCGGTGGCGGTGGC

GTGAAACGTTTCCGGTCAACTCCGGCGAGCTGGATAGAATCTTTTAATCTCAAAGAAGAA

GAGGAAGACGAAATCCAACAACAAGACAATTTCAGTTTTACTCAACTGCTTTCCAATAAC

GCTGCTGCTGGTCCTTCAACTCCCGATTCTCATCCATATTTACCTGATTATTACCACTAC

TCTTCACCGCATACTCCCACCGCCGGCAATGTTAGTAACAATCCTTTCACACAGGGGGTT

GAGGAGAGTGATAATGCATTGGATCTAAAAATAGACAAAATTTTAGAGGATTCGGTTCCT

TGCAAGATTCGGGCAAAGCGCGGCTGTGCTACTCATCCAAGGAGTATTGCTGAAAGGGTT

CGGAGGACTCGGATTAGCGACCGCATAAGAAAACTTCAAGAACTTGTGCCAAACATGGAT

AAGCAAACAAACACTGCAGAGATGTTAGATGAGGCAGTAGCTTATGTCAAGTTTCTACAA

AATCAAATTGAGGAACTATCAGAACACCAACAGAGGTGTACATGCACGATTCACGAGTAA

ATTCGTGATCTTGTCGTAAAGTACTTGGCTGTTTACATATTCTCCTTTGAGTTATCAGAT

TTTTTGATCTTCTAATGAAGATTAACCATGTCTCAGAAGATTAAGGCTGTGTCACATTTT

TGTGCAACAAGTACATATTGGAGGGTGCTATTATCAGTTTTATGAGTTCTTTCTATTGTG

TTATAATTGTATATTTATAGTGTTTTATAATAACTTATACACAAATCTGCTAAACTTACT

AGCTTATTGAGCTTCTTTTTGCTGAGATATAATTAATCATATGTGAGATTCTTCACATGA

GCAAAAGATTATGTGAACCGGCTCTTTAGATGATAGAATTTTGCATTAGGGTTTTTGTAA

TTTTTGTTTTATTTTACAATGTCTTCTCTC

>LsbHLHR25

AAACAAAAGAAAAGAAAAACAAAGCAATAAAGAAAGCAAACAAAGACAAAAGGTTCTCAA

AACTCTATGATTATTATTCAAACTCTTTCAAAAATAGGTCCAACCAATGGTGTGGTGCTC

TCCCTACTTAATCAACCTCAATAAGACCTCTCAAAATTTCTAAGAGAGAGAGAGAGAGAC

TAATTTCTCAATTTTATTTTCTTCTTCTTTTTTGTAATAAAATTCTTAAGGGTTGAAGAA

GCTTTAGGCATGAATAGAGGTGTTGTACAAAGCTCACCAGTTCAACAAATGATGGCTAGT

AACCCTAATTGGTGGAACATCAATAACATGAGGCCTCCATCTCCACAACCTCCTCCTTTC

TTTTCTAATCCTAATTCTAACTTTCTTATCCCTAATTTTACACCAAATTCTTCTTTTTCA

TCTTCTCTTCCTTTTCCTTCTTGGCATGATAATCATCAAGACCTTCCTGAATCTTGGAGC

CAACTACTCATGAGTGGAATTGTTGGTGAGGAAGATAAGATTGGAATGAGCCAATTTCAA

AATCAAATGCTAATAACTCAAGCTCCAAATTCTTCTCATGTTGATGTGAAACAAGAAAGC

TCAGGAAATAGCTATGCATATGGACATGTAAATGAAGAACTTAATTCATGTGTCACAAGT

TTCAACACCAACAATATGTTAGATTTCTCTAACAATAATAATAATACAGATTTGAGGCAT

CTACCACCAGATCTATCTTCTGAGTGCAACAGCACTGCATCTGGTGGGGCAATGAAAAAA

GCTAGGGTTCAACAAGCTACAACTCAATCAACCTTCAAGGTTAGGAAGGAGAAGTTAGGT

GACAGAATTACTGTCCTTCATCAGCTTGTTTCCCCATTTGGAAAGACTGACACAGCCTCT

GTCTTGTTAGAAGCTATTGGTTATATCAGATTCCTTCAGACACAAATTGAGGCTCTTAGC

TTACCATACTTGAGCAATGGATCTGGAAACACAAGACAATCACATTCAGTTCAAGGAGAT

AAGAATTGTTTATTTCCTGAAGATCCTGGTCAGCTGCTACATGAAAATAGCTTGAAGAGA

AAAGCAGCTGAAGAGGTTTCTCAAGAAGAAGCAAAGAAGGACCTACAAAGTAGAGGGCTG

TGTCTTGTTCCAGTGTCATGCACACTTCAAGTTGGGAGTGACAATGGAGCTGATTATTGG

GCACCTGCCCTAGGAGGAGCCTTTCGGTAGAAACCAAAATTTAAATTATTGGGGCAGCAT

ATAGATATTAATTAACATATGCTAGCATGACTATAGGTAATTAAATTCATGGAAAATGAT

ATAACATCATGAGCAGTCAATCTGCTCAAAAAAATTAAGGCTGATTGCTCATGATGCTAT

ACTTCATATATAATTCCTGAATAAATTGTGTGCTAGCTAGCCATGATGATGAACTAGTAT

ATTTGAAAGCCCTTATTAAGACTTTTTGATTGATTCATGTGTAATCAAGGTTTTATTTAG

TTTTATTGGCAATTTATATTTAAGAGGTTGGTATTAAGGTCTATTTTATTATGTAAATGC

TATATTTGTATTAATCATTTGAATGCTACCCCTTTTTTTTATCAACATTAAAAAATAGTA

TCATTATGATTGG

>LsbHLHR26

TTACCTTCAAATTCACTCGGTCGTTATTCTATCGAAATTGGGAATACAGAGACAACCGTA

CGACAGCACAAACCGTCGTTTGCTCTCATCCAAAACGACCCCGCTTCCAACTTTCGTTTC

ACTTTTACCTTTTTACGCTCAAACAATTAGAATCTGAATTCACAAAACAAGAATGGGAAT

TGGAAATTTTTGAAGATAAGCATCGACAATAGATAAAGGGACAAATTATCAAGCTCTGGT

TTTTGGAGGCGACAAAGATTACTGCTTGAAGAGTTGCATTCTGAAGTTGAACCGATGGAG

TGTACACCTGCTAGAAAGACACAGAAAGCTGACCGAGAAAAGTTAAGGAGGGATCGACTC

AATGAACAGTTTGTAGAACTGGGTAACATTTTAGACCCTGATAGACCTAAAAATGATAAA

GCAACCATTCTGGGCGATACAGTTCAATTGCTGAAGGACCTTACTTCTCAAGTTAGTAAA

CTAAAAGATGAATATACTATGCTAAATGAAGAATCTCGTGAGTTGTCTCAGGAGAAAAAT

GATCTCAGAGAAGAAAAGGCTTCTCTTAAATCAGATATTGAGAATTTGAATAACCAGTAT

CAGCTGCAACTCAGGACTATGTATCCATGGCCTACAATGGATCATTCAGTAATGATGGCT

CCACCATCATACCCGTATCCAGTTCCAATGCCGGTACCTCCTGGTTCAATTCCCATGCAG

CCATACCCCTACTATGCTAACCAACATCCTGCAATCATCTCTAATCCCTGTTCGACATAT

GTTCCGTTTTTAGCCCCCAACACAATTGTTGAACAGCAATCCACACAGTACGTATCCCCG

CCTCTTCATCCAGGTAGCCGGTCCCACATATCAGGAAAACAGGAATCCAAAAACAAATCA

TCGAGGGAAAGCAGAGCTGAAAGAAATGCAGATTCTAATGATGTCGCCACAGACCTTGAG

TTGAAGACTCCTGGGTCTTCTGCAGATCAGGATTTATCATCTGCACAGAAAAGATCTAAT

AAGTCTCCTTCGAGGAAGGAAAACAGTTACACAGAAGGTAGTTCGTTAGGCAAGTGTTCT

TCATCTCACAGTGTTCAGGATAGCTCATCAAGTAGTGTAGTTGGAAGCAGAAAGGCTAGT

GAATGAGAAAGGACATAATGTTAGACAAGTGATTATGCCTTTTGAAACTAATATGATGAG

GGTTTTCACTCTGCTTTTGCTGCAACTATTCTAGAGGTATATGCCACATTTATTCTTGTC

CAGAAAAGAAAGCCGCTTCTGTTCTCTCTTCCTGACTAGGAATTTATATTTTGAGTACTG

TGTAATTTAATCATAAACTACTTTTCCTTGTGATGGAAAAGCTTCACAGGTTCAATTGTT

TATATTCCTACTTGTGAAAACATCAGTCAGATCTAAATAGTGGTATTAGTATGTTATAAA

AAATGAATAAGAATTGTTTTCAAGTTTATTGAATTTGAATGATTCAAGTTAAGAAT

>LsbHLHR27

ATTGATATCTACCCATCTACTTCTTGGCATTTTATTTACCACATAGTCTTAATTTTACAC

GAGGAATTATAAATGCAGGCTTGTGTTCTCTCTCTCTCTTTCTTGTGGCAGTAAATGACA

GGTTCCATAGCAAGAAAACAGAAAGAGAGTTCCCAAAATCAACAACGTTTTTATGTTTAA

AACCTTGAAACATGGTGCCAAAAGTAGCTTGGAATCACAAATAGGTATTATCTCTCTCTT

CTCACTTTTTCTTCTTTTTTCCTTTTCTCTTGACTATTCTTCCTTCTGCACATTCTGACC

TAAGTTTCGAAATTGGGGCCATTTATGATACTCAATTCAATGCTCTCTTTTCATCATTGA

CTGCATATTTTCCCTTCACCCACCAGTTTCAACAACCTTTTTTTACCTTCTTCTTCTTCT

CTCTCTGCTGCCAAACTCGTGTGACATGACATCTAGTACTTCATTTTTTGGAAACTTGAA

GTAAATTAAAAAAAAATTCAATTTTTTGGTGTTTATGCTACTTTTCAAGAGCTGGGTATT

TCTCATTTGTTCAGTTTGGTTTTGAGATTTTGATTTCTTAGCTATAGGTGGAATAGTGGA

TACTTTGGTTTTGGTATATACATGTTAAAGAAATGAGTGAGAGAGAAGAGTTTGAGGTAG

ATGGAAAAAAGGGTCCTTTGGATTGGAGGTTTGTGAGTGGCAACCTTGCAAATTCATCTA

TGGGTTTGGTTTCCATGGAGAATTCTATGATGGGTTGTTCTCCTTCATGTTCTAATTCAA

TGGTGGATTCATATGGTTCTCACTTTTTGGACCTTCTTCCTAGTTCTGAAAGCTTTGGTT

TTTGTGATGTTAATGGCCACAGCAACGGAAATGGAAAAGATGGGCTATGTTTTGCAAGAG

TTGGTTGTGATGATAGAACACTAGGGTTTGGATGGAATGTTGCTAGTTCTATGATGAAGA

GGGATGGTGTTTTAACAAATGGACATGAGATGTTTCCTCAAAGTTTGTCTCAGTTTCCAA

CTGATTCTGGATTTATTGATGCTGCACAAATGCCGTGCTTTAATGCCGGCGGTTTCGGAG

ATATGGTTAACTCATGTAGGATTCCTCAATCTACGGCTCTACACGTGTCTCGGCCTGTTG

AGTATCCTGGTAGTGATGGAATTCCACTCCAAAATGATGGAAGAAGTGATTGTCCTGTAA

TGTCTCTAGATGAAGGGAAGCAAGCACTTGGAGGGTCTTGTGATGAAGTTGATAGGGCTG

AATCAACCGGTGAGGGCGATGATGGTGTTGCTGTTGGTAGCCACGATGGTTCCCAAATGT

TGGACTGCACAAGTGGAGAGCCTTCTATTAAAGGTCTAAACTCAAAGAAAAGGAAAAGAA

GTAGGCAGGATGGTGATAGTGATAATGCCGTTGGAACTCCTGAGTTACCAAGGGAAACTG

CAAAGGAGAGTAGACAGAAGGGAGAGCAGCAACCAAATTCAAAAGCGAAGGCTTCTGGGA

AAAATGCCAAACAGGGGTCTCAAGCTTCTGATTCTGCTAATGAGGGATATGTACATGTTA

GGGCTCGCCGTGGTCAAGCAACAAATAGTCATAGCCTTGCGGAGAGAGTGAGGAGAGAAA

AGATCAGTGAAAGGATGAAGTTTCTTCAAGATCTTGTACCGGGATGTAGCAAGGTCACAG

GCAAGGCATTGATGCTAGATGAAATCATTAACTATGTACAGTCTCTTCAACAACAAGTTG

AGTTTTTATCAATGAAGCTCGCAACAGTGAATCCACATGTGGATTTTAACGTGGAACGTT

TGCTTCCTAAAGATATTCTTCAACATCGACCTGTTCCGTCATCCACACTTGGGTTTCTCG

CAGAAATGCCAATGGCTTTTCCTCCATTATTACATCCATCTCAACAAGGACTGGTCCGTT

CGTCTCTACCTAACATGGCTAATTCATCAGATATACTCGGGCGGACCGTGGAACCACAAT

TCACACCCTTGACTGGAGAATTTAAAGAGCCAGATCAGGTTAATCCATTGCAAGAAGCCA

AGCACTGAAATTACCTCAACACTAATGCTTTATCAATTTCAGTTATATACTTAAAGTTTA

GTTTCTACATCATGCTAGTAGTAATTAGAAATGATGGTTGGAGTCAATGGACAGTGATGT

TAGTATTTAGTGTGCTATATTGCAGTTATTGCAATATTTGAAAATTTCACCGAAGTTATT

TTGTTTCTACTAGGTTCACTTGATTGTTTTATAAACTTATTATGTGTACATATTTGATAC

AATTTGTAATATAGGTGCATGAAATGTGGGAGGATGAACTTCATAATGCACTTGAGATTA

GATTTACGACAATTTCTCCCATTTGAAATGATTGTAAAAGGTAAAAAGAAAAATCAAT

>LsbHLHR28

GGCATAATGACTTGACGTCATCCTACCTTCCTTCTCTTTTCTTCCTCTCTTGTCTCAAAT

CTATTCTTTCTCATACTCAACAAACATAAGGACTTGAAGTTTGAATTTGAATTCATCTAA

ATTTGAATTTCATACCACCTTTATTTCCTTTCAAAGTTTTCATAACTTTCTTGTCTCAAA

ATCTAATCTTGATCACAGAACAGATAGAATTCAATAAAGATTCTCTCAGGTAATTGAAGA

AACAAGATGAGTGTTATGTATGAAGAGATAAGAAGAAACCAAGAAATAGATTCAAAACAA

CACTATCACCAACAGAACAATTCAACACTATCAACTCCATTGTTTACAAACCTTATTGAC

AACACTAGCAATCATCATCATGGCTATATCAACAATGAAGAATCTTTTACAACTGAGAAT

TATCTTCCATCCACAAGCTCAGAAATGGACACCATGTTATCCAAGTTGATGTCATCCAAC

AATGGATGGAACAATTGTGAAGAGCCTTTGGAAGAGTTTGATGTGAAGAATGTGAAGAAA

GAAGTAGGTGAATCTGTTGGACAGAATGGTGATTATAGTTATGGTGGATCTGAGTTGATT

TATCAAGGCTTTTCAAGTGGTTCTGGTAATGGTTTTTATGGATCTTTTGGTGGAGGAAAC

TTTAGGGATTCTGAGGATTGTGCTCAAGCTAAAATGGGTGTGAGGAGTTGCAATAATCTT

GTTAGGCAAAAGAGTTCACCTGCTGGTTTTTTCTCTCATGAAAATGGTCTTACAACATTG

AGAGAAGACACAAATGGACATGAAACTCCACATGGAACACTTAACTTCTCATCTATGCCA

TCTACATGCTTGAAAAGAATGCCACAAATTGCTGAAAATAGAATCCAAAGTTTAGAAGCA

AATGGTGACTCCAAAACTCAACATATGCCTAGCTTCACAAATGAATTTTGGGACAATTCA

TCATTCAATGCTCAAAAAACAGAAACTGAAGATGAAATCATGTTTTCTACTTCAAATGGC

TTGGAATCTCATGAAGCTGATTTTTGCTATCAAAATCTTGGTTTGACTCATCATTTGAGT

TTGCCTAGTTCTTCAACTAAGATAACATCATCCATAGAAAAGTTTCTTCAAATTCAAGAT

TCTGTTCCTTGTAAAATTCGAGCGAAAAGAGGGTTTGCAACTCATCCGAGAAGCATTGCA

GAGAGGGTAAGAAGAACGCGAATTAGTGATAGAATCAAGAAATTGCAAGGTCTTTTCCCA

AAATCGGATAAGCAAACAAGTACAGCAGATATGTTGGATTTGGCAGTTGAGTACATTAAA

GACTTGCAGGAACAGGTTCAGATACTCACAGATTGTAAAGAAAAATGCAAATGTGCAAGT

CATGAGAAGCAACACTCTAGACATTGTAGCTGATTTAATCCCTTTAGATAGCAAAAGGGA

GCAGAATTTTTGTGTAAATATGAAAAAATAATATGTAATGAGTGTGCCAAAGTTGGCAAT

TGTAATTATAGAAAATAATGCTGATATTTGCCTAGTATGAGTTTAGGTTGATGAGATTTC

AATTTTATTTTATTTTTAAATTTTTGTCTTGTGATTTTTCTTTTAGTTTAATTATAGGAT

CACTATGTAATATCATGTTTATAGTAGGGGTGTTTCTTTGTGGCTCTTCATTTTACATGT

GAAAAGTTTGATTGCATTACATTCTAATCTTCTAGAATATGTGATA

>LsbHLHR29

CGTGAATCTAGTTGGGAGAGTTCCTTCCCATTTTTCATCATGTATTGATTAATGCTTTTT

TTTCCTTTCCATAACAAAGACATCTAGATAACATCACTGATGCAAATGGCTGTGACTGAG

AAAACAATATTGAGGCATTTATGATTAATCAGAGCCCAGCATGTCATGTTTGTCTGGATT

TCTTATTGTCAAACAGGTGTTGGTAGCAGCATTCACTGAGTACAACATTAATGGCTGAAT

TTGAATGCTCAGAGCTGTCTCCCCTCAAAACACACTACAACTTGTGTGTTACTATCAAAT

TTCATTCCCTCAATAGTTCTCTTCTTAGGTCAAAAACTTACTTGTGTAAGATTACACTAT

ATATCATCAGCAAGAAATACTGTTTTTTTCAAACACAAATTTCCATAATTGCACACTGTA

AACCAAAACATGGCCAGCCCAACAAAAAATGCTATAGAATTTCGCCTGGTTGAACAATAT

TAGTGAGATCCTGCCTTTACATTCTTCAATTATATTTAGTGAGCAAACCTATTTCAGTAC

AATAGAGACTTCCACTTCTAAACATATGATACATATATTCACGCTTCAGACGAAAGGAAT

GTTCATTAATACTTTCCCTAACATCTACATCCAGTCATCTGTAGGGTACCACTTACCCCG

AAGGCCTCGGCATTAACAAGTTTGGCAAGTCATATGTAAAACTTCGTCTGTAGTTATTTC

CATCTGACTGGAACAAAAGCCGAAATAAGCGTTGTAGACTTAACATCAAAGTTCAACTTT

CATCTGATTTGATGCATTAGTACCATCCACATCTTCGTTACTTGTGGGAGCAGTTGTTGC

AAAGCTCATTTGTATAACATTGTGAAGCTCATCCTCCCACATATCAGGCATCTGATTTGG

CTCCTTGAACCCTCCACTCAGTTGTGGATGAATAGCTCTACGAAGGATATCGGACGGGTT

TGTCATGTTTGGAATAACTGACTGAATCAGCCCTGGTTGAGATTGATGTAATGGAGGAAA

ATTCATAGACATCTCTAAAGGAAACCCTAGTGCGGATGAAGGACCAGGCCGTTGGTGAAG

AATATCTTTAGCAAGAAGGCCTTCGATATTAAAATCCAGTCGCGGATTTACTGTTGCAAG

TTTCATAGATAAAAACTCAACTTGTCGTTGAAGTGACTGTACATAGTTGATGATTTCGTC

CAGCATTACTGCCTTGCCAGTGACCTTGCTACATCCAGGCACGAGTTCCTGAAGAAACTT

CATCCTCTCACTTATCTTTTCCCTCCTCACCCTTTCTGCAAGGCTATGGCTGTTTGTTGC

TTGACCCCGCCGAGCCCTAACGTGTATGTATTCCTCCTTAGGTGGATCAGAAGTTTGAGA

GCCCTGTTTGGCATTCTTCCCAGATACCTTGGTGGTTGATGTTGGTTGTTGGTCTCCCTT

CGGTTGATTCTCAGGGTTGTCCTTTGCTCCTTCACTTTGTAGTTCTTGAGCTTCATTAAC

TTTATTGTTATCAGCATCCTGTCCACTTCTTTTTCTTTTCTTCGAGTTTAGTCCTTTAAT

AGAAGGCTCCCCGCTAGTAGTACCCTCTAACATTGGGGAACCACCCTGTCTGCCACCACC

ACCATCATCATCTCCCGACTCACCTCTATCAGAATCGTTAGCATTCCGAACAAGAGTCTG

CTTCCCTTCATCTTGAGACATGGTGCGACTCTCGCTTTTATCATTCATTAAAGGGCTTCC

TCTAGCAACCAATTGCTCAATAGACGGAGACACGCCTTTTGTAGCTGCTTCAACTACATT

AGGATCACTACTCTCTTGTGATTGTCCCCCAGTTGCTATTTTCAATCCAACACCTGCCGC

AAGAGCATCCCTACCTCCATGAACCGCACCACCAACATACATAGCCATGGATTGAGGAAT

CCCATACGAGTTCATCGCATCACCAAAATTCCCTCCTCCAAAGCAAGATAACCTTGCAGC

CCTTTCAATAAAACCAGAATCAGTTGGAAACTGAGATAAACTCTGAGGAAAAAACCCTTG

TCCATTCGGTAAAAAACCATCCCCTTTCAACATAGAATTAGCCTGGTTCCAACCTATTTC

ACCATGACCACCTCTTCCAAAACCAAACCCATCTTTTCTAATTCCAACGGCGTTCGAAGA

ACTCCCATTATGTTGAATATTCATCTCACAAAATCCCAAATTTTGAGAATTTGCTGAATG

GTCCCAAAAGTTAGGACTATAAGAATCCACCATTGAGGCAGAGGAACATGAAGAAGAACC

AACCAAATCCCCTCTATTGATTTTCATAGAATCACCAATTGAAACCAAACCAACAGATGA

ATTCACAAGATTACCACCCCCAAATCTCCAATCAGGAGGCATACCAGAAGAGTAACTCAT

AGGATCCCCATTTCTATCCACCTCAAACTTTTCTTTGTCACTCATATCAAAACTAAAATG

AGAGAAAATTTGATCGAACACAAAGGGTACTTTCAGGTTTTAATAGTAGCCAAAGCAAAT

AGTAGAACATGTACATGTACAGCACAAAACTATGATGAAAAGCTTGAAGCAAGGGAAAAA

AGGGTGAGCGAGACAAAGAGAAAGAGGATGAAATAAATGATGAAACAAAAAGCAAGTACG

AAAAAGATGTACCAAAGGTTCACACGAGTTTAGCAGCTACCATTTCCAAAGCTTGAAAGT

ACCAAAAATTGATAAAGAAGGACCCCCCAGAAGACACAAAAAGAGTGAAAAATGCAAAAA

AAAAAACATTGATTTCAGGATAATAAATAAGGTCATTGAAGCCCTAGTCAGAGAGAGTTG

AAGATAATAAAAACCAAAACCAAACAAAACAAACCCTTGGAGAGAGAGAGAGAGAG

>LsbHLHR30

TATGAAAGATCTGAATCAAGCATTTGAATGTATAATGTTAAGATATATAAAAAAAACTAT

GCCATTAGATTGGACATGTTGTGGATGTACAAATGAGTGGCGAATCACAAGATACCAAAA

TTTGTGCAATTTTCAAATTTCGGAAATTAGAATTCCCATCACTATTTTGACGGATCAACC

TCAATGACTCCCAAATATCCTCTGTAGTAATCTGGATTGAACTTGCAAAGATGAGATACA

AGAGATTTTGGACTCACATTTCGGAAAACCTGATTCTTCTACTAGCCACGATCAATGCGT

AGTTTCATCAATCATAACCAATCACAAAGTGATATTTCCAGGATTAGCAGCTCCACTGTC

ATCAACCACCGAGGGATGACCACCATTATGCGCCAAAGCCGACTGATTAGGGATCTGGTT

TGTAAGATCACGGCCACCTGTGCTTTGTTGAATTTGAGAGTCCACATTCCGCATTGGATC

TTTTGAGCCCGACACTGAAGCCGGATTCACGGTTTCAGCATTTCCTGTGGATCCATAGGC

AGATGGTCCAGGAGCTGAAGAGTTCAAAACAGGCCCTCCTGAAAAAGGAAACACAGGAGC

TCGCGGCATTGGCATGGAAATTCCCTGACCTGGAAGCCCAAACCCTTGAGGATTCGATCT

TGCCATCCCATTTATAGCAGAAGGTCCAGACATAGGGATGTGAGTTCCTTGCATCTGAGG

CATCATTGAAAATCTAGAAGATCCGCCGTTCATGTCAGGTATTCCCATTCCATAACCCAT

ACCTAACCCCATATGCATGCCAATGCCCATAGGCGAAAATGCAGCCATGTGTGGTGCATG

CATGTGCTGCATTCCAGGAGGGAACATCATTTGAGGCATATATAAACCAGCACCCATTGA

CATCATTTGAACTTGGAGCTGAAGTGTTTTTAGATACTCAATTGCCTCATCGAGCATAGA

AGCTTTATCCACCTTATTGCAGTTAGGTATGAGTTCTTGTAATGCACGCATCTTCTCATT

TATCCTGTCTCTTCGCCTCCTTTCAGATAGATTATGCACTTCAGCAGCACGGCTTCTTTT

GGAACCTGCAACTCCTCGTCCGGAACCTCCCTTCTTAACACCGACTGATTCATCCTCAAC

ATCTTCACTATGGCATTCAGAGTCATCGGTGTCTCTACTTTTCCGTTTCAAATCTCGATT

TGGATCATCCGAGCCTCTGTCTGCACCATTGCCAGAACAAACTGAAGAAGAAGCTACTGC

CACCGCCTCCATACCTTTTTCAGCAGCTGTTTGTACTTTAGCACTACCGTCGCCAAGAAC

TGGATTGGAAGGTTGATCTTTTTTAATTCCACTCTCTTTGCAAACCGGCTCAGATTGTTT

AGAAGCTACAGCATTCGTTTCAAGAGATTTCGTCTGCAAAGGTTTCAAATCAGCCTTGGA

TTGCACTGCCACCTTCTGACAGTGAATCTCTGGTTCCTTTGAACACTCACCGCTTGAATT

AACAAGAGTTGATTCAGGAGGATTGCTGCTAGTTGAAGCTTCACCTCTATTCTTAATTCC

CATACTATCCGCTCTTGCTGAAGCTGAAACCAAACCAGACTTCAATCCAATGTGCTGAAG

ATTAGCTCTCACAATAGCAGCAGGCCTTGCGAAATGGGAAAAGTTCATTACATTGGAACC

ATTGCCGGACATTTTAGGATCTTGCTTCTGCGCCTTTAAGTTAGAAAAATCACCCGAGGA

AGATGGAATGCGAGTAATTTCACCGTAAGGCACATCCTGGTTACCGTTACTTGGATTATT

GTTTTCAGTTCTATCCGATTCCTTGGATCTAACAGTCACAAATGATGTTTGACATTGATT

CGATGATGGTGGATATAACTGATTCGTGCTTGCTTTATTTCTTGCAGTCTCGACCTGTTC

AGCCGAAGAACCCTTAGAAACATTCATCGGTTCTGCAGAATCTTTATTCGAGTCCCTAAA

TATCTGACTTCCATTACTCCTTTTATCGAATAAAGTAAAACTATTCGATGCAGGGATATC

ACTATGTAGTTCTTGTAAGAAATCAGAACCATATTCAGTTTGCAAAGAACCATCCATCGA

ATAATCCAACCAAGGCATCATTTCATCTACTTCGCGAGATGGCACTGATATCGGAATCTC

ATTCAAACCAGACTCTAAATCACCAAACTTTCCTATCTTTGGATTGTTACCATATCCTCC

TCCTCCAACGTCTTTATCTCGACCTCTCGGGCTATGTGACGGTAAGCAGTGAGATGGCAA

ACTCCTGCAAGACGGACTACTCTTTCTACCTCTACTTGATTGGCCTTGGCTCGAAATCTG

ACCACTCTCCCATACCAGCTCAAAAAAATCATTCTCAGGTGAGGAAGGTTGATCAGCTAC

ACTTGTGGTGTTAGTCTCATTCCCAAGGTTCTCTCTAGCCAAACGGTATAACTCATAAAG

AGGCATGATGATGAACAAATCAACGGATCAATCAACAGAACCAAAACTCAGACCACAAAA

ACACCTCAACTCAAAAAACCAAAACTCTTAGTCTTTGCATTGCTTCCAACATATTCTTCT

GCAAAAACATAGAAAACTTTGGAATCCAAATGAAGTTTGATGACAAAGCAGAAAAACCTT

GTCAAGTATATAAAGTATGTGACTTTAATCCGATCTTAATTTTATAATCTTAATTTCTTT

CCCTAATCAGCATTTAAGTCGGCGGTGACAAAATCCAAAATCCCAGTTGAGTTTTCCACC

AAATACAGTGAGATCAAGGAAAACCCAATTATCTTTTCTCAACTGGGTACTCAAAAAGTT

TCAAAATTTCATGAAAACTTAACCTCATTCACAATAAAAAGCTTTTTTTATTCCTATTCC

AATCTTTGAGTGGGAGTTATCATTAACATTCCCAATTCTATCAAAAAAGCAACCTTTGAA

GCTCGAAGAAAGAGAAAAATCCAAACTTTAAGACCCAAATAGACGCAGAAGTCGGCGAAA

GATTTGCAGAGTTTGCAGAAAACCAATGCAGCAACACACGCACACAACTGAGAAGACAGA

AACAGAGGAAAAAAATAGAGAGAGAAATCAAGGTTTCCTTTTCTAAACTATGGTTAATGT

TATGGTTAAGT

>LsbHLHR31

CTCTCACGTCATTTTTACTCTTTTTAAAACCGTAGCGCACTCACTCACCATTCTCTCTCA

AAAGCAAAACCAAACCCTGAAAAAGCTTCAACCACTGCCAAACTAGTACTTATAAAACCA

CTATCTCTTTATCTCTCTACACAAAACATTATCGGAAAACGGAACCCTCAAATGCGGCAA

TACTAGTCAAAACTGTTATACTCCGACAACTGACTCTGTGAACGAGAATCTTCCACATGG

CCGGTAATTGTTCGGAGGGATTGGGGGATGATTTTTTCGAACAGATCTTAGCAGTGCCTG

AAAGTGGAGTTGGTTATGGGAGGAATACTACAGGCATGGATCATGTTGGAGGTGTGTTGC

AACTCGGGTCCACACCTGGAATAATGCCGTTGGGGTTGAATTTGGAGCAAACTCATCATG

GGTTTCTCAGGCATCAAGATGCTGCAACAAGGTTCGTTGACAACATTGTTGATGTTGAAA

CCAGTATTACCAACGGTAACAACAACAACCATCATCATCTTCGTCTTCATGATATCAATA

ACAATAATAATACTTCTTCCTCACCCTCTTCTACTCCCGGAATCACTGATAGGGATTCGA

TGCAGCACATGAGGGGTTTGTTTTCAACGTTTGGGCAAATGCATACTCCCACTCATGCTC

AGCCTGTACGTCCCATGCTCCCATCTCCAACTCCTCAACCACAGATCCATCTTCACCATC

AACACCAACAACAGCATTTTCAGAGCCAGCAACCAAATCCAGCATCTGTTACTGCCATGC

CTCAACAACCGCCTGGCATCCGCCCTCGAGTTCGAGCAAGAAGAGGCCAAGCTACGGACC

CCCACAGCATTGCTGAGCGGCTGCGTCGTGAAAGAATCGCGGAAAGAATGAAGGCGTTAC

AGGAATTAGTCCCCAGCATCAACAAGACGGACAAAGCAGCTATGCTTGATGAAATCGTTG

ATTATGTGAAGTTCCTTAGGCTCCAAGTTAAGGTGTTGAGCATGAGTAGACTCGGCGGAG

CTGGTGCAGTTGCTCAACTTGTAGCTGATGTTCCTGTTTCTGCAGTTGAGGGAGAGGACA

TAGAAGGCGGAACCAACCAGCAAGCCTGGGCGAAGTGGTCGAATGATGGAACAGAACAAC

AAGTTGCTAAACTCATGGAAGAAGATGTTGGTGCAGCCATGCAACTTCTTCAATCAAAAG

CACTTTGTATCATGCCAATATCTCTAGCCTCAGCAATTTTTCGTATGCCTCAATCAGACT

CGTCATCAATCATCAAGCCTGAATCAAACAACCACACATAGACCATTAGCCTTAGCTAAC

AGAAAAATCTCACCACAATCTTGACATAAATTTCTTCCTCCATTTTCCTAAGGTTAATCA

TTCAACTTTTAATTTTATTTCTTCAAGTTTTGTCAAACTATAGAAGTAATTGTCATTGCC

AATAAAATGTAAAATTGGAGGAGCCTAACATGTCATGTCATTATCATGGTGAAAAATCAA

ATATTTTCAAAGACTATTGATATTTTTTCTATGGTGGGGTTGGTGAAGTATAATCAAAAT

ATTATATATTTGTAGTGGAGGCTTCTTTTTCTTAGTAATTTTTATTTTTTTGAGTAAGTG

GAAAGTGGGACCATTTAAAAGTAAGGGATATATAGGTTTGTAATGTATCATGTATTTGGA

GAAATTTTAAT

>LsbHLHR32

ATCGAAAAAGTAAAAAAAATAGAGGAAAGGAAAGGAAAAGGGTTTATTGCCAAATGAAGA

AGGCTCACGAGATACGCATTAGTTCAACCACATGCTAACTTACCTCTTCTCTCCGGTGGC

GGTCACTAATGTCCTTTGCCGGAGGCACTCACTCCCATTTCACCGCCGGCGTAGATCACC

CTCTAAGATTCAGAATTGTTGAGTGATACCCTTTTCCGTACAACAATGAAAGCAGGAAAA

GTGAACCACGAAGAAGACGAGTACGATGAGGAAGACTTCAATTCTTCAAAAAAACAAGGC

ACTTCCTCTGCCCCTAACACCAACAAAGATGGAAAAGCTACTGATAAGGCTAGTGTAATA

AGATCGAAACATTCTGTAACCGAGCAGCGAAGAAGAAGCAAGATAAATGAGAGATTCCAA

ATATTGAGGGATCTCATACCTCATTGTAATCAAAAGAGGGACACAGCGTCGTTCTTATTA

GAGGTGATTGAGTATGTTCAGTACTTACAGGAGAAGGTTCAAAAATATGAAGGCTCATAT

CAAGGTTGGAGTCAAGAGCCATCGAAGTTGATGCCATGGAGAAATAGCCATTGGCGTGTC

CAAAACTTTGTTGGTCAACCTCCCGTTGTAAAGAACGGTTCAGGTCCGGCATTACCATTT

CCCGGAAAGTTTGACGAAAGCAACATTAGTATCTCTCCAACTATGCTTAGTGGTACTCAG

AATATGATGGATCATGATCTGAGTCGGGATATTGTCAGAAAAACAATGGAGAGGCAACCC

GATTTAGCAAGCAAGGGAATTCCTCTGCCCGTACTACCTATGCATGCAAACATGTCTGTT

CCTGTCAGAAGTGATGGTGTATTTTCCCATCCTCTTCAGGGAACTGTTTCTGATACACAA

TCATCTGAGTGTCCCACAACTAGTGAACAACTGAACCAGCAGGATGAACTTACAATTGAA

GGAGGGACAGTCAGCATCTCGAGTGTATACTCCCAAGAGTTGCTGAACAATCTAACTCAA

GCGCTACAGAGTGCTGGGTTAGATCTCTCAAAGGCTAACATTTCAGTGCAAATTGATCTT

GGGAAGCGTGCAAACAAAGAGCCAAGCGGCACAACCTCTTCTCCGAAGGATCATGATAAC

TCTCTTTCTGGCAATCAAAACATTGCACATTTTAGAGATGTTGACATTAGAGAAGACTCA

AGTCAAGCTCAGAAAAGAATGAAAGCATACAAGTGATGATGATTCATATGGCTTTTTTTA

TGCATCCAGATTTCTTACTTTGTTTCTATCCCTTACTTCCTTGTGGTTATTGTACATCCT

CCCCCATTTGTTTGAGTTTAATTTCTCTCATGAACGTTACTTTAAACATACATAAACCTT

AATTACATAATTATTTGTGGAGATGATTAGGTT

>LsbHLHR33

ACACTCAACTTGTGAATCTCTGAATGCAGCAGTTATTTGTGTAAAAAGAGTGAGGCAATA

AAGTAATTTCACCTCGTATCCTTCCCAATTTATCAAAACCAAACCTCTTTACTTCTAAAT

ATAGCTTTAACCTCTGTTCTTTCTTTCTCTCTTCACAACCTTTTCCTCTGCCACGTTACA

CTCACACAAGGGCATTCTAGTAAAAACATCCTCTGAAAACATGTAACAAATCTGAAAGCT

GGAAATTATCACTCTGTTCACTCCTCATTGGCTGCACCTCCACAGCAACAAAATCCAATG

CTATTTTATACTATTATCTTCTCTCTTGAACCTTCCTATTCAGGTTTCTTCATTTTTTCA

TCACCACTCTCACCACTACACGCAACATAGAAATTTTTTCAGTTCGGTGCTCAGAGACAA

ACAGGAATTTTTTATAGAAGAAGCAAAAAATGGAGTCAGATCTTCAGCAGCATCCAACTA

TGTTTCTAGATCATCAAAATCATCATCACCAGCCACAAATGAATTCTGGGTTAACTCGAT

TTAAATCTGCACCAAGTTCATATTTTTCGAATATTATTGATAAAGAGTTCTATGAGCATC

TTTTTAACCGACCTTCAAGTCCTGAAACAGAACGAGTTTTTGCTCGGTTTATGAATAGTC

TTAGTGGTAGTGGTAGTGGCGGTGGTGATGCTGAAAGTGCTTCTGTTGCTGTTGCTGTAG

CTGCTGGAGATGATGATTCACTGACTCAGAATCTACTAACTGTTCAACAACAGCTTCCTA

TAGTTAAGGAAGAGATTGAGCAACAATCTCAAACTTTGATGAATTCAATGAATAATGAAA

CTGTGGATGTTCAACAGCTTCAACGACAACAAAGTAATATGAATAATTATGGTTCCTCTG

GTCCTCAGAAATTTTACCAAAGTTCTGGAAGACCACCTTTGCCAAATCAAATGAAGACTG

GTAGAGGAAGCTGTTCTAATCTTATTAGACATGGTAGTTCACCTGCTGGATTGTTTTCAA

ACATTAACATTGAAACTGGTTTTGCTGTTATGAGAGGCATAGGAACGATTGGAGCTGCTA

ACAGCACTAGCAAAGAAGGAAATTTCTCTTCTTCTGCGGTGCTGTTAAAGAATGTGAGGG

CTCCGAACTACTCGTCGGTGTTAGGAGGTGAAATCGGGAATAGTAGCAATCCACAAAATA

ATCTAGAATCCGAGGGTTTTGCTGAAACTCGGGGTAATGATTTTATCCCGGGTTTCCCTT

TAGGTTCTACATGGGAGGATACTGCAATGATATCCGACAACATTACCGGTCTAAAAAGAT

ACAGAGATGATGACGATGTAAAACCATTTTCTTCCGGTTTAAATGCGGCTGATACTAAGA

ATGAAACAGGAGGCCAGACTCCTGCCACTCCTTTGGCTCATCAGACGAGTATGCCTAATA

CCACGGCAGAATTGGCAGCCATTGAGAAGTTTTTACAGTTGTCAGATTCTGTTCCATGCA

AAATCCGCGCCAAGCGCGGCTGCGCCACTCACCCGAGAAGCATTGCAGAAAGGGTCAGGA

GAACTAAAATTAGCGAGCGAATGAGGAAACTACAAGATCTGGTGCCGAACATGGATAAGC

AAACAAACACATCAGATATGTTGGACTTAGCTGTTGAGTACATTAAAGACCTACAAAAAC

AAGTTGAGACTCTTTCACAAAATAGAGCAAAGTGCACGTGTTCACATCACCAATAAGGAA

ATGAAGGATGATGATGATGATGATAAAGGAGTTTGAGTTTTTCTTTTGTACAGATTTTGT

TGTAATAATTTGGTCTAGAAAAAAAGTTGCTTCATTTTCTGATATATTAATAATTTTTGA

TTTTGTTATATTAGTATTACTATGTTGTTGTTACTTTCTCTTTGGTTCTTGTGGAATGGT

GGGGGAACGAGATCATAAAAGTTGACTTTTATTATAAGATCATAGAATTTGATGTATGTA

TGTGATGTCATCACATATACAAAGCATTGTGTACGAAATTTGGTGAAGCAAGTTTATTAT

GTTGGTTTTAAT

>LsbHLHR34

TGTATAAATTGGTGAAGTTAATTTAGCAAGAAAGGGAAGCAAGCACATGCAGATTGCAGA

CACACTTGCTTGGATAGATAAACCTCACTCACTCAACCAGCCATTCCATTCCCTCTTTTC

TTTTCTTTTCCACACACAAGATAAACACAAACACAAGAACGTTCTCAACTCCCTCATCAT

TCTTCACACAATTCATCAAACAAAACAATAACCTCTTCACCTTTTTTGTCTCTCATGGAA

CGTTACACGCTACCCTTACCACAAGGACTTCACAACTCTCTTACTATACCTTGGACTCAA

CCACAACAACAACACCAAGAACAACAATCACCTTCATCATGGTCCACACCTAACTCCGAA

CCTAAACTCAACACCAACGACCAAGACATTGCCATTGCTATTGCCATGGCTGCTTCCACT

ACCTCTCTCCCTTTCTTCAAACCAGAACCTGATAACTTCTATAACCTCAACTCCACTACT

ACAACAACCACCAACAACAACAACGTTGTTCCGTTTGTTTCAAATCATACTTCAACTAAT

GATAATTTTCTCATGCACCAAAACACAAACACCATGGATTCAATTTCAAATCCACACCCT

TTCTTCCACAACAACAACAACAACTACTTCTTCAACAATACCAACAACAATAATAACCCT

TTTGAGATGGGATTCGAAAATGGATTCTTTATGGGAAACAATAACACCAACGCTTCCAAT

TCGCCTGTTTTTATGGGTGGCTCCCTTGACCTGAGTTCCGCTTCCGAGTTTCCACCGAGT

CTTGAACTCGATGCTTCTGTAGCACCTTTTAGTGCTTCTTTTTCTATGCCTCTTGAGCTG

TCTCAACCGCAACCTCAACAGCAGCAGCAGCAACAGCCCACGACACTGTTTCAGAAGCGG

CGAGGGGCGTTGGAGATTCCGAGGTTGGAGACGGTGGGGAACAAGAAGAAGAGAAAAGTG

GAGAAGAAGTGGGAAGAAGATGGAAATGGTGGTGGTGAGGATGATGTTGAGGATTTTTCT

GAGTTGAATTATGATTCTGATGAGAATGGAAATGATTTGAACAATTCCAATGGGACTGTT

GTTACTGGTGGAGATCAAAAGGGGAAGAAGAAGAAAGGGCTTCCTGCAAAAAATCTCATG

GCGGAGAGGCGTCGGAGGAAGAAACTCAATGATAGGTTGTATATGCTAAGGTCTGTTGTT

CCAAAGATTAGCAAGATGGACAGGGCTTCGATACTTGGTGATGCAGTTGACTACTTGAAG

GAGCTACTGCAGCGGATTAACAATCTTCATAACGAACTGGAATCAACTCCTCCTGGCTCA

TTGCTGCAACCTTCTGCAAGTGCAAGCTTTCACCCGTTGACACCCACTCCGCCGACCCTT

CCTTGCCGAGTCAAAGAAGATCTATATCCTAATGACTTGCTAAGCCCTAAAAACCAATCT

CCTAAGGTGGAAGTTAGGGTAAGGGAAGGGAGAGCCGTCAACATTCATATGTTTTGTACC

CGAAGACCGGGACTTTTGCTTTCTACCATGAGGGCTTTGGATAACCTTGGATTGGATGTT

CAACAAGCTGTTATTAGTTGTTTCAATGGCTTTGCTTTGGATGTTTTCAGAGCCGAGCAA

TGCAGAGAAGGTCAGGATATTCCCCCCGAGCAAATTAAAGCAGTACTTTTGGATTCGGCA

GGATATCATGGTTTGAATTGATTGAAGTTTCTGATAAAATGCTACCCTTATGGAGGTACT

AACTGTAGCTGCTGTAGCTCGCTGTTTGTAGACGCATTGCTTCTTGTTCTTCGCTTTAGT

AAAAGCCCGTGTCTTCAGGCCAAGATCAAATAGATTAGCTTAATCTTTAGGTGGAGAAGA

TAGCTGTTAACATTTTCTCAATTGCGCTTATGCCAAAAGCGACCGCATGTTATTCAATTT

ATGATTAAGTGGTGTAATGAATTGTTAATTTTTTTTTCATGTCCATTTCTATATTAAGTT

TTTTGCCTATTGAATTTTGAATGTGATGCAACTTGGCAGAA

>LsbHLHR35

AAAAGCATGCATATTTAGCATAACCAAACTTACATATAAATAGAAGTTTTGAACCAAACA

TAATAGATTATCATCTAAAATTAATTAGATTAGAGGATTTAAATATTCCAGTTATTATTA

TATAGCATCGTGAGCAATCAATCTTTTCATAATTACCACAGAATGACTGCTCATGATGTT

ATATTATATATCATCCACAAATTTTCTTCATCATGCATGTTAATTAAAATCCACTTCCAA

ATGCTGGTGCCCAATAATCAGCTCCATTTTCACTTCCAACATGTTGTGTGCATGAAATAG

GAACCAAACACAACCCTCTACTCCTCAAGTCCTTTATCTTATTACCTTCTACATTCTGGT

TTGGAATTGGAGCACCCTTTCTTTTTAGGCCAGTGTCATCCAACAGCTGACCAGGGTCCT

CAGGAAACACAGAGTTTCTTTCAACATGCACAGAATGTTGATTCCTCATCATGTTTTTTG

AAGCTGCAGTGTCCAAGTAAGGAGAGCTTAGTGCCTCAATTTGACTCTGCAGAAATCTGA

TGTATCCAATGGCTTCTAATAATACAGAAGCTGTGTCAGTCTTTCCAAATGGAGAAACTA

GTTGGTGAAGTGCTGTTATTCTATCACCTAGCTTCTCCTTCCTCACCTTTAGAGGTGCTT

GGCTTGAAGATGGCTGAACCCTAGCCTTCTTATTAACTCCAACAGTTGAACTATTGCACT

CAGATATTTGATCTGGTAGTTGGTTTTTGCTATGATCTAGCTTGTTATAGGTGAAATCCA

ATATATTATCACTACTTAAGCTAGTAGTGACATGAGAGCTTGGAGAAGAAACTGGCACCA

TGTGTGACCAAGATGAACCAAGTCCAGAAGTATGAAACTCTTCATGATGATGATGCCCTT

GGTTATAGAAATTCTCACTCTGAGAAACTTCGTGCTTTATTACATCCATAATAGGAACTC

TTGAAGATGGATTCAGAATTTGAACATCCCAGTTTTCTGTGTTTTTCGGTTGAAAATGAT

CGAAACCAAGCCTCTCTTCTTCTCCAGGTAGTCCAGTAAAAAGTAGTTGACTCCATGATT

GAGGAGGAACTTGAGCATTTTCTGACAAGGAATTAAAAGGGATTGAAGAAGATCCAAGCA

TATATTGAGGGATATTCAAAGATTGTGGATGCATGTTCCACCAGTTAGGGTTTCCAGCCA

TCATTTGCTACACTTGAAAAACACTCTCATATTCATGATATATGTGTATGAAAAAACTAG

TTTCATGTTTTCATCTTCAAGAAAAGAAAAATAATTAACCTCTCTTAAGAAAAATGAAAA

ATTTTAAGCTACTTTTTTCTTCTACTTTTCTACTTTCACCTTTGTGATGATAAATTTCCT

TGTATGTACAACCAAACAAGACTTTGGATAAAGTTTTCAAGAGTTGTGTTTTGCTTTCTT

ACTAGCTATGTGTGTTTGAAGAAACGTAAAGTCTTGGGGTCTCAAAGTATTTTATAGAGA

TGAGGTTAACAAACTTTGCATACTT

>LsbHLHR36

CTCATAGCAAATATTGTCCCATGCCCTAATTTAAAAACACTTTCTTTAAGTGAAGTAACT

CTCAAAAGGAGGTGAGAAAAGCTGCCACCTCCACTACCATATTTCAAGCAAATATAAGAG

TAAATTAAACCAACATCAAATACTTTATAACTTGCACATTTTTTTTCTGATCAAAATAAT

AATAAAATAAAAGTGGGTGCATGCATCCCCTTAGAAAGATATACATATATTCAAATATAT

CTACTAACAAGAACAAAAGGTGTTTTAGGTAGCTAGCACCATCTGCTAAATTCAGCCATA

AAACAAATTACAATTAAAACTATAATCTAATGTCAAATGCTGCTGAATTTAACACTTGTT

CCTGCCCCTCATCAACCTAATTTGTAGCTATTAAAATCTTAATTAAACAACCTATCTTTA

ATCTCTATTTTTTTATTTATTTTCATTCCCTTCTTTTTTCTTCTTTTTGCTTACCAATTA

ATAATCATGTCTTCTTCCTCTTCTTTTAAAATCATTTCTCTATCTGCCAAAGAGTGTTCC

TCCAAATGTAGGTGTCCAAAAATCAATACTAGTCTCATTAGTCATTGGAAATGTGCTTGA

AATTGGCACCAAACAAAGCCCTTGACTCCTTAAATCTTGTTTTTTTCTTTCTAACTCCTT

CACATTATCACAACCCTGTTGGTGTTGAATAGGAGATCCATTTTTCATGTATGGAGTGCT

CAAAACATTGACTTGATCATGAAGAAACTTGATGTAATCAATGGCTTCATGAAGAACAGA

AGCTGTATCCGTCTTTCCGAAAGGTGAAACCAACTGCTGAAGAGCTGTGATACGGTCCCC

TAGCTTCTCTTTCCTAACCTGCATGTCCACCATACATATACATACATATATAGTTACATA

CACATCAACTTTATCTAATCTATAGATTAAAGAGTAAT

>LsbHLHR37

CGGCAATAATTTACATGTTAAATGTGTATAAATTAGCTTGCTACTGAGATCTGCTAAAAT

GTTAGCTCTAGCACATTACACCACCATGACAGCTGCTATGATATCTAGATGCCGGATAAT

CTATCCTGCCTGATGCTTTTCTGAATTACCCTGGCCCCCACTGTTACCTGGCCTGTTGAC

ATTTTGTTGGTTCATTCCCTGTATTAATTGCTGCAACTGGTGGGGACCGAGGAATTGCTG

ATAAGGATCAGCGAAGTTTGTGTTTGGGATGCGTGATTGATCTGGAAGCAGAGTACCGAG

GGAGTTAAGGGGATTATCTGATGATTCAGGTACTGTAGGAACATGAGGCTGAGGCATATG

AAAAGGAGGCACAGAGAATCTTGATCCATATGGAGTAGCTACTGTTGTTGTTGTTGGTGG

CAAAGGTGAACTTGGTAAGATATTAGGAAAGGACATCACTGGTCTGTTCATTCCCATTTC

CATCCCTAAACTCATTCCCATCCCCATGCCCATTCTCATCCCCATCGTTGGCATATACTG

CTGCATTCCTGGAAACATCATCGGTACCATGCCGCATCCCATGGACATCATCTGCACTTG

TAACTGCAATGACTTCAAGTATTCGATTGCTTCATCCAACATTGAAGCTTTGTCGGACTT

GTTGCACCGGGGTATAAGTTCTTGCAGAGCTTTCATTTTTTCGTTAATTCTATCTCGACG

CTTCCTCTCTGAGAGATTATGAACCTCTGCAGCACGGGATCTCTTTGTAGATGTAGATCC

ACGAACTTGTTTTTTTCCTTCCTGCCTCTGTAATTCGGATTTGTCTGATACACGACCTTT

CCTCTTTCGTTCTAACTCCAGTTTTCTCTGAATCGGATCAACACTTCCACTTGAACAATC

ATGCGATGACGTCATTGTAATATCTAACTTTGAAGTCTCTTTCACTCCTTTAGTCGTTGC

AGTTGCAGCTTTTCCAACCTGGCTTATAGTACCACATCCAGTATATGCCGCCGCTTTTCC

GGCAGTGCTAACACTCACGCATTCTGTATACGCGGTGCTCTTGATTGTTTCAGAAAGCTT

TGAAGCTGTGTTAGTCATCTCCGTAATAATCGGTGTATCGCATGAATCCACCATCGTGGT

TTCTTGTCTAGCAGGAATCATTGAGCTTGAAGAGATACTTGGTTCTATACTAGCATTGCT

ATGGTTAGCAAAATATGTGAAATTATTATGCTTTCTATTCAGCACTTGATCCTCCTTCCT

CGCGGGATGAATCGGAGGCCGAGAAGCCGATGGTATCGGTTGCGGAAGTAACTGCTCAGA

CTGAAGTAGAGAAGAACCTGGTATAACACCGGTGGATGGATGAAAAAGTGTTTCAGTGTT

GATGATAGGAGGATCTTCATTGATTGAATCGAAAAGCCACGATGCCATTTCATCTTCTTG

CATGAACAAGTGTTGATTAATATTGAAGTTTTCTGCATCGGAGGATCGAATCTCTCTATG

ATCAGGAATCACTTGATTGCCGGTGTTAGTTACCGGCGATGGCGGTTTTTTAGATTGGCG

GTGGCTTTGGTTATGCATCACAACTGAACCGTTTTGCCATAACAGTTCCATGATCTCATT

GTTTCGCGCAGCGGAAGGTTTCTTTGAGAGAAGCACAGGGTTTTCTTCTTCGTCGTCGAT

TTGAATATCGAAATCTGGAACACAGTGATTCATTTTGCTTTTGCTTTGAACTGATCGAAT

CTATTGAAGCTAGTTAACTGATCCAGAACATAGTAAGCAAAAAAAGTTAATTTGTGTGGA

GCTTAAATTTTTAAGTTATATGAATAGAGATTAATAGGAAACTATGGTTGATTTGACTTG

TGTGGTTGGTTGTGTTGAATATGCGTTTTGTGTGAATAATTTAGGGGTATGAATATGATG

AATTGAAATGAAAAGTTAAAGAATAGAGCATAGAGAAAGAGAGAGATAAGATGTGTCTGT

GTCACCGAATAATGGATGAATGGGAAGTTAGTGCTAAGGACGAAAGGGATTTTTGATGTG

TCTGTGTCACCGAATAAT

>LsbHLHR38

TTGTCTCTCCTTCAAAAACAAATGCACTACACTCATTAGTCATCAGCCTTGTCTACTCTC

TCTCACTACCCTCTCACTCTCTCTCTAGAGAACAAAAATTTCTATTTTCAACAACAAATT

CTCCCATTATCAAAATCAGCAACAACTCTCACTCCCTCCTCCGCTCCGCCGCAAAACACT

TCACTCTCTCAACAAACACAGCTGACCATTGCATGGAATGAGAGATTACCGCTCACTTCC

GACAATGAATAGTATCTGGACAGACGAGAACTCCTCCGTCATGGAAGCTTTCATGAGCTC

CTCCGACCTTTCTTCTCTTTGGCTTCCAACACCAAATTCGGCCGCATCAACCACCACACC

AGGACCCGACACAACCAAACTTCCATCACAACAACAGCCTCTCTTCAACCAAGAAACTCT

CCAACACCGTCTTCAAGCTTTAATAGAAGACGCAAAAGAGAGCTGGACTTACGCCATCTT

CTGGCAAACTTCTTACGACTACTCTACCAGCAGACAGCTCCTCGGTTGGGGAGACGGTTA

TTACAAAGGCGAAGATGACAAAGAAAAAGCCAAAAAGGTTATCTTGCCTGAACAACAAGC

TCATCGCAATAAAGTCCTCCGAGAACTTAACTCCTTAATTTCTGGCTCGTCTGGCTCAGA

TGATGTTGTTGACGAAGATGTCACCGATACTGAGTGGTTCTTTCTCACTTCGATGACACA

CTCTTTCGTGAACGGCAGTGGTTTACTGAGCCAGGCTTACTTTAATTCTTCCCCGGTATG

GATCAACGATAGGCTTTCCATGTCCACATGTGAACGAACCCGCGCAGCACATGTTCACGG

GCTTCAGACTTTGGTATATATACCAGCACCGTCTTCAAACGGTGTCGTTGAGCTCGCATC

TACTGAGATAATTCCACATAGCGCTGGTATAATGGAGAAGGTTCGTTTTTTGTTTGATTT

CAATAATCCAGAAGCACGATCTTGGCCGTTGAATTCCGGCGACAACGATCCTTCTTCCAT

GTGGTTGGATATCCCCGGTTCCGGTGGGATTGAAATTAGAGACTCCGTCAATACTGTTAG

CGCCGTTAGTGTAACCACTTCAGCAAATACAACAATCACTAAAAAGTTGCCGTTTGAAAT

TCACGGTGCTTCTTCTAGTGTACCGGAAACATCCACCGCCGTTAACATTTCAACTGGTCA

CCGAGAAAACCAGAACCAAAATCAGAACCAGACCTTCTTTCCAAAGGAATTTAATTTTTC

AGGTTCTTTCAAACCGGAATCCGGCGAGATTCTGAACTTCGGCGAGAGTAAAAAGAGTTC

TTACAGCTCCGCCAATGGTAATTTCTTTCCCGGTCCGTCACCGTTTGCTGCCAATGAGGA

AAACAGAAAAAGGAGGTCTCCGGTGTCTAGAAGTAGTATCGACGATGGAATTCTTTCATT

CACTTCTGGAAAACTGTTACAGGCCTCGGGTATAAAATCTGGCGGCGGAGATTCCGATCA

TTCAGATGTGGAAGTTTCCGTGGTGAAGGAAGCTGTGAGCTGCAGAATTATGGAACCGGA

GAAGCGGCCTCGGAAGCGAGGTAGAAAACCAGCCAATGGAAGAGAAGAACCCTTGAACCA

CGTGGAAGCAGAGAGACAGCGACGAGAGAAACTGAATCAAAGATTCTACGCTTTACGGGC

TGTGGTTCCGAATGTTTCTAAAATGGACAAAGCTTCGCTTCTCGGAGATGCGATTTCTTA

CATAAATGAATTGAAATCCAAGCTACAAGGGCTTGAATCATCTAAAGGTGAATTGGAGAA

AGAACTAGATACAACCAGGAAGGAACTAGAAATTGCAACTAAAAAACCAGTTCTGTTAAA

TGAAGAAGAAAAAGAGAAAACAAACAACAACTCTAAGTTGATTGATTTAGACATAGATGT

GAAAATCATGGGATGGGATGCAATGATTAGGATTCAATGTAGCAAGAAGAATCACCCTGC

AGCGAAATTAATGGCAGCGTTAAAAGAGTTGGATCTTGATGTGAATCATGCAAGTGTTTC

TGTTGTTAATGATTTGATGATTCAACAGGCTTCTATTAACATGGGAAGTAGATTTTACAC

ACAAGAACAGCTTTTATCGGTTCTTTCTTCCAAAATTGGTGATACACAATGAGATTGAAT

GCTGCAAATTTTCTCTGCTTTCTTGTAAAACAATTGTGTTCCTTTTTATGAATTTTAGCC

AGTACTAGTACTTTGATTAGCTTGTTTTATATTTTTGAAGTAATTTTATCAGTAGTAGTA

GAATTAGGAGACATCTCTCTATGTAGGTAAAGTAGACTATTTCTTTTTTTTGTTCATAAT

AGTCTTGGGGCGGGGCGGCTTTCGCTCGTCTATCTACTTCCGCTTCTAATCGGAAACGGG

TTTTTCAATTATCATACATATGAACTCAGCTTGATCACGTTTATGTGTATTAATAATATT

GTTAACCATTTTATAAATTCTTCATAGGTGATTTCTATGTGTTTTTGGTTAGTTGTAATG

ATTATTATTATTAG

>LsbHLHR39

TCCCAACTAACAACAACTTGCTTCTTTATTTAATCATTCCATCTTCTTTTTTCTTAGTCT

TCTCTTCTTTTTTCAGCACACAGATTCATCATGTATCGTCCTCCTTCTTCTTCTGCTTCT

ACTTCTTCTTCCTCTTCTCAGCAACAACAGAGTTCCATTAGCCAAACTGGCCTCACAAGA

TACGGTTCTGCACCCGGCTCTCTCCTCACCAGCACCGTCGATGCTGTCTTAGGAGGATCG

CGCCTTCTCCCGGGAACCGGACATTATTTCTCCGGTGACTCCAGCCACCTGCAACCGCAA

CAACAGCAACAACACCAACAGCAGAGATCTTCTTACGAAGGCTTTGATGGATCATCTTTA

GTCCGTCAGAAAAGCTCTCCCGCCGGTTTCCTCAACCACCTCGCCACTCTCAATCATAAT

AACAGCGCCGGGTTCACAATCACGCGAGGCGGAAACGGAGGATCAAGATTGAAGTCTGAA

TTGAGCTTCACCGGAGGAGGACAAGGACAAGAATGTCTTTCTAGAATATCCGAAAATGGC

GTTGATTATGCAGCAGTAGCAGCAGGAAATGGATCATTGCATAACAGTAACTGGGGTGGA

GGACCTGATAGTAATAATAATAATAACAATAACAATTCGAATTCTATAGTGTTTTCTTCT

TCTGTTTCTCAAACTCAAACTAATAACAAGCGTTCGAGTAGAAACGACGACGATCCCGAC

CTTCTTCTTCATTGCCTTAACGCCCTCGAATCTCAGTATAGTCTCCCACAAACTTCTATG

GAAATGGACAAGCTAATGCATATTCCTCAAGACTCCGTCCCTTGTAAAATCCGCGCCAAG

CGTGGCTGTGCCACTCATCCCCGAAGCATCGCTGAACGGGAGAGAAGAACTAGAATAAGT

GGAAAGTTGAAGAAATTGCAGGATCTCGTGCCTAATATGGACAAGCAAACAAGTTATTCA

GACATGCTAGATTTGGCGGTTCAACATATCAAAGGTCTTCAAACTCAAGTCCAGAAGCTT

CATGAAGATCTTGAGAATTGCACTTGCGGATGTAAACAAAATACATAAACTTACTATGTT

TTATGAGAATCTATGGCACTGCATACAAGTCAAGGTTTTGATGGAAACTGATTTTTGTAC

CTTCCAACTTTAAAAACGAATAAGGAAGGAAATTGTACATACAGTTTATGTTATAATCTA

CCTGTGGTAGACATAGTCTAGAAATATCTATTCCTTTTCCTAATTTGATAGTTGTATTTG

AAAGTAAAAGTTGATTTTCTTTTGAGTTGGCTTTATTGTTTGAGTAAGTGGAATGAATAA

CTTATTCTTATAGCATCAGAAGGATAGAAGTGAGGATGAGACTAGTTAGAAAGATACAAT

AAAACAAGTATTCACGTAGTTACCATTTTATTT

>LsbHLHR41

TACCTCCATTGCCCAACTTGAACCGTGTTGATAACCAGAGGATCCATGAATGTCTTTTTC

CATGTGATCATGCAACTTTGTCTTTGTTTCAGAGAATTTATGTAGCTTATCAGCATGCTT

GGTTACGCTTTGGAGAAAGAGCATATGCTTTATTGTGCACTCCAAAAGTGAATCAATACT

GCACTTTGCTCCATTTGGCACCAGCTCTCGTAACTCCTTAATACGGTCTTGAATCAGTTG

TCTGTCCCTTGGTCTAGGCCTACAACTCTCCCCGGGCCTAGCCCTCTTTTTGCTGATTTT

AGATGGTTCAGAAGACCTCTCAAACTGGTTACTACACGAACTAGGACATGCTGAAGAAAA

GCTTTTGGGGGATATCACACCGCATATTCCTGATGAACTCAAACTATGATTATGCTTGTT

CTCTTTAACATGAGATGGTTGATCAATTGAATAGCCTTCTGAATTAATAGTGCTGACATT

ATGAATAGGAACTTCGGGATTTCTTCCAGAGACCATTGCATCGTGAACTGATGTAGAAAA

TGATAACTCGTTATCAACATTATTACTATGGCAAACTTTTGTCACCACTGCTTCCAAAAG

ATGCTCCGGACGAGATTCAGAAGTCAACTGGCTGCAGCTAAATTCATCCGACATCTCAGC

ATCTTTACTGTCTTGATTTCCCTGTACCGCCCAGTCAAAATATTTACTCTCTTTAAGAAA

ACCTGGTCCAAGTGCTTCATGTAGCTCATAGCCAGCAGGAAATTTCGACGCATACGAGAT

ATCCTCGTAATAAGACTCAGTCTGAAATGTTGACATCTCAGTATCAGCTCTAAGGTATGG

ACTTACTAAAACATTGGCACTAATACTATTATCATCCGTAACTGAATTGTGTAAAACCGG

TGAGACGTTTTTGTCTGATTTCAAACTAGTATCTTTGAAGCTATTATTGTTCCCTTCGCA

CTTCCTTTCGTTTGAAGGTTTCACCCCTTCTAGTTTCGGATTCTCTGCAGTCATCATATT

TGACATTGACTGAAGCAAAACGGAACTTTGATCACTGTAAATTTCAAGCCCTCCATGCCT

GGCATCAACAGTCGTTTTCTGATAAACAGTAGGAGGAGGTGAGTAGTTCTTCCCAGGACA

TTGGAAGGGCATTAACCTATCCGATGTTTCACCCATCATGGTTCTCTGTGTGTCATGTTT

CACAGTTTGGATTCGACTAGGAAGGTAACCTAATGTTTGATCATGAGTAGACAAAAAGAG

ACTTCTAATACGTGTTACAAACCCCGTGTCTTCACTAATTTTATTTAAAGAGCCGAGTGT

AACAACTCCAAGTGGGACTACCGCTACAACAACAATTGTCCTAATTCCAGCAGAACTGAG

TTTGCCACAAATCATC

>LsbHLHR42

TCCCTCTTATTATTGGTGTTGTTATTCTGCGTAGCCAAAATTGAAAGTTGTTATTCCCAG

CTAGAGCGGTTTTTGTTACAGCGAGAAATTCAACCGCAAATGGATTCTTTAGAGTTAGGA

AACACCGATTCTTGGGATTTTCTCGATTACTCATTCATCGACCCTCCTCCCACCGATTTC

CTTTGGTCCAATCCAAGTGATTTTGCGAGTGTGAACACGGAAATTGACATTCGAAGCAGT

AATTTTGCGAGTGTGAGTGCAGACATTGGCATTCCGAGTGGTGTTGTTGCGTGCCAAGAA

GAGAATAATACCAGGAAGAGGGGACGCGCTGAATCATGCCACAAGGCAGGAACAAAAGCT

TGCCGAGAGAAATTGCGGAGGGAGAAACTCAATGAAAGGTTTTGCGATTTGAGCGCTGTT

TTGGATCCCGGGAGACCTGTGAGAACAGATAAGCCTGCTATACTTGACGATGCTATTAGA

GTCTTGAACCAACTTAAAACTGAAGCTGAGGAACTCAAAGAAACAAATGGGAAATTGTTA

GAGGAAATAAAATGTTTAAAGGCAGAGAAAAATGAACTCCGCGAAGAAAAACTTGTTCTG

AAAGCTGATAAAGAAAAGATCGAGAAACAGCTGAAAACTTTGCCTATTTCACCAGCAGGA

TTTATGCCTCCTCCTCCCATGGCTGCTTATCAAACAAGGGTGAACAAGATGGCTGTTTAT

CCAAACTATGGATATATCCCAATGTGGCAATATCTTCCTCAATCAGCTCGCGATACATCC

CAAGATCACGAGCTCAGGCCTCCTGCTGCATAGTTGCTTTCTTTCTGTTACTTTCCTCTG

GTTAATTTTTCATTCACCATTTTGATAACACTGTCTTAGTATTTTGACTTGTAAATTACC

ATTGCTTCAAATTTTCAGGTCTTAATAATGATAAATTAGGCTTACGATTACCATTCTATG

TTAGTCCAAGTTTCAAATAGATCTCTCCTTTAACAGTGTCAGTTTTTTTTTAATAAACCT

AAGGGCATATTATTTCTTCTATGAGAGAAAGATGAAAGCAGATACTTTCATTTGGAACAG

TTAAGAATAAAAGGAATAAAAAGAAAAGGTGGTTTCCCATCATGGGAAAA

>LsbHLHR43

GGATATTGGTTGGAGAGTGGCTCGTGGAAGACACAAGCTCAATACGCGTGCGTTTCCATC

TCCACCTTTCTCCCCACTCTACATATTTAATTCATACCACACTTCAATTCTCACCGGAAT

ACCAACAGCATGAATCAACGCCACCATCACCCGCCGCTTAACTCCTCCATAACTCCATCC

TCTACGGCGCCGCCAGATTCACCTCCGCAACACATCACCAATCGCAAACACACTGTCAGG

GAGAGGAGTGAGGTGGAATCTCATGATCCAACTGCCGCTAGAAAAGTTCAGAAGGCTGAT

CGTGAGAAAATCAGGAGGGATCGATTGAATGATCAGTTTCATGAATTGGGAAACGCGTTA

GATCCTGATAGACCGCGGAACGACAAAGCAACCATCATCTCAGAGACAATACAAGTGTTG

AAGGACATAACTGCTGAAGTTGATAAGCTGAAAACGGAACATAAAGCATTTTCTGAAGAG

TCGCGCGAACTAATCCAAGAAAAGAATGAACTCCGAGAAGAGAAAGCG

>LsbHLHR44

AAGAGAGATTATGCAACTCTCACTACTGCCTCCAACCCTTCCATTAATTTCTCTCTCTCT

CTTCAACATATTAAATAATCTTTCATTCATTTCAATTTTCAACCCTCCCACTTTCTCATT

TCTCTCTTCTCTAACTTCCGCACTTTCATTCCTTCATGCAACCTTGTAGCAGAGAAATGC

AATCCCTTAACTCTCTTTTCAACTCTTCTTCTCCTCCTCCTCAAATTCCCATTCCTCTCC

ACAACCACACACAGATCCAAATCAACAACGACGACACATTCCAACAACAAGACGATTTTC

TCAAACAAATGCTCTCCAATCTTCCTCCTTCCTCTCCCTGGAACAACCCTAAACCCTTAT

GGGACCCTAACTCCGACGATAATCTCACTTTCCCTTACGATGAACAAACCAACCTTTCCT

CCAAGTTTCGTAACCACCAGATCACTGACAAAACCGCCGCCGCTCTCATGCTTCTCATGC

CTTCTGCCGCCGATTCCGGACTCCTCCACATCCCCGCTGACTTTGACTCCTCTCAAAACG

ACGTCGTGAACGCTTCTTCCGCTGGTGACGGTTCCGTTCAAGCTCTCTATAACGGTTTCT

CCGGATCTCTCCACGGTGTCGCCAATCAAACTCACCATTTTCAACCTCCTCAGGTGCAAA

GTTTTGGAAGTGGTTCAGTGAGTGCAACGAACCAGGCTCCGGTGAGTGGTGCTCCGGCTC

AGCCTAGGCAGAAGGTTAGGGCTAGGAGAGGCCAGGCCACTGATCCGCACAGCATCGCCG

AAAGGTTAAGGAGGGAAAGAATTGCTGAAAGAATGAAAGCATTGCAGGAGCTTGTTCCGA

ATGCCAATAAGACAGATAAGGCCTCCATGCTGGATGAGATAATCGATTATGTCAAGTTCC

TACAAGTTCAAGTCAAGGTTTTGAGTATGAGTAGATTAGGCGGAGCAGCAGCCGTCGCTC

CCCTTGTTGCTGATATGTCCTCTGAGGGTGTTAGTGACTGCGTCCAAGCTAACGGAAACG

GTGGGGTCCACCCTAGAAACCCTAAAACGTCGTCGTCCAACGAGAGTCTCACCATGACGG

AGCATCAGGTGGCGAAGCTCATGGAGGAAGACATGGGATCCGCCATGCAATACTTACAAG

GAAAAGGTCTTTGCCTCATGCCGATTTCTCTCGCCACTGCAATCTCCACCGCCACGTGTC

ACAACAGGAACCCTTTGATTAACGCTCCCAATAATATCAACCCTATCACCGCATCCAACG

GCGATGGGCCATCCTCTCCCGGAATGTCCGTGAACAGCACCGTCAAAGATGCTATCTCCG

CTTCTAAGTCGTAGACGCCGTTTGGTTTGGTTGACCTTTGGTAACAAAAACAAAAGAGTA

TCTGGAGCAGAGCAGAAATTTCTCAAATCTAGGAAAATAATACGTCACGTTTTGGACCTT

TACGACGTCGTATTTACCTAGATTTAAGGAGAGGCTAACTTTTATATTCATTTCTCTGCA

TATTGATCACAACCTTATCAATTTTCTAGCTTTTTTTTGTTGGTGTAATTTTCCCTTTTT

TTCTCGCAATGGTTCAATCTTACGCGTTCATTTTTTTCTTTTGGAGAATGGTAACAGGTG

CACTAAAATGTATTTGTTGAGAAATAAATAAAAAAAGTTTGC

>LsbHLHR45

AAAGAAATTTTGAAGCATAAGCGTACTCAAGAACCTATTAAAATCAGTTTGATTCTACTT

AGGTTAGGCAACAACTTTGCATTATCAATTTGCAACTAAAAAGGTAGAATTTTTATCACA

AAGGGAAAGTGAAACTAGCTCTAAAGATACCCTAGTGTATGTTAAATACAGGGTATAACC

CTAAGAGTGATTGCATCAACAAAAGCCAATTTATTTGGAATATCATTACAACTTTTAACA

ACCTAATTACTTGAATTACATAAGAGGAAAATTTTGCTCATTTTCACCATCTGCAAATTG

TTATGTCAGCTTTCCATCCTTTTCCTAGAGGCACCTCCCACCATAACCTGCATTCCTGAA

CAGTGATTGCTTTATCTCTTCAGGGTTCATACAATTTCTCTGTCCAGCTACCTCTGAGCA

AGATGCCTGCAATGAAAAATCATTGAAACTGCTGATAACACATTGGTGAATCTCAAGGCC

TAATGCTTCTAATGTGTTCACTGTTGACAGTAGTAAACCTGGCTTTGTGGCACAGCAGAT

GCTAATCCTTGTGTCTTGATCTCTCCTCTCAACATTAAACTTAGGGGAATTTCTTACCAT

GACTTCATTTGGCTTTAACTCCTTAGAAATTCCCAAAACGTTTATCTGATCTGTTCCTTC

ATCCATTTCTTCTTGCAACTTCCCAATCCTTTCAAGAAGCTCTTTCATGTAATCAATTGT

ATCTCCGAGAATCGATGTCCTGTCCATCTTACTGATCTTAGGGACAATCGACCTAAGCAT

GGAAAGCCTATCATTCAACCTCTTTCTCCTCCTTCTTTCCGCCATGAGATTCTTCGAAGG

TTGCCCCTCAACCCTTTTGGATTTACCTTTCTTCTGTCCACCTCCACCACCACACAAACC

CATATCAAAAACAGGAATCTCACTCACATGCTCCTCAACTTTACAACTAATTTTACCTTC

TTCCAAACCCTTACTCTCACTTCCAACAAAACCAAACTCTTCAACACCCTCTTGCGGTAA

AAGCGGTGTTGAATCATCAAGCTCGGGAACAGTAAACCCATCAACAAAAGGGTATGAAAC

AGCATCAGTTCCATAAGAGCATTCAAATCTATGGTCTAATGGTGTTGAAAACGAAGCAAA

GGAAGGGTTTAGAGACGGATTAATGAGTAGATTTTCATCAAATGAGTCGAAGTTCCAACC

ATTTGGAAGAAGAAGGTCGTTTAAACCGGTGGATAAAGTGTTCCATGTGTCTTTTCTTGG

AGCTAGTAGCTCCTCAAGGAAACCAAGTTGAGAAAGCTCCATGTTTTCTCTCAGTTATAT

TTCTCCTTTTGCAAGCTATGAATAAAACTAATGAAAAACCAAAGAGAAAGAGTCTTGGGA

TGAAGTAGAAGAAAATAAGAAGAGTTTCAAGTTTGTTTAAATAGACAAGTTCTATTTTGG

AACATCACAACTGA

>LsbHLHR46

AGTGTTTCAACTGGATACTCTAAGCGCCCGAATGAAGCTTGCAAATCTAATCGCAAAAGG

CTTAAACCAGGAGAGAATCCAAGGCCTCGACCTAAAGATCGTCAAATGATTCAAGATCGT

GTCAAAGAATTACGAGAGATTGTTCCAAATGGATCAAAATGTAGCATAGATGCACTGCTG

GAACGAACTATTAAGCATATGCTTTTCTTGCAAAGCGTGACAAAGCATGCGGACAAGCTG

AAAAAGACCGGGGAGTCTAAGATTATTAGTAAGGAAGGTGGACTGGTTTTGAAAGATAAC

TTTGAGGGAGGAGCTACATGGGCATATGAGGTTGGTTCCCAATCAATGGTTTGTCCGATT

ATAGTCGAAGATTTGAATACTCCCCGTCAGATGTTAGTGGAGATGCTTTGTGAGGAACGA

GGTTTCTTTCTGGAAATTGCCGACTTAATCAAAGGATTAGGCTTAACCATCTTGAAAGGG

GTAATGGAAGCTCACAATGACAAAATCTGGGCACGATTTGTTGTTGAGGCAAACAGGGAC

GTAACGAGGATGGAAATATTTATGTCACTCGTTCGTCTTCTAGAGCAAACGATGAAGGGC

AATGCGTCTTCATCCAATGCCATTGATGACATGTTAGGTTACAACTCTTTACCCCAGAAG

GCCTAATAGTATGAAATCAATCACTTGTTACATGGTTTCTTTCAAGTATGGATGTGTTGA

GAGCAAGTACAGTAATGCTTGCCATGACCAAACATCAAATCCATTCCAAGATTTCAGGTG

GTCTTAACATTTTCTAACTGTTTCTGTTTTACATCACATATATGAAAGTTTCATATATAA

AACCATTTTGAAGGGAAATGAGTGTTTAGGTAGTGAAAATCATAACTGTTTTCTGGTGTT

GACCGACATATAGTGTATCATGTGTTGGCGCAGTTATCTTCGGATGTTTTTTACTTCACT

CTCAGATGACATGCATGCATTCGTTTTTTTAGCGCGATGGCCGGAAGTTGTGTAGTGATT

TATAACAGGTGTATAGATAAATATTGAAGGGTATGGAAAGAAAAGGTTCATCATGTGTTT

GTTTTAGCATTGCACTTTTTCTTTTGTTGCTCTTGTAGTTTTGGTGTAACTTGAGGTTGT

GAAATGGGAAGTGTTATTACATGTAAATGTAAAGATCTTAAAGTGTGGTTTGTATGTCAT

TGGAATCAATGAGGTGACACTGACAGTAGATTCCGATTCGTTTATATATATTTTTTCACT

TTTGTTTTGAATTTTGAATCAAGTGCTCCAACTATGAGTTGTTTTGAGCTACTGAATAGC

AGTATTACGTTGGATTTGAAACGTGCGTCTGGCCAAAAGTTATAACT

>LsbHLHR47

CTATTATTAAGTTTCCATTTGATTTTTACACATGTTTTGAAACAAGAATATCATCCACTT

GTGTGCTTATATAGCTGTTGAAAACAAATCAAAGTTTCTTTTCATTTCTTGTATATATAA

TAACTGTTGAAAAGTTAAATCATTCAAAGTGGCCAAGTTGGTTGAAAGCATGAAATTCCA

CCATATCCTTCTCTCTTATACATTGCTAATTCCTTTGCCTCAGATGCTCTTGCTCTCTGC

ATTGTTTCTAAATCATCTGCTTCTGAATTGAAGTCATAATAAGTACTAGCTGCAGTAAGT

TTCAATGAAAGAAACTCTACTTGATGCTGTAATGATTGAACATAGTTTATGATTTCATCC

AACATTACTGCCATTCCCATAGTCTTGTAACATCCTGGAACAATATTCTGCAAGCATCTC

AATTTCTCATTGATTTTCCCTCTTCTAACCCTTTCTGCTAAACTGTGACTATCAGTAGCT

TGACCTCTTCTTGCTCTAACATGAACAACTTCCTTTGCTTTTTCTTCTTCTGTTTCATAG

CTTTTCATTTTTTTTCCTCTTCCACCACTAAGTTTGGTTTTGCTTCCACTTTCAGAAACT

GCAGGAGTTGAATTTGCAGAACTAGTCTCTTGAAAATCCATCATCTTCCTCTTTTTACCA

TCACTAACTTTATTTTCTTGTTGAAAATTTTGAAGTGAAACTTGAACTGCATTGTTGTGA

TTATTAACATGATGATGATGAACAAGACCATGAAAATTCTCTTCCAAGTTTCTCGGAAAC

ACATGTTCTTGTTCTTGTTGATGATGTT

>LsbHLHR48

TAAGCTCACTCTTATTTTATTTTTCTCGACAAAAATTTTCATCTAAAGAAGAAAGAAAAT

GGCTAGGTTTTCTAAAAGCAATCAAGATGAAGAATTGTTGGATGATGATGATCAAGACCT

CTATACTGTCAACACTTCTTCCGCTCTCAACATCAACAACAATAACAATGTGAAGGTGGA

TGAACCTGGAAGAGGGAAAAGAGCGAACCCTCATCGTTCTAAGCATTCTGAGACTGAGCA

GCGTAGAAGAAGCAAAATTAACGAAAGATTTCAGGTTCTGAGAGATCTTATACCTCAAAA

TGATCAAAAAAGAGATAAAGCTTCCTTTTTGTTGGAGGTTATTGAATATATTCAGTTCCT

ACAGGAAAAGTTACAAATTTATGAACAGTCTTATGAAGGATGGAATCAGGAGCCAAGTAA

ATTAATTCCATGGAGAAATCATCACGGACCTGCCGAAAACACCATAGATCCTTCTCGAGC

TATTCCAAATGGTTCTGTTCATGAAACACGTAACAATGTCTCTCTGTCATTGCCTAAAAA

TGTGCATAACCCAATAGAACCTGACCCCTCCACAACGATTCAGAAGGGTTGCACCCCTGG

TTCATCCGCAGAAGCCGCTCCCCTGACAATGCAAATGCGATTGGACATGTTTGATCCGGT

TGTCAGCAGTGGTATGGTGACTCAACAGATGCTGGAGTTGCCTGTATCCAGTGCTGACAT

GGCTTCCAATCTCCAGCCTCAAGTATGGCTTAGCAAACCAAACAAGGACGACTATATTGT

TCCCGATAATACATTGAAGGAAGTAGAGGAGCTGAAAAATGATAGTGGGTCAGAGTCAGA

TAGCATCTCAACTGCCTACTCTCAAAGAATATTGGGTACTCTCACCCAAGCGCTTCAATC

CTCGGGTGTAGATTTGTTGCAGACCAGTGTTTCGGTGCAAATTGATGTTGGTCGACGTAC

AAATACTGGCTTAACTCCTTCCCAATATAGCTCAAAGGGTCATGAAAATCAATATATGAG

CAATGAAGCAATGGCATGTGATTACTTCAGTGATGACTCTGAACAAAGTCCAAAGAGGTT

TAGAAGAGAAGCTAGCTAGTGATTCTTCTTCTCATGTTATTATTTTTTGGTGTTGAGCTG

AGGGAAACTGGGATTGGGTAGGTCAGGTTATTTTTGTCATGCTGGCCAGATCTAGATAAA

ATGTATATTTCATGATTCTGGTTTTGAAACAATGACATTTTGAAATAAGCTTATGGAAGA

GCTTATTTACAGCTTCTTTACAACTTATTTGG

>LsbHLHR49

ACTCAATCTCCCTCATACTCATGCAAATTCCTATATGATCTTACGAATCTCATCATTCAT

TACATTCTTTTGAAAAGAACAACACAAGAACAACCAAAACACAAATCTACAACACAATGA

TCTTAAAAAATCCATGCCCTGCAAGTCTATACAAAATTTCACAGAAATAATGATAGTTGC

ATACACTTTACTCACATGCTCCTCGAGTATCAATAAATAATACACCCTTGCAACTTGTTC

TGAATGATCAAGATTTTACACACATTCTCACAATATGTGTATCTCACTTTCCAATTGATA

TTGTCTAACAAAGTCATCTTCTACTAAAAATTCGAAGAGCCAATAGTTAGGTATTAATTA

AGATCCTATACAACTAAAACCTTTATTTTCCTTTTTTTTCCTTCTATTTTTTTTTCCAAC

TTCAGTTAAATTGTGCAGTCAACAGGAAAATTCCAAAGTCTTCTGATGTAGAAACAAGGC

TATATATTCAACATACTATAACTCTGGTTTCAACCTTCCATTGGGTCCCAAATTGTCAAG

TGCTGTATTAGAATCATATCCCATTCCATAAAAGCTTTGGAAGTCATGGTCCAAAACATT

CTGAGGCAAAGGAGGGAATTGTGTTGATGAACTAGGCAGGCCAGCCAGGTTTCCCTGGAA

ACTTGAACTTGGGAATGGATGAGAGGAGCTTATACCAGGAACAAATCCACCGATTCCGAG

TCCTAAGCGTGACTGGAGAATATCCTTGGATAAGAGCCGCTCTACATCAAAGTTCATTTC

GGGGTTCACGGTGGCAAGTTTCATGGACAAAAACTCAACCTGTTGTTGCAGTGATTGAAC

ATAGTTTATTATCTCGTCTAGCATTACTGCTTTGCCAGTTATCTTGTTGCATCCTGGAAC

AAGTTCTTGAAGCAACCTCATACGTTCACTTATTTTTTCTCTTCTAACCCTTTCTGCAAG

ACTATGACTGTTGGTGGCTTGACCTCTTCTAGCTCTAACATGAATGAAATTATCTTTTGA

AGTGTCTCCACTAGGAGAGTTGTCTTTAGCTTGCTTCCCTGATTGCTCGACTTTTGGTTT

CTTTTCATGTTCTTTTGAAGCATCAGAGATCTTCCTGGGAGAATCTTTCACCCCATCACC

CTCAGCATTCTTGTTTGGACTGAAATTAGAATTTTGATCAAATCCTCTCTTTCTTCTATT

TCCACTGCTAGGTGCAGATCCTAGAGCTCCATCCTCATGAATTGAGTCCTCAACTTGAGA

TTGTTCGCCGTGAATTTGACCCCTTTGGATTCCAGCATCCTTGACGTGGTTATAATGTTG

TGGTGGAATTGGATATCCTGATGTGTTAGCTACATGGTCACCATGTTGGCCAAAGGAACC

AACCATTTCAGAGAAACTTCCACTTCCATAGGAAGGAACCTTGTGGATCATGCCACTGAG

ATTCGAGTATTGAACAAGGTGAGAGGTGCTACTTATTCCTTGATGATTCTCCAAAACAAG

AGGGTTATAAGTTGAATTTGTATTAGCAAACTCATTGTGAGAAACCATTGAAGAACCCCC

AAAAGTATGAGCCTGACTCAATGAAACAAGTGGATTATCCCAAGCTGAAGAAGCAAGAAA

AGGATGATGATGGTTAACTATCTCTGAAGAAGGCTTATTAGCCATTGACACAGAACTCAT

ATCCATTTCTGAAGTCATAATGTTCTCATTTCCATGTTGAAACTCCATGGCTTTTTCTTC

ACCACCCATCTCAACTCAACCAAGAAAACAACACCTTTTTATGTCTCTCTCTTTCAATAT

TTATATATCAATTGATTAGAACAATCCTGACTCAGCAGTTGGCTCAAGAAATGCATATAA

ATGATTAAAGGGAAAGCAAATTTTGACTAAGGTTGGCTCAAGAGATATCTTGCAGGACTA

AACAAGGAACAAATAATTTTGCAGCTTTATAGGACTGAGAAGAAGTTAAAGTAAAAGAAA

AAAAAAACCTTGTTCTCATAAAATGGAATATAATCTAACTCGGATACAGATGCCACCTAT

TCCTTCAAAAACCCTAGGATCCAATCAAGTCTAATGAAGAGAAAATAATAAAAATGGTTC

CTCTGAGAGACAAGAAAAGTGAAACCTTTCAGTCTTGTTAAGAGCATAAAGTAGATGACA

TAGACACTGAGATTAGATTACCCAACAACAGCTCATCAAAACTACAAAATAAAGCAGATT

CCCTTTTCTACTTTCTCCACACAGCAACACAAACAATCTTCAATTTTGGTTATTCTTAAT

ATCATTACATAGAAGAAGAAAGTTTCAGTGAGGCAATTTAACAAACAGTTGATGGGATTA

AACTTTGAAAGTGAAAAAAAATGTTGTTTCTACTATTGTTACTAATAAATAATGGAATTT

GGAATCCAAGAAGAAACAAAGACCAAAAAGCATAAAATAGTATACTCAAATCCTGCAATG

ACTGAGGAAACTTGAAAGTGAAAGGGACAAGATTTAAGAAGCATGAAATGCTATGTAAAT

TGGATAATGAAAACCAATTTCATGAATTGGGTAGTGACCATAAACACCAAAGGTGACATA

AAAATCTAACTTTTATAGATTACAGACAACACTATAAGCTAGAAGTCTAGAGAGAGAGAA

AGGGAAGAGAGAGAAAGTAGTTTGCTTTGTATGCTTACCTGCCCATGCTATGAAGTGACA

TGTATCATTTTAAAAGACATACTAACTACTGCCAACTGCTCCTGTTGGTAAAT

>LsbHLHR50

TGCATGAATAAAACTTAGTATCTGCTGAACGGAGCTTGCTATATCTTCTGCCGTGAACCT

TGTATCACTAGCCACCTTGACATTGAATGAATAAAGAACGGTTTGTTCAATAGTGGTGAT

GTTGGTATGAAGAATAATAAGCTGCATATCTTCAAGGGCAGCAATGGTCTTGATCAACTG

TCCAGGCCTTCTTCTCGATAGGATTTTAATCATTGCATCAAATCCTAAAACTTTTACTTC

TACATCAGCCAAACATGACTTACTCTCTGCAGTTTCTTCTTCAAGTCCACTTTCCATCTC

AACTATCTTCATCTGCTCATTTGGTAAAGGAGCTTGTTGAAAAAATGGAGCTTGTTGTTG

TGTTGAATCCCCTACTTGTTTTAACTGTGCTTCTCCAACTAGTCTTCTTCTCTTTTGTGA

CTCTAGACACTGAAGAAGTTGTTCCAATTCCCTCACAAACTCTATAGCTCCACCAATTAT

AGATGCTTGATCACCCCTTTGAACGTACGAACCAGGCATGAGAGATCTGAGGACACGAAG

ATGCTCATTCATTTGCTTCCTTCGATTCCTTTCAACAGCAATATGAGTCATGCGTTGACT

CTCAACTTCTTCGCTTGTCTTAACAGTTCTAGGTCTCTTCCTCTTCTTGCTATTCTCTTG

CACTAGTGCACAGTTCGTCTCTTGCACAAGCTGCACCGAGTTGTTCTCATCGGAAACCCG

AGTTTCTTCATGATCTCTTAAATTGTCTGCCACTTTGAATCTATCTTCATTTTCTCCTAC

CCCATCTTGATGCAACATCAAGTTTTGGTCTTCCATCTTGTTGTTTAAGACGGGAAACTT

GAGAAAATAAACCGGGTCGTCGTCGATCTCGGACTCTTGGCGGTTTAAGGCTAGTTTAGG

TCCAAAATCTGCAAACTGCATCACATCTGCAAAGCTCATTTTATCAAAAGAGTTGTTTGT

TGTACCATAAAAGCCTCCTGAGTTTTGTGTTTGAGGCAGGTAATTATCCTCCATTCCGTT

ATTGTTTTCACCGGAAGTTTCGCCGACACGATATTTCGTGAACTGCTGGTATTGTTGATC

CAGGGAGTAGTCAAGCGTGTTGAAACATGGTGGCATGGGTGGTGCGGAATAGTTATTATC

TTTCTCCATTATGAACAAGTTGACTTTAATGGAACGAAACCCTAAAACAAGCTCAGTTGA

TGCCCCACCAGAGTTTCTTGAGGGCCCTCACCTGTTTGGAAGCGGACAAAGATGAAAGAA

AGTAAAGTGTAGAAGAAGTAGAAGAGAATCATGAAACCCTACAATATTAGACCGGAAGAA

GAAGACAATGTAAATGGGTGTTTTTATCGAAGATGATGATTTTTATATAGTGTGTGTGGG

AGCTGGGTATG

>LsbHLHR51

CCAACATTAACCTCAAAACCATATTCATTATTAGCTTATAACTGTTGCTTACAATATTCC

AACATTAACCTCAAAACCATATCCAACAATAATATGCATAAATATTTCAATTTATATTGG

ACATGAAAACAAGTTCATAATTCATATATCAAGAAAAATCAAATTTCTCTCAATCCTTTA

TCAACTAAGCATTTGTTTGGAAGTGGGACCCACCATTTGACAAGGTGATGAACAACCTCT

GAGAAAAGAAGAGTAGTTAACATTTCCATTAGAAATCATTCCTGGGAACCCAGCAACATT

CATAGGCCTGTTTGCTCCAACTTGTTCCAACGTTTGAACTTGTTTCTTCAAGAACTTCAC

ATAATGTATTGCTTCATCTAACATTGAAGCTGTGTCCATTTTTGTCCCACCAGGGACTAA

TCTCTGCAAGATTCTTATTCTCTCACTAATCCTTTCTCTTCGGTGTCTCGCTGCTACACT

TTGTGGATCCTTTGAAATCTTCACGTTCTTTCTCTTTGGTGGTTTGATTGTTTCCGGGTC

TATATTAACCGGCTGCATAACCGCCATACGGAATATCATCTCTCTCATGGCCGCCATTGA

GTTCTTCTTATCGGAGTACGACGAGAACGAAGCATTGTTGATTCTCCCGGATGGAATTTG

AACTCCGTTGTGTTGAAGAGACGGTGTCATTGGTTGTTGAGGATGAGAGAACGGTGGTTG

TGGATAAGTTACGTTTGAGTTTCCATAAGGGAACTCGTTATGTGAGTTGTTAAGATTGAT

ATTGTGATTGTAGCTATAAAAAGGCTGGTGGAAGTCAGGAAACTTTTCCATTTGCATCAT

CATTGTCATCATGTCCATGCTGCTTTCATCACTTTCCAAGGTTTTCAGAATGTCCTCATC

CATGAATGTTTCTAGAGACAAAGAGGAATGGAAGAAGGGACGAAAGTACAGAAAAATAGA

GGCTGAGGTGAGAAAAGTGAAGTAGCAGAGTATGGGAAGAGGTGAGAAAGTAGAATATTT

GCAGTAAGGGGATGGAGAGAGTGCAGAGTGTGCCGGTTATCTTTGTAACCTACCAACAAC

CACTTAGGCAATTCTACTGAGTATAGGAATGCCTTTTTATAGCAAATTTGAG

>LsbHLHR52

CATTTATTAGTGTGTAAACATTTATTAGTACAATGCTATACAAAATTAAAAATAAAATTA

AATATTCGATATTATTGCTCTTCAATTAAAACCCCTTTAATTTAAAGAACTTTTCCATTG

TGGTGAGGACCTACATTATATTATATGCTTTGTATTAAGTGTCACACTCAACAATAGCAT

CATAGCATTTTTATATTGGCTTTGTTTGTTTCTACTTATTTTTAAGCATAGCCCTAAAAG

TATCCGATCCTAATCAATCTTTGCATAAGAAACTTTTGTCAAGGAATTTGATTTGTATCG

GCCTGCTGCATCCTCATACATTTGCAGTTTGATTGTTTTTCACTTAGAGACTTGAATTGT

TTTTGTAGTTCTTTAATGTAATCAACAGCCAAGTCCAACATGTCTGATGTGTTAGTTTGC

TTGTCCATGTTTGGAACAAGCTCTTGTAATTTCCTCATTCTTTCACTGATCCTAGTTCTT

CTCATCCTTTCGGCAATGCTTCGAGGATGAGTAGCGCAACCTCGTTTTGCTCTGATTTTA

CAAGGAACGGAATCCGGAAACTGAAACAACTTCTCCATTGCAATCATCTCAGATGAAGTC

TTCGGTAAACTCAAGTGATGTGATAATGTATGAACTTGATTTTCTAGTCCTCCACTCTGA

AAATCAGAAAACATTTTTTCGCTACCGATTCGCCCTCGTTTCAAGCCACTGAGATTTTCT

GAGAATGATTGAGTATCATTCCAAGAACTATAGGGAAATCCAGAACCATAATGCGCAGTA

TCACCATTGCTGCCTCCATCGCCAGGGCTAGTTGCTTCAATATCCTCGCTATCTATTTCA

GAGATTTGTGATAACACTCCCAAGGAAGATGCGTTTCTTGAAGGAAAGCTAACTTGACAG

GTCAATCGGTTAATAGACGGACTAACATTGCCATTACTTCCATTCATTGCAGCATAGTTT

CCCGTACCCTTCATGGCAGCAAACCCATTTTGAAATGAAATGTTGGAGAAAAGACCAGCA

GGTGAACTACCCTGCCTAAGAAGATTTGAACCAAAGCTCTTATGAGATGTTTCATGATTC

ATTCCCAATGAACCAACCAATCCAAATGAACGATCCATAGCAGAATTTGAAGTTGCAGCA

GAACCGTGTCTAGGATAGTGAGGTGGCAACCCACCACCACCACCGCCGCCGCCG

>LsbHLHR53

CTTATTATCAACTATGAAGTAACAGATTACAAACAAAGACATGTTTTGTCAAAATATTTA

AGTAAGAAATTGACGTAAATGGGTCTAATTGAAACTTTCAATTTTACCCAAAAAAAATAA

CCCTAGATTCTTGAAACAAAAGGGTACATGATGTCATGACGATAACATGGTCAAAAGTTG

ACCATGCAAAAAAGACTAAAAAACCCTTCTGCAAAATGAACCTTTAGAGCAAACATTTTG

ATTTTGTGGAAGTGATTATTATTGATGAAGAGCTTAAAATTTAATTGCTTTATGGTTTGA

TTGTAGTAGTGAACAAATTATGAAACTTGTTCACATGAAAGATGAATATGCTGAACGTAG

CTTTCTCACAAGTTCCTTCAATGTCAAGCAAAATCCAACATCCATCTGAGCAATAATGGT

AATATCAAGGGTGAAATTCCCAAAAGTCAAGGCACTGCTGTTGATGACTTTTAGATGAAG

TTTCTCAATTTCACTCACTGTTTTCTCTATAACTCCTTGACTTTTCAAACAGTGAAGTCT

AATGAGAACACTTCTTTCACAAAATCTTGCTTCAATTTCAGGTAATGTTTCATCAAATGT

ATTATCAGTATCTGAACAAGAGTCTTCAGCATCATAAGATAGTTTAGATTTCTTCACAAT

TACAACAGATTCCACAGTTTTCTTCCTTTTCTGTTCCTCCTCAAGAACACTCACTTTCTC

TTCCATTTGCTTCAAGTACCTTATAGCTTCTCCAAGAACAGAAGCTTTATCCATCTTCTT

TAGGTTTGGAACAAGAGCCGAAAGAGCTATGAATCGCTGGCTAAGCTTCTCGCGACGCTT

CCTTTCAGCCACAATGTGATCATGAGCTTGAGAAGGTTTACTAGGACGAGTCTCAACATC

TTTAGCCTCTTGAAAAGCCTTAAAGACATGGTTCTGATGATTCTCCGACAAGTGTGCTTG

ATTCATCAACATGTTTGCAATAGTAGTAGTACTACTATCGATTTTTGGACACACCATCAT

CTCAGACTTAGGCTTCAATAGTCCCAATTGATTCGCATAATTCGAATCAGCAAACGAAAG

AAGATTCGCATACTGTGTTTCTGATGTTTGAAGACTACTGCTGTTGTAATTCCAGCTGTT

ATTCTTGAGCTGTTTCGCTGCTCTTTCAGAAGCTTCCATGCAAGTTTTGTTGTTGAAGTT

TGGAGTATTTGACAAAAAATGTTTCTGTAAAGCACCTCCAAAGTCAGAAGCACTTAAGTT

AGTTGTATCAATAGAATTTAAGTGATTATTCCACTGATGTAGAAAGTTAGGATCCTCTAT

TATTCCCAATTCTGGTAACCCTCTGATTGATGAAATTTCCATATGAGAACTCATTTTGGA

TCAAAAATCAGAAGCAAAAGTTAATCAGTTTTTGATAATTCAACTTAGAAGATTTGCATT

ATGATATATGGTCACGGGTTTGGATGTGAAATTGGATTTGATACAAGAATAGAAGGCTAT

ATATGAATGAAGGACATTATAGAGTTGATACCAATTTTATATTATAGAAGTTATTTCAAG

ATGACAAATAAATAAGTTCAATAATTTGTTTTTTTTTTGGACTTTGGAGGT

>LsbHLHR54

TATTACAAAAGAAAGAAAGAAGCTATACACCCAATTATTTTTATCTAAAGTGATAAAACA

ACGCGTGATTAGATTACAGACCTATGTGTATATATAAACAAAGTAGGCAAAGCAAAAGAC

ACCCAATTAACCAACCACCTTAAATTTCCTTCAATCAATAAAGCATCATATATATTCTCT

TTTCCTCGGTGTCTGTTTCCAGTTCAATTTCAAGTTTAAAACAAGCATGGAGAATATTAA

TGATGAAGAGTACAAACATTACTGGGAGACCAACATGTTCCTCCAAACACAAGAGCTTGA

TAGCTGGGGATTGGATGAAACGTTTTCAGGATACTATGATTCAAGCTCTCCCGATGGTGC

AGCTTCATCAAAGAACATTGTTTCTGAGAGAAATAGAAGGAACAAACTCAATCAAAGACT

CTTTGCACTTAGAGCAGTGGTCCCTAATATTAGCAAGATGGATAAGGCTTCGGTTATTAG

GGATGCAATTGAGTACATAAAACATTTGCATGAACAAGAGAAGAAGATTGAAGCTGAGAT

AATGGAGCTTCAATCTGGGATGCCAAATAATATTATTAATCGAAGTTGTGATTTTGATCA

TGAGCTTCCTGTGTTGCTGAGGTCCAAAAAGAAGAGAACAGATCAATTTTATGATTCTGT

GAATTCAACAAACTCTCCAATTGAACTTCTTGAGCTTAGGGTAACATACATGGGAGAGAA

TACAACAGTTGTGAGTTTGACATGTAGCAAAAGGACAGACACTATGGTTAAATTGTGTCA

AGTCTTCGAATCTTTGAATCTTAAAATCATCACTGCCAATATCACTTGTTTTTCTGGCAC

ACTTTTGAAGACACTCTTCATCCAGGCAAACGAGGATGATAAAGATCTATTGGAGATAAA

GATCCAAACAGCCATTGCATCTCTAAATGATCCTCTAAGTCCTATGAGCGTCTGATGAAG

TGCATATATATAGTATTTATCATTTTTAAATCGTTAGTATAGAAGTGTTGAGTTGAAGAA

CCTTGGCTTTCATGTTTTTATTAGAGTTCCTAATTATTGTGTTATTTACTACCTAGTTTC

TTGTCCTATTTCTACTTCCTACTCAAATTTCCAATTATTGAGGAGCTTTATTGTCTTGAA

AAGTGTATCTTTAATTGAATATCATATAAATGAATGTTATTGTAAATGTAGAGTGGAGAG

GTATGTTATTGTAAATGTGGAGTGAAGAGGTTCAAGGTGTAGTTGGAACCTGATACACCT

GCATCGCAAAATTGCAATAGAAACTTATTTGAAAACCTTTGATTGATAGAGCAAACAAAA

ACAGAAAAAGATAGAATCAGTCACATCTAGAAATAAACCAGGCTCTTCCTGGGAGATTTA

CCG

>LsbHLHR55

AGCTTTATGTATCTAAACTAAGTACAATGTCATAAAACAAGTTCATTCATTGTGTTTGGT

CAAAAGATTGAAACATCCTTAAATCAGGTATCAACCTAGCTATCTACAAAAGTTCCCAAC

ACATGTACAACCCAAGATTCACATCACATGTGAACATGTGAATATACCAACAACAAAAAA

ACAAGTTTTATAAGAAATCTCCTAAAGATGAAGAAGAACTGAATATAGAAATCCTCCTTC

TCTTTCTAGTTTCTAAAATATCTTGTGAAACAGAAAATCTATCAAGAACAGATCGAAGAG

CTTGATGAACCGATCCAGCGAGAAACTGTCGATACTCGGCATCTTCAAAATTCTGTTCTT

TGCAACTAATTATGACAAAGACATTCTTCATTCTACCTCCTAAAGTTGCAATCTCTGCCC

TTATTATCATAAGCTGAAGTTCATCAAGTGCTTGTCTTATATCAGACAATAAACCTGGTT

TGTATTCACAACATAGTGATGCTCTAATTGAATAAGGGAAACCATTTAATCCACCTTCTT

GTGATTCAACTCTTATCTCATCACTGTCTTTTGGTATCATTAGTCCTTCACTTGCTTGTG

CTTCTTTTGTTTTTAGCTCTTTCAAATGTGTAATTACCTCAGCTAGTAAAGATGCTTTGT

CCAACTTATTAGCACCTGGAATAACGGTACGAAGAGTATCAAGATGTGAATTAATTCTTG

CTCTTCTTCTCCTCTCAGCTTCACTATGATTCTTCAAAGCTTCAATAGTTCTCTCGGGTG

AAACACCTTTGCGTTCCAACTTAACGGATGCTTCCACAAGCTCTCCACTTTCTCTATCCA

AAACAAGAGAAGAAGATGAACCATACTCATTCATTCTGGTGGAAGAAAAATCCTCCATTT

CCATACACAGTTTCCAAGAAGGTTTTGGAGAGAGGACAAATGGATAGACAAGTTAGGAGG

AGGCAAAGGTTGTTTTGTTTGGGAGAAAAGAAAGTGAAATAGAATGATGAAAAGGGAAAG

GGTTGGGGTTGGAGAGTATTTGAAAAGTGAAGTGTGTGTCAGATATGATATGATGATGGT

GAGTGAGTTTGGTGAGATCCAGAAAGAGAATGGTGATGTTGCTGCTTGCTGTTTCTTCTC

TTAAAGATTGCTTCTTATGGTGGGGGACTACATCCACACACT

>LsbHLHR56

CATTTCAGTTACATAAATAAAACAATGGACATTGTAGGAACAATGTTTCTGATAATTAAA

CTGTGTATACTATAGACAAACATAAATTGCTTGTCCTATACACAGGTGTGTTAATACTAA

TAGAATCTAGCTATATATTATTCTACTTCTGTCTAAATGCCACTGCTTTGTTCTTAAGAT

GGTTTTTCACCTGCACAACAAAAATTTCTTACGAAGGTTTAGAAATTGGTTCCTTCTCTA

TGTATGATATGCCTATATGGACAATGTTGTTTGTGTTTGTAACGGGATGGATTCGCCGGA

AAATGCTGCAATCAACTTGTCCTTTGTGAGTTGTTCAGATTCTTCGGACTTAATTACAAA

TGTATGGTAGATAGTATCGTTTGCTGCAGTGAGTTTCGACTCCGCTACACCGATTTGTGC

ATCTTTGAATGTTTCGATTACTTTCGAAATAGGATGAGTATCGATAGGACAACTCACCTT

CACAATTACTTCGTCCTGTGAAGCTTGGATGTCAACGTCGTGAGGAGGAGGAACTTGACG

GGGATTTTCTGATCTTGTGTTGGATGATCCATCCCTCGAAGTGCTGCTTCCGAATGTCTC

TTTGTCAGACTCCATGACCTTGAGTTTCGCTTGAAGCTCGTTGATGTAAGCAATGGCATC

ACCGAGTAGAGATGCTTTGTCCATTTTGGAAATATTCGGCACAACAGCTCTTAAGGCGTA

AAAACGTTGATTAAGCTTCTCGCGTCTTTGTCTCTCCGCCTCCACATGATTGAGAGGCTC

GTCCCTACCACTTGCAGGCTTCCTTCCGCGCTTCCTCGGCCTCCTTTCATCATTAGCCTG

ATCCGCGTCAACATCAGCAGCAACAAGTCCCGATTCACCAATAACCGGTCTCACAGAATT

AACCCTCGATTTCGTTCCTGAAAAATCAATTTGCATTTGCATTTGAACCTGCTGCCTTTG

TGGTTGATAGTTGCTAAAACCCTTTCTAACACCATGAATAGAACCAAAAACTTCACCTTG

ACCTTGATTCTTTCCCCAACTAGTAGAAGCAGAAACACCATTTCTAGAACTAGGAAAGTT

AATACTACTATCCATTTTCCTAATAGCAAGTTTCTCTCTAAAATGTGAACGACCATTGTT

CAAATTCAAAGCAACCTTTGGTGGAGGAACATGAACACCATTTGAAATTCCACTCTCATC

ATCTCTTCCCTCATTGATCACACTAGGACTAGGATACAACGACGATGAGGATTGTGTGAT

AGAATTCGAAAGTGAAAACAATGACTTAACATTGTTCAACAATTCAAAGCTTTGAGGCAA

GATTCTAACGGAACCCAACTCAAGAACGCCAAAATCAGTAGGTAGTAAAACAACAGTTTG

AATTCCAGCAGATTTAGCCAAGAAAGAACGAACACAGTAATCAGAAACAGATTTCAACCA

CAAATTTTGACCAGAATCAAAACACTTACCGGGTCCACCATACCCTTTAGGAAAAGAAAA

GTACATAGAAGCTAAAAAAAACATCTCTGTGTCGGTTACACGGTCAAGGCCAAAAGCGTA

ATTGTCTTCATCAGAACCACCAAAAGCT

>LsbHLHR57

ATAAAATAAGAAATGCTGACTAGAAATATTGAGTACTAGCAACCAGCGAACAGATCACAA

ACTTCAAAGACAGTCCAGTAATTAACAGTCTGTTCAATGATTTTACAATCAGAACCCAAT

CAATCACCTAAGATTGAATGACCACTATAGTTAGTATACACACTGATATTAATTAATATA

TTATATGGAAATAGCATTCGAATTCTTCTTCCTTGCTAGCCTGCTGGCTGAATAAAATTC

AGGTTTCCCAACTTACTCTGACAAACAGAGGACAATGAAATCCTGCTGAATGTTGGGCCT

GCTCAAATCAAGGCTAATTTCTAAAACTATATTTTTTATATACACTACTAGACTAATTGT

TACTAGTACCACTGCTTCTGTACCTCTTCATAGCTCCATCTTCATCTGATTTGAGTGCAG

AGATACTGAATTTGCCGATGAGTCATAACTCAATAGTGAGTTTTCTGGGGTCATGAAATT

AAGGGTATTTTCTTCTCTCCCCCAAAGTAGTTGGTGGAATGCTTCAGATTGCCCTGGTTG

CTGAAATTGCTGTCTGTTTCCATTGTGTGGGATTTCTGGCCACACGACGGGTGCAACTGC

GGTAGGGAAGTTACTGTCCATGAGAGACACTCCTTCTGTAGCCAACATGCTGTCAAGGTT

GAAATCAATTATTGGATTAACTGCTGCAAGTTTCATTGATAAAATCTCTACTTCATGCTG

TAGAGATTGCACATGGTTGATGATTTTATCCAACACCAATGCCGTTCCTGATATCTTGTT

GCAACCAGGGACCAGCTCTTGCAACAGTTTCATTCGAGCATTTATCTTCTCTCTCCTAGC

CCTTTCTGCTAAACTATGGCTATCAGTGGCTTGACCTCGACGAACTCGAACATGAACATA

CGGAAGTTCCTCGCCGTTGCCGGAATTCTCGTCGGCGACGCTTTTGCTTTTCTTGGAAGA

TGCTTTAACCTTCTTCTCTGGCTCTTTCCTCTTCGCGTGTTTGGAATTCTTGTTCTCGAC

TGTGCGATCAGAGACGCATCCACCTTCCTCGATTTCCGGTAGTTCATCTTTCACCTCCGG

CAGCGGCGAGTTTGCCAAATTCTCACCGGCGAAAACGGAGAATCTCGCGGCGCGTTCGAC

CAACGAAGTGTTGGAAGGGAAGATCAGATTATCTGTGTCAGAAGTTAGAGGATAGGGTTT

TGGGCTAATGACATGGCGAGGCGGTTTTCCGGTGATTTCCGGTGAGTGGAGAAGCTCGAC

GACCTGCGTCGGTGGTAGCTCGAGAAGTGCCGTGAAAGAGCTTGTGGTTTCCGACGCCGG

CGCCATGATTCCCTGAATTTCTTCGTTGAATTGAATGGATTCAAACGCGGTTTGCTCCAT

GATCGAATTGAACCAAACCGAACCGGGTTTGGGAAAGATGAACCGGGAGGGAAATGGTTT

GGGAGGAAGTGATAGAGGAAAAAAAGAGAGAAAGAAAGAGAATGGAAAATGGGGAAAGTA

AAGAGTAGTGTAGTAGTGTGTGTTTATTTAATAGTGGTCAATGTGCTTGTTTGTGATATT

GTTTCTGAGAAGGTTGCTTCTTTCTCTGCCATTTCAAGTAGCAAGAAAGCAACGTTGTTA

CGCTACGGTTTGTTTCGTTTGGTTATTGCAAATAAACGGGAATTTCCGTACTGTTGTAAC

GGAAGTGTTGAATATGATGTTTGTAGTAAAA

>LsbHLHR58

CCAAAGATTAGCAAAATGGATAGGGCTTCAATTCTTGGGGATGCAATTGAGTATTTGAAG

GAACTTCTGCAAAGGATCAATGACCTTCATAATGAGTTGGAATCAACACCTGCTGGATCT

TCACTAACACCTGTTTCAAGCTTCCATCCGTTGACACCGACTCCATCTTCGATGCCCAGC

CGTATCAAAGAAGAACTATGTCCCAGCTCATTGCCAAGCCCCAATGGACAGCCTGCCAGG

GTTGAGGTTCGGTTACGGGAAGGAAGAGCCGTAAATATCCACATGTTCTGTGCTCGCAAA

CCTGGTCTGTTACTTTCAACCATGAGGGCTATGGATAGCCTTGGATTAGACATTCAGCAA

GCTGTTATAAGCTGTTTCAACGGATTTGCTATGGACATTTTCAGAGCCGAGCAATGCAAA

GAAGATCAAGATGTTCATCCGGAGCAAATCAAAGCAGTACTCTTGGAATCAGCTGGTTTC

AATGGCATGATGTAATTAGTTTAGGCAACAAACAAACTAGTAATTTTCATTTCAAGTTAT

CAGTTTTTACATTTTAATATGAGAACAATTCAAGTGTATGATACTGGCTCTTGTGAATTC

TGTTTAAGTTGAATACCTTGGATTACAAGGAATTTTCAGGTGAATGCATGGATTTTATCT

GCA

>LsbHLHR59

GCTCTCTCTCTCTCTCTCTCAAGAATGAACAACAACATCAATGGTTGGGTAGAAGAAAGA

GAAGGTGAAAACACCACTACTTCACCTTCATCTTGGCCTAATACCAACCCAAACACTCCT

TCACCTGCTTCTACTTTACTCCAAAACAAACAACATTTCGCTGCTTTCAAAACCATGCTT

GAAATCGACGATGATGATGAATGGTACATGAAAGACATGGCTTTTCCGCCTAACTTAGAT

ACCGTTTTACTCAACACCGTTGATTCCGTTTCTTCCTCTTCGTGTTCACCTTCTTCCACC

GTTTTCAACGCACTTGATCCTTCACTGTCCAATCTTCAGTATCTTCTTCCTCAAAATCAC

AAACCCGTTGACACCCTTTCTTCTCTTCTCAACAACCCTTTTGAGATTCCATGTGAAGCT

GGTTTTCTTGAACCTCAAGGTTCAGCATTACCTCCTTCTCCAAAAATGGGTAGTTTGGTT

GATTTCACAGCTACAGAGATGATGAGCTTGCCCCATTTGGCTCAAACTAGCAATGGCTTC

ATGGGGTTTCAGAACTCGGAAGAGGGTTCTGGAAAGTCGCTGTTTTTGAACAGGCCCAAG

GTTTTGAGACCGCTTGATTCTTTGCCTCCTTCTGGAACACAGCCTACTCTCTTTCAGAAG

AGAGCGGCACTCAGAAAAAACATGGGCAAGGGTGAAATTGGGGAAGGGAGTGATAAGAAA

AGGAAGTTCAGTGGTGGAGATGAAATTGAAGATTTGAGTTTTGATGGGTCTGGTTTGAAC

TATGATTCTGATGATTTCACTGAGGGTAATCGCAAAAACCGTGGAAATGCTTCCAATGGT

AACAGCGGTGTAACTAATCAGAAAGGGAAGAAGAAAGGAATGCCTGCTAAGAATTTGATG

GCCGAGCGTCGCCGCAGGAAGAAGCTCAATGATAGACTCTATATGCTGAGGTCTGTTGTT

CCAAAGATTAGC

>LsbHLHR60

AATCACTAAAATAAAAATTTTCAACAACAAAAACAAAAAATGAAAATTTATTTCATAGAT

GATGATCACTCCCTTGTTGCAAAATAACTTTCATTTTAGGCCATTGAACATGACACTACA

CATCTAAACAGCAAACAAGATAAGCTATGTCAGAAATATGTTTTCCCATTAGGTTTCAAC

CCTAATTTGTTTAACCTTTGTGTTAATAACCATTCCAAACAAACTACTCTAGCTAGGAAC

AAAAGCATAATCAACAGCTACTATAATACTCTAAAGTTAACATTAACATTTAACACACCA

AATTTACTAAAAAAACAAAAACTCAAACCATCCACAAATACTAGTTGAGGGTTAATTTTG

TCATTTCATTGAAATCATGCACCCTCAGTTTAACAGCATAGTGAGGCTTCTTCCTCAATT

CTTCCAAGTAAATGATGAACTGCTGTTGCAATGCCATCTACTGAACCAAGTTGAAACCCT

TCTTCAACCTTGGCACTGATAGAGTAGAAAACCAAAGGTTGTATGGTAGTGACATTGAGA

TGAAGAATGGAGAGAAAGAGTGTTTGAAAACCAGCAACCAATTTAGTTAACTGTCCTGGT

CTTGTTTTAGTGAGAATTCTAAGATTTGCATGAGTCTCGATTAACGTAACCTCGATATCG

GCTATGGCAGCTTTGGTTTTGGATGTGTATTTGTTATTAGTAGTTTGAGACCATGTGTAT

TGAGGATACACAAAAAATTGTGCAAATGGTGGTTTCATATTATTGAGCTTTGAAACATTG

GATGTTTCTTCAATGTTGTTTTGTTGTAATGCTAATTCTTGTTGGAAGAGTTGTAGCTTT

CTTGCTTCAAGTGATTGCAAAATATGTTCTAGTTCCTTCACAAACTCTATGGCTCCACCA

ACTATTGAGGCTTGGTCACCCCTTTGAACATAGGATTCAGGCATGAGTGACCGAAGAACA

GCGAGATGTTCATTCATTTGTTTTCGACGGTTTCTCTCAACCGTAATGTGTGTGATTCTC

TGAGTCTCAGCTTCTTCTTTATTCTTACAAACTCTTGGCTTCCTTCTCCTCTTCTTCCTC

CCTTCCACAACTTGATGACTACTCATTGTTTCATCTTGCTCCATAAACTCTCTTTTTCTG

TTATTCTGATCAAATATTACACCTTCATGATATTGTTGCTGCTCATAGCTCATTGCATTT

TCCAAGAAACAACAAGACTCTATTGTATCATGATGATAAGAACTAGTACTACTAAATGGA

GTTGCAGAAATGGTGTCATAGATAATAGTTTCCAAAGCCATTTTACTCTCTTCAACTCAA

CTATGTGCTCATTTATAGCAAAATATATATAAAAGTTTGTGAAGTTTTTTGATGAGTGAC

AAAGAGGAAGAAGAAGAAGAGAGTAAA

>LsbHLHR61

CCAGTTACATATGAATACATATTTATTGATAATCTGATTCTTACAATGACTCAATTTTCC

TAACAAGGGGTAACTTTCATACCTGAATACATATACTGCCAGTTACATATGAATACATAT

CGCGAAAGCAAAATCCCATCACAGCAGATGAGCATAGTTTTAAAATCAAAACAACAATAA

GATTATTTCAGTTGCTACATTATTGATAATGCAAATGGAGATTATATGTAATTTTTACAA

AAGATGTCGTACAATGTTCTACACAATGACTAGGTCGTCAATAGAGCGCGGTGACCAACC

CCTATGAATTTTTGTATTTTTAAATGAAAAACAAACCATACTAATTGCAGTGTCATTCTC

CACGAATCGTCCTTTAGTTACGATGTTTTATCTAAACCACCAGCTAGCTGCATATGTAGC

CATCCTGGCTGTGATCCTTGAGCATAGCCCCTTCTCGCTTGCGATCCAAATACTATTCCA

GAAAGATCAATTGGTTGAGCACCAACATCTTTTAAAGGAAAACATTCAATAGAAGGTTGC

ATATTCAATCTTGAATTAACTGCTTCAAGTTTCATTGAAAGGAACTCAACTTGATGTTGT

AGTGATTGAATATAATTGATTATCTCATCAAGCACAAGTGCTTTACCAATTACCTTGTTA

CATCCCGGAACTAAATCTTGTAGAATTTTCATTCTCTCACTAATCTTTTCTCTTCTCGCT

CTTTCAGCGAGACTGTGACTATCGGTGGCTTGGCCTCTTCTTGCTCTAACGTGAATGTAA

TCTTGCTTGGGCGGCGGCGGCGGCGGTTCAGAAGAACGTTTCTCATTCTGTTGTTGTTTC

TTTGGTGAAATTGCGTTTGGTTTGGATTTAAACGCAGCGTTTTGATCATTCTCCGGTAGT

GCTAGTTTGATATGCTTAGTGGCAGAGTCATTTTCCGAGAGGGAGTGGTTTCGTTTGTTT

TGGGATGGAAAGTGAGGCCAAATTTCTGATAAAGAAGGGTTAGCAGAAGAGAAAGTGCAA

TCGTTGATGAGAGGATGCTCCATTGTGTTGATAACAGTAATAATGATTATGATTTATTTA

TTGAGGTAGCAGTAGTGGTGGTACTGGTATAGTACCGGCAATTATGAGTTTGATGGTGAA

ATGATCGTAATTTTTTTGGAGTAGAGTTTGTCTGATTCGAGAATTAGGCTATGCAGTTGC

GTGGGGAAGAGAAGAGAATTATGAAGAAATTGACGGAGAGAGTGAGATGAGTAGTGAGGT

TAGTTTGTGAGAGTATTGAGGAGTGAGGTTCTTATCTAATTAT

>LsbHLHR62

AACCGATTAATGTTTAATCCTACACACGATGATTCTTTGATATCTTAAGTTATCATCCCT

AAAATTAGGATGCACGTTTTAAATAAGACAACTACAATCAACTCTTTCAGAATAATTAAA

ATGGATCTACACACACGTAATGTAAATTAGATACCACAACTGTAACTGGAATGAAACTGA

ATTATTACAATTAGTACTTGCTATTGTGTTTAGATCAATGTTTGGAGGGTTTTCACGAGG

TCATCCACTGTAATATTATATCCATCACCCATCTGGGCAACAATGGTGATACCAAATGTG

GATTTCCCAAATTGCAATACACTGCTACTCTTTACTATAAGATGAAGTTTTTGAAGCTGG

GTCAATATTTTGACCATGACACCATTTTGTTTCTCACAATGGATTGTGATGAGAACTTCC

ATTTTTGACACTTTTGCTTTGACCTCAGGAAGAATATAAGTACTTGTTCCACTATTGTTG

GGTTTAGCATTTTGTTCGAGCTTTTTAACACGTTCTTGAAGTTGTTTCACATAGCTTCTA

GCTTTGTCCAAGATTGAAGCATCGTCCATGATCTTATTCGAGTCAGGAAGGGTGGCTGAA

AGTTCAAGCATCATCTCTGTCAAATTTTTCTCATTAGAATGGTACTCATCATCAAAAGTG

GCATCATCAATTAAACCCAAATCAGGATCAGAAGGCCAATTTTCTCCAGATTCCTCCATT

AATTTTGTGGTGAAATGCTATTATAATGATGTGGAAGAAAGTATATTGTTATAAGAAGTG

GAGTTGTTCTTGTTCTTCTCTTGTC

>LsbHLHR63

TCATGTCTAAGTAAGTATTTCATAGTGCAGTAGTTGAGATCTAAAATCAAGCCTCTTGCC

TACTTTTCCATTTGGAAGCAGAAAGTGATCACACACCTGATTTCTCAACTTTTTGCATAT

GCTTGCTTTCAATTTCCCACTTTAACTTTTCCTTTTCCTTAATTGGTGGTACCAAATCAA

CATTTCATCTCCCTAATGAACAAACTATATATCTAATAACTATATAATAATTATTCAAAA

CCAAACAAAGAATTGCCCCTTACTATGACCCTCCTAATAAAAAAAAGGAGGTGAAGACAA

AATAAATTTTCACTATACGAAATGTGAAGGCAAAAAGAACCTTTGCCTCTTACTTCTAGT

AGGACAATTAGACACCATCGATGATGAAGCAATTTTGGTCACTGCTGATTTAAGGGACTG

TTTGAGAGTGTTAATGCAAACATTCTCTTCACTGTCCTTAGAACAAAGCACCAATATGCT

TTTGATTCTGCCACCAACACTTGCTATGTCTGCTTTAACTGTTGTAAGTCTTAGCCCTTT

GAGGACTTGAATTAGTTCAGAGAAAAGTTCAGGTCGATCGTCGCAGCAAACAGAAGCTTT

GATGATTATATTGTTCTTGAATTTGTCGACTTTGTTAGTGCTGGTTTCGTCTTCAATAAC

ATGGTTGTAGTCTATTGATACTTCGTCGATTTCAGTTGGAACGTTGATGACTCTGCTAAC

ATCCATTGCTTTTCGCTTTAGGTCTTTGACATGATCTATTACACTCCCTAGTAAAGCTGC

TTTGTCCATCTTATCAGACTTAGGAATAAGTTTTCTGAGATTAGCAAGCTGAGTATTGAT

TCTATCTCTGCGTCTTTTCTCTGCTTGACTATGACTCTTGGAAGCACTTGCAGCTC

>LsbHLHR64

TCAGATGGGAGATGGATACAATATCACTATTCTGAGTGTTTTCACTAGTTCATCCATTGT

AATATTGTATCCATCTCCCATCTGAGCAACGATGGTGATATCAAAATTGAATTTTCCAAA

TTGCAAGACACTACTACTCAGTATAAAAAGATGAAGATTTTCAAGATGGGTAAGTATCTT

GAGCATAACACTCTTTTGTTTCTCACAGTGGATTGTGATAAGTATTTGCTTTTGTAACAC

TCTTGCTTTCACATCAGGAATAATATTATTACTTGTTCCACAATTGTCGCTAGAAATATT

GTTGGATCCAACGCCTTCCAACTCTTTCACACGTTCTTGAAGTTGTTTTACGTACTTTCT

GGCTTTCTCTAGGATCAAGATGTCGTCCATCTTCTCCAAACCAGGTATGGTGGCTGAAAG

TGCCAGCATTTTCTCTCTCAGGTCCCTCTCATATCTGTCGGTGGAATCTCCAAACTCATC

ATCATATGATTCAAATTCTCCATCTTCAAAAATAACGTCATCACAAATTTCCAAATCAGA

ACTAGAAGGCCAATTTTCTCCTGACTCCTCCATTAATTTTGTGGTGAAGATATATACATA

TATTGGTGATAAGATAGTGAATCAATTGAGAAGAGAGAATGAGAGAAATGAAAAAATAAA

TAGAAGAAAAGCAATGAACCAAACTTCATTGATAAGTATTC

>LsbHLHR65

AGCAAGAAGAGGTCAAGCTACTGACAGCCATAGTCTTGCAGAAAGAGTTAGAAGGGAGAA

AATAAGTGAAAGAATGAAGCTTCTACAAGATCTCGTACCGGGTTGCAATAAGGTTACTGG

AAAAGCACTTATGCTTGATGAAATTATAAACTATGTTCAATCATTGCAGCGTCAAGTTGA

GTTCCTATCTATGAAATTGGCTTCTGTCAACACAAGGGTGGATTTTAGTATTGAAAACCT

AATCTCAAAAGATATATTTCAATCAAATAATTCTTTGGCACACCCAATATTCTCTCAAGA

TTCCTCAGCACCATCCTTTTATGGGCAACAACACCAGCAAAATCCAGCAATCCACAATAG

CATTTCTAATGGAACTATGCCCCACAACTCATTGGACCCATTAGAAAATGCTTTGTGCCA

AAACCTTGGCATGCACTTATCTTCACTTAATGGATTCCATGAAGCTGCCTCTCAGTATCC

ATTAACATTCTCTGAAGATGACCTCCACACAATTGTTCAAATGGGATTTGGACAAACTGA

CAATAGAAAATCACCAATACAATTTCAGAATTTGAACGGCACAAATCAACTACCCCTATG

AAATTAAGCTTTCATTACGCATGAATTAAAGCTAAAGAGGAATAGGAAGATTGCTGTTGT

ACACATCAACAACAAATTTAATTGGAATAACCTCATCAATTATTAAGCCATTATTGTTAT

TGTTATTATTAGGTCTATGATTTTTAATTTTTGTTATCATGGAGGTTTTTTTTTTTTTTT

TTGTAATAAGTTGTATCAGTGTATATGTGATAATTGGTTTCGATAAGAAATGGTCTACAC

AAATTCTGTTTGTTAGTGATCCAATTTGAGTATCAGAGTTGTCCCCCCAGCAGTTCATAC

ACAACA

>LsbHLHR66

GATTATTAATTAATACTAAGTTTAGTAATTTTTTTGTTGAAGATTAATTGATAATTATCA

AGCATGGCTGAAGAACAGTTTCAAGGTAGTGGAAATTGGTGGGAGACACCAGCTCGAAAC

ATGAGATTTGAAAGTGTTGAACAACAACAACAACAACAATCTTCCTCGTTTGGTGGTTGG

CAGCAGCAGCAACAACATCATGATACTATGTCTGCTTCCGGTAGTTCTTCGATTGTTTTT

CATGATACTACTGAAAAGCTTCAACCTTCAGATTCTTCTACTTCAAATAATGACAACAGC

AACTTGCATATGATGAATTTGGGTCTTTCCTCTCAAACCATTGATTGGAATCATGCATCT

TTAATAAGAAGTGACAAGGCTTCAGAAGGAAGTTTCAGGTCTATGTTGCAAGAGAATTTG

AACTCATCAAGCACAAATTTCGATGAAGAAACCGGTGGAGTTGGATTGAGTAATTGGAGG

CAAGAAAAGTTGTTTTCGACAGAATCTTCTAACAATGAATTCAAGCAAGTAAATAGAGGT

TTCTCGTTGGATCAAACACAGTTTAGTCCTCAATATAGTTCGGGAGATAGTAACATGATA

AGCCAAATGGATTCTTCAGCTTTATATGGAACTCCATCAATTTTACAAGGCTCATCCATG

ACTAGTTTTCCTTATCCAACAAATAACTATGGATTATTAAACTCTAATAATGAGTTGAAT

ATGAACATGCCTTGTAATAATTGGTCTAATAATAAAGTTCCTCAGTTTTTGATGAGAACT

TCACCTCCAAAACAATCATCAACAAGTAACCAGTTGCATTTTACTAATAACACCCCTTTT

TGGAATGCTTCCGAGGCTCCGAATTCTATTAAAGATGTTAGGTCAAGTTTCTTTCCCTCT

TTGCAACCTCAATTCTCCACGCCAAATTTTGATTCACACTCGAAGAATATTAGTGAAGTG

AATACTGTGGTGAAGAAAAGTGGGAGTGAGCCAGCACCCAAAAGGACTCGTAACGAAACT

CCATCAACTTTGCCAGCTTTTAAGGTCAGAAAAGAAAAGATGGGAGACAGAATCACTGCA

CTCCAACAATTAGTGTCTCCTTTTGGAAAGACTGATACAGCCTCAGTGCTTTCTGAAGCC

ATTGAATACATCAAATTTCTCCATGAACAGGTTACACTTTTGAGCACCCCATATATGAAA

AGTGGAGCTCCATCAGATATTCAACAGAATTCGGGTAAATCAAAAAAATCCGACGGCGCA

AAGCAAGATCTAAGAAGTCGTGGTCTATGTTTGGTACCAATTTCAAGCACATTTCCAGTG

ACTCATGAAACAACGGTTGATTTTTGGACACCAACATTTGGAGGAACATCAAGATAATTA

ATGAAACCAATACCCTACAACTAGGGCATGGTTGTATGTTCCTTATCAAACCTAGCCAAG

TTGGGAATGATAAGATAGAAGAAAAGATAAGAAACATGAAAAAAGACAAAGAATGCCCAC

TTAAATGTGTAAAGTTAGTTAGCTCTGATGAGGGAAATGGGAAAAGAGAGAGTGGGGAAA

GGAAAATTTTTAGTTAAAAGAGAAAGAAAAAGAAGTGACTCTTCAAAAGATAGTTATTGA

TGGGCCAACTACATTTAGAACTATTTAGGGTTTGGAAAACATTTGGGACCATTTAGAGTT

GAAATAATTGAAGTGTTATGCTTACAAGTTTGAGATTAGGAATGGGAAAGTTGATTATAA

TAGAGAAAGTTCTAATTTTTTTAGAGTTAACAGGAGAAAAAAGAAATGAATAGATTTTTC

TACG

>LsbHLHR67

CATCATTATCTTTATATATAAAGGCTTGGAGTTTGGATATATAAGCAGAGAGAATATGAT

ACAAGAAGATCAAGGACAGTGTTCATCTCAAACAATGAACAACTTTGAAACCTATCAACA

AGAACAGTTTCTTCTCCATCAACAACAAATCATGCGGCAACAAAACAGTTTCTCTTTCCC

CGGTGGTGATCATCATCAAGTTGTTTCACCAATCTTGCAACAACAACCATGGTCATCCAT

GTCTATGCAACAATTCCATGTCCATGACCCTTTTGTTCTTCCTCAACAGCAAACTTCATC

ATCACCTTACGCAAGTTTGTTCAACAGATCTTCTAGGGTTCCGTCTCTTCAGTTTGCATA

TGATCATCATCATCACGGAGGGTCTGAGCAGCATCTTAGGATCATATCTGATACACTTCA

CCAACATGGATCCTTTGGTGGACTTCAATATCAAGGTGGCGATGTTGGAAAGATGAGTGC

TCAAGAAATCATGGAGGCTAAAGCTCTTGCAGCTTCCAAGAGTCATAGTGAAGCTGAGAG

AAGACGCAGAGAGAGAATCAATAATCATCTTGCTAAGCTTCGTAGCTTGTTACCAAGCAC

AACCAAAACGGATAAAGCATCATTGCTAGCGGAAGTGATACAGCATGTGAAAGAGTTGAA

GCGTCAAACATCATTAATATCAGAAACATGTCAAGTTCCAACGGAATGTGATGAGTTAAC

AGTAGATGCTGCAAATGATGATGATGAGGAGGAATATGGAAATGGAAATGGAAACAAGTT

TATTCTTAAAGCTTCACTTTGTTGTGATGATAGGTCAGATCTTTTGCCTGAATTAATCAA

GACATTGAAAGCATTAAGGTTAAGAACAGTTAAAGCTGATATAACAACACTTGGAGGACG

TGTGAAGAATGTTTTGTTCATTGCTGGTGAAGATCATGATCATGAATATTGCATTAGTTC

TATACAAGAAGCACTTAAAGCTGTGATGGAGAAGAGTGTTGGAGATGAATCTGCTTCTGG

AAATGTTAAGAGACA

>LsbHLHR68

ATTCTTGTACATCATAAATTATGCAGTATTCAATAAAACTACCTAGTTTAATTCATTAGA

TTCTAAAGACAATACAAATTCCTAGTTTCTTGTTATTCATCATCATTCTAATCACTGAGG

CAACAGTAGACAATCAAAATCCACTTGATATCAGAGTCGAGTTGAGAGTTTTATGTACAT

ATTATAAGCCAATGTGTACCAATGATGTATCATTAGACTATGTTTGGATTAACAACGAGT

TTGACAGAATCACAACAATCATCGCGATTTTGTCGAACTCGTTGTGAATTCAAACATACA

CTAAATATTATTATGAGGTATAATTTGGTTAAGTGCTCTCTTAACTTCCACAATGCTAAC

TTTCTTCCCATTACCATTTTCCTTAACCTTAGCCCTCAATTCCGCGACGAAAACGCCATT

GTTAAGCGAAGATTGAACTCCGATAACCTCAATTCTCAACTCTCTCAACATCATCATAAC

ATCCAACAACAACCCTTCTCTTTGTAAACATTCAATCTCCAACAAAGCATCACTTTCTAT

AATCGAAACCTGAACCGACGCAACAACCTCTTTCTCAACAACCTCAACTGCTTTCGCTCT

AACGCCCCCACCACCGCCATTCCCTTCCACAATCCTCACTTTTTTCTTCTCAGTCGGACC

CACCAAAACGGGTACTCCACTTTTCTCGGACTCCATCTGACGGTTACGTGTCTCAAGATC

TTGAATCTTTCTCCGAAGCTGTTTCAGATACTCAATCGTGTCGCCTAAGATAGAAGCTTT

ATCCATCTTCGTAACAAAAGGAACCAATGATCTTAGAATTATGAACCTCTCGTTGAGTTT

CTCTCTCCTCCGTCGCTCAGCGAGGACATGGTTCGCGCTGAGTTCGTCTTGGGGAGTTCC

CTTGCCGCGTAGCCTGGCGGAGGGATCGTTGCTGTTGACTCCGGCGGTGTCGTGTGTTTG

TGGGGAGGTTTCGTCGTGGTTTTTGGTGTGGAGGTATGGGACTGTGAAGAGGATGTATTT

GAGGAGCCATTGGGAGGTGGCGGTGTCCGGCGGCGGTGGCGGGTGGAAGTGGTGGTTGGT

CCAGGTGGTGAAAGAGGATTGGGTGGAGTAGTTGATGTAGTTGATGGAAGGTGAATCGAT

CCATTGGTTTTGGAGAACGGTGGTTACTGTTTGAGAGTAGTGTGTGTCTTCTTGTGTTAA

GTCTTCTAATGGATGATGAAGCACTGCATAGAAAGAAAGAACAAAGACATGTCAATTGTT

AAGTATTCTACTCAATTACTATTTCAAAATGATTTTTTTTC

>LsbHLHR69

AAAAAACAATGAACAAAAAATCACAAAAAAAGCACAACATTGTTTCATCATGTTACATAG

ATTCTGAACATTCTGACTCAGAATATTATCCACAATTACCAACACCAACAACAACAAATG

ATTCTTTTGAGAAAAGAGAACCGAAGAAGAGAGGAAGAAAACCACTAACCGGAATTCAAA

CTCCGATGAACCATGTAGAAGCAGAGAGACAGAGGAGAGAGAAACTAAACAACAGATTCT

ACGCTCTAAGAGCAGTGGTTCCAAACGTTTCAAGGATGGATAAAGCTTCATTGTTATCAG

ATGCAGTGGATTACATCAACGAGTTAAAAGCGAAAATCGAAGAACTAGAATCAGAGAATC

AAAAGGAATCAAAAAAACAGAAGATGGAAACAATCGAAAGTACCGTAACAACAACTTCAA

CTGTGGTGGACCAAAAAACAACATGTAGCAGTAACAACAACAACAACAATGTTAGTGCTT

TGGATATTGATGTGAAGATTATAGGTAATGATGCTATGGTGAGAGTTCAATCCGAGAATG

TTAATCACCCTGGAGCAAGATTAATGAGTGTGTTTAAGGATTTGGAGTTTCAGGTACATC

ATGCGAGTATATCATGTTTTAATGAAATTATGGTTCAAGATGTTGTGGTTGTTCAACTTC

CTGATGAGATGAGAAATGAAGAGAGTCTTAGATCTGCGATTCGAATGAGATTGGAACATG

AATGAATGAACGAACAAGAAGAAAATTATAATCCTAATAATGATTAGCTTTTTTTAATTA

ATCTGTTTTTGATTAATCTACAATCATGTATATGTAGTAACATTATTGTTTTGAGGAGTT

TAAGAAACTTGTCTCTTTGTGACTGTGCATATTGTTTTGGATTTCAATGTTGCAGACAAA

ACTAAATTAATACTGTTGTCCTATCATAGGTTCTGT

>LsbHLHR70

ATATTATATAGAAAAAGAAAAACCACCAAAGCAACCACATATGAAACCAGAGGGAGAAAA

ACCCAAAACCAAGTAGACAGAAGCTATAAGAAGGAGAATAATTCATCATGGATTCAAAGG

TTCAAAGAAGAGTTTCTCTCCAACACACGTTTCAACAACTTCGAGAAGTCACAAGATCTA

GTGCTATAAACAAAGCGTCTATTATTGTTGATGCCTCCAAGTACATACAAGAAATGAAGA

AAAAAGTTGAGGGACTGAACTCTGAGTTAGGGATTGTTGAGTCATCTTCCTCTCAAATGG

ATGAGCTACCTATGGTGAGTGTGGAAACCCTAGAAAAGGGTTTTCTAATTAATGTGCTTT

TAGAAAAGAATAAACCTGGTATGCTGGTGTCAATACTTGAAGCTTTTGAAGATCTGGGAC

TTGATGTGCTTGATGCTAGGGTTTCTTGTGAAGATAATTTCCAACTAGAAGCTGTGGGAG

GAGATAGTCACAAGGACGACAGTATCAACGCGCAAGTGGTGAAGCAAGCAGTGTTGCAAG

CAATCAAGAACACGGACGACTAATCAAAACAATGCTTACTAATAAGTCCAGTACACTTTT

TTGTCTTGTTTTATTAGCTTCAAATTTGCACATATATTTCCAATGTTATGCCAAAATCTG

AATGGCCCTATCATCAACTTCTTCTGGATAGGTTGGAAAATGAATGAATGGACATGCATT

TTTCAAGATGATATAAATATATTTATAGTAACATTTTGGCTTTCAGTGTGGGTGGGGGAG

AGAGTCGACAATAACTATTCTTTTCCAGATAATATTTGTAGGAGATTTGTTTGTTGTTAT

>LsbHLHR71

TTGTCATTTGTGTGACTTAAGTTATTCATGATGAGACGAGAGGAGCTAAAATGGATCTCT

CCTCCTAATCAATTTTCTTATATGCAAGAGTTCCATATCAAACTTCTAACCACAACAATG

GTTTCAATGGGCATAATCGACATTTCGACTGAAGCATGCACCAAAATCCACCGTACAGTT

CATGTCACTACCGCAACCAGTACATCTCCGCAGCACAACAACCCACGTTCTCAGCCTCGT

TGCATGTACGTGCCTGCCCTCCAACAGAAAAACAGAGCACGCCATTTCTCTCTCTCTTCT

CTTCATTTTCACTTCCAACTTTTTCATCACAAAATCACAACCTTTCACTAACTTAGTTTA

ATGTTAAAAAAAAATGGAAGGAGAAAGTATCAACAACAAAGACAACAGTTCAGAGCCAGT

GTCATATGAAAATGAAGAAGACACAAACGACCACGATGTCTCGGAGGATCATCATCTTCA

AAATCAACAATTTGGAAAAATTGAGGCCCAGTGGAATTGTTTATCAAAACTACCTTATAG

TAACTATGTTGAATATCTCTCTGAATCTTCAACTTATCTTCCAACAGCTGAGTATAATTT

GGTTGGGAACAATTATCAAACACTTGGTGGCACAAACAGCTTCAATGATCAAGGAAGAGC

TTTTGGTTTTAAACCGATTGGTTCTTCAAGTAATGACTATGGTAGTAGGAAGCATGTTGG

GTTTTGGAGAGACAATGGAGAAGAAGAAGAAGCAATTACAGCTAAAACTGAAACTACTCA

AAACCTTCTCAATGCTGAGGGTGATGCAACATGGCCAAGTGATTCAGTTGGAGATAAGCA

CAATGCTTCAAGATTTGATCCAATGGGAGTAGTTGGTGATGTTGGGCCTTTTTTACTTCC

GAATCCGAAATCAGGAAGCAGCTCCACAAAACATAAAAGTGAGAAAGCACGTTGCACTGA

CCGTCAACGCAGGCAACGTATCGCTGATAACCTCAAAGCCTTACATGAATTGCTTCCAAA

TCCAGAAGGGGGTAGTCAAGCTCAAGCTTACATATTGGACGACATCATCGACTATGTTAA

ATACTTGCAGAATCAACTCAAGGAACTAAGTGGAAGTAAATTGCAAAGTGACTCAAATGC

TATACCATTAGTTTTTCATGAGGGTTATGGGCATTATATAAAGGATCAAATGCTGAATGA

GCCACTTGAAGAGATAATGGGGAAACTAGTTGAAGAACATTCAGCAGCAACAAGTCAGCT

ACTTGAAAGCAAAGGTCTTATTCTACTGCCTATTGCTTTGGTTGAAGAATTGAACCAAGA

TAGCTAGTTAACTATTTAGGGTGATATCTGTTTACTGTGTTTGGATCATTATGGTGTGAG

AATCAAATTAACTTAGTTAGGCCGTATGTTGTTTTTCTAACTAAAACTCACCTCTTGAAA

CTCTTTTGTACTAGGAGGTAATGTGCAATGCTGTTTCTGT

>LsbHLHR72

AGACAATTCACGTACTAATTTGTGTAAGAAACATGATATTTGAAAATCACATGACATTGA

TCATAACCCTATTCTTTCCAAGGCCCTAGAAACAAAAAGCAAGAAAACCAAACAAATCCT

ATTACAAAACAAATATAACTGGAAGATACATAATCATTACTAGAAAATGAAAACGAGGTC

ATCATCATCATCACACACTGCATTCATCGTGTTAACCTAAGGCATATTCATTCTTCTGTC

CATCCCACGACGCCGTTTCGACCTATCACTACAACTACTCGACCTATCCAACAATGACCT

AAGCGAGTTCTGAAGAAAATGTATTGATTCAATGCTGCTATCTTCTTTATCACCAGCAAC

AATAAGAACGTTCCGAGTTCTTCCTCCCAGTGTGCAAATTTCAGCTTTCACTGTCTTCAG

CCGCAGAGATTTCAGGATTTCTATTAGCTCCGGGATTAGGTCGGACCGATCCTCGCAGCA

TAACGACGCTTTGAATATAAGTCTTCCGTCTCCGGTGAAGTCTCCGCCCCCGGCGGAGAT

TACTGTTATTTCGTCCGTTTCTGAGGGAACTGTTTCCAGTTCTGTGATTTCAGATGTTTG

CTGCTTTAACTCTTTCACTCGTTCCACAACTTTTGCAAGAAGCGAAGCTTTGTCTGTCTT

TGAATTGCAAGGGAGAAGAGTACGAAGATGATCAAGGTGAGAATTGATTCTCTCTCTTCT

TCTCTTCTCAGCCTCCTTATGATTCTTCAAAGCAGCAAGAGCTCTATCTTGTTGGGAGGA

GGGTGTATCAGTAATTTCAGAAACTTCAAAAGGGTGATAATAGTTGTTGTTATCAGAAGA

GCTACATAAACTTGGTTGCATCATAGGAGTACTTGAATAGTCAAAGAATGAAGGGTTGTT

AGCAACAATGAATTGAAAGAGTTCAGAGTTGTTTTCCATTGCAATGTTTTGATGAAGCAT

ATTGTGAAAAGAAGAAAGAGAGGGGGTGTAGATAGCTATAAGAATAGAAAGCTAGTAATA

AGAA

>LsbHLHR73

TTACCCCAAACCCAATCTCCTTTGCCAATAATTTGGGAAGTCTTTGAGCTGACCAAACAA

ATATGTTTCCTTTACAACGATGCAATGAACTGTCGTTCCCCTTATCTAATAGTCTCAACC

ACCACCCTCAAAATCACAATATCTCCCAAGATCTGATTCGTGAAGATTGTGATTCTCTTG

TCCTTGATTTTTCTCATAAAAAATTGTGTAGCAGCCGGCCACCAAAGAACTTATTTTATA

CACCAGGAGTTGATACTGCATATCATTGTAGTAATCACAATTCAATCAAAGATAATAATA

AGAAAATGATTCATAAGGAGATTGAGAAGCAAAGGAGGCAAGAAATGACCACACTTCATG

CTTCTCTTAGATCCCTTCTCCCTCTTGAATTCATCAAGGGAAAACGTTCAATATCTGATC

AAATGAATGAGGGAGTGAATTACATAAATCACTTGAAGAAGAATATAAAAGAACTAAGTG

CTAAAAGGGATGAACTCAAAAGCCATTCTTCATGTAGGTTCAGTATCCACAAGAATAATA

CAACTGTGGGAGTTGAAATTAGTACTAGAGAAGAAGGGGTTCCACTATCTAAGTTGTTGG

AACAACTTCTGAAAGAGGGACTTGATGTTGTTAGCTGCTTTTCAATCCAAGTCAATGGCA

GATTACTCCACAGTGTGCAGTGTGAGGTTATAGATTCCAAGAGTGTAGATCTATCTGAGC

TAAGAAAGAAAATTTCCAAAATTAATCCATCATTTAGTTGTTCTGATTAAATAACTCTCA

TAGTTGCATAAAGGCTTTCGATATTTCGGTTGACACAAGTATTGCATACTGAACGTGGTT

GTTGCAGTGTGAATTATCAACAATTTCTTTTTCATATAAAAATAGTAAATAACTGTATCG

ATTATTCTTGGATGTGATTACATACAATTTGCATGGCTATTTACAGGAATTGATAAATTC

ATATTCTTGCCCTTTTTAAGTGGCTGGTAATATATTATATGTAGTGTGAGTTTGCTTGTG

TGTAATGTAAATGTTGAATCACTTTGATCTCTCTTTGGCTTGCAGTCAGATATATGTGGG

TAATTAGAAATGATATATTTTTGATGGGTGGTAATAAAGGAAACATTGAAATGGTGGATT

TGGCGAAATTTTTATGTGAGATTGGTTCAGAGCATGTTCGAAAGGTCTTGCGGTGTGCAA

TAAAAGCATTTGGTTCATGAACCCCTTGTTTCGAAAGGTCTTGTA

>LsbHLHR74

ATATTGATAAAAATACAGTAGTAGTACACAGTTAGTTAGTTACACACTCTCTCTACCTTC

CTCATATGGACATGGATTCCACTGGTGGTGGTTCCTCCTGTTGGCTCTATGATTATGGCT

ATGATATCTCTCTTGCTGCTGCTGATTTCATGGTTTCTTCTAATCATCCCTCTTCTGCTG

CTGCTGATTTCATGGCTCCTTCTGATCATTACTCTTCTGCTGCTGCTTTCAACTGGATGC

CTCACTCTCAGTCTCAGACTCACATCATCAACCCTCCTTCCTCCAATATCAGCTTGGAAA

TGGAATACTCACTAGATTCGACCACTGTATTCGAAAGTGGCCCTTCAAATCCTTTAAAGC

GCTTGGAAATGGAATATTCATTTGATTCAACTTTACTCGAAAATGGCCCTTCAAAGCGGT

TAAGGACAGAATCATATGCTTCTGGCTCCAAAGCAGGTCGTGAGAAATTGAGAAGGGATA

AACTGAATGATAAGTTTCTGGAATTGAGTTCTGTCTTAGAGCCTGATACACTGCCCAAAA

CAGATAAGGTTACTCTATTAAATGATGCGGTTCGAGTGGTGACTCAATTAAGAAACGAAA

CACAGAGGCTCAAGGAAAGGAACGATGAATTACGCGAAAAAGTTAAAGAGCTTAAGGCTG

AGAAGAACGAGCTTCGTGACGAGAAGAATAAGCTGAAGCTAGACAAAGAAAAGCTGGAAC

AACAAGTGAAATTAACAAGTGTGCAGTCAAGCTTCGTCTCTAATGCCATGGCTGCTAAAG

CGCAAACTGCTGGCCACAAGCTGATGCCTTTCATTGGTTATCCTGGAATTTCAATGTGGC

AGTTTATGTCACCTGCTACCATTGATACATCACAGGATCATCTCCTTCGACCACCCGTTG

CTTAAAGTGGTTAGCAACAGCATCCACTCTCTTATTGTACAAAAATGTTATTCGAACATC

AACTTGATACACGGAGTTCTTGAATTTATGCGTATCAAATTGTTGTTGGAATAACATTAC

TGTCATCGAATGCGTCCTTTGCTTGTATTATGTTTTTGTCCAAGTTATGCACTTTGTTTC

TGTTTGTTTTTTAAATTCAGACTAAAAGTTTGCTTGGATTTGGTTGATATCATAAATTTG

TGC

>LsbHLHR75

TGTCTCTTTTTCTTTTCTTTTTTTCTTTTTTTTTTTACCTATACAAGCATCCTCTATATG

CTGGTGTCCAGTAATCTGGTCCAGTGTTCTCTCTATAAACTACTGGTGTACATGAAGTTG

GAACTAAACAAAGACCTCTGCTTCGAAGATCCATTTTCGCATCGTCCTTATCTTTTCTAT

CGAAGTACATTCCCCTTGGATCCTTGTGTGAATTTGCCTTCAAGTAAGGGTTACTCAGTA

ACTGGACTTGTTCTTCAAGATACTTTATGTACCCTATTGCTTCAAACAGCACTGAAGCTG

TATCTGTCTTTCCAAATGGAGACACAATTTGTTGAAGAGCTGTGATCTTTTCTCCTAGCT

TGACTTTAGGTGCTTGCACCTTTGAAGAAGATGAATTAGTTGTCGATGTATCTTGTTTTG

GCTTCTTCAAGTTTGCTTCTAATGATTCTTCAGATCTTTTCTTTTTTACTTCACGCGTTG

TCCCCTCTCTTCCACTATTAGTCCTCATATGTGATGATGTATGAAGTCCTTGCTTTCTTG

ACTCAGATAAGTTTGAGTATTTCATGCTAGGTCTTTGTGCATGGATTCCAATAACTGGCC

TTCCAAACCTACTATTAAAGGATATAACATCTGAGAAACTTTTGGTGCATGGTGAAATAT

TTGGCAACCCTTGATAAAATTTTCCATTTTCTCCAACAGAATTAAGACTACTATTAAATC

CATCAAGATATCCATTTGGATTCATAAATGATTTTCCAAATACTTCATGATTTCCATGAT

AATGATGCTGATGATGATGTTCTTGTTTAACCTTATTCATGTCTTGAATATCATTGACAT

TAGAGAACATTTTATTACATACTCCTCCAATAGAATCTCCAAAAGGTAATTGTTTGAGAT

GACAATTAGGGTCAGATTCATAGTGATGATTATCCATATTGGAGGAATTGGAATTCAAAT

TATTTGATAAGTTGTTTGTTTGTGGATCAAAGTGGCTACTAACTTCAGGGTCAGGTGGGG

CAATAGACCAAGTGCTAACAAGATTGGATAATTTTGTCAACCTTTCATTATTCTCAATAA

GAGCTTCACTGTACCCATTTAAGTGCTTTTCAAAACTTGTATTGAATGAAGTTGAACCAC

TATATTCCCAACTTGTTGTATCAAGTTTCTTCAAATAGTCACAAGCAGGTTCATCAAACA

TGGTTT

>LsbHLHR76

AACAAAGATAATAATTAAACATTACTGTAACAATTAGTAGAATCCTTTTGCATCTCTGGC

TAAAAAACTAATGAAGATCAGATACATTAATCATTGTCTTTGCTAATAATAATGAAATGA

TTAGAGATGATTATGATAATTAAAATCAACCCTGTGAATGATGATTGTAACTACTAGGAA

CCATTTGCATGGAAAAAGAAGGATCAAAAGAGAAAGCAATACTATTTGGATTATTACAAT

CCATAGCATTAACTTTGTTACCTAAACTTTCAAGTGCCTTAATTTGTGACCTTAAAAACT

TGAGATAATTTGCAGCTTCTTCAAGCATTGAAGCAGTGTCCATTTTGTTTCCACCAGGTA

CTATCTTTTGCAAAACCCTAATCTTATCACTTATTTTTTCCCTTCTTTGCCTTGCAGCCA

CAGTTTGTGGATCATTTGATATCCTAACATTCTTTCTCTTAGGCTTTTTTTCAATCACAT

CGATGTCGTCGATTCCGAAACTCACTGGCCTAAATGCTGCAGCTCTATACATCATTTCCT

TCATTTGCGCTATTGCTTCTCGATCAGGCTCTTCGGTTGAAGATGAACAGCATATTGATG

ATGATGATGAATTTGGTTGTTGGAAATTTATGTTGGTTGAACAAGGTCTTTTGTCAGATC

TCGGTTTCTTGCTTTTTGGTGGTTTATCAGTAATAAGGCTAAAACCACCTTCAAGAGATG

AAGAATTATGAATAGTACTTGAGTTGATTGCTTTTCTTTGAGTGATGTATTGATCTGATG

AGCTTTGAGACACTGTTTTGTCAAGTTCAACATTGAATGGACATCCCACGGCTTTCTTAC

CAACGCGTCTGATCAAAGACTTATCTGGTGTCTGTATCTGTGTTTTATAACTTATACCAT

TTGAACTAACACTTCTGATTGAACGTGTGACTGGTGCACGTGTTTGTCCCAATGCTTCGT

AACTTGTACCATTTAAACCAACACTTCTGGTTGAAGGTTTGTCTGACACCCGTGTTTGTG

TCAGCGTTTCATAGTTTGAAAATTTTGAAACATTACTATCGGTTTCACCGGATGACGATG

TAGGACTGACATAATTGAAGCTCCATAAATTTCTGCAGTCAGAAAATAACTTTGAAATTC

CACCATCATGATCATTTTCAACAGAAGTGTCTGTGTTGCTATTAGTATCTGAAAGCAAAC

AATCAAAAGAGTTGTTAAATTTGAGTTGTTGTTGTTGTTGGTGGTGG

>LsbHLHR77

AATCTCCATACTTTCCATAATTTCCATACTAAATTAAGCCAACTCCTTCTAGTCCTCTCT

CATTCTTTTAAATCCTAATATTGTTCACATATTCATATTTAGTGTTTGTCACTACTTTTC

AATCTCCATCTTCACAGCAACAAAAGTCCTTTTTTCATGATCCATCAACTTCCTTCTTAA

GTCGTTTGTCCTTCTTGGATGGTATCTACAATGTGATAAGCAGCCACCAACCAAGTTCTT

CATTGGGAAGTCTCGCACAACAGCTTTTTGATCAGTACACGATTTTATCATTCGATATCA

ATGATGACCGGATTCCTGGACTAGCTTTCAGGAACCAACGACGTTATCTAGAGCTGCAAC

AGGTGGAACTTCTGGCACTGTCTTCAACAGAAATACAGAAACAATTCTACAAGGAAGCAA

GAATTAAGACTTCTGTTTTCATGGGATGCAACAAAGGAGAAATTGAGCTTGGTTTCTTAA

ATATGTCCCATACTGATATTCAAACAGCACTTAGGAGTTTATTTCCTGAAGATTTTTCAA

GACAAATTCAACAAATTGATCAGAATAATAACAATACTAATCCACCTTCATCATCTTCAT

CTTCAATGAGATCATTATCAACAGCAGGTAGTCCTGAATATTCATCTCTCATTTTCAACA

CAAATCCAGCCGGAACTTCTCCATCATCACATCACTTTCCTGATCATATCCTTGGAGGAG

TAAATATTCCTCCAATGAGACCTGTTTCGAACACACTACCCTTTCATCTTCAACAACTAC

CTCAAATCACACCAACACAGTTATTCCCTATTGATCACAATGATGCTATAATGAGAGCAA

TCCAGAATGTTCTATCTACACCCCCTTCTCAGCAAAGTTACGCGGCGCATCCCGGAGCTA

GTGCATTTGGAAGGTATAGAAACGATAAAAGTCCTATTATCGTAGGGTCGAATTTTAGAC

GACAGAGTTTGATGAAGAGATCGTTTGCATTCTTTAGAAGCTTGAATTTGATGAGACTGA

GAGAACGAAATCAAGCCATGCGTCCTTCTAGTAACCAACTTCATCATATGATATCGGAGC

GGCGAAGGCGCGAAAAGCTCAACGATAACTTCCAAGCACTTAGGGCATTACTTCCTCAAG

GAACTAAGAAAGACAAAGCCTCCATACTGATAACAGCAAAAGAGACCTTAAGATCATTGA

TGGAAGAAATAGAGAAACTAAGCAAAAGAAACCAAGAGTTGATGTCAGAAAAGTTAACTG

CTTCGAATAAAGAAACCATGAAATTCTCATCGAATGAGAGAATTAACGTGCGAGTTTTGC

ATGTATCGGAATCAAGTTCATCCGACGATGAACCAATGGTAGTGGATTTACAAGTGAACG

TGATCGGACAAGTTTCTCAAGTTGATATGTTGATTCGATTATTAGAATTCTTAAAGCAAG

TTCATCATGTCAATTTGATTTCAATGGATGCAACAAATTCAAATTCAAATATTCCACAAC

TTCATCAAATAACATTCAGGCTAAGGATTACTCAGGTGAGTGAATGGGACGAAGAGGCTT

TCCAAGAAGCAGTGAGAAGAGTAGTTGCTGACTTGATACAGTACCAAGTGGACCAAAATC

TATGACTCACTTGTTATTATCTTAAATTGATTATTGTGCGGCGGTATCAAATTGTTTTGA

TTATGTATAAGCCAATTAAATTAGCTTCACTTATTTTGTTTGTTTCATGATTAATTTGTT

TAGTATAGGGGGTTTATACTATATGT

>LsbHLHR78

TAAACAATAGGTAGCAAAAAGTACGATATAATTAATGATACAAGATGTTTAATTTAATGG

ATCAAACTTGGCCATCATTGATTCTTACGAAGGAAGATCAAGTTTGCTCCATTAAACATG

GATACACTTCGATTTGTTTTTAAACTCATAACTTCCATTTGTAGGATTATTTGATATTAT

TCTATCAATAATCAAGTTGGTCCCCCAAAGTTCGGGCGTTGTTGCCACACCTCACTGCTG

CCATCACCTGCTATGTATGACATGCATGACAATGGCACCAGACAGAGGCCTCGACTTCTC

AGATATTGCTTTGGTTCTCCATTTGTGTCACCTATGGCCAAATCCCCTTGCATCAATCTG

TTGTTTTGGTTTTGCGATGACTTCATATAGGGAACACTCAGTGTCTCTACTTGGCCTTGA

AGGAATTTAATGTATCCGATAGCTTCCATGAGTACAGATGCAGTGTCAGTCTTTCCAAAA

GGAGCCACTAACTGCTGAAGAGCCGCAATTCTATCTCCTAATTTTTCTTTCCTAACCTTA

AAGGGTGCACACGAAGGCGGTCGTGATTCTGATGAAGATCGTGATTTCTTCAACGCGGTT

TCAGATTGAGAAGCCTTCGATTGCATCACTCTCTTTGTTTCTCCGCCACCATTGCCGAGT

AGCGACGGCTTATTAATGGAGTTACTAGAAGAGGATCTGTTTGCGAGTTGGTTCTGCATA

GGATGAAGATGAAACGAAAGGTTTTCATGATCATTAGTAGTGAATCTACCAAGATGATGA

TCTTGTGAACTACTTAATAGATCTAATGACTGCATGTTCATATCCAAGGTTGAAGTCGTA

GACGAGGACGAGTAATGATTCAAGTTCGAGATATTTATACTAGGGTGAATCTGAGTCTGA

TTACCAATGTTAGGATAATTTTGATTACTATAAAAGTCTCCACCAGTGTAAAGATTCTTG

AGCATGAGAGCATTCATATCCTTGTGTTCACCATTAGTACTATTCTTCATCTGTATTGTA

GTGGAAGAATCTTCAAGTATTCCATAGTTCTCCATGTTTGAATTAGGAGGAGTGTAATTA

AGCATTTCAGTAAATTTAGGGAAATTCGCAAACGAAATCTCTTCCTTAACATCATCATTT

GTCCATTGATGGTGATGATAACCTAAATCTTGAATCGTAGTTCTCGATCTCGAATATCTC

GGATCAACTTCTTGGAGATTTGAGTTGAAGTTACCTGCAGCATTATTCAAAGTGATGTTT

GTTGGGGTCCAAGAGTGAATGGTGGTGGTTCCTCCAACAGCATAAGAACATGGGATAGAA

GAAGATAAAGAAGAAACAGGAAGAGGAGGTTGTTGGTCTTGGAGATGATGGAAATTTGTA

GACTCCATAATCATAAATTTATAATTATACTTCTTTTATTTAACTTAATAAAGG

>LsbHLHR79

TTTCAAGATTAAAAAAAAGTTAAAGTTTGGATCTTAATTCAATATTGACTCTTGTTGAAT

CCTTTCCAACATTTTGTATACAGCTGAAGCTATCTCATCCACTGATCCAAACTTGCAATC

CTCTTCCACCTTAACACTGATAGAATAGAAGACAAATTCATGAGATGTAGTGACATTAAG

ATGCAAGATTGTGAGACACATTCCATGCAAACTAGAAACCATTTTCAAGAGTTGTTTTGG

TTTTTTCTTTGATCTTATTTTCAGATTTGCATGGCTTTCCACCATTGTCACTTCAATGTC

AGCAATAGTATTGCTAGATTGATGAACTTCATCACCCATTTTTGTCTCACAGACACTTGT

TGAGTATTGTGGAAAGGTGAAAAATTCAGAAAAGGGTTTGTTTTTATTTCCATCAATAGT

TTCAAATTCACCCTCTTTTTCTTTCTCAACACCAAGAAATTGTAGCTTCTGTTCAAGATT

CTTCACAAAGTTAATAGCTCCTCCAATTATTGATGCTTGATCCACCCTTTGGACGTAAGA

TTGAGGCATGAGAGACCGAAGAACAGCGAGATACTGATTCATTTGTTTTCTCCTGTTACG

TTCTACAGCAATGTGAGTCATTCTCTGGTTCTCGATTTCTTCCTTGTTCTTACTTGTTTT

GACTCTGCGTTTCTTGGAACGAGAACGAGTGGGAATTGAGGTATCTAGAGATGGATTTGA

CACCACATTAAGTACTTGATCCTCCATTAACGGTGAAGAATTGTTGTTACTCCAATTCTC

TGTATGATTGTTGAGAAAAGCATGCTGTTGTTCATTATGAAGGTTGAAGTTACTGTAGTC

ATAGTTAGGTTCAGAATCTGTTAATAGGTTGTAGTTGAAGAGTTCTTTGAATCCGTAGGT

AAAAGGGTCTTGTTGTTGCGGTGGTGGTGGTGGATATAGTAGAGTTGGTTCTAGTGCCAT

GGCTTTTTCTCTCTCTTCCTGGGAGAAACATATGAAGAAGATGATGATGAAAGAAAAAAG

>LsbHLHR80

TGGACCAAACCTTTTCTAATACTTCTCTTTTCTCTCTTCATAACACTCCCAAAATGCTTT

GTTTTGGTGGTAACTACCAAAATGAAATTGAAATAATCACACCTACTCAGAAATCTGTTG

TCACTTCTAGTGACTCTTCTTCTGCTTCTTCATGCAACCACACCAACACTGTTTTCAATA

ATAAAAAGCGGAATGGGTCAGAAGGACAACAACTGGGGACCAGGGCAGGTGTTGGAGGGC

AGAAAGGTCCTAAGAAGACCAAAGGAGACAACCACCCAACTTCAACCGGACATGCAAAGA

AAAAAGAGAAACTTGGTGAAAGAATTGCAGCATTACAACAACTTGTTTCTCCCTTTGGCA

AGACTGATACAGCTTCGGTGCTTCACGAGGCAACTGGTTACATAAGATTCTTACATGATC

AAGTTCAGGTTCTGTGCTCTCCATACCTACAACCTTCACAAATTCAGTTTCAAAATCACC

CTGGGGACGGAGATAACAATGGAAGAGAAGAAGAAGAAGAAAACACAAAAGTGAACAAAG

ATTTAACAAGCCGAGGTTTATGTTTGATCCCTGTTGGAAGCACCTTACACGTGGCAGGCA

GTAACGGTGCTGATTTTTGGTCACCAGCAACAACAACAGACAACAATGTTGTTTCACCTT

CAACCACTATTCCTAAGCAGTAGGAGTAATAAAAATTAATTAATGGAATTGAAAAAAAAA

GTTACATCTTTTGAGAAATGGGTTTCTATGTCTGCTCAAAATGGAGATGTACTCAATATG

ATGGTGACAGGTTATTTGGAGAAGGAAAAGAAGAATGAGAAGGTAAACTTTTGGAAAAGG

GAAAGAGGGATTAACAATTAACAAGATTAGATTAAAGTTTACACATGGGGTACAAATGAA

ATGTGTCATGATTTTGTCCTTAATTATGTAATATCTGTTTTTGGGGGGTCACTTTGTTTT

TCTATAGAAAGAAGCAAATATTTTGATGTGTGTGGC

>LsbHLHR81

AACACCACAAAATGCTTAAGTGATACCTATTTCTATGAGAAAGTAAAATTAATAAAGTTT

TGGAAGTATTCCAAACTTCAAAAATCGATTTTTTTTTCCAGTCTTCTAATTACTGATACC

ATTTAGCTACTTAACTGCGATGTGAATATAGCATTTGGCCCGCTACTTCTCATGATCGCA

CCTGACTGGTCTCTTCCGGCGATACACGTTTGCAAAGAGTTGTTAGTAGAAGGTGCAACC

ATCATGCCTGACTCAAATTCTTCGACACCTTGAGAACCACCTAATAGATTGGTAGCAGAA

TAGATTGCATTTGATTGCTGGGTCTGGTTCGGCAACATGTCTTCACCGTGTACCGCCTCT

GAAGTTTGTTGAAGAGGTCTTTTATGTGGCGGGATACGGGATTCTACCGCGAAAGAGGTT

TCTGCATTGTTAACATACGAAGGATAGGAGGACATCGACGGCTGACTCGTCAATGTGTTT

TTGTTAGGAAGATTGGATGCATAGAGCAAAGGGCTGTTGTCTTGGGGATGAGGTAAAGTA

GATGTCATGTTTAAAGGGGTAGACCGGTTTCCGTCACTCAATTCCATACTCAACCGAGAC

AACGACAGAGGCTGCAAGCCTTCGGGATAGCACATAGGGTGCAAACTTAATCCATTTTTC

AATGACAGCATCTGTACTTGAAGCTGAAGCTGTTTAAGGTATTCAATAGCTTCATCTAGC

ATTGAAGCCTTATCAGTCTTGTTAGAATTAGGTATCAGATTTTGTAAAGCCTTCATTTTC

TCATTGATCCTACTCCTCCTTCTCTTTTCAGACAAATTATGAACTTCTGCTGCTCTACTT

CTCTTTGAAGAACTCCTAGAAGGTACAGATTTTGTTGGAGCTTCTTCTGTCAACACTTCA

ACACCCTCCTCACTTTCACAGTCATATTCATCAGTGTCGTTACCACTGAGTCCTCCTCCG

ACAGAAGAGGAAGGGTTTGGTCTGTTAGTAGGATGAAGGGGTGAGATGTTTGCATGTTGA

AGTGGGTTTGATAGGATGGATCCAGGCATGAGATGGGAAGAGGAAGACGAAGAGGAAGAA

GAACGGAGG

>LsbHLHR82

GGTCATCTTCCTGACTGAGAGTAAAAGCTTCTTTGTCATTTGTCGATGAAACACCACCAA

TTGTTGCTTCACACTGGATAGACATTGGATTTGCCGAGTTACAGTCATTGATGTTTTGCA

TAACTTGTGAGTTCACATTGGGCATTGGAAAAGAAAACATTGAAGCGCCGGGCATTGGCA

TGTGGAGTCCATGGCCAGGAAGTCCGAGCATTTGAGGATTTGGTCTTGCCCCTAACCACA

TTTGCATGGCAACACCCATGGGAGAAAAGGGAGACATATGCATCTGCTGCATTCCCGCCG

GTAACATCATCGGCATGTATAAACCACCGCCTCTCATTGACATAATTCGCAGTTGAAGTT

GAAGTGTTTTAAGATACTCTATTGCGTCATCCAGCATTGAAGCTTTATCCACCTTATTGC

AATTCGGTATGAGTTCTTGCAATGTACGCATCTTCTCATTGATCGTGTCTCTTCGTTTCT

TTTGAGATAGATTGTGCACTTCCGCCGAACGGTTTCTCTTCGAGCCAAAACCTCCTCGTC

CATGAGCCGTCCTTTTAATACCAACCGATTCAACCTCAACGTCCTCTCCATTTAGGATGA

ATTCCCAAAGAGCCTTCCCCGGAAAAATTGTAAATGAGCTCAAAAGTACCAGATTTCCAA

ATCGTGATAAGCCCTCCTGATCTCCCCTCCATCCACTTCGCACTCCACTTTGCACCAAAA

TCATCCCACAAAGAATCCATGTCGAACTTATCAAGATTGTGAAGTTTTGTCTCCTGGATG

AAACAAATATTCGTCTTAACTTTACGAAAGAAACACCTGCCTTAACTTTAAGCGCCATTA

TATATAAACACTCCTTAATTTCAATACAT

>LsbHLHR83

CTCTAACATTTTGTGATCTTTTTTATTTCATTTGGTACAACTAACATTCCTTCATTCATT

AGAAATTACAATCACAAACATGGGAAAACCATGCTCCAATTAAGCAAGTTAACATCTTAA

TACTACTAGCTAAGCAATTGTTTAGAAGTGGACCCCACCATTTGAAAAGGTTGACAACCC

TTCATCATCAAAGCAGAAGAATAATTCATCACATTGAAATTGTTCACAACACCACCAGCA

CCACCAATGTGTGATGATCTATTACTAGCTTGTTCCAAACTCTGAACCTGTTTCTTCAAA

AACTTCACATAATGTATAGCTTCATCTAGCATAGAAGCAGTGTCCATTTTAGTTCCACCA

GGTACTAATCTCTGCAATATCCTTATCCTTTCGCTTATCCTTTCCCTTCTATGCCTCGCC

GCTACGCTCTGCGGATCCTTCGATATCTTCACGTTCCTTCTCTTCGGCGGCCTAATCGAC

TCCGGATCGATATGCACCGGTTGCATCACTGCTATTCTAAATATCATTTCCCTCATGGCC

GCCATGGAATTCTTCTTATCAGAAGAATTAGGGTTATAAAGAGATGATTGTTGTTGTTGG

ATAGCGTTGTTGAAATTGACATTGGAGTGTGGAAATGGATCAGTCAAAGTTTGTGGAGAT

AAGTTAAATAATGGAACGGTATTGGTAGTGGTGGAAGTTCCATTGATAATTTCTTGGTAT

GTGGAAGGTTCAGAAAAATTATCAGGGAACTTTTCCATTTGCATCATCATT

>LsbHLHR84

CTTCTTCTTCTAACAACTCTGTTTCTTTTTCCTTTTCCTCCATTGATGAGAGCTTCCACT

ACTTTTCCCTTCAATAACCAGTTCATCACTTTCTCTATCAATCTTTGAGCTAGTAGTGGT

GTCTCTGACTTTGATCAAGTTTTTCTTATTTTATTTTTCATGCAAAAGTGTTGGGAATGA

AATTGTGAACACAGACACTGCATAAAGTTACAAACTTTCAAGCTTTTGGAACATACCCAC

AAAGGGTTTTTGCTTCAAAGTTTTAGATGATAGCTAGCTCCTTCTGCAATGCTGATTCTA

TGTTTTTAAGTGAAGAGGGGATAGATGTTAGAAAGATGATGGAACATAAAAGGAGCATTT

GCTCTGTTGATCAAAGCAATTATAATTCAATTGCGTCTAAAAAGCAAAAGCCTGATTTAT

CGATCACCACTAAGGATAGAAAGGAAAAGATTGGCGAACGAATTGTTGCTCTTCAACAGC

TTGTTTCACCTTATGGAAAGACAGATACGTCATCTGTTCTAAAGGAGGCTATGGAATACA

TAGGGTTCCTTCATAAACAAGTTAAGCTGCTTAGTGCTCCTTACCTCGAAACTACACCAG

CAATACAGATGCAGGATACAGAGTCATGCAGCTTGAGAAGTAGAGGGTTATGTCTTGTTC

CTGTCTCATTTACTATTGGAGTTGCCGAGGGAAATGGTGCAGACATTTGGGCTCCGATCA

AGACCACATCGCCCGGTTCTGAGAAAGATGTTTCTCAAATTCAGTGATTAATTTTGTCTG

TCAGCTGAGCAAACTTAAGGTTGACATTGACAAGTTGCTAAACAAGTTTATTAGCATTAA

AATTTATTGTCTAATTAAAATTTATTGTCTAATAGATGGAATCATAGTACCTGAAATAGT

CATAAGCTTTAAGAACTCTTCTATGATGAAGTTATCCCATTCCTTAATAATTTTAAAC

>LsbHLHR86

ATTTTGATGCACTAGAACAGATCACTAATGACTCTCACATACCTCCAACAGATATTTTTT

CAGATTTTTCACAAGCTCATCCATCCTCATGCTCTCTTCTTCATCCATCTGAGCAAAAAT

GTTAATGACAAGAACTGAAGCACCAAATGGCAATATGCTACTAGAGGTTATTGTGAGATT

GAAGTTTTCAAGCATTTCATGTATTTTGAGCACTAAGTCCTTTTTCTTCTCACAGTGAAT

CCTAATGAGCACTTCCTTTTTCATGACTCTAGCTTCAACCTCAAGCACTGATTCTGAGTA

CTTCTTTTTATTTGATGAAGATTGAGATTTCCACTTGTATATTTCTGGATTTACTGTATC

ATCTTTGTTTTGATTATTCTCCAATTCTTTTATTCTTTTTTGAAGCTGTTTTGTGTAACT

CACAGCTTCACTGAGTACATAGCACTTGTCCATCTTTTTCAAGCCAGGTATCATAGCTGA

AAGTGTGATAATACTCTCAGTCAATTCTCTTCTTCTTTTCCTCTCAGCCATTAAATGATC

TTGCACTGTCTCACAAGACCTTCTAAGCCTCTTTGTTGCTTCCTTCACCTTTCTTTCATC

TTTCTTCAAACTCCTTTTATTTGACATTGGTTCAGTAATCGGTTCAACACTTGAATTATC

GAAAGATAAAAGATACGTAGCTGGAGATCCAACACACGATCTTTCCAAAGAAATTATCGA

GTTTGAACTAAAACTTTTGTGGATTGCTTCAATATTCTCGTGATCAAAGGACAAAATACT

ATTTGTAGGGCTAGTGACACCACCTTCAGTATTAATATTTCCTGCGGAATTTGAACTGTT

GTTATGGATTTCATTGGAGCAAAGATAAGAAAAGGGTTCACATTCTGGGTCCTCAAGGAT

GATATCTTTGAGAAACTCTTGCTCATGAAATGAGTTGATACTTTGATTCATCTCTTCATC

TTCC

>LsbHLHR87

ATCAAGCATAAGATCTGAACATTGCTTCTAATTACTTGCACAACTGCACTTTGCCTTAGT

GTCTGATAACATCTTAAGTTGTTTCCTAAGGTCTTTAATGTAATCAACTGCTACATCCAA

CATATCTGCTGTGCTACTTTGCTTATTTGAGTTTGGGAAAAGGTCTTGCAATTTCTTGAT

TCTTGCACTAATTCTTATTCTTCTTTCCCTCTCTGCAATGCTTCTTGGGTGAGTGGCAAA

GCCTCTTTTGGCACGAATTTTAAATGGAACAGATCCTTGAATGTGAAAATACTTGTCCAT

ACTAGTCATCTTGGCAGAAGAGCTTGGCAGACTCAAATGGTGGCTCAAACCAAGTTTTTG

GTATCCAAAGTCTAATTCCTGAGTTTCCAAAGCATTTGAGGTGGAAAACATGATTTCACC

ATTAATAGTGGATGTTTTTGGGGGATTGAATGTAGAACTATCCCAACAGTCAGTAGTGAA

GCTAGGCATGTAACATTTTCCAATGTTCTTACGTGCTTCACGTTCATCATTTTGAACAAT

TAGTGGCATGTTGATAGAGCAAGAGGATTGTGTAGATGAGAAATTCATAGAACCATTATG

AAGTGAATAATTGGAGAAAAACTCAGCAGGGGAACTCTTGTGCCTAATGAGATTGGAGCA

ATTCCTAGTAGAAGAATCAATGTTAGTTTCTGTGTCATAATTCTCTGAAGCCAAAAATGA

AGCATCAAAATCGTTAAGCAATCCTTGATTCTTCTGAGTCTGGTAAATTATCTGATTTGA

GGATAATACATAAGAAGAGTAACCATTGTAGTGTGATGAAAAAGGTTCATTTTCTTCTTG

TTTCACTAACACATGTTGAGAATCGGTAAACATTGTTTCCATTTCTGAACTTGTGGAGGG

ACAATAATATTGCTGATTATCATTTCTGAAAGTTTCTTGATTCATAAAACCAATGGTTGT

ATCAACAAGGCTTGACTCAAATTCAGAATAGTTGAGAG

>LsbHLHR88

AAACCACACGTAAGAACTACTTCCAGCGAAGAACCAAATTCATGAATTTCGAAACATGGC

GATTTCGGGACACCAAAACAACATTCACGCGATCTTTTCTTCTTCTCCATCAACAATCTC

TCTTTCTTCTCCTCAGCCATCTTCACATTTGCCTCTAAACTCTTAATGTAGTTTATGGCT

TCTTCCACTTGGTCCGGTAACGCCAACGGCAACTCCTTGGGGTTATAGTTAGGGAGAAGA

GAGTTGAGTTTGGTGTAGAGAATCTTCATCTGATTTCTTCTGTTTTTCTCCACAAGTCTT

CTTTCAACTTTGTTTGTAGAGGTAGAAGGTTGAT

>LsbHLHR89

AAAAAAAAAAAAAAAAACGATAATGAAGAAAGGTCATTGCTTTAGACTAAATAGTCATGA

GTCAGAAAATTCGGATGCCTTGAAAATTGATGTTTTGAATCAAATGAGCTGCTTCAAAAC

CTGATTTATGCTTCTTATCAGATCTTTTATCGTCAAGCAATATTCTTTATTCACCTCAGC

AACAATGGTTATGTCAATATACTTTTTTCCAAATGGTAAAACGGAACTGCTCTGAATGGT

GAGATGATGTTTTTCGAGTTTGTTGAGTATTGCGGTCGCACTGCGACCGTGGTGTTTTTC

ACAGTGGATTCTGATAAGCATGTGTTTACCAGAAACTCTTGCTTCGATTTTTGGAAGTGA

TTGGTCTGAGTTTTCGTTGAAAGAGGAGGAGCTGTCATCGTTGGCAAAGAGAATAGATCT

CTTCACAAAAACTGCGGATTCGACTTTTGTATCGGCTACTTGTTCCTCTAAACTCTCCAC

TCGTTCTTTTAATTGCTTTAGATGAATTATAGCATCCTCCAAAATAGTTGCCTTGTCCAT

CTTTTTGAGTCCAGGAAGAATGGAGGAAAGAGTAATGAATTTCTGGTTAAGCTTTTGCCT

TCTTTTTCTTTCGGCTATTACATGATCTCTTGCTTGTATCGGGTTTCTGTTTGTTATCGT

TGTCGCAGCCTTGTTCTCTAGTTTATCATACTCACTTCCAAAATTCAAGTTTTCACCGTA

CCCCGACTCGGTTTTAGGCTTCTTAATCCTTGCATCCATATTATCAAAGGAAATTATTTT

GGAAGATGATGAAGCCTTGAGTTGTAGCTTTGTTGGTGGTGTTGCAGAGGCGACAGATTG

ATCAGGACTTTCAATGGGGAAAGAGTTGTTTTGTGTGCTTTCTGAATGAAATCTCTTATT

ATTATTATTATGAGAGGAATTAGTACTCTCAGGTGTGGATGTTTCTGAGAAAGAGTTGAA

GTATGAAATATCATCAAAGTTGAATGCAAATGAATCCATTTCTTGAAACAAACTAGTATC

TTCCATTCCAAATTCAGGCATGTAATTGGAAGACGAAATTTGAATCATCTTTGAGATTGA

ATTCCTATATGTACTTGGTGGAGGAAAAGTAGATGAAGAAAAGGAAGAAAGTGTTGTTGT

T

>LsbHLHR90

AAAAAACATTATGGATCCATTTTTTCCTGAGAATCTTCTTCTAGGTGATGTTTTCTGGGA

TGAACCTCTTCTTTCAACAACTAATTTTGTTCAAACACAACAACCTTGTCCTATTCAAAA

CCCAAGTGCTTTTGTTCAGTATAGAGACCAACCCAAGATTAGTTTAGGAAAACAAAACTC

TCTAAAAGGGTCCAATTCACATTCCATGAACAAGAGAATGTTTGCATTTTTGAGAAAAAG

TTTGCCGTCAGAGAGAATTAAAGCAGCTGAATGTGAAAGAGAACGTGGCTTCAAACACAT

GATTAGTGAAAGAATGAGAAGACAAAGACAGAGACAGTGTTGTTCCAACTTACATTCAGT

TTTACCTCATGGAACCAAAACAGATAATAATTCAGTAGTGCAAACAGCAGCTAAGGAAAT

TCAAAGGCTGCAAGGATGCAAAGAGGAGTTAGAGAGGAAAAACTTTGTGCTTGAAGGAAA

TATCGAGGGAAGAAAATTGCAGCATTTGAGAGTGACATATTCATCTACATCTGGAATTGA

TTCTATAGTGGAGACACTGAAATTGTTGAAAGGACATGGTGTGGATACTAGAAGGGTCAA

GACAGATTTTTCTCAGCAGGAGTTTTTTGCAGTCTTAGAGATAGAAACTGAGATTGCAAG

AGCTGACGTGGAAGAGGCTGTTAAG

>LsbHLHR91

TTGAGAGCTTGATAGATAAAGACTCAGATAAAATCAACGGCGAGAAACCAAAGGTGGAGA

GAAAATCAACAGAAGCATGCAAGAGTCACAGGGAAGCTGAAAGAAGACGCAGACAAAGAA

TCAACAACCATCTCTCCACACTTCGGTCTCTCCTCCCCAATACCGCCAAGTCAGATAAGG

CGTCGTTGCTAGCGGAAGTGGTGCAGCACGTGAAACGGTTACGGAAGGAAGCTGATGTGG

TAGCGAATCGTTGGAATGATGAACCGTCGTCTTCGTGTAGTGGAGAGCCGGGTTCGGTTG

TTTCTGGGGAGGAAGCTGAGGCATGGCCGTTTCCGGGAGAATCGGACGAGGCAACGGTGG

GTTGTTGCGGCGAGGAGGAAGGGGGAGCTCGGAGGATGAAAGTGACGGTGTGCTGTGAGG

ACCGGCCCGGACTGAACCGGGATCTGGCACAAGTGATCCGGTCGGTTCGAGCGAACCCGG

TTCGGGCGGAGATAATGACGGTTGGGGGGAGGAGTAAGAGTGTGGTGGTGGTGGAGTGGG

GTGATGATGGAAGAGAGGGGAAGGAAGTTGAAGCGTTGGAAAGGGGTTTGAAGGCGGTGA

TGGAGAATCGGGCTTTTGTGGATTCTGGAATGGGCCCATTGTTGTTGGGCCGTAA

>LsbHLHR92

AACAAGAACCAGGACAGTCAAAACATGTTGGTGGTCGATGTTGTTTACCAAAGACATTCA

TTCTGTCGTTTGATAACTCAACTGTGATACCTGCTACTACATCTCAACCTTGTGTCAATT

CGGAAGCTAAACGTGATTCGAAGAATAAACGGAGCCGTGAAAGTAGTGAAAAGATGAAAA

GAAATGAAGAAAAAGTAGTGAAAAAAGGTAGAAACAGTTTTCAGTGTGCTGATCATATAA

TCGCAGAGAGAAAACGGAGACAGGAATTGACGGAGAGATTCATCGCGCTTTCAGCAACCA

TTCCCGGATTGAGCAAGACGGATAAAGCTTCTATACTTCGAGCAGCAATTGATTATGTGA

AACAGCTTCAAGAACGTGTTCATGAGCTAGAAAAACAAGACAAAAACGTTGGTGTCACAT

CAATGATGGTCCTCAACAAACCTAATCTACGTGGAATTATTAACAATAATGAAGGAGACA

AAAATTCTGGTGAAACGAGTAGTGATGATGATGATTGTAACAAAAACATCCTCCCAGAGA

TTGAAGCAAGAGTTATGGGAAAGGAAGTGCTTATTGAAATTCATTGTGAGAAACAAATCG

GAATTGAGCTTAAAGTACTAAAACACATTGAAA

>LsbHLHR93

ATTGGAATCATCAAGTGAGTATGAGGAACCATTTCATAAACAAACATAATGCAGGAACAA

TACATTGATAACCATGATACTAATGTTCCATTGTCATAAAGTTTATGACATAATTATAAC

CTAATTTGTTTATAGTAAGTTTTGTTGCATGTCTCTCACATGCTTTTATTTTATTATCAC

ATGCACGATCCAAATCGAACATGTCTCTCTTAGAAATGAAATATGGTCACACAGCAATCA

ACTTCACAGCCTCTGAACAAAAGCTTTGTTGAACTTCCATAGCTAGTTCCTCCAAACTTA

GCTGACATCCCAGTTCTATCTTGACTACAAATTGGTATAGAACAGTCTCTTCCATGCTAC

TGATATTAAGATGAAGAACTTCAAAAGAAAAACTTTCTAAAACGGTTATAATCCTTGAAA

CTTGACCCGGAATTCTTTGAGATATCACTCTTAAAATCACATTTGGACCTGAGATTTTCA

CTTCTACATCTGCAACTGAAGAATTGCAGCTTGCTCCTAGCTCCTTGAAAGTATTGTTAG

CCTCAATTCCTCCGGAGGAGATATCAAATTGATGAAAAGTTGGTTGCAGTGTTTTTGGAC

TTGGATTAGGGCTAGGACTTAAACTCTTCCTTCTCTTTTGAGATTCCAAAGATTGAAGAA

CTTGGTGCAACTCATTGATGAATTCTATAACTCCCCCTATTATTGATGCTTGGTCTCCCC

TTTTAATATAGAAACAAGGGGTCAAGGACCTTAAAACTTTGAGATGTTCATTCATCTGTC

TTCTTCTGTTCCTCTCAACAGCTATATGAGACATGATCAATTTGGTTCTAGAAAACTAAG

GTTGATCGTTTTTTTTTTAT

>LsbHLHR95

CTACGTTGCTCAAACAAGACCAACCAAGAAGATCAAAACATCTATAAACCCTTCTTCCTA

TTCGTCACCTCAACTCATTTCTTTTGAGCACTTCAATGCAACGCCGGTTGCTTCTAACGA

ACTTTACAACCTCGATTATTCTGATGTAAAGCCTAAACTCGAGAAAGGGTGCAATGAAAA

CAAAGATTTTGCCGCAAATTATGATATCCGAGCAAATCAGACGAGGAACACGGCTCAAGC

AAAGGAACATGTAATGGCTGAAAGAAAACGAAGGGAAAAGCTCACCAGAAGTTTCATAGC

TCTTTCTGCCATCCTTCCTGGCCTCAAGAAGATGGATAAGGCTTCTGTGTTGGGAGATGC

AATAATGTATATGAAAAAACTACAAGCACGTTTGCATACACTAGAGGCACAAGTTGAAGA

CAACAAAAAAACTGGATCTGCAATTCAAGTGAAGAGATCTGTTATCTTCTCCACTGATGA

TCATGATGATGACAGCAACTCCAACAACCAGACACTTCCTGAAATCGAAGTAAGAGTTTC

AAGAAAAGATGTACTCATCAAAATTCAATGCGACAAACACAGTGGACGTGCCACTACCTC

TACGGTACTCGGAAAATTAGAAACCCTAAATCTCACTGTCCAAAGTAGTAACTTATTGCC

CTTCGGAAACAACATTGTTGATCTAACTATTGTTGCTCAGATGAACGAGGAAAACTGTGT

GACAGCAAAGGATCTCCTAGGAAGCATACGACATGCATTAATCACCAATAAATAA
